# Diastereoselective C–H carbonylative annulation of aliphatic amines: a rapid route to functionalized γ-lactams†
†Electronic supplementary information (ESI) available: Experimental procedures, characterization data and kinetic details. CCDC 1850700–1850701. For ESI and crystallographic data in CIF or other electronic format see DOI: 10.1039/c8sc02855a


**DOI:** 10.1039/c8sc02855a

**Published:** 2018-07-31

**Authors:** Zhuang Mao Png, Jaime R. Cabrera-Pardo, Jorge Peiró Cadahía, Matthew J. Gaunt

**Affiliations:** a Department of Chemistry , University of Cambridge , Lensfield Road , Cambridge , UK . Email: mjg32@cam.ac.uk

## Abstract

A palladium(ii)-catalysed C(sp^3^)–H carbonylation of free(NH) secondary aliphatic amines to 2-pyrrolidinones is described. A correlation between the nature of the carboxylate ligand and the diastereoselectivity and yield of the process was observed. As such, under these optimal conditions a range of aliphatic amines were converted to the corresponding trans-4,5-disubstituted 2-pyrrolidines with good d.r. and yield.

## Abstract 2

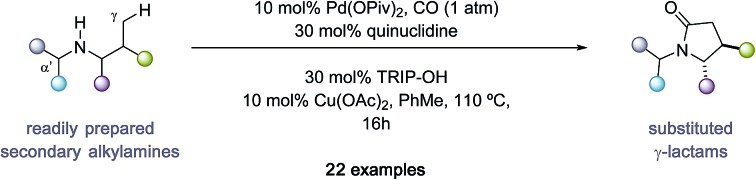
A palladium(ii)-catalysed C(sp^3^)–H carbonylation of free(NH) secondary aliphatic amines to 2-pyrrolidinones is described.

## 

Five-membered ring saturated nitrogen-containing heterocycles are ubiquitous structural features in alkaloid natural products, small-molecule biological probes and pharmaceutical agents (Fig. 1a).^1^ Among these scaffolds, 2-pyrrolidinones represent a synthetically versatile class of heterocycle due to the diverse reactivity imparted by the amide functional group. The majority of transformations that generate the 2-pyrrolidinone scaffold can be mainly divided into two reaction classifications: cyclization of bifunctional substrates^2^–^5^ and transformations of appropriately functionalized 5-membered ring core.^6^ Of particular importance to the successful realization of these processes is the need for reactive functional groups resident within the framework of the precursors to the nitrogen heterocycle. Given the relevance of these saturated nitrogen heterocycles, the development of new streamlined catalytic methods for their construction from simple precursors remains a continual challenge in chemical synthesis.

**Fig. 1:**
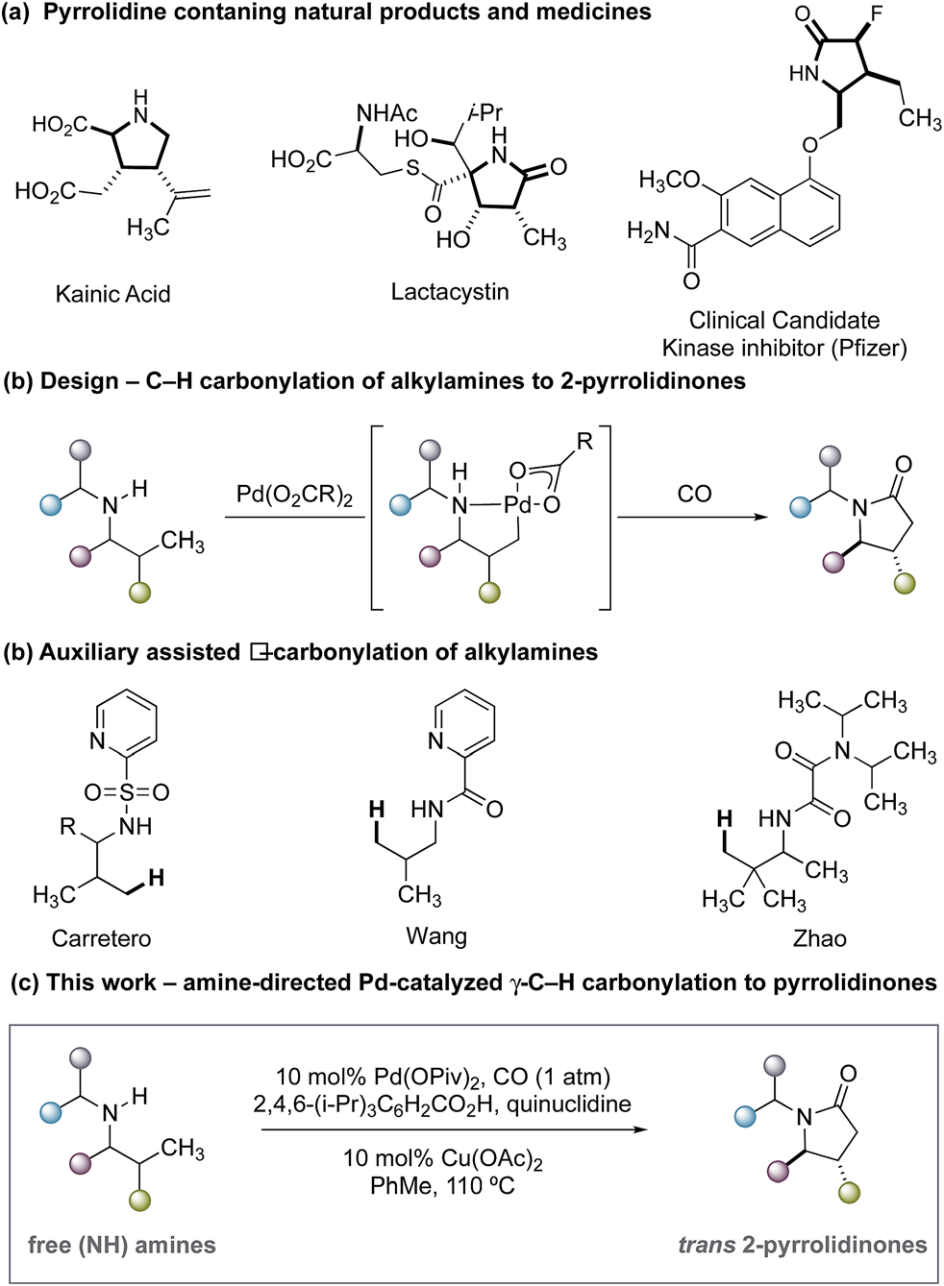
Overview of γ-C–H carbonylation of aliphatic amines.

As part of an ongoing research program in our laboratory towards establishing secondary alkylamines as viable feedstocks for catalytic C–H activation-based synthesis strategies,^7^ we reasoned that a single step process capable of forging 2-pyrrolidinones from carbon monoxide (CO) and unfunctionalized secondary alkylamines would represent a useful synthetic transformation.^8^,^9^ Formally, such a process to form 2-pyrrolidinones would require the insertion of CO into the C–H and N–H bonds of an unactivated secondary alkyl amine *via* a dehydrogenative ‘4 + 1 cycloaddition-type’ process. While important independent work by Carretero,^10^ Wang^11^ and Zhao^12^ on Pd-catalyzed C–H carbonylation of auxiliary-derived alkylamines has enabled the synthesis of some 2-pyrrolidinone-derived scaffolds (Fig. 1b), the use of more tractable unprotected secondary alkylamines is unknown and has presented a number of significant hurdles to the development of an effective process. This methodological gap is mainly due to the strong binding of unprotected secondary amines to electrophilic Pd(ii) salts, forming thermodynamically stable and off-cycle bis-amine Pd(ii) complexes that ultimately preclude catalysis.^13^ Despite this, the streamlining advantages and late-stage functionalization opportunities offered by C–H methods that directly utilize unprotected secondary alkylamines means that the development of appropriate catalytic transformations is an important synthetic goal. In light of these challenges, our group developed a C–H carbonylation strategy wherein the rapidly formed bis-amine Pd(ii) complexes were destabilized by intense steric interactions imparted by ligated hindered amine substrates and led to higher concentrations of the putative mono-amine Pd(ii) complexes empirically required for C–H bond cleavage.^14^ Furthermore, we identified that a hydrogen bond between the NH group of the ligated amine and the carbonyl group of the acetate ligand played a crucial role in orienting the amine such that C–H activation is facilitated.14*c* While we have developed a number of distinct transformations based on this sterically-controlled C–H activation pathway, a notable limitation of these methods has been the reliance on using substrates that display fully substituted carbon centers around the carbon–nitrogen bond of the amine.^14^ We questioned whether a γ-C–H carbonylation process to form 2-pyrrolidinones could be developed for less substituted secondary alkylamines by using ligands on the palladium catalyst to minimize the formation of the off-cycle bis-amine Pd(ii) intermediates and the deleterious amine degradation that often plagues oxidative transformation of secondary alkylamines displaying α-C–H bonds.^15^ Furthermore, we saw the opportunity to deliver a diastereoselective C–H carbonylation process, which would provide a direct route to substituted variants of the synthetically important 5-membered ring lactams; previous examples of auxiliary-controlled C–H carbonylation to 2-pyrrolidinones generally display low diastereoselectivity, which limits their synthetic utility.^10^–^12^ Herein, we detail the successful realization of these ideals through the development of a diastereoselective palladium-catalyzed C–H carbonylation of unprotected secondary alkylamine to form *trans-*4,5-disubstituted 2-pyrrolidinones (Fig. 1c). We identified that a carefully balanced mixture of sterically hindered carboxylate and ligands afforded a diasteroselective C–H activation step and synthetically practical carbonylation process. Interestingly, the ligand cocktail is also capable of influencing the mechanism of the reaction by subtly controlling the position of carbonylation at either the β- or γ-C–H bond with respect to the directing amine motif.

At the outset of our studies, we selected amine **1a** as a representative substrate which would probe both the viability of a catalytic C–H carbonylation as well as the diastereoselectivity of the process. Guided by conditions previously developed for C–H carbonylation with sterically engineered alkylamines,14*d* we found that treatment of **1a** with 10 mol% Pd(OAc)_2_, 200 mol% AgOAc in toluene at 100 °C and under a 2 bar atmosphere of CO (100 °C, 16 h) delivered *trans*-4,5-disubstituted γ-lactam **4a** in 30% yield and 7 : 1 d.r. (Table 1, entry 1). We were surprised to find that changing the oxidant from AgOAc to 10 mol% Cu(OAc)_2_ increased the observed diastereoselectivity of the reaction, although the yield remained modest (Table 1, entry 2). Further improvements to the diastereoselectivity could be realised by changing the catalyst to Pd(OPiv)_2_ and increasing the reaction temperature to 110 °C; despite the improvements in selectivity the yield of the reaction remained low (Table 1, entry 4). Next, we studied the impact of introducing external ligands and carboxylic acids additives to the Pd(OPiv)_2_/Cu(OAc)_2_ catalyst system. Previous work in our group has shown that ligands and carboxylic acids additives enhance C–H carbonylation of aliphatic amines.16*a*

**Table 1:** Selected optimization^*a*^

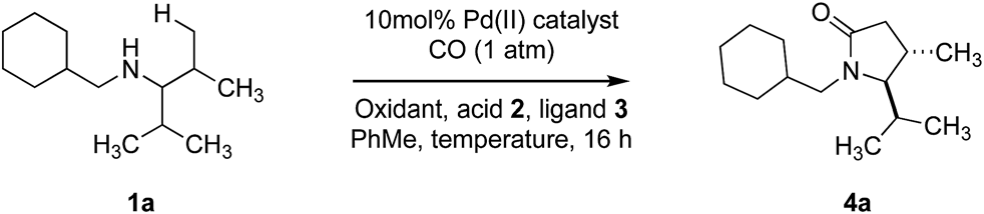						
Entry	Catalyst	Oxidant^*b*^	*T*, °C	Acid^*c*^	Ligand^*c*^	Yield % (d.r.)
1	Pd(OAc)_2_	AgOAc	100	—	—	30 (7 : 1)
2	Pd(OAc)_2_	Cu(OAc)_2_	100	—	—	30 (11 : 1)
3	Pd(OPiv)_2_	Cu(OAc)_2_	100	—	—	30 (15 : 1)
4	Pd(OPiv)_2_	Cu(OAc)_2_	110	—	—	35 (17 : 1)
5	Pd(OPiv)_2_	Cu(OAc)_2_	110	—	**3a**	37 (11 : 1)
6	Pd(OPiv)_2_	Cu(OAc)_2_	110	PhCO_2_H	—	40 (13 : 1)
7	Pd(OPiv)_2_	Cu(OAc)_2_	110	PhCO_2_H	**3a**	50 (11 : 1)
**8**	**Pd(OPiv)**_**2**_	**Cu(OAc)**_**2**_	**110**	**2a**	**3a**	**70 (10 : 1)**
9	Pd(OPiv)_2_	Cu(OAc)_2_	110	**2b**	**3a**	81 (5 : 1)
10	Pd(OPiv)_2_	Cu(OAc)_2_	110	**2a**	—	39 (11 : 1)
11	Pd(OAc)_2_	Cu(OAc)_2_	110	**2a**	**3a**	48 (8 : 1)

^*a*^Yields were determined by 1H NMR analysis with 1,1,2,2–tetrachloroethane as internal standard. Diasteromeric ratios (d.r.) were determined by GC-MS.

^*b*^10 mol%, except entry 1, 200 mol%. 2a = 2,4,6-triisopropylbenzoic acid. 2b = 2-methyl-6-nitrobenzoic acid. 3a = quinuclidine.

^*c*^30 mol%.

Although addition of quinuclidine **3a** or benzoic acid to the reaction mixture showed little effect on the outcome (entry 5–6), a positive synergistic effect was observed by adding these reagents together with the yield increasing to 50% and the d.r. remaining at 11 : 1 (*trans* : *cis*) across the 2-pyrrolidinone core (entry 7). Further screening of different carboxylic acid additives^17^ showed that the sterically encumbered 2,4,6-triisopropylbenzoic acid **2a** (TRIP-OH) was the best additive, delivering the γ-lactams in both high yield and good diastereoselectivity (entry 8). Electron deficient 2-methyl-6-nitrobenzoic acid (**2b**) was also notable for delivering the desired product in the highest yield of 81%, although the diastereoselectivity was lower (entry 9). When TRIP-OH was used in the absence of quinuclidine, the yield dropped significantly (entry 10). Another control experiment, using palladium acetate instead of palladium pivalate, also decreased the yield to 48% (entry 11). While we found that no further increases to the diastereoselectivity were observed, we were able to significantly improve the yield of the reaction, accompanied by a minimum reduction in selectivity. At this point, despite our best efforts, we remain uncertain of the precise role the different additives and ligands play in influencing the yield and diastereoselectivity of the reaction.^18^ However, Zhao have previously shown that electron deficient benzoic acid additives improve the yield of the reaction by stabilizing Pd(0) in the catalytic cycle and our results reinforce these observations.^12^ In the end, we compromised between diastereoselectivity and reaction yield, opting for optimal conditions which involved treatment of amine **1a** with 10 mol% Pd(OPiv)_2_, 10 mol% Cu(OAc)_2_, 30 mol% quinuclidine, 30 mol% 2,4,6-triisopropylbenzoic acid under a CO/air atmosphere at 110 °C in toluene for 16 h, which yielded *trans*-substituted lactam **4a** in 70% yield and a 10 : 1 diastereoselectivity. The reaction was also successful on a 5 mmol scale (1.05 g of **1a**), producing lactam **4a** in 69% yield.

With the optimal conditions in hand, we next examined the scope of the new γ-C–H carbonylation process. We found that a range of structurally and functionally diverse amines underwent efficient C–H carbonylation to access 4,5-disubstituted γ-lactams in synthetically useful yields and good diasteroselectivities (Scheme 1). First, we assessed the effect of substituents on the non-reacting side of the free (NH) motif. Branching at the α-position on the non-reacting side of the amine was well tolerated and provided the corresponding γ-lactams in good yields and d.r. (Scheme 1, **4b–l**). These substituents included different sized carbocycles, tetrahydropyran, protected piperidine and fluorocycloalkanes, all of which could be tolerated in the reaction to deliver the desired lactams efficiently (**4d–f**). Acid sensitive ketal-protected ketones and 1,3-diols were also accommodated in the γ-C–H carbonylation process without any deleterious hydrolysis (**4g,h**). A pharmaceutically-relevant heterocyclic derivative was also converted to the corresponding γ-lactam product **4i** in moderate, but still synthetically useful yields to access to amino-azetidine cores. Replacing the branched (non-reacting) substituent with linear functionalized alkyl groups also enabled an effective reaction, with the corresponding γ-lactams produced in reasonable yield and good diastereoselectivity (**4j–l**). A notable feature of this C–H carbonylation process is its capacity to accommodate the presence of Lewis basic heteroarenes, which frequently interfere in related C–H transformations due to competitive coordination to the palladium catalyst.

**Scheme 1:**
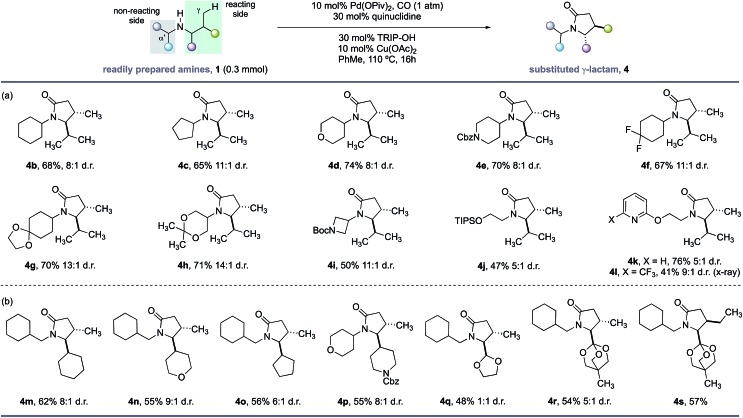
Scope of γ-lactams *via* C–H carbonylation. (a) Functional group tolerance. (b) Lactam substitution patterns.

A range of functional groups can also be incorporated on the reacting side of the amine, providing a means to introduce different functionalities on the γ-lactam scaffolds (Scheme 1, **4m–s**). For example, amines containing 5- and 6-membered ring cycloalkanes, tetrahydropyrans and protected piperidine motifs adjacent to the nitrogen atom delivered the γ-lactams in reasonable yields and diasteroselectivities (**4m–p**). Surprisingly, an acetal substituent imparted no diastereoselectivity in the C–H carbonylation reaction, whereas the corresponding orthoester derivative gave a 5 : 1 mixture of diastereomers under the same reaction conditions suggesting a subtle steric influence of the substituents in this position on the stereoselectivty (**4q–s**). A derivative of isoleucine presented a competition between γ-methyl and γ-methylene C–H bonds; C–H carbonylation was only observed at the γ-methyl group and formed lactam **4s** in good yield as a single stereoisomer.

Previous work from our group has shown that under a set of highly optimized reaction conditions a variety of alkyl substituted secondary amines can be converted into β-lactams through a Pd(ii)-catalyzed methyl or methylene β-C–H carbonylation process involving a putative Pd(ii)-carboxamide intermediate even when more commonly activated γ-C–H bonds were available.^16^ For example, when amine **1t** was treated with 10 mol% Pd(OAc)_2_, 10 mol% xantphos, AgOAc, benzoquinone and CO at 80 °C in a toluene solution (conditions previously shown to proceed *via* methylene β-C–H activation) only the corresponding β-lactam **5t** was observed in an unoptimized 40% yield (Table 2). Under the standard conditions for γ-C–H carbonylation (*vide supra*), we observed the appearance of the γ-lactam product **4t** but as the minor product to the corresponding β-lactam **5t** (0.4 : 1), Table 2, entry 1. Interestingly, by simply changing the acid additive from TRIP-OH to nitrobenzoic acid derivative **2b**, we found a reversal of the selectivity with the reaction now producing the γ-lactam (Table 2, entry 2). A similar result was also observed when the concentration of CO in the reaction was lowered through the use of a commercial 6.25% CO/air mix, wherein a 1.6 : 1 mixture of γ : β-lactams was observed (Table 2, entry 3). However, we were pleased to find that the amount of β-lactam could be reduced to almost trace amounts under reaction with the same low CO concentration and by using nitrobenzoic **2b** as additive; under these conditions a 29 : 1 mixture of lactam isomers (Table 2, entry 4) was obtained highlighting that manipulation of the reaction conditions results in a change in the mechanism of the C–H activation pathway. We also demonstrated the selective C–H carbonylation could be applied to other amine substrates selectively forming the γ-lactam products with only trace amounts of the β-lactams observed by GC-MS assay (Scheme 2).

**Table 2:** Controlling regioselectivity in C–H carbonylation

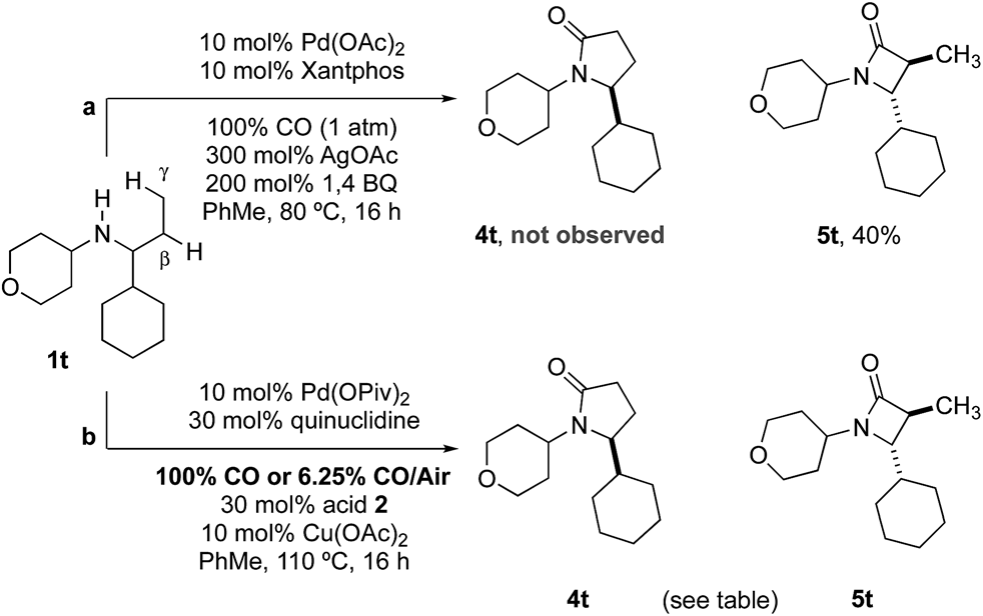					
Entry	Acid	CO source	**4t**, %	**5t**, %	**4t**/**5t**
1	**2a**	100% CO	11	26	0.4
2	**2b**	100% CO	41	25	1.6
3	**2a**	6.25% CO/Air	36	23	1.6
**4**	**2b**	**6.25% CO/Air**	**50**	**1.7**	**29**

**Scheme 2:**
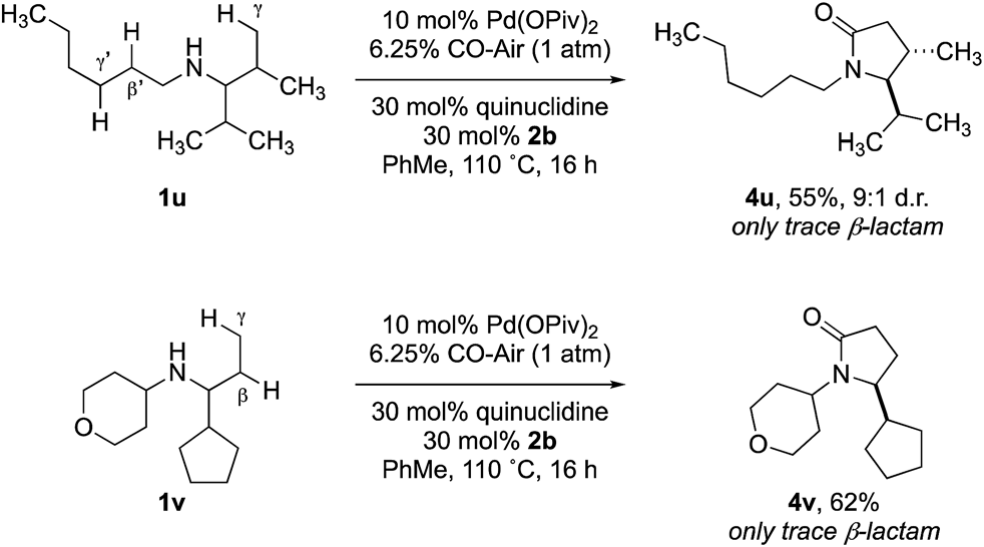
Selective carbonylation between β and γ C–H bonds.

Yields were determined by ^1^H NMR analysis with 1,1,2,2–tetrachloroethane as internal standard.

To demonstrate that the γ-carbonylation proceeds *via* a ‘classical’ cyclopalladation/carbonylation pathway rather than γ-C–H activation through a Pd(ii)-carboxamide-type species, we showed that reaction of amine **1f** with 1.5 equivalents of Pd(OAc)_2_ gave γ-C–H activation to form the ‘classical’ five-membered ring palladacycle **int-I** as a 10 : 1 mixture of diastereomers (Scheme 3a). The structure of this complex was confirmed by analysis of the X-ray diffraction pattern of a single crystal.^17^ To rationalize the diastereoselectivity of the reaction, we propose that selection of the two diastereotopic methyl groups is controlled by a destabilizing interaction between the non-reacting methyl group and vicinal isopropyl substituent, which therefore, favours reaction through **int-IIb** that lacks the steric clash between the two groups (**int-IIa**, **int-IIb**. Scheme 3b).^19^

**Scheme 3:**
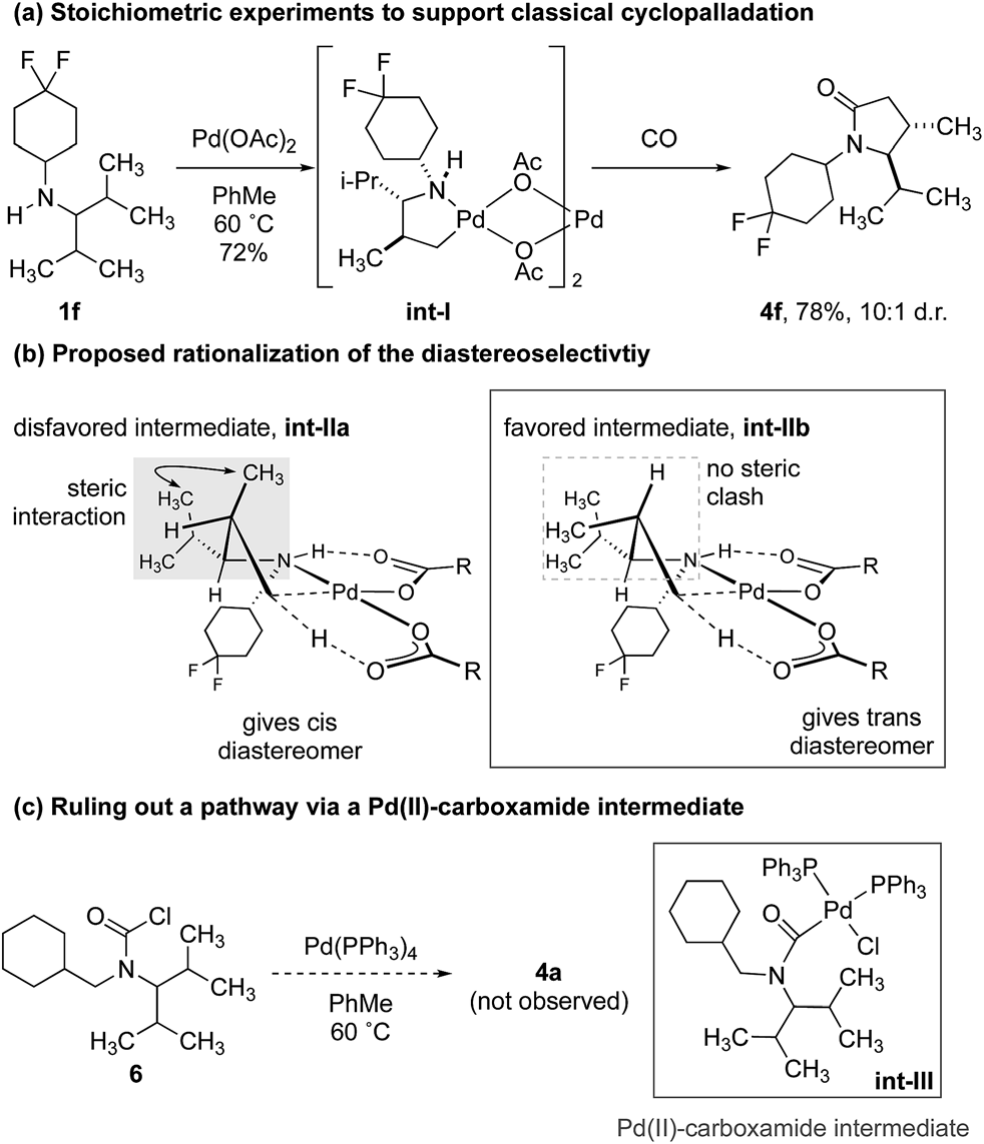
Initial experiments to probe the mechanism of reaction.

In turn, subjecting **int-I** to an atmosphere of CO gave the γ-lactam, again as a 10 : 1 mixture of diastereomers. Conservation of the diastereomeric ratio is important because it suggests that the C–H activation is not reversible under a carbon monoxide atmosphere, as has been observed for the C–H carbonylation of some hindered alkylamines.16*a* To rule out that the reaction was proceeding *via* a Pd(ii)-carboxamide intermediate, previously identified in our C–H activation reactions to β-lactams,^16^ we prepared the carbamoyl chloride **6** and found that its treatment with a Pd(0) source did not result in any lactam products, which suggested that the reaction to γ-lactam does not proceed *via* such a Pd(ii)-carboxamide intermediate (Scheme 3c).

Finally, we demonstrated that the γ-lactam products could be transformed into further useful building blocks (Scheme 4). Reduction of **4a** delivers the corresponding *trans*-2,3-disubstituted pyrrolidine (**7**), a scaffold that is not trivial to access conveniently by other methods. Enolate alkylation can also be used to functionalize the 3-position in diasteroselective fashion, leading to 3,4,5-trisubstituted 2-pyrrolidinones **8**. Fluorination with NSFI produced the corresponding 3,3-difluoro-2-pyrrolidine **9**, a compound whose structure is related to a component of a Pfizer compound able to inhibit kinases (IRAK4) at nanomolar potency in cell-based assays.^20^

**Scheme 4:**
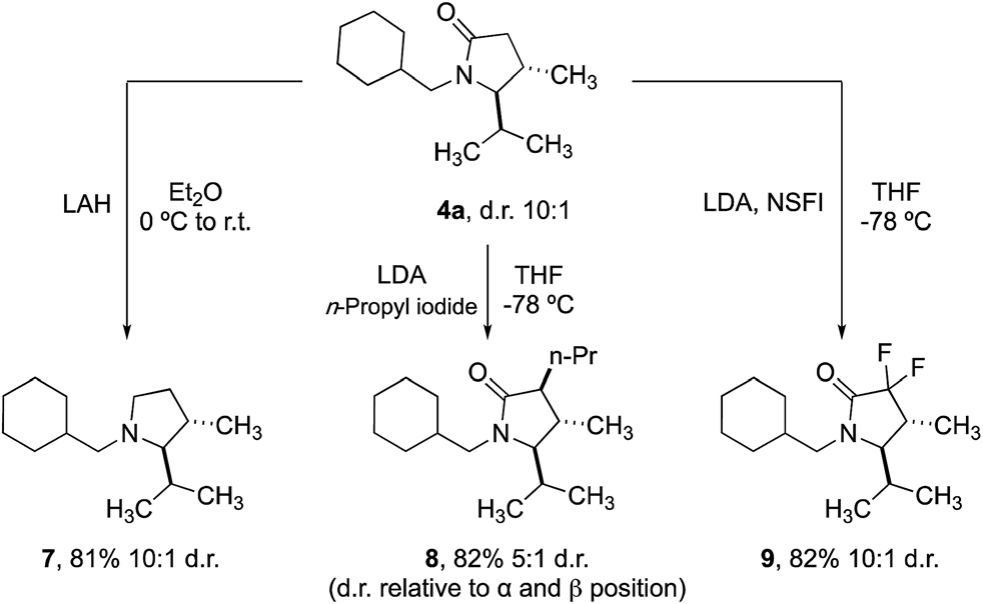
Derivatization of lactam scaffolds.

In summary, we have developed a diastereoselective C–H carbonylation process converting unprotected secondary aliphatic amines to γ-lactams. We believe this reaction proceeds *via* a classical five-membered cyclopalladation, with the yield and diastereoselectivity strongly influenced by a cocktail of carboxylate and amine ligands. In addition, these additives are capable of influencing the mechanism of the C–H carbonylation process. The reaction can accommodate a range of functionalities and substitution patterns in the amine appendages that we believe will be of significant interest to synthetic and medicinal chemists.

## Conflicts of interest

There are no conflicts to declare.

## Supplementary Material

Supplementary information

Crystal structure data
