# Supplementary material for: Diastereoselective C–H carbonylative annulation of aliphatic amines: a rapid route to functionalized γ-lactams
Source: Chem Sci. 2018 Jul 31;9(39):7628–33. doi: 10.1039/c8sc02855a (PMC6182607; doi:10.1039/c8sc02855a)

Electronic Supplementary Material (ESI) for Chemical Science.
This journal is © The Royal Society of Chemistry 2018

Supplementary Information

General experimental procedure 1

Experimental conditions for carbonylation 2

Synthesis of amines 2

Carbonylation of amines 23

Functionalisation of lactams 37

Palladacycle studies 39

Selected optimisation studies 41

References 45

Proton and Carbon NMRs 46

1.1 General Experimental Procedure

All reactions were conducted using standard techniques using oven-dried glassware. Anhydrous solvents were obtained from solvent stills (DCM and toluene were distilled over CaH_2). Commercial reagents were used as supplied from Sigma Aldrich, Alfa Aesar, or Fluorochem, except for 1-4-benzoquinone, which was recrystallized from hexane and stored in the dark.

Thin-layer chromatography (TLC) was performed on Merck Kiesegel 60 F254 0.20 mm precoated, glass backed, silica gel plates; visualisation was achieved by aqueous $KMnO_4$. Flash column chromatography was performed using silica gel (Merck Geduran Si 60 [40-63 μm]) with the relevant solvent.

Nuclear magnetic resonance (NMR) spectra were recorded on a Bruker DPX 400 with chemical shifts (δ) reported in ppm from TMS with the $CDCl_3$ as internal standard (^{1}H, 7.26 ppm; ^{13}C, 77.16 ppm). Data is reported as follows: chemical shift (integration, splitting (s = singlet, d = doublet, t = triplet, q = quartet, qt = quintet, sxt = sextet, spt = septet, m = multiplet), coupling constant, assignment).

Infrared spectra (FT-IR) were recorded using a Perkin-Elmer Paragon 1000 Fourier transform Spectrometer equipped with ATR and analysed as neat thin films, with absorption maxima (λ_{max}) being quoted in wavenumbers (cm^{-1}). High Resolution Mass Spectrometry (HRMS) was carried out by the ESPRC Mass Spectrometry Service at the University of Swansea using an LTQ Orbitrap XL spectrometer with positive ion nano-electrospray.

1.2 Experimental conditions for carbonylation

Various conditions involving carbon monoxide at different concentrations are described in this work. Where relevant, the following conventions are used to describe experimental techniques. “CO” refers to 100% CO, with evacuation and backfilling of the reaction vessel with three times. “CO balloon” refers to 100% CO being used, but with no evacuation and backfilling. “6.25% CO / air” refers to the commercially available mixture being used, with evacuation and backfilling of the reaction vessel. “6.25% CO / air balloon” refers to the same mixture used with no evacuation and backfilling. As such, the concentration of CO decreases in the order of CO > CO balloon > 6.25% CO / air > 6.25% CO / air balloon. In all cases, the balloon was left attached to the reaction vessel until the end of the reaction. Reaction optimisation was carried out on a 0.1 mmol scale in a boiling tube with a 12 mm stirrer at 400rpm, sealed with a new B24 suba seal and parafilm. Reaction scope was carried out on a 0.3 mmol scale in a 100 mL RBF with an oval stirrer at 400 rpm, also sealed with a new B24 suba seal and parafilm. The reaction was also scaled to a 5 mmol scale, which was carried out in a 1 L RBF with an oval stirrer at 600 rpm.

1.3 Synthesis of Amines

General Procedure A for Reductive Amination

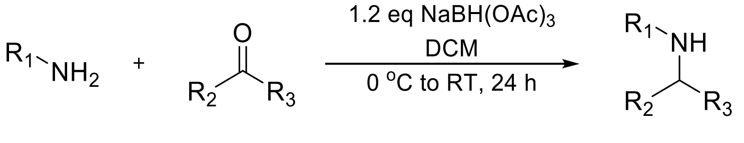

To a stirred solution of amine (1 eq) and ketone / aldehyde (1 – 1.5 eq) in dichloromethane (0.2 M) at 0 °C under nitrogen, sodium triacetoxyborohydride (1.2 eq) was slowly added over 10 mins. The reaction mixture was stirred for 24 hours, warming up to room temperature. 1 M sodium hydroxide was added to quench the reaction mixture until the bubbling stops and stirred for an additional 30 mins. The mixture was extracted with dichloromethane and the combined organic extracts were washed with brine and dried over magnesium sulphate. Removal of the solvent *in vacuo* gave the crude amine. Further purification by kugelrohr or flash column chromatography gave the pure amine as a colourless liquid.

General Procedure B for Reductive Amination

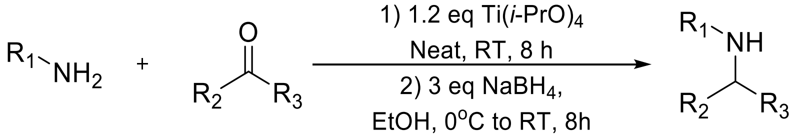

A solution of the amine (1 eq), ketone (1 - 1.2 eq), and Ti(*i*-PrO)$_4$ (2 eq) was stirred neat for 8 hours at room temperature under nitrogen. The mixture was cooled to 0 °C. Absolute ethanol (dilute to 0.25 M) was then added, followed by sodium borohydride (3 eq) portion wise over 10 mins, and the resulting mixture was stirred for an additional 8 hours, warming up to room temperature. The mixture was then poured into 2.5 M sodium hydroxide, filtered, and washed with acetone. The mixture was extracted with dichloromethane and the combined organic extracts were washed with brine and dried over magnesium sulphate. Removal of the solvent *in vacuo* gave the crude amine. Further purification by kugelrohr or flash column chromatography gave the pure amine as a colourless liquid.

N-(cyclohexylmethyl)-2,4-dimethylpentan-3-amine (1a)

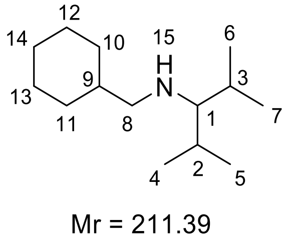

Mr = 211.39

General procedure B was applied to cyclohexylmethylamine (1.1 g, 10 mmol), 2,4-dimethylpentanone (1.1 g, 10 mmol) and Ti(*i*-PrO)$_4$ (5.7 g, 20 mmol) neat. After stirring for 8 hours, the mixture was cooled to 0°C. Absolute ethanol (40 mL) was added, followed by sodium borohydride (1.1 g, 30 mmol) portion wise and the resultant mixture was stirred for another 8 hours. Purification by kugelrohr (155 °C, 3mbar) gave **1a** as a colourless liquid (1.3 g, 61%). IR (λ_{max}/cm^{-1}) 2955, 2921, 2852, 1448, 1116. ^{1}H NMR (400.1 MHz, CDCl$_3$) 2.45 (2H, d, J = 6.7 Hz, H8), 1.80 - 1.63 (8H, m, H10a, H11a, H12a, H13a, H14a, H1, H2, H3), 1.39 (1H, m, H9), 1.29 – 1.09 (3H, m, H10b, H11b, H14b), 0.92 (2H, td, J1= 12.7 Hz, J2 = 3.5 Hz, H12b, H13b), 0.89 (12H, d (overlap), J = 6.6 Hz, H4, H5, H6, H7). ^{13}C NMR (100.6 MHz, CDCl$_3$) 66.47 (C1), 59.26 (C8), 39.10 (C9), 31.68 (C10, C11), 30.90 (C2, C3), 26.83 (C12, C13), 26.16 (C14), 20.89 (C4, C5), 18.20 (C6, C7). HRMS (FTMS +p NSI) m/z: [M + H]$^+$ calculated for $C_{14}H_{30}N$ 212.2372, found = 212.2373, Δ = 0.5 ppm.

N-(2,4-dimethylpentan-3-yl)cyclohexanamine (1b)

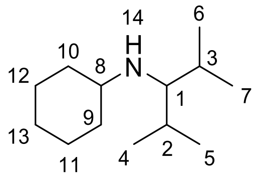

Mr = 197.36

General procedure B was applied to cyclohexylamine (1.0 g, 10 mmol), 2,4-dimethylpentanone (1.1 g, 10 mmol) and Ti(*i*-PrO)$_4$ (5.7 g, 20 mmol) neat. After stirring for 8 hours, the mixture was cooled to 0 °C. Absolute ethanol (40 mL) was added, followed by sodium borohydride (1.1 g, 30 mmol) portion wise and the resultant mixture was stirred for another 8 hours. Purification by kugelrohr (150 °C, 4mbar) gave **1b** as a colourless liquid (1.1 g, 55%). IR (λ_{max}/cm^{-1}) 2955, 2925, 2852, 1465, 1449, 1366, 1112. ^{1}H NMR (400.1 MHz, CDCl$_3$) 2.35 (1H, tt, J1 = 11.1 Hz, J2 = 3.9 Hz, H8), 2.00 (1H, t, J = 5.62 Hz), H1), 1.90 – 1.82 (2H, m, 9a, 10a), 1.77 – 1.66 (4H, m, H2, H3, H11a, H12a), 1.62 – 1.54 (1H, m, H13a), 1.28 – 1.08 (3H, m, H11b, H12b, H13b), 1.08 – 0.94 (2H, m, H9b, H10b), 0.90 (6H, d J = 7.2 Hz, H4, H5), 0.87 (6H, d J = 7.2 Hz, H6, H7) ^{13}C NMR (100.6 MHz, CDCl$_3$) 65.5 (C1) 57.1 (C8), 34.7(C9, C10), 31.0 (C2, C3), 26.2 (C13), 25.5 (C11, C12), 21.1 (C4, C5), 18.4 (C6, C7). HRMS (FTMS +p NSI) m/z: [M + H]$^+$ calculated for $C_{13}H_{28}N$ 198.2250, found = 198.2248, Δ = - 1.0 ppm.

N-(2,4-dimethylpentan-3-yl)cyclopentanamine (1c)

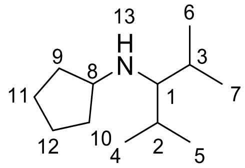

Mr = 183.34

General procedure B was applied to cyclopentylamine (425 mg, 5 mmol), 2,4-dimethylpentanone (550 mg, 5 mmol) and Ti(*i*-PrO)$_4$ (2.4 g, 10 mmol) neat. After stirring for 8 hours, the mixture was cooled to 0 °C. Absolute ethanol (20 mL) was added, followed by sodium borohydride (550 mg, 15 mmol) portion wise and the resultant mixture was stirred for another 8 hours. Purification by silica column (5% to 20% ethyl acetate / petroleum ether) gave **1c** as a colourless liquid (450 mg, 49%). IR (λ_{max}/cm^{-1}) 2954, 2870, 1464, 1383, 1359, 1123, 1066. ^{1}H NMR (400.1 MHz, CDCl$_3$) 3.09 (1H, p, J = 6.8 Hz, H8), 1.97 (1H, t, J = 5.4 Hz, H1), 1.83 – 1.61 (6H, m, H2, H3, H9a, H10a, H11a, H12a), 1.55 -1.43 (2H, m, H11b, H12b), 1.31 – 1.20 (2H, m, H9b, H10b) 0.91 (6H, d, J = 6.9 Hz, H4, H5) 0.88 (6H, d, J = 6.9, H6, H7). ^{13}C NMR (100.6 MHz, CDCl$_3$) 67.1 (C1), 60.5 (C8), 33.7 (C9, C10), 30.8 (C2, C3), 23.5 (C11, C12), 21.1 (C4, C5), 18.4 (C6, C7) HRMS (FTMS +p NSI) m/z: [M + H]$^+$ calculated for $C_{12}H_{26}N$ 184.2060, found = 184.2059, Δ = - 0.5 ppm.

***N*-(2,4-dimethylpentan-3-yl)tetrahydro-2*H*-pyran-4-amine (1d)**

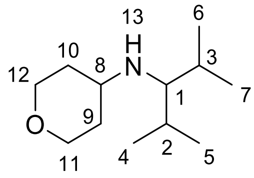

Mr = 199.33

General procedure B was applied to 4-aminotetrahydropyran (1.0 g, 10 mmol), 2,4-dimethylpentanone (1.1 g, 10 mmol) and Ti(*i*-PrO)$_4$ (5.7 g, 20 mmol) neat. After stirring for 8 hours, the mixture was cooled to 0 ^{o}C. Absolute ethanol (40 mL) was added, followed by sodium borohydride (1.1 g, 30 mmol) portion wise and the resultant mixture was stirred for another 8 hours. Purification by kugelrohr (120 ^{o}C, 5mbar) gave **1d** as a colourless liquid (0.8 g, 41%). IR (λ_{max}/cm^{-1}) 2954, 2839, 1466, 1383, 1363, 1236, 1120. ^{1}H NMR (400.1 MHz, CDCl$_3$) 3.99 – 3.92 (2H, m, H11b, H12b), 3.36 (2H, td, J1 = 12.0 Hz, J2 = 3.0 Hz, H11a, H12a), 2.67 (1H, tt, J1 = 10.5 Hz, J2 = 4.1 Hz, H8), 2.02 (1H, t, J = 6.2 Hz, H1) 1.84 – 1.77 (2H, m, H9, H10), 1.73 (2H, oct, J = 6.2 Hz, H2, H3), 1.42 – 1.29 (2H, m, H9 , H10) 0.91 (6H, d, J = 6.7 Hz, H4, H5), 0.88 (6H, d, J = 6.7 Hz, H6, H7). ^{13}C NMR (100.6 MHz, CDCl$_3$) 67.1 (C11, C12), 65.2 (C1), 54.3 (C8), 34.8 (C9, C10), 30.8 (C2, C3), 21.1 (C4, C5), 18.3 (C6, C7). HRMS (FTMS +p NSI) m/z: [M + H]$^+$ calculated for $C_{12}H_{26}NO$ 201.2043, found = 201.2042, Δ = - 0.5 ppm.

Benzyl 4-((2,4-dimethylpentan-3-yl)amino)piperidine-1-carboxylate (1e)

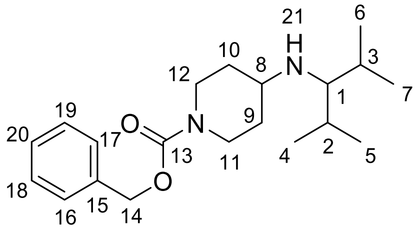

Mr = 332.48

General procedure B was applied to benzyl 4-aminopiperidine-1-carboxylate (1.0 g, 10 mmol), 2,4-dimethylpentanone (1.1 g, 10 mmol) and Ti(*i*-PrO)$_4$ (5.7 g, 20 mmol) neat. After stirring for 8 hours, the mixture was cooled to 0 ^{o}C. Absolute ethanol (40 mL) was added, followed by sodium borohydride (1.1 g, 30 mmol) portion wise and the resultant mixture was stirred for another 8 hours. Purification by silica column (20% to 40% ethyl acetate / petroleum ether) gave **1e** as a colourless liquid (1.1 g, 33%). IR (λ_{max}/cm^{-1}) 2954, 2870, 1694, 1429, 1225, 1116. ^{1}H NMR (400.1 MHz, CDCl$_3$) 7.37 – 7.28 (5H, m, H16, H17, H18, H19, H20), 5.12 (2H, s, H14), 4.09 (2H, br, H11, H12), 2.85 (2H, br, H11, H12), 2.61 (1H, tt, J1 = 10.1 Hz, J2 = 3.8 Hz, H8), 2.00 (1H, t, J = 5.5 Hz, H1), 1.89 – 1.79 (2H, m, H9, H10), 1.73 (2H, oct, J = 7.7 Hz, H2, H3), 1.31-1.16 (2H, m, H9, H10), 0.90 (6H, d, J = 6.7 Hz, H4, H5), 0.87 (6H, d, J = 6.7 Hz, H6, H7). ^{13}C NMR (100.6 MHz, CDCl$_3$) 155.3 (C13), 137.0 (C15), 128.5 (C16, C17), 127.9 (C20), 127.8 (C18, C19), 67.0 (C14), 65.4 (C1), 54.9 (C8), 43.0 (C11, C12), 33.4 (C9, C10), 30.9 (C2, C3), 21.1 (C4, C5), 18.3 (C6, C7). HRMS (FTMS +p NSI) m/z: [M + H]$^+$ calculated for $C_{20}H_{33}N_2O_2$ 334.25705, found = 334.25687, Δ = - 0.5 ppm.

2,4-Dimethylpentan-3-amine hydrochloride (1f-I)

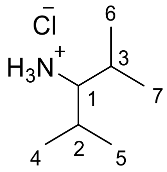

Mr = 151.67

General procedure B was applied to 2,4-dimethylpentanone (2.2 g, 20 mmol), ammonia (7.1 mL, 7 M in methanol), and Ti(*i*-PrO)$_4$ (12 mL, 40 mmol). After stirring for 8 hours, the mixture was cooled to 0 °C. Absolute ethanol (40 mL) was added, followed by sodium borohydride (1.1 g, 30 mmol) portion wise and the resultant mixture was stirred for another 8 hours. The crude was quenched with 2.5 M sodium hydroxide and the resulting white solid was filtered off and washed with diethylether. The aqueous phase was washed with diethylether (3 x 100 mL), and the combined organic layers extracted with 1 M aq. hydrochloric acid (3 x 50 mL), followed by basification with 2.5 M sodium hydroxide (until pH ~ 10). The aqueous phase was extracted with diethylether (3 x 50 mL) and the combined organic layers dried over magnesium sulphate, filtered, treated with 2 M hydrochloric acid in diethylether (20 mL) and concentrated *in vacuo* to afford the title compound **1f-I** as a white solid (2.5 g, 83%). m.p.: 192 – 195°C (lit. 160°C). IR (λ_{max}/cm^{-1}): 3027, 2959, 2889, 1578, 1507, 1463, 1137, 1070, 960. ^{1}H NMR (400.1 MHz, D_2O) 2.85 (1H, t, *J* = 6.1 Hz, H1), 2.08 (2H, m, H2, H3), 1.04 (6H, d, *J* = 6.9 Hz, H4, H5), 0.98 (6H, d, *J* = 6.9 Hz, H6, H7). ^{13}C NMR (100.6 MHz, D_2O) 63.4 (C1), 27.6 (C2, C3), 19.0 (C4, C5), 16.17 (C6, C7). HRMS (ESI) m/z: calculated for $C_7H_{18}NClNa$ $[M+Na]^+$ 174.1017, found 174.1020, Δ = 1.7 ppm.

N-(2,4-dimethylpentan-3-yl)-4,4-difluorocyclohexan-1-amine (1f)

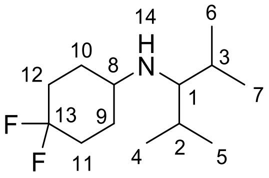

Mr = 233.35

General procedure B was applied to 2,4-dimethylpentylamine (550 mg, 5 mmol), 4,4-difluorocyclohexanone (670 mg, 5 mmol) and Ti(*i*-PrO)$_4$ (2.4 g, 10 mmol) neat. After stirring for 8 hours, the mixture was cooled to 0 °C. Absolute ethanol (20 mL) was added, followed by sodium borohydride (550 mg, 15 mmol) portion wise and the resultant mixture was stirred for another 8 hours. Purification by silica column (5% to 20% ethyl acetate / petroleum ether) gave the **1f** as a colourless liquid (500 mg, 43%). IR (λ_{max}/cm^{-1}) 2957, 2871, 1470, 1448, 1376, 1107. ^{1}H NMR (400.1 MHz, $CDCl_3$) 2.66 – 2.58 (1H, m, H8), 2.16 – 2.03 (2H, m, H11a, H12a) 1.98 (1H, t, J = 5.5 Hz, H1), 1.90 – 1.82 (2H, m, H9, H10), 1.79 – 1.67 (4H, m, H2, H3, H11b, H12b), 1.50 – 1.38 (2H, m, H9b, H10b), 0.91 (6H, d, J = 6.7 Hz, H4, H5), 0.87 (6H, d, J = 6.7 Hz, H6, H7). ^{13}C NMR (100.6 MHz, $CDCl_3$) 123.6 (C13), 65.8 (C1), 54.0 (C8), 31.9 (C11, C12), 30.9 (C2, C3), 29.7 (C9, C10), 21.1 (C4, C5), 18.3 (C6, C7) ^{19}F NMR (MHz, $CDCl_3$) -96.5 (d, 240 Hz), -99.9 (d, 240 Hz) HRMS (FTMS +p NSI) m/z: $[M + H]^+$ calculated for $C_{13}H_{26}F_2N$ 234.2028, found = 234.2029, Δ = 0.4 ppm.

N-(2,4-dimethylpentan-3-yl)-1,4-dioxaspiro[4.5]decan-8-amine (1g)

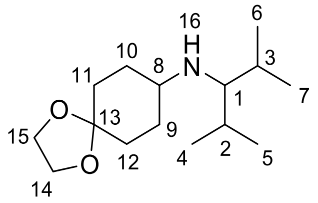

Mr = 225.40

General procedure B was applied to 2,4-dimethylpentylamine (550 mg, 5 mmol), 2,4-1,4-dioxaspiro[4.5]decan-8-one (780 mg, 5 mmol) and Ti(*i*-PrO)$_4$ (2.4 g, 10 mmol) neat. After stirring for 8 hours, the mixture was cooled to 0 ^{o}C. Absolute ethanol (20 mL) was added, followed by sodium borohydride (550 mg, 15 mmol) portion wise and the resultant mixture was stirred for another 8 hours. Purification by silica column (10% to 30% ethyl acetate / petroleum ether) gave **1g** as a colourless liquid (710 mg, 63%). IR (λ_{max}/cm^{-1}) 2950, 2871, 1473, 1378, 1245, 1195, 1152, 1039. ^{1}H NMR (400.1 MHz, $CDCl_3$) 3.92 (4H, s, H14, H15), 2.50 (1H, tt, J1 = 9.9 Hz, J2 = 3.6 Hz, H8), 1.92 (1H, t, J = 5.9 Hz, H1), 1.88 – 1.64 (6H, m, H9a - 12a, H2, H3), 1.51 (2H, td, J1 = 13.2 Hz, H2 = 4.0 Hz, H11b, H12b), 1.42 – 1.30 (2H, m, H9b, H10b), 0.89 (6H, d, J = 7.0 Hz, H4, H5), 0.86 (6H, d, J = 7.0 Hz, H6, H7). ^{13}C NMR (100.6 MHz, $CDCl_3$) 108.9 (C14, C15), 65.7 (C1), 64.3 (C8), 55.2 (C13), 33.2 (C9, C10), 31.2 (C11, C12), 30.9 (C2, C3), 21.0 (C4, C5), 18.3 (C6, C7) HRMS (FTMS +p NSI) m/z: [M + H]$^+$ calculated for $C_{15}H_{30}NO_2$ 256.2271, found = 256.2271, exact match.

N-(2,4-dimethylpentan-3-yl)-2,2-dimethyl-1,3-dioxan-5-amine (1h)

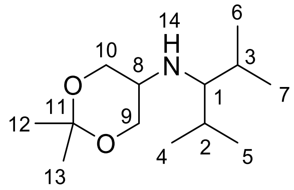

Mr = 229.36

General procedure B was applied to 2,4-dimethylpentylamine (550 mg, 5 mmol), 2,4-2,2-dimethyl-1,3-dioxan-5-one (650 mg, 5 mmol) and Ti(*i*-PrO)$_4$ (2.4 g, 10 mmol) neat. After stirring for 8 hours, the mixture was cooled to 0 ^{o}C. Absolute ethanol (20 mL) was added, followed by sodium borohydride (550 mg, 15 mmol) portion wise and the resultant mixture was stirred for another 8 hours. Purification by silica column (10% to 30% ethyl acetate / petroleum ether) gave **1h** as a colourless liquid (430 mg, 37%). IR (λ_{max}/cm^{-1}) 2957, 2870, 1471, 1379, 1369, 1245, 1197, 1155, 1039. ^{1}H NMR (400.1 MHz, $CDCl_3$) 3.93 (2H, dd, J1 = 11.3 Hz, J2 = 4.1 Hz, H9a, H10a), 3.58 (2H, dd, J1 = 11.8 Hz, J2 = 7.0 Hz, H9b, H10b), 2.67 (1H, sep, J = 3.7 Hz, H8) 1.95 (1H, t, J = 6.0 Hz, H1), 1.71 (2H, oct, J = 6.2 Hz, H2, H3), 1.42 (3H, s, H12), 1.41 (3H, s, H13), 0.90 (6H, d, J = 6.7 Hz, H4, H5), 0.88 (6H, d, J = 6.7 Hz, H6, H7) ^{13}C NMR (100.6 MHz, $CDCl_3$) 97.8 (C11), 66.6 (C1) 64.9 (C9, C10), 51.7 (C8), 30.9 (C2, C3), 25.3 (C13), 22.4 (C12), 21.0 (C4, C5), 18.1 (C6, C7) HRMS (FTMS +p NSI) m/z: [M + H]$^+$ calculated for $C_{13}H_{28}NO_2$ 230.2115, found = 230.2116, Δ = 0.4 ppm.

***tert-butyl 3-((2,4-dimethylpentan-3-yl)amino)azetidine-1-carboxylate* (1i)**

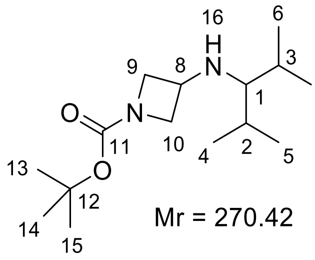

General procedure B was applied to a suspension of 2,4-dimethylpentan-3-amine hydrochloride (1.0 g, 6.6 mmol), tert-butyl 3-oxoazetidine-1-carboxylate (1.7 g, 9.9 mmol), Ti(O*i*Pr)$_4$ (50 mL, 165 mmol), and sodium hydrogen carbonate (550 mg, 6.6 mmol) in tetrahydrofuran (20 mL). Absolute ethanol (40 mL) was added, followed by sodium borohydride (0.5 g, 13.2 mmol) portion wise and the resultant mixture was stirred for another 8 hours. Purification by silica column (0% to 40% ethyl acetate / petroleum ether) gave **1i** as a light yellow oil (1.1 g, 62%). IR (λ_{max}/cm^{-1}): 2955, 2875, 1691, 1476. ^{1}H NMR (400.1 MHz, CDCl$_3$) 4.08 – 4.02 (2H, m, H9a, H10a), 3.67 – 3.54 (3H, m, H8, H9b, H10b), 1.89 (1H, t, J = 5.7 Hz, H1), 1.71 (2H, oct, J = 6.7 Hz, H2, H3), 1.43 (9H, s, H13 – H15), 0.90 (6H, d, J = 6.8 Hz, H4, H5), 0.87 (6H, d, J = 6.8 Hz, H6, H7). ^{13}C NMR (100.6 MHz, CDCl$_3$) 156.6 (C11), 79.2 (C12), 68.1 (C1), 58.0 (C9, C10), 48.9 (C8), 30.3 (C2, C3), 28.4 (C13 – C15), 20.9 (C4, C5), 18.3 (C6, C7). HRMS (ESI) m/z: calculated for $C_{15}H_{31}O_2N_2$ [M+H]$^+$ 271.2380, found 271.2378, Δ = - 0.7 ppm.

2-((2,4-Dimethylpentan-3-yl)amino)ethan-1-ol (1j-I)

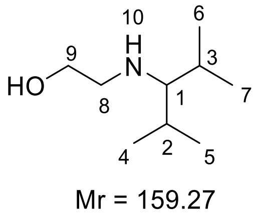

General procedure B was applied to a 2,4-dimethylpentanone (3.9 g, 35 mmol), ethanolamine (2.0 mL, 33 mmol), and Ti(O*i*Pr)$_4$ (50 mL, 165 mmol) Absolute ethanol (150 mL) was added, followed by sodium borohydride (5.5 g, 55 mmol) portion wise and the resultant mixture was stirred for another 8 hours. Purification by silica column (10% methanol / dichloromethane) gave **1j-I** as a colourless oil (0.85 g, 16%). IR (λ_{max}/cm^{-1}) 3326, 2956, 2871, 1469, 1384, 1058, 1025. ^{1}H NMR (400.1 MHz, CDCl$_3$) 3.54 (2H, t, *J* = 5.2 Hz, H9), 2.81 (2H, t, *J* = 5.2 Hz, H8), 1.88 (1H, t, *J* = 5.5 Hz, H1), 1.83 – 1.63 (2H, m, H2, H3), 0.90 (6H, d, *J* = 6.8 Hz, H4, H5), 0.88 (6H, d, *J* = 6.8 Hz, H6, H7). ^{13}C NMR (100.6 MHz, CDCl$_3$) 69.2 (C1), 61.7 (C9), 52.8 (C8), 30.9 (C2, C3), 21.0 (C4, C5), 18.3 (C6, C7). HRMS (ESI) m/z: calculated for $C_9H_{22}NO$ [M+H]$^+$ 160.1696, found 160.1692, Δ = - 2.5 ppm.

The hydrochloride salt was obtained as an off white solid by treatment of the amine with 4M HCl in dioxane, followed by removal of the solvent *in vacuo*. m.p. 82–86 ^{o}C. IR (λ_{max}/cm^{-1}): 3663, 3274, 2974, 1584, 1407, 1394, 1378, 1074. ^{1}H NMR (400.1 MHz, D$_2$O) 3.95 – 3.85 (2H, m, H9), 3.36 – 3.31 (2H, m, H8), 2.94 (1H, t, *J* = 5.8 Hz, H1), 2.39 – 2.12 (2H, m, H2, H3), 1.07 (6H, d, *J* = 6.9 Hz, H4, H5), 1.06 (3H, d, *J* = 6.9 Hz, H6, H7). ^{13}C NMR (100.6 MHz, D$_2$O) 70.8 (C1), 56.6 (C9), 50.7 (C8), 27.7 (C2, C3),

18.9 (C4, C5), 17.1 (C6, C7). HRMS (ESI) m/z: calculated for $C_9H_{22}ON$ $[M+H]^+$ 160.1696, found 160.1692, Δ = - 2.5 ppm.

2,4-dimethyl-*N*-(2-((triisopropylsilyl)oxy)ethyl)pentan-3-amine (1j)

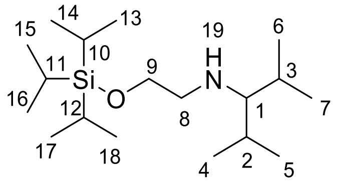

Mr = 315.62

To an ice-cold solution of **3i-I** (0.25 g, 4.8 mmol) and triethylamine (1.0 mL, 14.5 mmol) in dry dichloromethane (10 mL), was added dropwise TIPS-OTf (2.0 mL, 7.3 mmol) and the resulting mixture was stirred for 16 h under an nitrogen atmosphere at 21 °C. The reaction was quenched with saturated aqueous sodium hydrogen carbonate (20 mL) and the aqueous phase extracted with diethylether (3 x 25 mL). The combined organic layers were washed with brine (75 mL), dried over magnesium sulphate, filtered and concentrated *in vacuo*. Purification by silica column (0% - 3% ethyl acetate / petroleum ether) gave **1j** as a colourless oil (490 mg, 95%). IR (λ_{max}/cm^{-1}) 3675, 2943, 2866, 1463, 1088, 1065, 881. ^1H NMR (400.1 MHz, $CDCl_3$) 3.77 (2H, t, *J* = 5.4 Hz, H9), 2.76 (2H, t, *J* = 5.4 Hz, H8), 1.84 (1H, t, *J* = 5.7 Hz, H1), 1.80 – 1.69 (2H, m, H2, H3), 1.29 (1H, br s, H19), 1.15 – 1.01 (21H, m, H10 – H18), 0.90 (12H, t, *J* = 6.9 Hz, H4 – H7). ^{13}C NMR (100.6 MHz, $CDCl_3$) 69.5 (C1), 63.5 (C9), 54.3 (C8), 31.0 (C2, C3), 21.0 (C4, C5), 18.3 (C6, C7), 18.2 (C13 – C18), 12.1 (C10 – C12). HRMS (ESI) m/z: calculated for $C_{18}H_{42}NOSi$ $[M+H]^+$ 316.3030, found 316.3029, Δ = 0.3 ppm.

2,4-Dimethyl-*N*-(2-(pyridin-2-yloxy)ethyl)pentan-3-amine (1k)

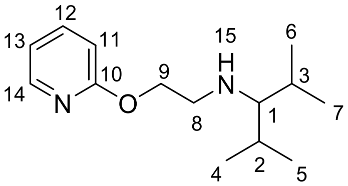

Mr = 236.36

To a suspension of **3i-I**·HCl (460 mg, 3.1 mmol) in dioxane (10 mL) was added sodium hydride (200 mg, 6.4 mmol, 60% in mineral oil) portion wise. The mixture was refluxed for 30 min, then cooled to 21 °C. A solution of 2-chloropyridine (270 mg, 3.1 mmol) in dioxane (10 mL) was added dropwise, then refluxed for an additional 16 hours. The crude was cooled, concentrated *in vacuo*, re-dissolved in ethyl acetate (100 mL), washed with water (100 mL) and brine (100 mL), dried over magnesium sulphate, filtered and concentrated *in vacuo*. Purification by silica column (0% to 5% methanol / dichloromethane) gave **1k** as a light yellow oil (495 mg, 89%). IR (λ_{max}/cm^{-1}) 2955, 1596, 1474, 1431, 1285, 1270, 777. ^1H NMR (400.1 MHz, $CDCl_3$) 8.16 (1H, dd, *J* = 5.0, 1.4 Hz, H14), 7.67 – 7.48 (1H, m, H12), 6.96 – 6.82 (1H, m, H13), 6.76 (1H, d, *J* = 8.4 Hz, H11), 4.42 – 3.96 (2H, m, H9), 3.12 – 3.01 (2H, t, *J* = 5.4 Hz, H8), 1.94 (1H, m, H1), 1.86 – 1.67 (2H, m, H2, H3), 1.31 (1H, br s, H15), 0.9 – 0.6 (12H, m, H4 – H7). ^{13}C NMR (100.6 MHz, $CDCl_3$) 163.9 (C10), 146.9 (C14), 138.4 (C12), 116.6 (C13), 111.1 (C11), 69.4 (C9), 66.1 (C8), 50.7 (C1), 30.9 (C2, C3), 20.8 (C4, C5), 18.1 (C6,

C7). HRMS (ESI) m/z: calculated for $C_{14}H_{25}ON_2$ $[M+H]^+$ 237.1961, found 237.1963, Δ = 0.8 ppm.

2,4-dimethyl-*N*-(2-((6-(trifluoromethyl)pyridin-2-yl)oxy)ethyl)pentan-3-amine (1l)

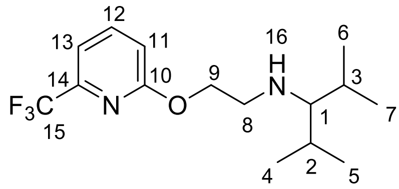

Mr = 304.36

To a suspension of **3i-i**·HCl (230 mg, 3.1 mmol) in dioxane (5 mL) was added sodium hydride (200 mg, 6.4 mmol, 60% in mineral oil) portion wise. The mixture was refluxed for 30 min, then cooled to 21 °C. A solution of 2-chloro-6-(trifluoromethyl) pyridine (500 mg, 2.8 mmol) in dioxane (10 mL) was added dropwise, then refluxed for an additional 16 hours. The crude was cooled, concentrated *in vacuo*, re-dissolved in ethyl acetate (100 mL), washed with water (100 mL) and brine (100 mL), dried over magnesium sulphate, filtered and concentrated *in vacuo*. Purification by silica column (0% - 10% ethyl acetate / petroleum ether) gave **1l** as a light yellow oil (701 mg, 84%). IR (λ_{max}/cm^{-1}) = 2959, 2873, 1284, 1184, 1139, 1123, 809. ^{1}H NMR (400.1 MHz, $CDCl_3$) 7.66 (1H, t, *J* = 7.8 Hz, H12), 7.21 (1H, d, *J* = 7.3 Hz, H11), 6.89 (1H, d, *J* = 8.4 Hz, H13), 4.42 (2H, t, *J* = 5.5 Hz, H9), 3.04 (2H, t, *J* = 5.5 Hz, H8), 1.91 (1H, t, *J* = 5.8 Hz, H1), 1.75 (2H, m, H2, H3), 1.48 (1H, br s, H16), 0.90 (12H, m, H4 – H7). ^{13}C NMR (100.6 MHz, $CDCl_3$) 163.8 (C10), 145.5 (q, *J* = 34.7 Hz, C14), 139.2 (C12), 121.3 (q, *J* = 273.6 Hz, C15), 114.6 (C11), 113.1 (q, *J* = 3.1 Hz, C7), 69.3 (C9), 66.7 (C1), 50.3 (C8), 30.8 (C2, C3), 20.8 (C4, C5), 18.0 (C6, C7). ^{19}F NMR (376 MHz, $CDCl_3$) δ -68.54. HRMS (ESI) m/z: calculated for $C_{15}H_{24}ON_2F_3$ $[M+H]^+$ 305.1835, found 305.1833, Δ = -0.7 ppm.

1-cyclohexyl-2-methylpropan-1-ol (1m-I)

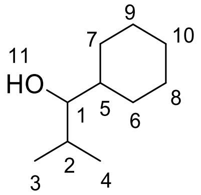

Mr = 156.27

A solution of isobutyraldehyde (3.8 mL, 42 mmol) in tetrahydrofuran (50 mL) was cooled to 0 °C. Cyclohexylmagnesium bromide (30 mL, 60 mmol, 2M in THF) was added portion wise. The mixture was stirred for an additional 2 hours, warming up to room temperature. The reaction mixture was quenched with saturated aqueous ammonium chloride (100 mL) and extracted with ethyl acetate (3 x 50 mL). The combined organic layers were washed with brine (100 mL), dried over magnesium sulphate, filtered, and concentrated *in vacuo*. Purification by silica column (0% to 20% ethyl acetate / petroleum ether) gave the **1m-I** as a colourless liquid (2.1 g, 32%). IR (λ_{max}/cm^{-1}) 3376, 2922, 2851, 1448, 989, 976. ^{1}H NMR (400.1 MHz, $CDCl_3$) 3.03 (1H, t, *J* = 5.7 Hz, H4), 1.91 – 1.82 (1H, m, H5), 1.80 – 1.70 (3H, m, H6a, H7a, H10a), 1.69 – 1.61 (1H, m, H8a), 1.61 – 1.54 (1H, m, H9a), 1.46 – 1.33 (1H, m, H2), 1.30 – 0.97 6H, (m, H6b – H10b, H11), 0.91 (3H, d, *J* = 6.8 Hz, H3), 0.89 (3H, d, *J* = 6.7 Hz, H4). ^{13}C NMR (100.6 MHz, $CDCl_3$) 81.3 (C1), 40.8 (C2), 30.1 (C6), 30.0 (C7), 27.9 (C5),

26.8 (C8), 26.7 (C9), 26.4 (C10), 20.1 (C3), 16.8 (C4). HRMS (ESI) m/z: calculated for $C_{10}H_{24}ON$ $[M+NH_4]^+$ 174.1852, found 174.1853, Δ = 0.6 ppm.

1-cyclohexyl-2-methylpropan-1-one (1m-II)

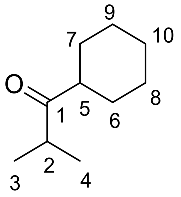

Mr = 154.25

To an ice-cold solution of **1m-I** (1.8 g, 11.5 mmol) in dry dichloromethane (75 mL) was added celite (5g), followed by pyridinium chlorochromate (3.1g, 14.3 mmol). The reaction mixture was warmed to room temperature and stirred for 24 hours under nitrogen. The crude reaction was then filtered through a pad of celite / silica, and the filtrate concentrated *in vacuo* to afford the crude **1m-II** as a colourless oil (1.9 g, >95%). IR (λ_{max}/cm^{-1}) 2929, 2855, 1706, 1449, 996. ^1H NMR (400.1 MHz, $CDCl_3$) 2.74 (1H, hept, *J* = 6.9 Hz, H2), 2.55 – 2.42 (1H, m, H5), 1.84 – 1.61 (5H, m, H6a – H10a), 1.42 – 1.13 (5H, m, H6b – H10b), 1.06 (6H, d, *J* = 6.9 Hz, H3, H4). ^{13}C NMR (100.6 MHz, $CDCl_3$) 217.9 (C1), 49.2 (C5), 39.1 (C2), 28.8 (C6, C7), 26.0 (C10), 25.9 (C8, C9), 18.6 (C3, C4).

1-cyclohexyl-*N*-(cyclohexylmethyl)-2-methylpropan-1-amine (1m)

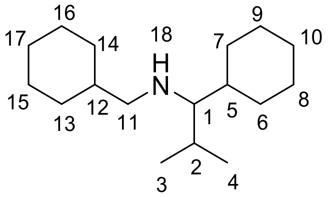

Mr = 251.46

General procedure B was applied to cyclohexanemethylamine (210 µL, 1.6 mmol), **1m-II** (249 mg, 1.6 mmol), and Ti(O*i*Pr)$_4$ (5.0 mL, 16.6 mmol). After stirring for 8 hours, the mixture was cooled to 0 °C. Absolute ethanol (7 mL) was added, followed by sodium borohydride (0.136 g, 3.6 mmol) portion wise and the resultant mixture was stirred for another 8 hours. Purification by silica column (0% to 10% ethyl acetate / petroleum ether) gave **1m** as a colourless liquid (265 mg, 65%). IR (λ_{max}/cm^{-1}) 2919, 2850, 1448, 1129, 1112, 892. ^1H NMR (400.1 MHz, $CDCl_3$) 2.50 – 2.35 (2H, m, H11), 1.85 – 1.55 (12H, m, H1, H2, H6a – H10a, H13a – H17a), 1.44 – 1.30 (2H, m, H5, H12), 1.30 – 0.87 (10H, m, H6b – H10b, H13b – H17b), 0.89 (3H, d, *J* = 6.5 Hz, H3), 0.85 (3H, d, *J* = 6.5 Hz, H4), 0.62 (1H, br s, H18). ^{13}C NMR (100.6 MHz, $CDCl_3$) 68.9 (C1), 59.5 (C11), 41.7 (C5), 39.2 (C12), 30.3 (C2), 31.9, 31.8, 31.4, 29.1, 27.0, 26.9, 26.8, 26.3, 26.3 (C6 – C10, C13 – C17), 21.0 (C3), 18.0 (C4). HRMS (ESI) m/z: calculated for $C_{17}H_{34}N$ $[M+H]^+$ 252.2686, found 252.2684, Δ = - 0.8 ppm.

2-methyl-1-(tetrahydro-2H-pyran-4-yl)propan-1-ol (1n-I)

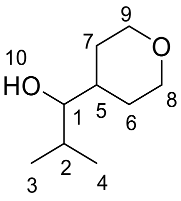

Mr = 156.27

A solution of tetrahydro-2H-pyran-4-carbaldehyde (1.7 g , 15 mmol) in THF (50 mL) was cooled to 0 °C. Isopropylmagnesium bromide (20 mL, 20 mmol, 1M in THF) was added portion wise. The mixture was stirred for an additional 2 hours, warming up to room temperature. The reaction mixture was quenched with saturated aqueous ammonium chloride (100 mL) and extracted with ethyl acetate (3 x 50 mL). The combined organic layers were washed with brine (100 mL), dried over $MgSO_4$, filtered, and concentrated *in vacuo*. Purification by silica column (0% to 50% ethyl acetate / petroleum ether) gave the **1n-I** as a colourless liquid (0.4 g, 17%). IR (λ_{max}/cm^{-1}): 3422, 2957, 2912, 2847.0, 1092, 1004, 987. ^{1}H NMR (400.1 MHz, $CDCl_3$) δ 4.12 – 3.89 (2H, m, H8a, H9a), 3.45 – 3.31 (2H, m, H8b, H9b), 3.16 – 3.02 (1H, m, H1), 1.84 – 1.73 (2H, m, H6a, H7a), 1.71 – 1.59 (1H, m, H2), 1.49 – 1.35 (3H, m, H5, H6b, H7b), 1.33 (1H, d, *J* = 5.6 Hz, H10), 0.96 (3H, d, *J* = 6.8 Hz, H3), 0.89 (3H, d, *J* = 6.7 Hz, H4). ^{13}C NMR (100.6 MHz, $CDCl_3$) δ 80.3 (C1), 68.2 (C8), 68.0 (C9), 38.3 (C5), 29.6 (C2), 29.4 (C7, C8), 28.7 (C6, C7), 20.1 (C3), 15.9 (C4). HRMS (ESI) m/z: calculated for $C_9H_{19}O_2$ $[M+H]^+$ 159.1380, found 159.1383, Δ = 1.9 ppm.

N-(Cyclohexylmethyl)-2-methyl-1-(tetrahydro-2H-pyran-4-yl)propan-1-amine (1n)

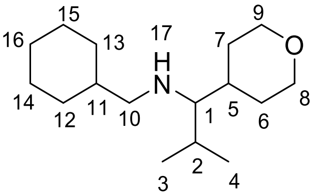

Mr = 253.43

To an ice-cold solution of **1n-I** (300 mg, 1.9 mmol), in dry CH_2Cl_2 (15 mL) was added celite (1.0 g) followed by pyridinium chlorochromate (610 mg, 2.85 mmol). The reaction mixture was allowed to warm to 21 °C and stirred for 24 h under an nitrogen atmosphere. Then, the crude reaction was filtered through a pad of celite / silica and the filtrate concentrated *in vacuo* to afford the crude 2-methyl-1-(tetrahydro-2H-pyran-4-yl)propan-1-one as a brown oil (250 mg, 84%), which was used for next step without further purification.

General procedure A was applied to cyclohexanemethylamine (170 µL, 1.6 mmol), 2-methyl-1-(tetrahydro-2H-pyran-4-yl)propan-1-one (200 mg, 1.3 mmol), and sodium triacetoxyborohydride (325 mg, 1.6 mmol). Purification by silica column (0% to 15% methanol / dichloromethane) gave the **1n** as a colourless liquid (255 mg, 78%). IR (λ_{max}/cm^{-1}) 2920, 2848, 1448, 1123, 1095. ^{1}H NMR (400.1 MHz, $CDCl_3$) 4.21 – 3.74 (2H, m, H8a, H9a), 3.47 – 3.14 (2H, m, H8b, H9b), 2.53 – 2.35 (2H, m, H10), 1.87 (1H, dd, *J* = 6.4, 5.0 Hz, H1), 1.84 – 1.61 (7H, m, H2, H10, H13a – H16a), 1.60 – 1.50 (1H, m, H5), 1.49 – 1.31 (4H, m, H6, H7), 1.30 – 1.09 (3H, m, H12b, H13b, H16b), 0.93 (3H, d, *J* = 6.7 Hz, H3), 0.98 – 0.88 (2H, m, H14b, H15b), 0.86 (3H, d, *J* = 6.7 Hz, H4). ^{13}C NMR (100.6 MHz, $CDCl_3$) 68.6 (C8), 68.4 (C1), 68.3 (C9), 59.4 (C10), 39.4 (C5),

39.3 (C11), 31.8 (C12), 31.7 (C13), 31.1 (C6), 29.8 (C2), 29.7 (C7), 26.9 (C14, C15), 26.3 (C16), 21.0 (C3), 17.3 (C4). HRMS (ESI) m/z: calculated for $C_{16}H_{32}ON$ $[M+H]^+$ 254.2478, found 254.2477, Δ = -0.4 ppm.

(*E*)-*N*-(Cyclopentylmethylene)-2-methylpropane-2-sulfinamide (1o-I)

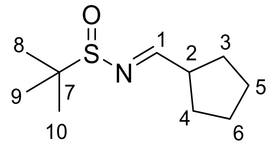

Mr = 201.33

A solution of cyclopentanecarbaldehyde (3.1 g, 32 mmol) in dichloromethane (100 mL) was treated with 2-methylpropane-2-sulfinamide (3.5 g, 29 mmol), copper sulphate (5.0 g, 32 mmol) and magnesium sulphate (7.6 g, 63 mmol). The mixture was stirred under an nitrogen atmosphere for 16 h at 21 °C. Then the crude was filtered through celite and concentrated *in vacuo*. Purification by silica column (0% to 20% ethyl acetate / petroleum ether) gave the **1o-I** as a colourless liquid (2.5 g, 40%). IR (λ_{max}/cm^{-1}) 2956, 2867, 1618, 1081. ^1H NMR (400.1 MHz, $CDCl_3$) 8.01 (1H, d, *J* = 5.6 Hz, H1), 3.02 – 2.91 (1H, m, H2), 1.99 – 1.83 (2H, m, H3a, H4a), 1.78 – 1.60 (6H, m, H3b, H4b, H5, H6), 1.20 (9H, s, H8 – H10). ^{13}C NMR (100.6 MHz, $CDCl_3$) 172.7 (C1), 56.6 (C7), 45.8 (C2), 30.1 (C3), 30.0 (C4), 25.7 (C5), 25.7 (C6), 22.5 (C8 – C10). HRMS (ESI) m/z: calculated for $C_{10}H_{20}ONS$ $[M+H]^+$ 202.1260, found 202.1260, exact match.

***N*-(1-Cyclopentyl-2-methylpropyl)-2-methylpropane-2-sulfinamide (1o-II)**

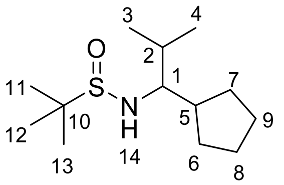

Mr = 245.43

To a stirred solution of **1o-I** (2.2 g, 11 mmol) in dichloromethane (50 mL) at -78 °C was added isopropylmagnesium bromide (17.0 mL, 1 M in tetrahydrofuran) dropwise and the reaction was stirred at the same temperature for 2 hours. The mixture was allowed to warm to room temperature and stirred for an additional 2 hours. Then, the reaction was quenched with saturated aqueous ammonium chloride (100 mL) and diluted with ethyl acetate. The aqueous phase was extracted with ethyl acetate (2 x 50 mL) and the combined organic layers dried over magnesium sulphate, filtered and concentrated *in vacuo*. Purification by silica column (10% to 50% ethyl acetate / petroleum ether) gave **1o-II** as a white solid (1.19 g, 44%). m.p.: 58–61 °C. ^1H NMR (400.1 MHz, $CDCl_3$) 3.12 (1H, d, *J* = 8.0 Hz, H14), 2.93 (1H, td, *J1* = 7.9, J2 = 3.8 Hz, H1), 2.05 – 1.86 (2H, m, H2, H5), 1.81 – 1.48 (6H, m, H6a, H7a, H8, H9), 1.36 – 1.16 (2H, m, H6b, H7b), 1.25 (9H, s, H11 – H13), 1.06 (3H, d, *J* = 6.9 Hz, H3), 0.92 (3H, d, *J* = 6.9 Hz, H4). ^{13}C NMR (100.6 MHz, $CDCl_3$) δ 67.0 (C1), 56.5 (C10), 44.4 (C5), 31.8 (C2), 30.6 (C6), 30.1 (C7), 25.4 (C8, C9), 23.1 (C11 – C13), 20.4 (C3), 17.2 (C4). HRMS (ESI) m/z: calculated for $C_{13}H_{28}ONS$ $[M+H]^+$ 246.1886, found 246.1889, Δ = 1.2 ppm.

(*R*)-1-Cyclopentyl-2-methylpropan-1-amine hydrochloride (1o-III)

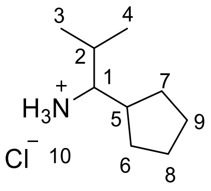

Mr = 177.70

1o-II (1.04 g, 4.3 mmol), hydrochloric acid (7 mL, 6 M in isopropanol), and methanol (7 mL) was stirred at room temperature for 1 hour. The reaction was concentrated *in vacuo* and the white solid was triturated over diethylether, filtered, and washed with diethylether to afford the title compound **1o-III** as a white solid (469 mg, 61%). m.p.: 192–196 °C. IR (λ_{max}/cm^{-1}) 2961, 1578, 1508, 1075. ^{1}H NMR (400.1 MHz, D_2O) 2.91 (1H, dd, *J1* = 9.6 Hz, J2 = 3.2 Hz, H1), 2.12 – 1.92 (2H, m, H2, H5), 1.86 – 1.67 (2H, m, H6a, H7a), 1.67 – 1.37 (4H, m, H8 , H9), 1.23 – 1.06 (2H, m, H6b, H7b), 0.96 (3H, d, *J* = 7.0 Hz, H3), 0.87 (3H, d, *J* = 7.0 Hz, H4). ^{13}C NMR (100.6 MHz, D_2O) 62.3 (C1), 40.7 (C5), 29.3 (C6), 29.2 (C7), 28.7 (C2), 24.6 (C7), 24.3 (C8), 18.9 (C3), 14.5 (C4). HRMS (ESI) m/z: calculated for $C_9H_{20}N$ [M+H]$^+$ 142.1590, found 142.1586, Δ = - 2.8 ppm.

(*R*)-*N*-(Cyclohexylmethyl)-1-cyclopentyl-2-methylpropan-1-amine (1o)

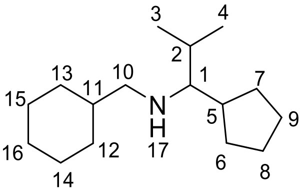

Mr = 237.43

General procedure A was applied to **1o-III** (397 mg, 2.2 mmol), cyclohexanecarboxaldehyde (270 µL, 2.2 mmol), sodium hydrogen carbonate (170 mg, 2.2 mmol), and sodium triacetoxyborohydride (460 mg, 2.6 mmol). Purification by silica column (0% to 5% ethyl acetate / petroleum ether) gave **1o** as a colourless liquid (453 mg, 85%). IR (λ_{max}/cm^{-1}) 2920, 2852, 1448, 1114. ^{1}H NMR (400.1 MHz, $CDCl_3$) 2.55 – 2.39 (2H, m, H10), 2.01 (1H, dd, *J* = 7.7 Hz, J2 = 3.7 Hz, H1), 1.88 – 1.44 (14H, m, H2, H5, H6a, H7a, H8, H9, H10, H12a - H16a), 1.43 – 1.33 (1H, m, H11), 1.20 (5H, dt, *J* = 22.7, 10.1 Hz, H6b, H7b, H12b, H13b, H16b), 0.92 (3H, d, *J* = 6.9 Hz, H3), 0.97 – 0.81 (2H, m, H14b, H15b), 0.86 (3H, d, *J* = 6.9 Hz, H4). ^{13}C NMR (100.6 MHz, $CDCl_3$) 68.3 (C1), 58.6 (C10), 44.5 (C5), 38.9 (C11), 31.7, 31.5, 30.6, 30.10, 26.8, 26.1, 25.3, 25.2 (C2, C6 – C9, C12 – C16), 20.5 (C3), 17.2 (C4). HRMS (ESI) m/z: calculated for $C_{16}H_{32}N$ [M+H]$^+$ 238.2529, found 238.2532, Δ = 1.3 ppm.

Benzyl 4-(1-hydroxy-2-methylpropyl)piperidine-1-carboxylate (1p-I)

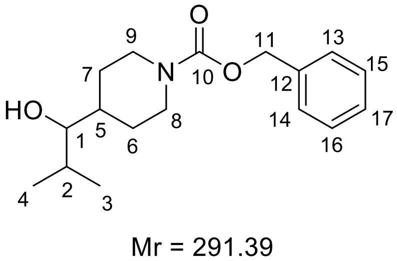

Mr = 291.39

A solution of benzyl 4-formylpiperidine-1-carboxylate (2.47 g, 10 mmol) in dichloromethane (100 mL) was cooled to 0 °C. Isopropylmagnesium chloride (10 mL, 20 mmol, 2M in tetrahydrofuran) was added portionwise. The mixture was stirred for an additional 2 hours, warming up to room temperature. The reaction mixture was quenched with saturated aqueous ammonium chloride (100 mL) and extracted with ethyl acetate (3 x 50 mL). The combined organic layers were washed with brine (100 mL), dried over magnesium sulphate, filtered, and concentrated *in vacuo*. Purification by silica column (0% to 30% ethyl acetate / petroleum ether) gave the **1p-I** as a colourless liquid (2.5 g, 85%). IR (λ_{max}/cm^{-1}) 3464 (br), 2959, 2869, 1679, 1431, 1364, 1278, 1213, 1124, 1070, 976. ^1H NMR (400.1 MHz, $CDCl_3$) 7.39 – 7.27 (5H, m, H13 – H17), 5.12 (2H, s, H11), 4.23 (2H, br s, H8a, H9a), 3.08 (1H, br s, H1), 2.74 (2H, br s, H8b, H9b), 1.90 – 1.82 (1H, m, H7a), 1.77 (1H, oct, J = , 6.3 Hz, H2), 1.63 – 1.48 (2H, m, H5, H6a), 1.47 – 1.38 (1H, br, OH), 1.36 – 1.19 (2H, m, H6b, H7b), 0.94 (3H, d, J = 6.9 Hz, H3,), 0.90 (3H, d, J = 6.9 Hz, H4). ^{13}C NMR (100.6 MHz, $CDCl_3$) 155.3 (C10), 137.0 (C12), 128.5 , 127.9, 127.8 (C13 – C17), 79.9 (C1), 67.0 (C11), 44.1 (C8), 44.0 (C9), 39.0 (C5), 29.7 (C2), 28.7 (C6), 27.5 (C7), 19.9 (C4), 16.1 (C3). HRMS (FTMS +p NSI) m/z: $[M + H]^+$ calculated for $C_{17}H_{26}NO_3$ 292.1907 found = 292.1908, Δ = 0.3 ppm.

Benzyl 4-isobutyrylpiperidine-1-carboxylate (*1p-II*)

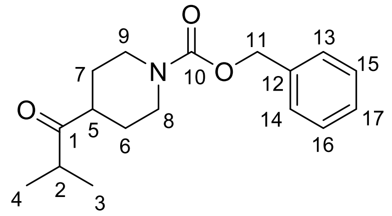

Mr = 289.37

A solution of **1p-I** (1.7 g, 6 mmol) in DCM (60 mL) was cooled to 0 °C. Dess-Martin periodinane (2.8 g, 6.6 mmol) was added portion wise. The mixture was stirred for an additional 2 hours, warming up to room temperature. 2.5 M NaOH (30 mL) was added and the mixture stirred for 30 minutes. The reaction mixture was extracted with dichloromethane (3 X 50 mL), dried over magnesium sulphate, filtered, and concentrated *in vacuo*. Purification by silica column (0% to 20% ethyl acetate / petroleum ether) gave the **1p-II** as a colourless liquid (1.4 g, 82%). IR (λ_{max}/cm^{-1}) 2967, 1693, 1427, 1277, 1221, 1127, 1090, 1013, 994. ^1H NMR (400.1 MHz, $CDCl_3$) 7.37 – 7.28 (5H, m, H13 – H17), 5.12 (2H, s, H11), 4.19 (2H, br s, H8a, H9a), 2.93 – 2.81 (2H, m, H8b, H9b), 2.77 (1H, sep, J = 7.2 Hz, H2), 2.67 (1H, tt, J1 = 11.2 Hz, J2 = 3.6 Hz, H5), 1.75 (2H, br, H6a, H7a), 1.57 (2H, qd, J1 = 12.9 Hz, J2 = 4.8 Hz, H6b, H7b), 1.08 (6H, d, J = 7.0 Hz, H3, H4). ^{13}C NMR (100.6 MHz, $CDCl_3$) 215.8 (C1), 155.2 (C10), 136.8 (C12), 128.5, 128.0, 127.9 (C13 – C17), 67.1 (C11), 46.5 (C5), 43.5 (C8, C9), 38.9 (C2), 27.7 (C6, C7), 18.4 (C3, C4). HRMS (FTMS +p NSI) m/z: $[M + H]^+$ calculated for $C_{17}H_{24}NO_3$ 290.1756 found = 290.1754, Δ = - 0.6 ppm.

Benzyl 4-(2-methyl-1-((tetrahydro-2H-pyran-4-yl)amino)propyl)piperidine-1-carboxylate (1p)

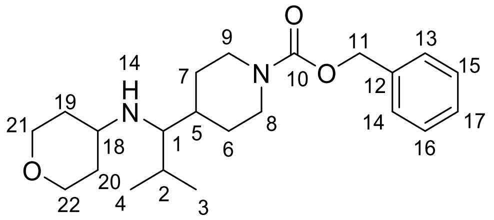

Mr = 374.52

General procedure B was applied to 4-aminotetrahydropyran (300 mg, 3 mmol), benzyl 4-isobutyrylpiperidine-1-carboxylate (800 mg, 2.7 mmol) and Ti(*i*-PrO)$_4$ (1.7 g, 6 mmol) neat. After stirring for 8 hours, the mixture was cooled to 0 °C. Absolute ethanol (15 mL) was added, followed by sodium borohydride (330 mg, 10 mmol) portion wise and the resultant mixture was stirred for another 8 hours. Purification by silica column (50% to 70% ethyl acetate / petroleum ether) gave the **1p** as a colourless liquid (700 mg, 62%). IR (λ_{max}/cm^{-1}) 2953, 2850, 1690, 1469, 1431, 1363, 1278, 1250, 1215, 1121, 1088, 1010. ^{1}H NMR (400.1 MHz, CDCl$_3$) 7.37 – 7.26 (5H, m, H13, H14, H15, H16, H17), 5.11 (2H, s, H11) 4.21 (2H, br, H8, H9), 3.98 – 3.88 (2H, m, H21a, H22a), 3.33 (2H, td, J = 12.0 Hz, J2 = 1.8 Hz, H21b, H22b) 2.80 – 2.63 (2H, br, H8b, H9b), 2.58 (1H, tt, J1 = 10.0 Hz, J2 = 3.6 Hz, H18), 2.07 (1H, t, J = 5.9 Hz, H1), 1.83 – 1.70 (4H, m, H2, H19b, H20b), 1.61 – 1.41 (2H, m, H6a, H7a), 1.39 – 1.13 (5H, m, H5, H6b, H7b, H19b, H20b), 0.92 (3H, d, J = 6.7 Hz, H3), 0.86 (3H, d, J = 6.7 Hz, H4) ^{13}C NMR (100.6 MHz, CDCl$_3$) 155.2 (C10), 137.0 (C12), 128.5, 127.9, 127.8 (C13, C14, C15, C16, C17), 67.0 (C11), 66.9 (C21, C22), 63.7 (C1), 54.4 (C18), 44.5, 44.4 (C8, C9), 40.1 (C5), 34.8, 34.6 (C19, C20), 29.9 (C2), 28.6 (C6, C7), 21.1, 17.6 (C3, C4) HRMS (FTMS +p NSI) m/z: [M + H]$^+$ calculated for $C_{22}H_{34}N_2O_3$ 375.2642, found = 375.2642, exact match.

Benzyl (*S*)-(1-hydroxy-3-methylbutan-2-yl)carbamate (1q-l)

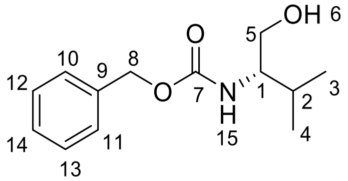

Mr = 237.30

To a solution of L-valinol (7.7 g, 74.6 mmol) in dichloromethane (150 mL) was added triethylamine (23 mL, 157 mmol) followed by dropwise addition of benzyl chloroformate (8.55 mL, 67.1 mmol) at 0 °C under an nitrogen atmosphere. The mixture was allowed to warm to room temperature and stirred for 16 hours. The reaction was diluted with dichloromethane (100 mL), washed with 1 M aqueous hydrochloric acid (2 x 200 mL), brine (200 mL), dried over magnesium sulphate, filtered and concentrated *in vacuo*. Purification by silica column (20% to 50% ethyl acetate / petroleum ether) gave **1q-l** as a white solid (7.1 g, 40%). $[\alpha]_D^{20}$= -25.7° (c 1.1 in CHCl$_3$); m.p.: 55–57 °C (lit.[1] 57–59 °C). IR (λ_{max}/cm^{-1}) 3302, 1682, 1542, 1241, 1046. ^{1}H NMR (400.1 MHz, CDCl$_3$) 7.40 – 7.28 (5H, m, H10 – H14), 5.28 – 4.89 (3H, m, H8, H15), 3.73 – 3.56 (2H, m, H1, H5a), 3.56 – 3.40 (1H, m, H5b), 2.68 (1H, br s, H6), 1.92 – 1.70 (1H, m, H2), 0.94 (3H, d, *J* = 6.8 Hz, H3), 0.91 (3H, d, *J* = 6.8 Hz, H4). ^{13}C NMR (100.6 MHz, CDCl$_3$) 157.2 (C7), 136.5 (C9), 128.6, 128.2, 128.2 (C10 – C13), 67.0 (C8), 63.8 (C5), 58.7 (C1), 29.3 (C2), 19.6 (C3), 18.6 (C4).

Benzyl (*S*)-(3-methyl-1-oxobutan-2-yl)carbamate (1q-II)

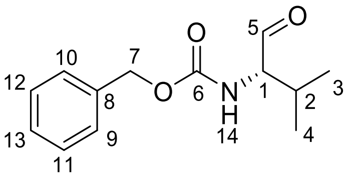

Mr = 235.28

To an ice-cold solution of **1q-I** (1.7 g, 7.2 mmol) in dry dichloromethane (75 mL) was added celite (5g), followed by pyridinium chlorochromate (3.1g, 14.3 mmol). The reaction mixture was warmed to room temperature and stirred for 24 hours under nitrogen. Purification by silica column (0% to 30% ethyl acetate / petroleum ether) gave the **1q-II** as a colourless oil (1.1 g, 67%). $[\alpha]_D^{20}$= +31.8°(c 0.96 in $CHCl_3$). IR (λ_{max}/cm^{-1}) 3322, 2964, 1698, 1514, 1229, 696. ^{1}H NMR (400.1 MHz, $CDCl_3$) 9.61 (1H, s, H5), 7.42 – 7.26 (5H, m, H9 – H13), 5.38 – 5.23 (1H, m, H14), 5.08 (2H, s, H7), 4.29 (1H, dd, *J1* = 7.7 Hz, J2 = 4.2 Hz, H1), 2.37 – 2.20 (1H, m, H2), 1.00 (3H, d, *J* = 7.0 Hz, H3), 0.91 (3H, d, *J* = 7.0 Hz, H4). ^{13}C NMR (100.6 MHz, $CDCl_3$) 199.9 (C5), 156.5 (C6), 136.3 (C8), 128.7, 128.4, 128.3 (C9 – C13), 67.3 (C7), 65.2 (C1), 29.2 (C2), 19.1 (C3), 17.7 (C4).

Benzyl (S)-(1-(1,3-dioxolan-2-yl)-2-methylpropyl)carbamate (1q-III)

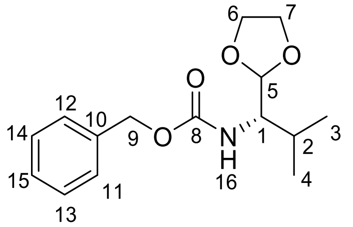

Mr = 279.34

A round-bottomed flask containing a mixture of **1q-II** (1.0 g, 4.3 mmol), *p*-toluenesulfonic acid (10 mg, 0.06 mmol), and ethylene glycol (3 mL, 43.1 mmol) in toluene (50 mL) was coupled to a Dean-Stark and refluxed for 16 h under an nitrogen atmosphere. The reaction was cooled to room temperature and concentrated *in vacuo*. Purification by silica column (10% to 30% ethyl acetate / petroleum ether) gave the **1q-III** as a colourless oil (950 mg, 80%). $[\alpha]_D^{20}$= -32.7° (c. 0.85, $CHCl_3$). IR (λ_{max}/cm^{-1}) 3339, 1683, 1530, 1239, 1024, 700. ^{1}H NMR (400.1 MHz, $CDCl_3$) 7.45 – 7.28 (5H, m, H11 – H15), 5.12 (2H, s, H9), 4.96 (1H, d, *J* = 1.8 Hz, H5), 4.89 (1H, d, *J* = 9.8 Hz, H16), 4.05 – 3.81 (4H, m, H6, H7), 3.77 (1H, ddd, *J1* = 9.8 Hz, J2 = 6.5 Hz, J3 = 1.8 Hz, H1), 1.95 – 1.78 (1H, m, H2), 0.99 (3H, d, *J* = 6.8 Hz, H3), 0.95 (3H, d, *J* = 6.8 Hz, H4). ^{13}C NMR (100.6 MHz, $CDCl_3$) 156.9 (C8), 136.7 (C10), 128.5, 128.1 (C11 – C15), 103.1 (C5), 66.7 (C9), 65.5 (C6), 65.0 (C7), 57.4 (C1), 29.8 (C2), 19.7 (C3), 18.5 (C4). HRMS (ESI) m/z: calculated for $C_{15}H_{22}O_4N$ $[M+H]^+$ 280.1543, found 280.1546. Δ = 1.1 ppm.

(*S*)-1-(1,3-Dioxolan-2-yl)-2-methylpropan-1-amine (1q-IV)

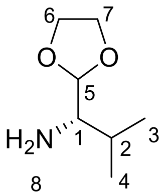

Mr = 145.20

To solution of **1q-III** (750 mg, 2.7 mmol) in methanol (50 mL) was added palladium on carbon (550 mg, 10 wt. %). The mixture was stirred for 16 h under a hydrogen atmosphere. The reaction was filtered through celite and concentrated *in vacuo*. Purification by silica column (20% methanol / dichloromethane) gave the **1q-IV** as a colourless oil (327 mg, 84%). $[\alpha]_D^{20}$ = +3.1° (c. 1.06, $CHCl_3$). IR (λ_{max}/cm^{-1}) 3380, 2959, 2877, 1120, 1025, 947. ^1H NMR (400.1 MHz, $CDCl_3$) 4.81 (1H, d, *J* = 3.5 Hz, H5), 4.03 – 3.95 (2H, m, H6), 3.92 – 3.79 (2H, m, H7), 2.60 (1H, dd, *J1* = 5.1 Hz, J2 = 3.7 Hz, H1), 1.90 – 1.72 (1H, m, H2), 1.38 (2H, br s, H8), 0.98 (6H, t, *J* = 7.1 Hz, H3, H4). ^{13}C NMR (100.6 MHz, $CDCl_3$) 105.0 (C5), 65.3 (C6), 65.1 (C7), 58.3 (C1), 30.1 (C2), 20.0 (C3), 18.0 (C4). HRMS (ESI) m/z: calculated for $C_7H_{16}O_2N$ $[M+H]^+$ 146.1176, found 146.1172, Δ = -2.7 ppm.

(*S*)-*N*-(Cyclohexylmethyl)-1-(1,3-dioxolan-2-yl)-2-methylpropan-1-amine (1q)

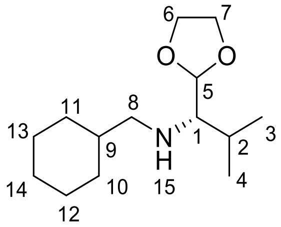

Mr = 241.38

General procedure A was applied to **1q-IV** (200 mg, 1.4 mmol), cyclohexanecarboxaldehyde (172 µL, 4.4 mmol) and sodium triacetoxyborohydride (1.5 g, 7.0 mmol). Purification by silica column (0% to 30% ethyl acetate / petroleum ether) gave the **1q** as a colourless oil (200 mg, 60%). $[\alpha]_D^{20}$= -1.4° (c. 1.0, $CHCl_3$). IR (λ_{max}/cm^{-1}) 2920, 2850, 1464, 1448, 1117, 1028, 944; ^1H NMR (400.1 MHz, $CDCl_3$) 4.83 (1H, d, *J* = 3.8 Hz, H5), 4.04 – 3.90 (2H, m, H6), 3.89 – 3.78 (2H, m, H7), 2.60 (1H, dd, *J1* = 11.3 Hz, J2 = 6.4 Hz, H8a), 2.46 – 2.34 (2H, m, H8b, H1), 1.97 – 1.84 (1H, m, H2), 1.82 – 1.57 (5H, m, H10a – H14a), 1.46 – 1.33 (1H, m, H9), 1.31 – 1.07 (4H, m, H15, H10b, H11b, H14b), 0.98 – 0.92 (6H, m, H3, H4), 0.99 – 0.78 (2H, m, H12, H13). ^{13}C NMR (100.6 MHz, $CDCl_3$) 105.7 (C5), 65.2 (C1), 64.7 (C6), 64.7 (C7), 57.0 (C8), 38.8 (C9), 31.7 (C10), 31.6 (C11), 29.6 (C2), 26.9 (C14), 26.3 (C12, C13), 19.8 (C3), 18.5 (C4). HRMS (ESI) m/z: calculated for $C_{14}H_{28}O_2N$ $[M+H]^+$ 242.2115, found 242.2109, Δ = -2.5 ppm.

((Benzyloxy)carbonyl)-L-valine (1r-I)

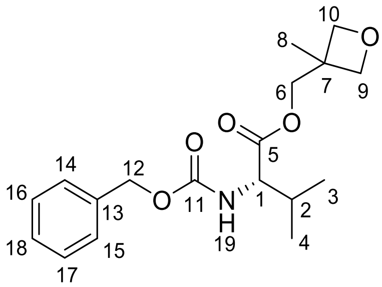

Mr = 335.40

To an ice-cold solution of L-valine (4.1 g, 16.3 mmol) in 1 M aqueous sodium hydroxide (100 mL) was added dropwise a solution of benzyl chloroformate (6.4 mL, 44.8 mmol) in dioxane (40 mL). The mixture was allowed to warm to room temperature and stirred for 16 hours. The crude aqueous mixture was washed with diethylether (2 x 100 mL), neutralized with 3 M aqueous hydrochloric acid until pH ~ 2 and extracted with diethylether (3 x 50 mL). The combined organic layers were dried over magnesium sulphate, filtered and concentrated *in vacuo* to afford crude ((benzyloxy)carbonyl)-L-valine as a thick colourless oil (8.1 g, 95%).

To an ice cold stirred solution of N,N'-dicyclohexylcarbodimide (6.0 g, 29.3 mmol), 3-methyl- 3-oxetanemethanol (3.3 mL, 33.4 mmol), and DMAP (34 mg, 0.3 mmol) in dry dichloromethane (150 mL) was added dropwise a solution of ((benzyloxy)carbonyl)-L-valine (8.0 g, 27.9 mmol) in dichloromethane (50 mL). The reaction mixture was stirred for 5 h and the formed precipitate was filtered off. The filtrate was washed with water (100 mL), 0.01 M aqueous HCl (2 x 100 mL), and brine (100 mL), dried over magnesium sulphate, and concentrated *in vacuo*. Purification by silica column (0% to 50% ethyl acetate / petroleum ether) gave the **1r-I** as a colourless oil (7.6 g, 71%). $[\alpha]_D^{20}$= +4.38° (c. 0.89, $CHCl_3$). IR (λ_{max}/cm^{-1}) 3390, 2964, 2876, 1717, 1525, 979. ^1H NMR (400.1 MHz, $CDCl_3$) 7.55 – 7.14 (5H, m, H14 – H18), 5.48 (1H, d, *J* = 8.9 Hz, H12a), 5.09 (2H, s, H6), 4.5 – 4.42 (2H, m, H9a, H10a), 4.35 (2H, d, *J* = 6.0 Hz, H9b, H10b), 4.31 (1H, dd, *J* = 9.0 Hz, 4.3 Hz, H1), 4.20 (2H, q, *J* = 11.1 Hz), 2.23 – 2.11 (1H, m, H2), 1.30 (3H, s, H8), 0.97 (3H, d, *J* = 6.8 Hz, H3), 0.89 (3H, d, *J* = 6.8 Hz, H4). ^{13}C NMR (100.6 MHz, $CDCl_3$) 172.1, 156.3, 136.3, 128.5, 128.1, 128.1, 79.3, 69.4, 67.0, 59.1, 39.0, 31.1 21.1, 19.0, 17.5.

(S)-2-Methyl-1-(4-methyl-2,6,7-trioxabicyclo[2.2.2]octan-1-yl)propan-1-amine (1r-II)

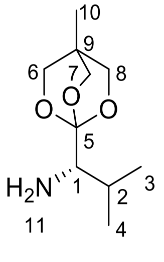

Mr = 201.27

A solution of **1r-I** (7.0 g, 20.9 mmol) in dichloromethane (100 mL) was treated with $BF_3 \cdot Et_2O$ (5 mL, 40.5 mmol) and the mixture was stirred under an N_2 atmosphere for 16 h at room temperature. Triethylamine (8.0 mL, 60.7 mmol) was added to the reaction and the crude mixture concentrated *in vacuo* until dryness. The residue was dissolved in ethyl acetate, washed with diluted aqueous potassium carbonate (2 x 100

mL), brine (200 mL), dried over magnesium sulphate, filtered, and concentrated *in vacuo* to afford crude benzyl (S)-(2-methyl-1-(4-methyl-2,6,7-trioxabicyclo[2.2.2]octan-1-yl)propyl)carbamate as a colourless oil (6.8 g, 95%).

To a solution of benzyl (S)-(2-methyl-1-(4-methyl-2,6,7-trioxabicyclo[2.2.2]octan-1-yl)propyl)carbamate (6.0 g, 17.9 mmol) in dry methanol was added palladium on carbon (5.0 g, 10 wt. %) and the mixture was stirred under a hydrogen atmosphere for 24 hours. Then the crude was filtered through celite and concentrated *in vacuo*. Purification by silica column (0% to 20% ethyl acetate / petroleum ether) gave the **1r-II** as a colourless oil (1.97g, 55%). $[\alpha]_D^{20}$= -12.2° (c. 1.0, $CHCl_3$). IR (λ_{max}/cm^{-1}) 2960, 2876, 1031, 1597, 976; ^{1}H NMR (400.1 MHz, $CDCl_3$) 3.87 (6H, s, H6 – H8), 2.60 (1H, d, *J* = 3.2 Hz, H1), 2.05 (1H, sept d, *J* = 6.9, 3.2 Hz, H2), 1.38 (2H, br s, H11), 0.96 (, 3H, d, *J* = 6.9 HzH3), 0.88 (3H, d, *J* = 6.9 Hz, H4), 0.78 (3H, s, H10). ^{13}C NMR (100.6 MHz, $CDCl_3$) 109.8 (C5), 72.6 (C6 – C8), 60.2 (C1), 30.6 (C9), 27.7 (C2), 21.9 (C3), 16.4 (C4), 14.6 (C10).

(*S*)-*N*-(Cyclohexylmethyl)-2-methyl-1-(4-methyl-2,6,7-trioxabicyclo[2.2.2]octan-1-yl)propan-1-amine (1r)

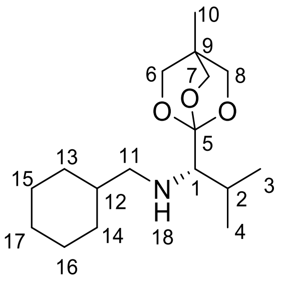

Mr = 297.44

General procedure A was applied to **1r-II** (510 mg, 2.5 mmol), cyclohexanecarboxaldehyde (270 mg, 2.5 mmol) and sodium triacetoxyborohydride (625 mg, 3.0 mmol). Purification by silica column (0% to 10% ethyl acetate / petroleum ether) gave the **1r** as a colourless oil (595 mg, 79%). $[\alpha]_D^{20}$= -8.6° (c. 0.9, $CHCl_3$). IR (λ_{max}/cm^{-1}) 3370; 2923, 1043, 969, 749, 688. ^{1}H NMR (400.1 MHz, $CDCl_3$) 3.86 (s, 6H, H6 – H8), 2.68 (1H, dd, *J1* = 11.3 Hz, J2 = 6.4 Hz, H11a), 2.40 (1H, d, *J* = 3.0 Hz, H1), 2.33 (1H, dd, *J* = 11.3, 6.9 Hz, H11b), 2.13 – 1.96 (1H, m, H2), 1.87 – 1.56 (5H, m, H13a – H17a), 1.45 – 1.31 (1H, m, H12), 1.30 – 1.06 (3H, m, H13b, H14b, H17b), 0.94 (3H, d, *J* = 6.9 Hz, H3), 0.98 – 0.81 (2H, m, H15b, H16b), 0.87 (3H, d, *J* = 6.9 Hz, H4), 0.78 (3H, s, H10). ^{13}C NMR (100.6 MHz, $CDCl_3$) 110.2 (C5), 72.3 (C6 – C8), 66.6 (C1), 57.3 (C11), 38.6 (C12), 31.6 (C13), 31.5 (C14), 30.4 (C9), 28.1 (C2), 26.9 (C15, C16), 26.2 (C17), 21.7 (C3), 17.0 (C4), 14.6 (C10). HRMS (ESI) m/z: calculated for $C_{17}H_{32}O_3N$ $[M+H]^+$ 298.2377, found 298.2379, Δ = 0.7 ppm.

N-(cyclohexylmethyl)-2-methyl-1-(4-methyl-2,6,7-trioxabicyclo[2.2.2]octan-1-yl)butan-1-amine (1s)

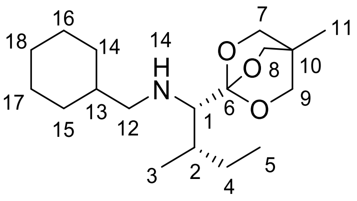

Mr = 311.47

2-methyl-1-(4-methyl-2,6,7-trioxabicyclo[2.2.2]octan-1-yl)butan-1-amine was prepared as per literature procedure.[2] Spectra matches reported ^{1}H NMR (400.1 MHz, $CDCl_3$) 3.90 (6H, s, H7 – H9), 2.66 (1H, d, J = 3.4 Hz, H1), 1.79 – 1.69 (2H, m, H2, H4a), 1.39 (2H, br, NH), 1.09 – 1.00 (1H, m, H4b), 0.97 (3H, d, J = 6.9 Hz, H3), 0.87 (3H, t, J = 7.0 Hz, H5), 0.80 (3H, s, H11). ^{13}C NMR (100.6 MHz, $CDCl_3$) 109.8 (C6), 72.5 (C7 – C9), 60.2 (C1), 34.9 (C2), 30.4 (C10), 23.1 (C4), 17.5 (C3), 14.5 (C11), 12.0 (C5).

General procedure A was applied to 2-methyl-1-(4-methyl-2,6,7-trioxabicyclo[2.2.2]octan-1-yl)butan-1-amine (400 mg, 2 mmol), cyclohexanecarbaldehyde (220 mg, 2 mmol) and sodium triacetoxyborohydride (500 mg, 2.4 mmol) in dichloromethane (40 mL). Purification by silica column (10% to 30% ethyl acetate / petroleum ether) gave the **1s** as a colourless liquid (320 mg, 52%). IR (λ_{max}/cm^{-1}) 2958, 2922, 2873, 2851, 1459, 1394, 1351, 1269, 1193, 1054. ^{1}H NMR (400.1 MHz, $CDCl_3$) 3.86 (6H, s, H7, H8, H9), 2.64 (1H, dd, J1 = 11.0 Hz, J2 = 6.2 Hz, H12a), 2.45 (1H, d, J = 3.0 Hz, H1), 2.34 (1H,dd, J1 = 11.0 Hz, J2 = 6.2 Hz, H12b), 1.83 – 1.59 (7H, m, H2, H4a, H14a, H15a, H16a, H17a, H18a), 1.45 – 1.31 (1H, m, H13), 1.29 – 0.99 (4H, m, H4b, H16b, H17b), 0.94 (3H, d, J = 6.7 Hz, H3), 0.90 – 0.81 (5H, m, H5, H14b, H15b), 0.78 (3H, s, H11). ^{13}C NMR (100.6 MHz, $CDCl_3$) 72.3 (C7, C8, C9), 66.7 (C1), 57.0 (C12), 38.6 (C13), 35.4 (C2), 31.6 (C14), 31.5 (C15), 30.4 (C10), 26.9 (C18), 26.2 (C16, C17), 23.8 (C4), 17.3 (C3), 14.6 (C11), 12.4 (C5). HRMS (FTMS +p NSI) m/z: $[M + H]^+$ calculated for $C_{18}H_{34}NO_3$ 312.2533, found = 312.2532, Δ = -0.3 ppm.

N-(1-cyclohexylpropyl)tetrahydro-2H-pyran-4-amine (1t)

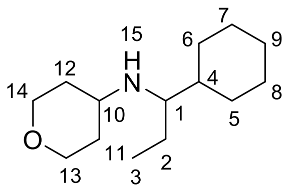

Mr = 225.38

General procedure B was applied to 4-aminotetrahydropyran (1.0 g, 10 mmol), 1-cyclohexylpropan-1-one (1.4 g, 10 mmol) and Ti(*i*-PrO)$_4$ (5.7 g, 20 mmol) neat. After stirring for 8 hours, the mixture was cooled to 0 °C. Absolute ethanol (40 mL) was added, followed by sodium borohydride (1.1 g, 30 mmol) portion wise and the resultant mixture was stirred for another 8 hours. Purification by silica column (30 % to 50% ethyl acetate / petroleum ether) gave the **1t** as a colourless liquid (1.8 g, 79%). IR (λ_{max}/cm^{-1}) 2921, 2848, 1448, 1367, 1140, 1087. ^{1}H NMR (400.1 MHz, $CDCl_3$) 3.95 (2H, dt, J1 = 11.6 Hz, J2 = 3.3 Hz, H13b, H14b), 3.38 (2H, tt, J1 = 11.5 Hz, J2 = 1.8Hz, H13b, H14b), 2.65 (1H, tt, J1 = 10.2 Hz, J2 = 4.2 Hz, H10), 2.27 (1H, m, H1), 1.83 – 1.58 (7H, m, H5a, H6a, H7a, H8a, H9a, H11a, H12a) 1.51 – 1.38 (1H, m, H2), 1.39 –

0.92 (8H, m, H4, H11b, H12b, H(5-9)b), 0.87 (3H, t, J = 7.6Hz, H3). ^{13}C NMR (100.6 MHz, $CDCl_3$) 67.0 (C13, C14), 60.3 (C1), 51.9 (C10), 41.0 (C2), 34.6 (C11), 34.5 (C12), 29.2 (C5), 29.1 (C6), 26.8 (C7), 26.7 (C8), 24.3 (C4, C9), 10.7 (C3). HRMS (FTMS +p NSI) m/z: $[M + H]^+$ calculated for $C_{14}H_{28}NO$ 226.2165, found = 226.2164, Δ = - 0.4 ppm.

N-(2,4-dimethylpentan-3-yl)hexan-1-amine (1u)

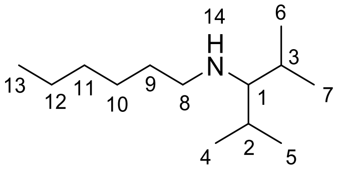

Mr = 199.38

General procedure B was applied to 1-hexylamine (1.0 g, 10 mmol), 2,4-dimethylpentanone (1.1 g, 10 mmol) and Ti(*i*-PrO)$_4$ (5.7 g, 20 mmol) neat. After stirring for 8 hours, the mixture was cooled to 0 ^{o}C. Absolute ethanol (40 mL) was added, followed by sodium borohydride (1.1 g, 30 mmol) portion wise and the resultant mixture was stirred for another 8 hours. Purification by kugelrohr (120 ^{o}C, 5 mbar) gave the **1u** as a colourless liquid (1.2g, 60%). IR (λ_{max}/cm^{-1}) 2956, 2924, 2871, 1466, 1381, 1115. ^1H NMR (400.1 MHz, $CDCl_3$) 2.60 (2H, t, J = 7.2 Hz, H8), 1.82 (1H, t, J = 5.9 Hz, H1), 1.74 (2H, oct, J = 6.7 Hz, H2, H3) 1.49 – 1.39 (2H, m, H9), 1.37 – 1.23 (6H, m, H10, H11. H12), 0.92 – 0.86 (15H, m (overlap), H4, H5, H6, H7, H13) ^{13}C NMR (100.6 MHz, $CDCl_3$) 69.5 (C1), 52.3 (C8), 31.9 (C9), 31.0 (C10), 30.8 (C2, C3), 27.1 (C11), 22.7 (C12), 20.9 (C4, C5), 18.2 (C6, C7), 14.1 (C13). HRMS (FTMS +p NSI) m/z: $[M + H]^+$ calculated for $C_{13}H_{30}N$ 200.2373, found = 200.2372, Δ = - 0.5 ppm.

1-cyclopentylpropan-1-ol (1v-I)

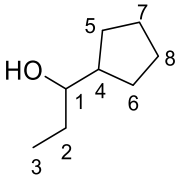

Mr = 128.22

A solution of cyclopentanecarbaldehyde (1.9 g, 20 mmol) in dichloromethane (200 mL) was cooled to 0 ^{o}C. Ethylmagnesium bromide (5 mL, 15 mmol, 3M in ether) was added portion wise. The mixture was stirred for an additional 2 hours, warming up to room temperature. The reaction mixture was quenched with saturated aqueous ammonium chloride (100 mL) and extracted with dichloromethane (3 x 100 mL). The combined organic layers were washed with brine (100 mL), dried over magnesium sulphate, filtered, and concentrated *in vacuo* to give the crude **1v-I** as a colourless liquid (2.1 g, 81%). IR (λ_{max}/cm^{-1}) 3335 (br), 1708, 1452, 1313, 1115, 967. ^1H NMR (400.1 MHz, $CDCl_3$) 3.32 (1H, td, J1 = 7.8 Hz, J = 3.0 Hz, H1), 1.86 (1H, sep, , J = 8.2 Hz, H4), 1.81 – 1.13 (10H, m, H2, H5 – H8), 0.95 (3H, t, J = 7.8 Hz, H3). ^{13}C NMR (100.6 MHz, $CDCl_3$) 76.7 (C1), 45.9 (C4), 29.1 (C5), 28.9 (C6), 28.5 (C2), 25.7 (C7), 25.6 (C8), 10.0 (C3). HRMS (FTMS +p NSI) m/z: $[M + NH_4]^+$ calculated for $C_8H_{20}NO$ 146.1539, found = 146.1537, Δ = -1.4 ppm.

1-cyclopentylpropan-1-one (1v-II)

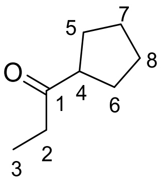

Mr = 126.20

A solution of **1v-I** (1.9 g, 15 mmol) in dichloromethane (200 mL) was cooled to 0 °C. Dess-Martin Periodinane (7.0 g, 16.5 mmol) was then added portion wise. The mixture was stirred for an additional 2 hours, warming up to room temperature. 2.5 M aqueous sodium hydroxide (100 mL) was added and the mixture stirred for 30 minutes. The reaction mixture was extracted with dichloromethane (3 X 100 mL), dried over magnesium sulphate, filtered, and concentrated *in vacuo* (caution, volatile) to give the crude **1v-II** as a colourless liquid (1.5 g, 81%). IR (λ_{max}/cm^{-1})2953, 2869, 1708, 1451, 1413, 1362, 1128, 1023. ^{1}H NMR (400.1 MHz, $CDCl_3$) 2.86 (1H, p, J = 8.0 Hz, H4), 2.45 (2H, q, J = 7.6 Hz, H2), 1.86 – 1.50 (8H, m, H5 – H8), 1.04 (3H, t, J = 8.8 Hz, H3) ^{13}C NMR (100.6 MHz, $CDCl_3$)214.0 (C1), 51.1 (C4), 34.9 (C2), 28.7, 26.0 (C5, C8), 7.9 (C3). HRMS (FTMS +p NSI) m/z: [M + NH_4]$^+$ calculated for $C_8H_{18}NO$ 144.1383, found = 144.1384, Δ = 0.7 ppm.

N-(1-cyclopentylpropyl)tetrahydro-2H-pyran-4-amine (1v)

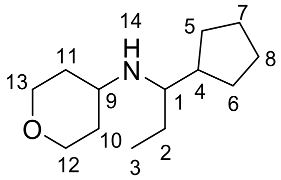

Mr = 211.35

General procedure B was applied to 4-aminotetrahydropyran (1.0 g, 10 mmol), 1 1-**1v-II** (1.3 g, 10 mmol) and Ti(*i*-PrO)$_4$ (5.7 g, 20 mmol) neat. After stirring for 8 hours, the mixture was cooled to 0 °C. Absolute ethanol (40 mL) was added, followed by sodium borohydride (1.1 g, 30 mmol) portion wise and the resultant mixture was stirred for another 8 hours. Purification by silica column (60% to 80% ethyl acetate / petroleum ether) gave the **1v** as a colourless liquid (600 mg, 28%). IR (λ_{max}/cm^{-1}) 2936, 2866, 1463, 1366, 1235, 1141, 1111, 1087. ^{1}H NMR (400.1 MHz, $CDCl_3$) 3.95 (2H, dt, J1 = 11.6 Hz, J2 = 3.4 Hz, H12a, H13a), 3.38 (2H, qd, J1 = 10.8 Hz, J2 = 2.2 Hz, H12b, H13b), 2.67 (1H, tt, J1 = 10.4 Hz, J2 = 4.2 Hz, H9), 2.41 (1H, dt, J1 = 7.4 Hz, J2 = 5.2 Hz, H1), 1.90 – 1.11 (15H, m, H2, H4 – H8, H10, H11), 0.88 (3H, t, J = 8.2 Hz, H3). ^{13}C NMR (100.6 MHz, $CDCl_3$) 67.2 (C12), 67.0 (C13), 59.3 (C1), 51.5 (C9), 44.1 (C4), 35.1 (C10), 34.2 (C11), 29.7 (C7), 29.6 (C8), 25.4 (C2), 25.4 (C5), 25.4 (C6), 9.3 (C3) HRMS (FTMS +p NSI) m/z: [M + H]$^+$ calculated for $C_{13}H_{26}NO$ 212.2009, found = 212.2011, Δ = 0.9 ppm.

1.4 Carbonylation of Amines

General Procedure C for stoichiometric palladium carbonylation of amines

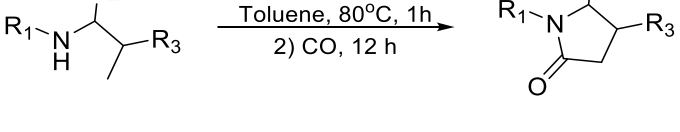

To a 10 mL round-bottomed flask with large oval stirrer bar was added palladium (II) acetate (66 mg, 0.3 mmol, 1 eq), the amine (0.3 mmol, 1 eq) and anhydrous toluene (3 mL). The flask was sealed with a new septum and Teflon tape. The flask was placed into an oil bath at 80 °C and stirred at 400 rpm for 1 hour. A balloon of carbon monoxide was placed on top, and the flask was stirred for a further 12 hours. The reaction mixture was cooled, and the contents were filtered through celite and washed with ethyl acetate. The solvent was removed *in vacuo* and the crude mixture was purified by column chromatography to obtain the corresponding *γ* lactam.

General Procedure D for catalytic palladium carbonylation of amines

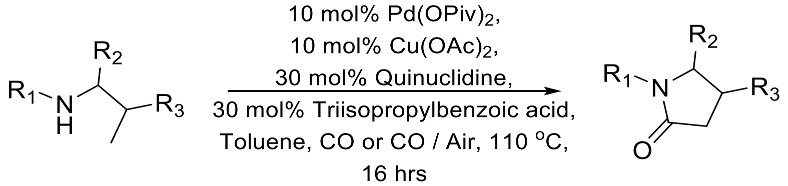

To a 100 mL round-bottomed flask with large oval stirrer bar was added palladium (II) pivalate (9.0 mg, 0.03 mmol, 0.1 eq), copper acetate (5.4 mg, 0.03mmol, 0.1 eq), quinuclidine (9.9 mg, 0.09mmol, 0.3 eq), triisopropylbenzoic acid (22.5 mg, 0.09mmol, 0.3 eq), the amine (0.3 mmol, 1 eq) and anhydrous toluene (6 mL). The flask was sealed with a new septum and parafilm. A balloon of CO was placed on top. The flask was placed into an oil bath at 110 °C, and stirred at 400 rpm for 16 hour. The reaction mixture was cooled, and the contents were filtered through celite and washed with ethyl acetate. The solvent was removed *in vacuo* and the crude mixture was purified by column chromatography to obtain the corresponding *γ* lactam. Separation of cis and trans diastereomers is usually difficult, although the trans diastereomer can sometimes be isolated by repeated column chromatography.

Trans-1-(cyclohexylmethyl)-5-isopropyl-4-methylpyrrolidin-2-one (4a)

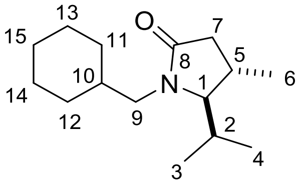

Mr = 237.39

General procedure C was applied to N-(cyclohexylmethyl)-2,4-dimethylpentan-3-amine (63.4 mg, 0.3 mmol). The crude reaction product was purified by alumina column chromatography (0% to 20% ethyl acetate / petroleum ether) to give **4a** as a yellow oil (38.5 mg, 54 %, *d.r.* = 8:1). IR (λ_{max}/cm^{-1}) 2959.2, 2923.7, 2852.2, 1682.6, 1423.5, 1260.5. ^{1}H NMR (400.1 MHz, $CDCl_3$) 3.58 (1H, dd, J_1 = 15.0 Hz, J_2 = 10.3 Hz, H9a), 3.07 (1H, t, J = 2.7 Hz, H1), 2.62 – 2.52 (2H, m, H7a, H9b), 2.19 – 2.10 (1H, m, H5), 2.10 – 2.02 (1H, m, H2), 1.96 (1H, dd, J_1 = 17.5 Hz, J_2 = 3.5 Hz, H7b) 1.77 – 1.54 (8H, m, H10, H11b, H12b, H13, H14, H15b), 1.30 – 1.13 (3H, m, H11a, H12a, H15a), 1.10 (3H, d, J = 7.0 Hz, H6), 0.96 (3H, d, J = 7.1 Hz, H3), 0.74 (3H, d, J = 7.0 Hz, H4). ^{13}C NMR (100.6 MHz, $CDCl_3$) 174.42 (C8), 70.45 (C1), 45.85 (C9), 39.42 (C7), 35.24 (C10), 31.25 (C13), 30.51 (C11), 28.26 (C2), 26.43 (C14), 25.90 (C12), 15.68 (C15), 25.17 (C5), 23.29 (C6), 18.50 (C3), 14.96 (C4). HRMS (FTMS +p NSI) m/z: $[M + H]^+$ calculated for $C_{15}H_{28}NO$ 238.2165, found = 238.2165, exact match.

Trans-1-(cyclohexylmethyl)-5-isopropyl-4-methylpyrrolidin-2-one (4a)

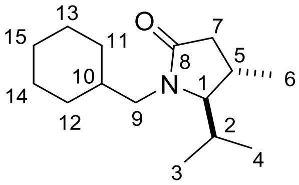

Mr = 237.39

General procedure D was applied to N-(cyclohexylmethyl)-2,4-dimethylpentan-3-amine (63.4 mg, 0.3 mmol). The crude reaction product was purified by alumina column chromatography (0% to 20% ethyl acetate / petroleum ether) to give **4a** as a yellow oil (50.1 mg, 70 %, *d.r.* = 10:1).

Trans-1-(cyclohexylmethyl)-5-isopropyl-4-methylpyrrolidin-2-one (4a)

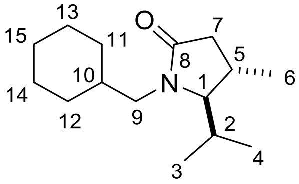

Mr = 237.39

To a 1 L round-bottomed flask with large oval stirrer bar was added palladium (II) pivalate (150 mg, 0.5 mmol, 0.1 eq), copper acetate (90 mg, 0.5 mmol, 0.1 eq), quinuclidine (165 mg, 1.5 mmol, 0.3 eq), triisopropylbenzoic acid (375 mg, 1.5 mmol, 0.3 eq), N-(cyclohexylmethyl)-2,4-dimethylpentan-3-amine (1.05 g, 5 mmol, 1 eq) and anhydrous toluene (100 mL). The flask was sealed with a new septum and parafilm. A balloon of CO was placed on top. The flask was placed into an oil bath at 110 ^{o}C, and stirred at 600 rpm for 16 hour. The reaction mixture was cooled, and the contents were filtered through celite and washed with ethyl acetate. The solvent was removed *in vacuo*, and the crude reaction product was purified by alumina column chromatography (0% to 20% ethyl acetate / petroleum ether) to give **4a** as a yellow oil (807 mg, 68 %, *d.r.* = 10:1).

***Trans*-1-cyclohexyl-5-isopropyl-4-methylpyrrolidin-2-one (4b)**

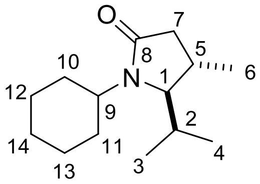

Mr = 223.36

General procedure D was applied to *N*-(2,4-dimethylpentan-3-yl)cyclohexanamine 238 (59.1 mg, 0.3 mmol). The crude reaction product was purified by silica column chromatography (0% to 30% ethyl acetate / petroleum ether) to give **4b** as a yellow oil (44.0 mg, 66 %, *d.r.* = 8:1). IR (λ_{max}/cm^{-1}) 2958, 2930, 1675, 1420, 1253, 894, 751, 663. ^{1}H NMR (400.1 MHz, $CDCl_3$) 3.63 (1H, tt, *J* = 12.0, 3.9 Hz, H9), 3.09 (1H, d, *J* = 2.2 Hz, H1), 2.58 (1H, dd, *J1* = 17.1 Hz, J2 = 8.9 Hz, H7a), 2.13 – 1.94 (2H, m, H2, H5), 1.93 – 1.59 (6H, m, H7b, H10a – H14a), 1.51 (1H, dq, *J1* = 12.3 Hz, J2 = 3.5 Hz, H14b), 1.39 – 1.10 (4H, m, H10b – H13b), 1.03 (3H, d, *J* = 7.1 Hz, H6), 0.94 (3H, d, *J* = 6.9 Hz, H3), 0.80 (3H, d, *J* = 6.8 Hz, H4). ^{13}C NMR (100.6 MHz, $CDCl_3$) 174.3 (C8),

71.1 (C1), 53.3 (C9), 40.2 (C7), 31.6 (C10), 31.4 (C11), 29.9 (C2), 26.2 (C12), 25.7 (C13), 25.6 (C14), 23.3 (C6), 19.0 (C3), 15.1 (C4). HRMS (ESI) m/z: calculated for $C_{14}H_{26}ON$ $[M+H]^+$ 224.2009, found 224.2008, Δ = - 0.4 ppm.

Trans-1-cyclopentyl-5-isopropyl-4-methylpyrrolidin-2-one (4c)

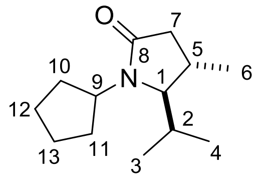

Mr = 209.33

General procedure D was applied to N-(2,4-dimethylpentan-3-yl)cyclopentanamine (55.0 mg, 0.3 mmol). The crude reaction product was purified by alumina column chromatography (5% to 25% ethyl acetate / petroleum ether) to give **4c** as a yellow oil (40.8 mg, 65%, *d.r.* = 11:1). IR (λ_{max}/cm^{-1})2957, 2869, 1674, 1449, 1422, 1373, 1322, 1263, 1234. ^1H NMR (400.1 MHz, $CDCl_3$) 3.96 – 3.81 (1H, m, H9), 3.06 (1H, d, J = 2.1 Hz, H1), 2.58 (1H, dd, J1 = 17.5 Hz, J2 = 8.6 Hz, H7a), 2.14 – 2.09 (1H, m, H5), 2.04 – 1.97 (1H, m, H2), 1.96 – 1.48 (9H, m, H7b, H10 – H13), 1.05 (3H, d, J = 7.5 Hz, H6), 0.94 (3H, d, J = 6.8 Hz, H3), 0.80 (3H, d, J = 7.0 Hz, H4). ^{13}C NMR (100.6 MHz, $CDCl_3$) 174.3 (C8), 72.2 (C1), 55.1 (C9), 40.2 (C7), 30.9 (C2), 29.7 (C10), 28.3 (C11), 25.5 (C5), 23.9 (C12), 23.8 (C13), 23.2 (C6), 18.8 (C3), 15.0 (C4). HRMS (FTMS +p NSI) m/z: $[M + H]^+$ calculated for $C_{15}H_{28}NO$ 210.1852, found = 210.1852, exact match.

Trans 5-isopropyl-4-methyl-1-(tetrahydro-2H-pyran-4-yl)pyrrolidin-2-one (4d)

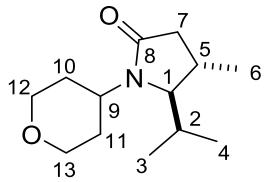

Mr = 225.33

General procedure D was applied to N-(2,4-dimethylpentan-3-yl)tetrahydro-2H-pyran-4-amine (59.8 mg, 0.3 mmol). The crude reaction product was purified by alumina column chromatography (50% to 90% ethyl acetate / petroleum ether) to give **4d** as a yellow oil (55.0 mg, 74%, *d.r.* = 8:1). IR (λ_{max}/cm^{-1}) 2957, 2870, 1673, 1421, 1375, 1145. ^1H NMR (400.1 MHz, $CDCl_3$) 4.08 – 3.92 (3H, m, H9, H12a, H13a), 3.50 – 3.40 (2H, m, H12b, H13b), 3.11 (1H, dd, J1 = 3.2 Hz, J2 = 1.0Hz, H1), 2.60 (1H, dd, J1 = 17.6 Hz, J2 = 9.0 Hz, H7a), 2.19 – 1.99 (3H, m, H5, H10a, H11a), 1.88 (1H, dd, J1 = 16.8 Hz, J2 = 2.4 Hz, H7b), 1.87 – 1.77 (2H, m, H2, H10b), 1.59 – 1.52 (1H, m, H11), 1.04 (3H, d, J = 7.3 Hz, H6), 0.95 (3H, d, J = 7.0 Hz, H3), 0.80 (3H, d, J = 7.0 Hz, H4). ^{13}C NMR (100.6 MHz, $CDCl_3$) 174.5 (C8), 70.7 (C1), 67.9 (C12), 67.7 (C13), 50.1 (C9), 39.8 (C7), 31.6 (C2), 31.2 (C10), 29.7 (C11), 25.5 (C5), 23.1 (C6), 18.9 (C3), 14.8 (C4) HRMS (FTMS +p NSI) m/z: $[M + H]^+$ calculated for $C_{13}H_{24}NO_2$ 226.1802, found = 226.1801, Δ = -0.4 ppm.

Benzyl-4-(trans-2-isopropyl-3-methyl-5-oxopyrrolidin-1-yl)piperidine-1-carboxylate (4e)

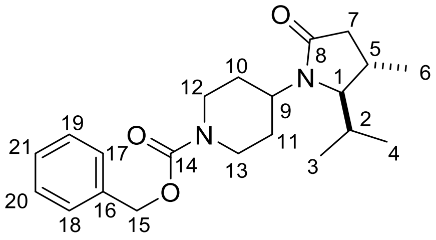

Mr = 358.48

General procedure D was applied to benzyl 4-((2,4-dimethylpentan-3-yl)amino)piperidine-1-carboxylate (99.7 mg, 0.3 mmol). The crude reaction product was purified by alumina column chromatography (70% to 100% ethyl acetate / petroleum ether) to give **4e** as a yellow oil (71.7 mg, 70%, *d.r.* = 8:1). IR (λ_{max}/cm^{-1}) 2959, 2871, 1671, 1422, 1233, 1127. ^{1}H NMR (400.1 MHz, $CDCl_3$) 7.37 – 7.27 (5H, m, H17 – H21), 5.11 (2H, s, H15), 4.27 (2H, br, H12, H13), 3.86 (2H, tt, J1 = 12.5 Hz, J2 = 4.0 Hz, H9), 3.05 (1H, d, J = 2.5 Hz, H1), 2.81 (2H, br, H12, H13), 2.58 (1H, dd, J1 = 17.0 Hz, J2 = 9.2 Hz, H7a). 2.17 – 2.07 (1H, m, H5), 2.00 – 1.81 (3H, m, H10a, H11a, H7b), 1.73 – 1.57 (2H, m, H10b, H11b), 1.02 (3H, d, J = 7.5 Hz, H6), 0.92 (3H, d, J = 7.1 Hz, H3), 0.77 (3H, d, J = 7.1 Hz, H4). ^{13}C NMR (100.6 MHz, $CDCl_3$) 174.4 (C8), 155.1 (C1), 136.7 (C16), 128.49, 128.03, 127.99, 127.94 (C17 – C21), 70.7 (C1), 67.2 (C15), 51.2 (C9), 13.8 (C12), 43.7 (C13), 39.8 (C7), 31.2 (C2), 28.7 (C10, C11), 25.5 (C5), 23.1 (C6), 18.2 (C3), 14.8 (C4). HRMS (FTMS +p NSI) m/z: $[M + H]^+$ calculated for $C_{21}H_{31}N_2O_3$ 359.2329, found = 359.2332, Δ = 0.8 ppm.

Trans-1-(4,4-difluorocyclohexyl)-5-isopropyl-4-methylpyrrolidin-2-one (4f)

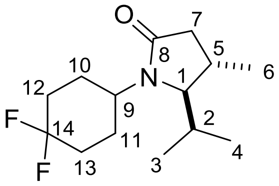

Mr = 259.34

General procedure D was applied to N-(2,4-dimethylpentan-3-yl)-4,4-difluorocyclohexan-1-amine (70.0 mg, 0.3 mmol). The crude reaction product was purified by alumina column chromatography (50% to 20% ethyl acetate / petroleum ether) to give **4f** as a yellow oil (52.0 mg, 67%, *d.r.* = 11 : 1). IR (λ_{max}/cm^{-1}) 2962, 2877, 1670, 1443, 1423, 1378, 1269, 1105, 956. ^{1}H NMR (400.1 MHz, $CDCl_3$) 3.93 -3.81 (1H, m, H9), 3.10 (1H, d, J = 2.9 Hz, H1), 2.59 (1H, dd, J1 = 17.1 Hz, J2 = 9.6 Hz, H7a), 2.27 – 1.61 (11H, m, H2, H5, H7b, H10 – H13), 1.04 (3H, d, J = 6.7 Hz, H6), 0.96 (3H, d, J = 6.6 Hz, H3), 0.80 (3H, d, J = 6.7 Hz, H4). ^{13}C NMR (100.6 MHz, $CDCl_3$) 174.5 (C8), 70.6 (C1), 50.5 (C9), 39.6 (C7), 33.1 (C14, t), 31.4 (C10), 27.1 (C12), 25.5 (C11), 25.4 (C13), 23.2 (C6), 18.9 (C3), 14.8 (C4). ^{19}F NMR (376.5 MHz, $CDCl_3$) -94.0 (d), -103.5 (d) HRMS (FTMS +p NSI) m/z: $[M + H]^+$ calculated for $C_{14}H_{24}F_2NO$ 260.1820, found = 260.1822, Δ = 0.8 ppm.

Trans-5-isopropyl-4-methyl-1-(1,4-dioxaspiro[4.5]decan-8-yl)pyrrolidin-2-one (4g)

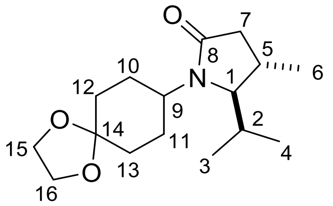

Mr = 281.40

General procedure D was applied to N-(2,4-dimethylpentan-3-yl)-1,4-dioxaspiro[4.5]decan-8-amine (76.6 mg, 0.3 mmol). The crude reaction product was purified by alumina column chromatography (50% to 70% ethyl acetate / petroleum ether) to give **4g** as a yellow oil (59.1 mg, 70%, *d.r.* = 13 : 1). IR (λ_{max}/cm^{-1}) 2957, 2874, 1671, 1420, 1265, 1102. ^{1}H NMR (400.1 MHz, $CDCl_3$) 3.98 – 3.84 (5H, m H9, H15, H16), 3.12 (1H, s, H1), 2.58 (1H, dd, J1 = 17.4 Hz, J2 = 9.1 Hz, H7a), 2.17 – 1.60 (11H, m, H2, H5, H7b, H10 – H13), 1.02 (3H, d, J = 7.1 Hz, H6), 0.94 (3H, d, J = 7.1 Hz, H3), 0.79 (3H, d, J = 6.9 Hz, H4). ^{13}C NMR (100.6 MHz, $CDCl_3$) 174.3 (C8), 107.9 (C14), 70.2 (C1), 64.4 (C15, C16), 51.2 (C9), 39.8 (C7), 34.1 (C12), 34.1 (C13), 31.4 (C2), 28.6 (C10), 26.6 (C11), 25.4 (C5), 23.2 (C6), 18.9 (C3), 14.8 (C4). HRMS (FTMS +p NSI) m/z: $[M + H]^+$ calculated for $C_{16}H_{28}NO_3$ 282.2064, found = 282.2065, Δ = 0.4 ppm.

Trans-1-(2,2-dimethyl-1,3-dioxan-5-yl)-5-isopropyl-4-methylpyrrolidin-2-one (4h)

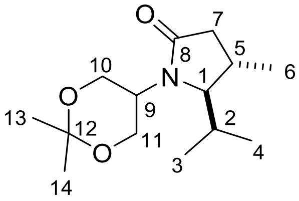

Mr = 255.36

General procedure D was applied to N-(2,4-dimethylpentan-3-yl)-2,2-dimethyl-1,3-dioxan-5-amine (68.8 mg, 0.3 mmol). The crude reaction product was purified by alumina column chromatography (10% to 40% ethyl acetate / petroleum ether) to give **4h** as a yellow oil (54.2 mg, 71%, *d.r.* = 14 : 1). IR (λ_{max}/cm^{-1}) 2961, 2875, 1675, 1423, 1372, 1257, 1199, 1123, 1092. ^{1}H NMR (400.1 MHz, $CDCl_3$) 4.51 (1H, dd, J1 = 10.6 Hz, J2 = 8.5 Hz, H10a) 4.37 (1H, dd, J1 = 11.6 Hz, J2 = 8.1 Hz, H11a), 3.87 – 3.80 (2H, m, H10b, H11b), 3.70 – 3.62 (1H, m, H9) 3.17 (1H, d, J = 2.4 Hz, H1), 2.59 (1H, dd, J1 = 17.5 Hz, J2 = 9.5 Hz, H7a) 2.26 – 2.14 (2H, m, H2, H5), 1.87 (1H, J1 = 17.5 Hz, J2 = 1.8 Hz, J7b), 1.55 (3H, s, H13), 1.43 (3H, s, H14), 1.09 (3H, d, J = 7.0 Hz, H6), 0.98 (3H, d, J = 6.7 Hz, H3), 0.83 (3H, d, J = 7.0 Hz, H4). ^{13}C NMR (100.6 MHz, $CDCl_3$) 175.2 (C8), 98.1 (C12), 73.0 (C1), 60.7 (C10), 59.9 (C11), 48.0 (C9), 39.7 (C7), 30.3 (C2), 26.0 (C5), 25.9 (C14), 23.1 (C6), 21.8 (C13), 18.8 (C3), 15.1 (C4). HRMS (FTMS +p NSI) m/z: $[M + H]^+$ calculated for $C_{14}H_{26}NO_3$ 256.1907, found = 256.1910, Δ = 1.2 ppm.

Tert-butyl 3-(trans-2-isopropyl-3-methyl-5-oxopyrrolidin-1-yl)azetidine-1-carboxylate (4i)

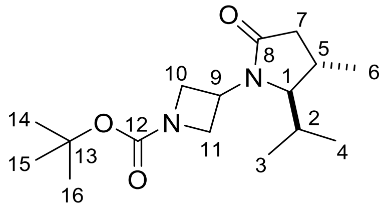

Mr = 296.41

General procedure D was applied to tert-butyl 3-((2,4-dimethylpentan-3-yl)amino)azetidine-1-carboxylate (81.1 mg, 0.3 mmol). The crude reaction product was purified by alumina column chromatography (40% to 60% ethyl acetate / petroleum ether) to give **4i** as a yellow oil (44.3 mg, 50%, *d.r.* = 11:1). IR (λ_{max}/cm^{-1}) 2963, 1685, 1390, 1364, 1255, 1132. ^{1}H NMR (400.1 MHz, $CDCl_3$) 4.37 – 4.02 (5H, m, H9 – H11), 3.16 (1H, dd, 3.7 Hz, 1.4 Hz, H1), 2.57 (1H, dd, J1 = 17.3 Hz, J2 = 9.3 Hz, H7a), 2.21 – 2.12 (1H, m, H5), 1.93 (1H, dd, J1 = 17.1 Hz, J2 = 2.7 Hz, H7b), 1.89 – 1.82 (1H, m, H2), 1.10 (3H, d, J = 6.9 Hz, H6), 0.94 (3H, d, J = 7.1 Hz, H3), 0.78 (3H, d, J = 6.9 Hz, H4). ^{13}C NMR (100.6 MHz, $CDCl_3$) 175.0 (C8), 156.4 (C12), 79.8 (C13), 71.4 (C1), 53.8 (C10 – C11), 42.9 (C9), 39.6 (C7), 30.9 (C2), 28.4 (C14 – C16), 25.9 (C5), 23.1 (C6), 18.5 (C3), 15.5 (C4). HRMS (FTMS +p NSI) m/z: [M + H]$^+$ calculated for $C_{16}H_{29}N_2O_3$ 297.2173, found = 297.2174, Δ = 0.3 ppm.

Trans-5-isopropyl-4-methyl-1-(2-((triisopropylsilyl)oxy)ethyl)pyrrolidin-2-one (4j)

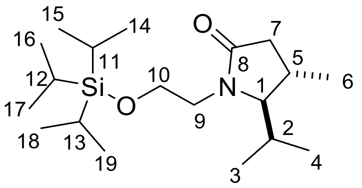

Mr = 341.61

General procedure D was applied to 2,4-dimethyl-N-(2-((triisopropylsilyl)oxy)ethyl)pentan-3-amine (94.7 mg, 0.3 mmol) with 2-methyl-6-nitrobenzoic acid (16.3 mg, 0.09 mmol) in place of triisopropylbenzoic acid. The crude reaction product was purified by alumina column chromatography (10% to 30% ethyl acetate / petroleum ether) to give **4j** as a yellow oil (48.1 mg, 47%, *d.r.* = 5:1). IR (λ_{max}/cm^{-1}) 2959, 2942, 2866, 1682, 1462, 1258, 1104. ^{1}H NMR (400.1 MHz, $CDCl_3$) 3.90 – 3.77 (3H, m, H9a, H10), 3.39 (1H, t, J = 3.0 Hz, H1), 3.02 – 2.93 (1H, m, H9b), 2.56 (1H, dd, J1 = 17.8 Hz, J2 = 9.2 Hz, H7a), 2.21 – 2.08 (2H, m, H2, H5), 1.93 (1H, dd, J1 = 17.4 Hz, J2 = 3.4 Hz, H7b), 1.09 (3H, d, J = 7.0 Hz, H6), 1.07 – 1.03 (21H, m, H11-H19), 0.94 (3H, d, J = 7.0 Hz, H3), 0.74 (3H, d, J = 7.0 Hz, H4). ^{13}C NMR (100.6 MHz, $CDCl_3$) 174.4 (C8), 71.5 (C1), 62.1 (C10), 42.8 (C9), 39.2 (C7), 28.4 (C5), 25.4 (C2), 23.0 (C6), 18.5 (C3), 18.0 (C14 – C19), 15.0 (C4), 11.9 (C11- C13). HRMS (FTMS +p NSI) m/z: [M + H]$^+$ calculated for $C_{19}H_{40}NO_2Si$ 342.2823, found = 342.2824, Δ = 0.3 ppm.

Trans-5-isopropyl-4-methyl-1-(2-(pyridin-2-yloxy)ethyl)pyrrolidin-2-one (4k)

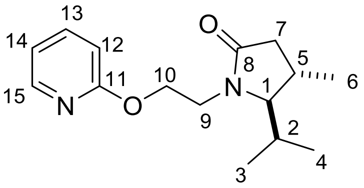

Mr = 262.35

General procedure D was applied to 2,4-dimethyl-N-(2-(pyridin-2-yloxy)ethyl)pentan-3-amine (70.9 mg, 0.3 mmol) with 2-methyl-6-nitrobenzoic acid (16.3 mg, 0.09 mmol) in place of triisopropylbenzoic acid. The crude reaction product was purified by alumina column chromatography (30% to 50% ethyl acetate / petroleum ether) to give **4k** as a yellow oil (60.0 mg, 76%, *d.r.* = 5:1). IR (λ_{max}/cm^{-1}) 2960, 1680, 1595, 1570, 1466, 1431, 1273, 1142. ^{1}H NMR (400.1 MHz, $CDCl_3$) 8.14 – 8.10 (1H, m, H15), 7.58 – 7.52 (1H, m, H13), 6.87 – 6.83 (1H, m, H14), 4.53 – 4.45 (1H, m, H10a), 4.46 – 4.38 (1H, m, H10b), 4.07 (1H, ddd, J1 = 14.5 Hz, J2 = 6.3 Hz, J3 = 5.7 Hz, H9a), 3.29 – 3.25 (1H, m, H9b), 3.23 (1H, t, J = 3.1 Hz, H1), 2.57 (1H, dd, J1 = 17.7 Hz, J2 = 9.3 Hz, H7a), 2.19 – 2.04 (2H, m, H2, H5), 1.92 (1H, dd, J1 = 17.4 Hz, J2 = 2.9 Hz, H7b), 1.04 (3H, d, J = 6.5 Hz, H6), 0.94 (3H, d, J = 7.4 Hz, H3), 0.76 (3H, d, J = 7.5 Hz, H4). ^{13}C NMR (100.6 MHz, $CDCl_3$) 174.7 (C8), 163.3 (C11), 146.9 (C15), 138.7 (C13), 117.0 (C14), 111.0 (C12), 71.2 (C1), 63.0 (C10), 39.7 (C9), 39.2 (C7), 28.7 (C2), 25.7 (C5), 22.9 (C6), 18.5 (C3), 15.2 (C4). HRMS (FTMS +p NSI) m/z: [M + H]$^{+}$ calculated for $C_{15}H_{23}N_2O_2$ 263.1754, found = 263.1756, Δ = 0.8 ppm.

***trans*-5-Isopropyl-4-methyl-1-(2-((6-(trifluoromethyl)pyridin-2-yl)oxy)ethyl)-γ-lactam (4l)**

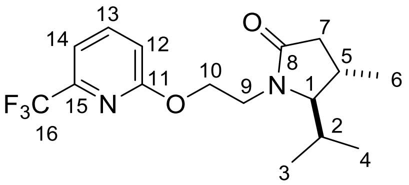

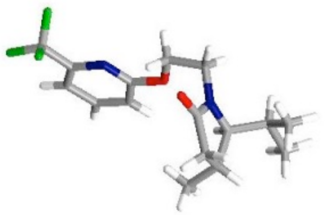

Mr = 330.35

General procedure D was applied to 2,4-dimethyl-N-(2-((6-(trifluoromethyl)pyridin-2-yl)oxy)ethyl)pentan-3-amine (91 mg, 0.3 mmol). The crude reaction product was purified by silica column chromatography (0% to 40% ethyl acetate / petroleum ether) to give **4l** as a white solid (49.1 mg, 49 %, *d.r.* = 9:1). Crystals of 262 suitable for X-ray were grown *via* recrystallization in Et_2O. IR (λ_{max}/cm^{-1}) 2963, 1670, 1266, 1133, 1124, 823. ^{1}H NMR (4000.1 MHz, $CDCl_3$) 7.70 (1H, dd, *J1* = 11.7 Hz, J2 = 4.0 Hz, H13), 7.26 (1H, d, *J* = 7.3 Hz, H12), 6.89 (1H, d, *J* = 8.4 Hz, H14), 4.68 – 4.37 (2H, m, H10), 4.05 (1H, dt, *J* = 14.3, 6.1 Hz, H9a), 3.29 (1H, dt, *J1* = 14.2 Hz, J2 = 5.8 Hz, H9b), 3.25 – 3.10 (1H, m, H1), 2.59 (1H, dd, *J1* = 17.3 Hz, J2 = 9.3 Hz, H7a), 2.27 – 2.03 (2H, m, H2, H5), 1.92 (1H, dd, *J1* = 17.3 Hz, J2 = 3.0 Hz, H7b), 1.03 (3H, d, *J* = 7.0 Hz, H6), 0.96 (3H, d, *J* = 7.0 Hz, H3), 0.77 (3H, d, *J* = 6.8 Hz, H4). ^{13}C NMR (100.6 MHz, $CDCl_3$) 174.9 (C8), 163.4 (C9), 145.6 (q, *J* = 34.9 Hz, C15), 139.7 (C13), 121.4 (q, *J* = 273.6 Hz, C16), 114.8 (C12), 113.6 (q, *J* = 3.2 Hz, C14), 71.3 (C10), 63.3 (C1), 39.5 (C9), 39.3 (C7), 28.9 (C2), 25.8 (C5), 23.0 (C6), 18.7 (C3), 15.3 (C4). ^{19}F NMR (376.5 MHz, $CDCl_3$) - 68.48. HRMS (ESI) m/z: calculated for $C_{16}H_{22}O_2N_2F_3$ [M+H]$^{+}$ 331.1628, found 331.1631 Δ = 0.8 ppm.

Trans-5-cyclohexyl-1-(cyclohexylmethyl)-4-methylpyrrolidin-2-one **(4m)**

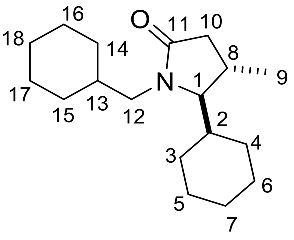

General procedure D was applied to 1-cyclohexyl-N-(cyclohexylmethyl)-2-methylpropan-1-amine (75.4 mg, 0.3 mmol). The crude reaction product was purified by silica column chromatography (0% to 30% ethyl acetate / petroleum ether) to give **4m** as a yellow oil (53 mg, 62%, *d.r.* = 8 : 1). IR (λ_{max}/cm^{-1}) 2923, 2847, 1672, 1448, 1424, 1271, 1250, 894; ^{1}H NMR (400.1 MHz, $CDCl_3$) 3.56 (1H, dd, *J1* = 13.8 Hz, J2 = 9.1 Hz, H12a), 3.07 – 2.93 (1H, m, H4), 2.63 – 2.51 (2H, m, H9b, H10a), 2.23 – 2.11 (1H, m, H8), 1.94 (1H, dd, *J1* = 17.2 Hz, J2 = 2.8 Hz, H10b), 1.86 – 1.40 (12H, m, H2a – H7a, H13a – H18a), 1.37 – 1.10 (6H, m, H3b, H4b, H7b, H14b, H15b, H18b), 1.08 (3H, d, *J* = 7.0 Hz, H9), 1.05 – 0.77 (4H, m, H5b, H6b, H16b, H17b). ^{13}C NMR (100.6 MHz, $CDCl_3$) 174.5, 70.4, 46.1, 39.5, 39.2, 35.5, 31.4, 30.7, 29.4, 26.7, 26.7, 26.6, 26.2, 26.0, 25.9, 25.8, 23.3. HRMS (ESI) m/z: calculated for $C_{18}H_{32}ON$ $[M+H]^+$ 278.2478, found 278.2478, exact match.

Trans-1-(cyclohexylmethyl)-4-methyl-5-(tetrahydro-2H-pyran-4-yl)pyrrolidin-2-one (4n)

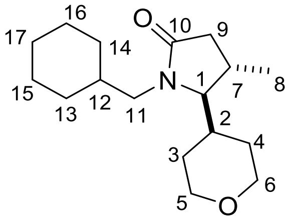

General procedure D was applied to N-(cyclohexylmethyl)-2-methyl-1-(tetrahydro-2H-pyran-4-yl)propan-1-amine (76.0 mg, 0.3 mmol). The crude reaction product was purified by silica column chromatography (100% ethyl acetate) to give **4n** as a yellow oil (49.1 mg, 55%). IR (λ_{max}/cm^{-1}) 2914, 2847, 1674, 1435, 1423, 1091. ^{1}H NMR (400.1 MHz, $CDCl_3$) 4.12 – 3.93 (2H, m, H5a, H6a), 3.60 (1H, dd, *J1* = 13.8 Hz, J2 = 9.2 Hz, H11a), 3.40 (1H, td, *J1* = 11.7 Hz, J2 = 2.3 Hz, H5b), 3.36 – 3.25 (1H, m, H6b), 3.13 – 3.02 (1H, m, H1), 2.65 – 2.48 (2H, m, H9a, H11b), 2.27 – 2.17 (1H, m, H7), 1.97 (1H, dd, *J1* = 17.3 Hz, J2 = 2.7 Hz, H9b), 1.98 – 1.86 (1H, m, H2), 1.78 – 1.48 (7H, m, H3a, H4a, H13a - H17a), 1.45 – 1.37 (1H, m, H12a), 1.35 – 1.13 (5H, m, H3b, H4b, H13b, H14b, H17b), 1.11 (3H, d, *J* = 7.0 Hz, H8), 1.06 – 0.86 (2H, m, H15b, H16b). ^{13}C NMR (100.6 MHz, $CDCl_3$) 174.5 (C10), 69.6 (C1), 68.1 (C5), 68.1 (C6), 46.2 (C11), 39.2 (C9), 36.7 (C2), 35.5 (C12), 31.4 (C13), 30.6 (C4), 29.0 (C3), 26.6 (C7), 26.5 (C17), 26.3 (C10), 26.0 (C15), 25.8 (C16), 23.2 (C8). HRMS (ESI) m/z: calculated for $C_{17}H_{30}O_2N$ $[M+H]^+$ 280.2271, found 280.2274, Δ = 1.0 ppm.

Trans-1-(cyclohexylmethyl)-5-cyclopentyl-4-methylpyrrolidin-2-one (4o)

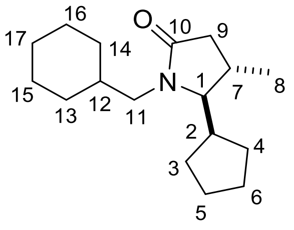

Mr = 263.43

General procedure D was applied to N-(cyclohexylmethyl)-1-cyclopentyl-2-methylpropan-1-amine (71 mg, 0.3 mmol). The crude reaction product was purified by silica column chromatography (0% to 35% ethyl acetate / petroleum ether) to give **4o** as a yellow oil (49.1 mg, 56%, d.r. = 6 : 1). IR (λ_{max}/cm^{-1}) 2922, 2851, 1681, 1447, 1422. ^{1}H NMR (400.1 MHz, $CDCl_3$) 3.61 (1H, dd, *J1* = 13.7 Hz, J2 = 9.2 Hz, H11a), 3.23 (1H, dd, *J1* = 4.8 Hz, J2 = 2.0 Hz, H1), 2.73 – 2.60 (2H, m, H9a, H11b), 2.28 – 2.10 (2H, m, H2, H7), 1.99 (1H, dd, *J1* = 17.2 Hz, J2 = 2.8 Hz, H9b), 1.81 – 1.45 (12H, m, H3, H4, H5a, H6a, H12a – H17a), 1.36 – 1.10 (5H, m, H5b, H6b, H13b, H14b, H17b), 1.13 (3H, d, *J* = 7.0 Hz, H8), 1.06 – 0.86 (2H, m, H15b, H16b). ^{13}C NMR (100.6 MHz, $CDCl_3$) 174.3 (C10), 68.5 (C1), 46.5 (C11), 42.3 (C7), 39.3 (C9), 35.5 (C12), 27.8 (C2), 31.3, 30.6, 29.0, 27.4, 26.5, 26.0, 25.8, 25.8, 25.4 (C3 – C6, C13 – C17), 23.1 (C8). HRMS (ESI) m/z: calculated for $C_{17}H_{30}ON$ $[M+H]^+$ 264.2322, found 264.2324, Δ = 0.8 ppm.

Benzyl 4-(trans-3-methyl-5-oxo-1-(tetrahydro-2H-pyran-4-yl)pyrrolidin-2-yl)piperidine-1-carboxylate (4p)

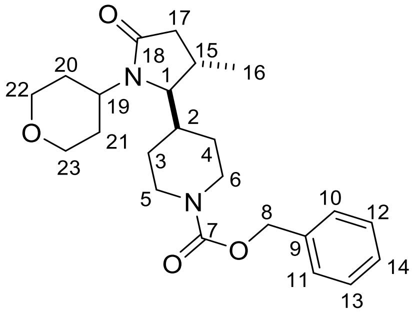

Mr = 400.52

General procedure D was applied to benzyl 4-(2-methyl-1-((tetrahydro-2H-pyran-4-yl)amino)propyl)piperidine-1-carboxylate (112.3 mg, 0.3 mmol). The crude reaction product was purified by alumina column chromatography (70% to 100% ethyl acetate / petroleum ether) to give **4p** as a yellow oil (66.1 mg, 55%, *d.r.* = 8:1). IR (λ_{max}/cm^{-1}) 2958, 1680, 1423, 1274, 1224, 1144, 1085. ^{1}H NMR (400.1 MHz, $CDCl_3$) 7.39 – 7.27 (5H, m, H10 – H14), 5.12 (2H, s, H8), 4.30 (2H, br, H5a, H6a), 4.10 – 3.95 (3H, m, H19, H22a, H23a) 3.51 – 3.41 (2H, m, H22b, H23b), 3.14 (1H, d, J = 2.7 Hz, H1), 2.76 (1H, br, H5b), 2.63 (1H, br, H6b), 2.55 (1H, dd, J1 = 16.9 Hz, J2 = 6.7 Hz, H17a), 2.17 – 1.96 (3H, m, H15, H17b, H21), 1.93 – 1.70 (5H, m, H2, H3a, H4a, H20), 1.61 – 1.47 (3H, m, H3b, H4b, H21), 1.03 (3H, d, J = 6.9 Hz, H16). ^{13}C NMR (100.6 MHz, $CDCl_3$) 174.3 (C18), 155.2 (C7), 136.7 (C9), 128.5, 128.1, 128.0 (C10 – C14), 69.3 (C1), 67.6 (C22), 67.5 (C23), 67.2 (C8), 50.1 (C19), 44.2 (C5), 44.2 (C6), 40.7 (C15), 39.5 (C17), 31.9 (C20), 29.8 (C21), 28.5 (C3), 26.8 (C15), 25.1 (C4), 22.9 (C16). HRMS (FTMS +p NSI) m/z: $[M + H]^+$ calculated for $C_{23}H_{33}N_2O_4$ 401.2135, found = 401.2432, Δ = -0.7 ppm.

(4S,5S)-1-(cyclohexylmethyl)-5-(1,3-dioxolan-2-yl)-4-methylpyrrolidin-2-one (4q)

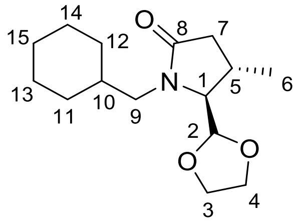

Mr = 267.34

General procedure D was applied to N-(cyclohexylmethyl)-1-(1,3-dioxolan-2-yl)-2-methylpropan-1-amine (72.0 mg, 0.3 mmol). The crude reaction product was purified by silica column chromatography (0% to 35% ethyl acetate / petroleum ether) to give **4q** as a yellow oil (52.3 mg, 48%, d.r. = 1 : 1). IR (λ_{max}/cm^{-1}) 2923, 1686, 1120, 1028, 944. ^{1}H NMR (400.1 MHz, $CDCl_3$) 4.88 (1H, d, *J* = 3.8 Hz, H2), 4.11 – 3.78 (4H, m, H3, H4), 3.59 (1H, dd, *J1* = 13.7 Hz, J2 = 8.8 Hz, H9a), 3.29 (1H, dd, *J*1 = 3.8 Hz, J2 = 2.1 Hz, H1), 2.95 (1H, dd, *J1* = 13.6 Hz, J2 = 5.1 Hz, H9b), 2.72 (1H, ddd, *J1* = 16.8, J2 = 8.7 Hz, J3 = 0.7 Hz, H7a), 2.43 – 2.28 (1H, m, H5), 1.95 (1H, dd, *J1* = 16.8 Hz, J2 = 2.8 Hz, H7b), 1.82 – 1.52 (6H, m, H10a – H15a), 1.33 – 1.09 (2H, m, H11b, H12b), 1.17 (3H, d, *J* = 7.1 Hz, H6), 1.10 – 0.77 (3H, m, H13b – H15b). ^{13}C NMR (100.6 MHz, $CDCl_3$) 175.4 (C8), 104.8 (C2), 66.9 (C1), 65.7 (C3), 65.0 (C4), 47.8 (C9), 38.6 (C7), 35.7 (C10), 31.3 (C11), 30.7 (C12), 27.7 (C5), 26.6 (C15), 26.1 (C13), 25.9 (C14), 21.8 (C6). HRMS (ESI) m/z: calculated for $C_{15}H_{26}O_3N$ $[M+H]^+$ 268.1907, found 268.1908, Δ = 0.4 ppm.

(4S,5S)-1-(cyclohexylmethyl)-4-methyl-5-(4-methyl-2,6,7-trioxabicyclo[2.2.2]octan-1-yl)pyrrolidin-2-one (4r)

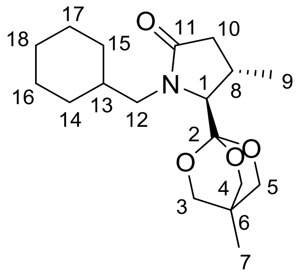

Mr = 323.43

General procedure D was applied to N-(cyclohexylmethyl)-2-methyl-1-(4-methyl-2,6,7-trioxabicyclo[2.2.2]octan-1-yl)propan-1-amine (89.1mg, 0.3 mmol). The crude reaction product was purified by silica column chromatography (0% to 40% ethyl acetate / petroleum ether) to give **4r** as a yellow oil (52.8 mg, 54%, d.r. = 5 : 1). $[\alpha]_D^{20}$= -8.6° (c. 0.9, $CHCl_3$). IR (λ_{max}/cm^{-1}) 2925, 1683, 1046, 987, 736; ^{1}H NMR (400.1 MHz, $CDCl_3$) 3.89 (6H, s, H3- H6), 3.56 (1H, dd, *J1* = 13.6 Hz, J2 = 9.2 Hz, H12a), 3.22 (1H, d, *J* = 1.1 Hz, H1), 3.05 (1H, dd, *J1* = 13.6 Hz, J2 = 5.0 Hz, H12b), 2.73 (1H, dd, *J1* = 16.5 Hz, J2 = 8.7 Hz, H10a), 2.59 – 2.48 (1H, m, H8), 1.83 (1H, dd, *J*1 = 16.6 Hz, J2 = 1.7 Hz, H10b), 1.80 – 1.50 (6H, m, H13a – H18a), 1.38 – 1.12 (3H, m, H13b, H14b, H18b), 1.10 (3H, d, *J* = 7.2 Hz, H9), 1.06 – 0.85 (2H, m, H16b, H17b), 0.82 (3H, s, H7). ^{13}C NMR (100.6 MHz, $CDCl_3$) 176.0 (C11), 109.3 (C2), 72.5 (C3 - C6), 68.1 (C1), 47.7 (C12), 38.5 (C10), 35.0 (C13), 31.1 (C6), 30.7 (C14), 30.5 (C15), 27.5 (C8), 26.5 (C18), 26.0 (C16), 25.7 (C17), 22.0 (C9), 14.4 (C7). HRMS (ESI) m/z: calculated for $C_{18}H_{30}O_4N$ $[M+H]^+$ 324.2169, found 324.2161, Δ = - 2.5 ppm.

(4R,5S)-1-(cyclohexylmethyl)-4-ethyl-5-(4-methyl-2,6,7-trioxabicyclo[2.2.2]octan-1-yl)pyrrolidin-2-one (4s)

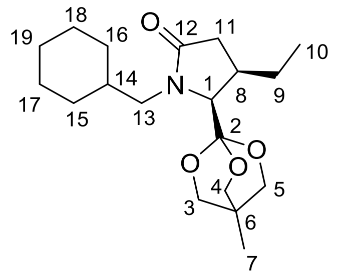

Mr = 337.46

General procedure D was applied to (1S,2S)-N-(cyclohexylmethyl)-2-methyl-1-(4-methyl-2,6,7-trioxabicyclo[2.2.2]octan-1-yl)butan-1-amine (93.4 mg, 0.3 mmol) with 2-methyl-6-nitrobenzoic acid (16.3 mg, 0.09 mmol) in place of triisopropylbenzoic acid. The crude reaction product was purified by alumina column chromatography (10% to 40% ethyl acetate / petroleum ether) to give **4s** as a yellow oil (57.5 mg, 57%). IR (λ_{max}/cm^{-1}) 2959, 2923, 2874, 1683, 1452, 1419, 1266, 1065, 1042. ^{1}H NMR (400.1 MHz, $CDCl_3$) 3.81 (6H, s, H3 – H5), 3.59 (1H, dd, J1 = 14.2 Hz, J2 = 9.2 Hz, H13a), 3.49 (1H, d, J = 6.3 Hz ,H1) 3.05 (1H, dd, J1 = 14.6 Hz, J2 = 6.3 Hz, H13b), 2.30 – 2.18 (2H, m, H8, H11a), 1.83 – 1.48 (9H, m, H9 H11b, H14a, H15a, H16a, H17a, H18a, H19a), 1.28 – 1.09 (3H, m, H15b, H16b, H19b), 0.99 – 0.81 (5H, m, H17b, H18b, H10), 0.78 (3H, s, H7). ^{13}C NMR (100.6 MHz, $CDCl_3$) 176.4 (C12), 110.1 (C2), 72.5 (C3 – C5), 62.9 (C1), 48.6 (C13), 40.3 (C8), 36.5 (C11), 36.4 (C14), 31.0 (C6), 30.6, 30.5, 26.6, 26.0, 25.8 (C15 – C19), 22.8 (C9), 14.5 (C7), 13.2 (C10). HRMS (FTMS +p NSI) m/z: $[M + H]^+$ calculated for $C_{19}H_{32}NO_4$ 280.2271, found = 280.2272, Δ = 0.4 ppm.

5-cyclohexyl-1-(tetrahydro-2H-pyran-4-yl)pyrrolidin-2-one (4t)

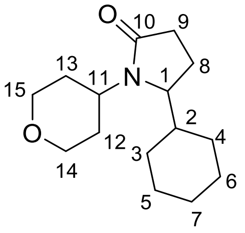

Mr = 251.37

General procedure D was applied to N-(1-cyclohexylpropyl)tetrahydro-2H-pyran-4-amine (67.6 mg, 0.3 mmol) with 2-methyl-6-nitrobenzoic acid (16.3 mg, 0.09 mmol) in place of triisopropylbenzoic acid, and 6.25% CO / Air in place of CO. The crude reaction product was purified by alumina column chromatography (50% to 75% ethyl acetate / petroleum ether) to give **4t** as a yellow oil (30.1 mg, 40%,). IR (λ_{max}/cm^{-1}) 2925, 2852, 1666, 1451, 1421, 1382, 1281, 1145, 1087, 1009. ^{1}H NMR (400.1 MHz, $CDCl_3$) 4.10 – 3.88 (3H, m, H11, H14a, H15a), 3.59 (1H, dt, J1 = 9.1 Hz, J2 = 2.8 Hz, H1), 3.49 – 3.41 (2H, m, H14, H15), 2.42 – 2.20 (2H, m, H9), 2.10 (1H, qd, J1 = 12.1 Hz, J2= 4.9 Hz, H8a), 1.97 – 1.47 (11H, m, H2, H(3 – 7)a, H8b, H12, H13), 1.35 – 0.86 (5H, m, H(3 – 7)b). ^{13}C NMR (100.6 MHz, $CDCl_3$) 175.2 (C10), 67.7 (C14), 67.6 (C15), 62.1 (C1), 50.2 (C11), 41.9 (C2), 31.6 (C12), 31.4 (C9), 29.8 (C8), 29.6 (C13), 26.6 (C3), 26.5 (C4), 25.9 (C5), 25.0 (C6), 19.7 (C7). HRMS (FTMS +p NSI) m/z: $[M + H]^+$ calculated for $C_{15}H_{26}NO_2$ 252.1958, found = 252.1961, Δ = 1.2 ppm.

Trans-4-cyclohexyl-3-methyl-1-(tetrahydro-2H-pyran-4-yl)azetidin-2-one (5t)

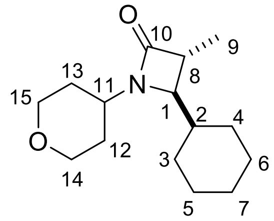

Mr = 251.37

General procedure D was applied to N-(1-cyclohexylpropyl)tetrahydro-2H-pyran-4-amine (67.6 mg, 0.3 mmol). The crude reaction product was purified by alumina column chromatography (50% to 75% ethyl acetate / petroleum ether) to give **5t** as a yellow oil (19.6 mg, 26%,). IR (λ_{max}/cm^{-1}) 2923, 2851, 1734, 1449, 1376, 1358, 1142, 1088, 1008. ^1H NMR (400.1 MHz, $CDCl_3$) 4.01 – 3.93 (2H, m, H14, H15), 3.63 (1H, tt, J1 = 10.8 Hz, J2 = 4.1 Hz, H1), 3.45 – 3.34 (2H, m, H14, H15), 3.12 (1H, dd, J1 = 4.7 Hz, J2 = 2.1 Hz, H1), 2.83 (1H, qd, J1 = 7.4 Hz, J2 = 2.3 Hz, H8), 1.99 (1H, qd, J1 = 12.1 Hz, J2 = 4.5 Hz, H2), 1.84 – 1.58 (9H, m, H3a – H7a, H12, H13), 1.22 (3H, d, J = 7.7 Hz, H9), 1.33 – 1.09 (3H, m, H3b, H4b, H7b), 1.06 – 0.92 (2H, m, H5b, H6b). ^{13}C NMR (100.6 MHz, $CDCl_3$) 171.1 (C10), 67.0 (C14), 66.9 (C15), 64.3 (C1), 49.4 (C11), 44.9 (C8), 39.9 (C2), 31.8 (C12), 30.6 (C13), 30.0 (C7), 26.6 (C3), 26.4 (C4), 26.2 (C5), 25.8 (C6), 13.8 (C9). HRMS (FTMS +p NSI) m/z: $[M + H]^+$ calculated for $C_{15}H_{26}NO_2$ 252.1958, found = 252.1959, Δ = 0.4 ppm.

Trans-1-hexyl-5-isopropyl-4-methylpyrrolidin-2-one (4u)

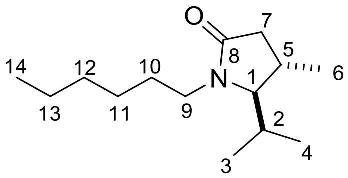

Mr = 225.38

General procedure D was applied to N-(2,4-dimethylpentan-3-yl)hexan-1-amine (59.8 mg, 0.3 mmol) with 2-methyl-6-nitrobenzoic acid (16.3 mg, 0.09 mmol) in place of triisopropylbenzoic acid, and 6.25% CO / Air in place of CO. The crude reaction product was purified by alumina column chromatography (0% to 20% ethyl acetate / petroleum ether) to give **4u** as a yellow oil (37.2 mg, 55%, *d.r.* = 9:1). IR (λ_{max}/cm^{-1}) 2958, 2927, 2871, 1683, 1441, 1423, 1259, 1066. ^1H NMR (400.1 MHz, $CDCl_3$) 3.68 (1H, ddd, J1 = 15.0 Hz, J2 = 9.1 Hz, J3 = 7.2Hz, H9a), 3.07 (1H, t, J = 3.0 Hz, H1), 2.78 (1H, ddd, J1 = 13.9 Hz, J2 = 8.7 Hz, J3 = 5.0 Hz, H9b), 2.57 (1H, dd, J1 = 17.2 Hz, J2 = 9.2 Hz, H7a), 2.19 – 2.09 (1H, m, H5), 2.09 – 1.99 (1H, m, H2), 1,93 (1H, dd, J1 = 17.2 Hz, J2 = 3.0 Hz, H7b), 1.60 – 1.38 (2H, m, H10), 1.34 – 1.22 (6H, m, H11 – H12), 1.08 (3H, d, J = 7.5 Hz, H6), 0.96 (3H, d, J = 7.5 Hz, H3), 0.88 (3H, t, J = 6.5 Hz, H14), 0.76 (3H, d, J = 6.5 Hz, H4). ^{13}C NMR (100.6 MHz, $CDCl_3$) 174.2 (C8), 70.4 (C1), 40.2 (C9), 39.4 (C7), 31.5 (C10), 28.7 (C2), 26.9 (C11), 26.6 (C12), 25.3 (C5), 23.0 (C13), 22.6 (C14), 18.5 (C6), 15.1 (C3), 14.0 (C4). HRMS (FTMS +p NSI) m/z: $[M + H]^+$ calculated for $C_{14}H_{28}NO$ 226.2165, found = 226.2165, exact match.

5-cyclopentyl-1-(tetrahydro-2H-pyran-4-yl)pyrrolidin-2-one (4v)

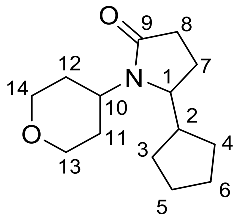

Mr = 237.34

General procedure D was applied to N-(1-cyclopentylpropyl)tetrahydro-2H-pyran-4-amine (63.4 mg, 0.3 mmol) with 2-methyl-6-nitrobenzoic acid (16.3 mg, 0.09 mmol) in place of triisopropylbenzoic acid, and 6.25% CO / Air in place of CO. The crude reaction product was purified by alumina column chromatography (60% to 80% ethyl acetate / petroleum ether) to give **4v** as a yellow oil (44.0 mg, 62%). IR (λ_{max}/cm^{-1}) 2953, 2868, 1666, 1448, 1421, 1144, 1087, 1008. ^{1}H NMR (400.1 MHz, $CDCl_3$) 4.78 – 3.80 (4H, m, H1, H10, H13, H14), 3.43 (2H, td, J1 = 11.9 Hz, J2 = 1.8 Hz, H13, H14), 2.50 – 2.24 (3H, m, H7a, H11a, H12a), 2.18 (1H, tq, J1 = 12.5 Hz, J2 = 4.8 Hz, H8a), 2.06 – 1.86 (2H, H2, H8b), 1.87 – 1.52 (9H, m, H3, H4, H5a, H6a, H7b, H11b, H12b), 1.27 – 1.13 (2H, m, H5b, H6b). ^{13}C NMR (100.6 MHz, $CDCl_3$) 175.1 (C9), 67.8 (C13), 67.7 (C14), 59.7 (C1), 50.4 (C10), 43.5 (C2), 31.4 (C11), 31.3 (C8), 31.0 (C12), 30.2 (C7), 25.9 (C3, C4), 25.8 (C5), 19.7 (C6). HRMS (FTMS +p NSI) m/z: $[M + H]^+$ calculated for $C_{15}H_{25}NO_2$ 238.1802, found = 238.1804, Δ = 0.8 ppm.

1.5 Functionalisation of Lactams

***Trans-1-(cyclohexylmethyl)-2-isopropyl-3-methylpyrrolidine* (7)**

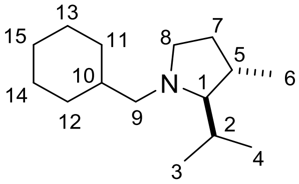

Mr = 223.40

To an ice-cold solution of trans-1-(cyclohexylmethyl)-5-isopropyl-4-methylpyrrolidin-2-one (0.2 g, 0.8 mmol) in dry diethylether (15 mL) was added lithium aluminium hydride (128 mg, 3.4 mmol) and the reaction mixture was stirred for 48 h under an nitrogen atmosphere at room temperature. The reaction was diluted with diethylether (50 mL) and quenched with dropwise addition saturated aqueous sodium hydrogen carbonate at 0 °C. The aqueous phase was extracted with diethylether (2 x 50 mL), and the combined organic layers washed with brine (50 mL), dried over magnesium sulphate, filtered, and concentrated *in vacuo*. The crude reaction product was purified by alumina column chromatography (0% to 10% ethyl acetate / petroleum ether) to give **7** as a yellow oil (155 mg, 82%). IR (λ_{max}/cm^{-1}) 2956, 2916, 2785, 1448. ^{1}H NMR (400.1 MHz, $CDCl_3$) 3.05 – 2.95 (1H, m, H9a), 2.42 – 2.30 (1H, m, H8a), 2.13 (1H, ddd, *J1* = 11.0 Hz, J2 = 8.7 Hz, J3 = 6.1 Hz, H9b), 2.03 (1H, dd, *J1* = 11.9 Hz, J2 = 4.1 Hz, H8b), 2.02 – 1.58 (8H, m, H5, H7a, H10a – H15a), 1.48 – 1.33 (1H, m, H10), 1.30 – 1.07 (4H, m, H7b, H11b, H12b, H15b), 0.93 (3H, d, *J* = 6.8 Hz, H6), 0.88 (3H, d, *J* = 6.8 Hz, H3), 0.84 (3H, d, *J* = 6.5 Hz, H4), 1.00 – 0.70 (2H, m, H13b, H14b). ^{13}C NMR (100.6 MHz, $CDCl_3$) 78.5 (C1), 63.3 (C8), 52.9 (C9), 37.5 (C10), 32.9 (C7), 32.4 (C5), 32.4 (C11), 31.8 (C12), 29.9 (C2), 27.2 (C15), 26.5 (C13), 26.3 (C14), 22.9 (C6), 20.0 (C3),

16.7 (C4). HRMS (ESI) m/z: calculated for $C_{15}H_{30}N$ $[M+H]^+$ 224.2373, found 224.2374, Δ = 0.4 ppm.

1-(cyclohexylmethyl)-5-isopropyl-4-methyl-3-propylpyrrolidin-2-one (8)

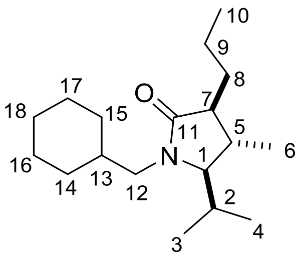

Mr = 279.47

Trans-1-(cyclohexylmethyl)-5-isopropyl-4-methylpyrrolidin-2-one (71.1 mg, 0.3 mmol) was dissolved in tetrahydrofuran (6 mL) and cooled to - 78 °C. LDA (2.0 M in tetrahydorfuran / heptane / ethylbenzene, 0.16 mL, 1.1 eq) was added dropwise and stirred for 10 mins. 1-Iodopropane (56.1 mg 1.1 eq) was then added to the solution and stirred for another 1 hour, warming up to room temperature. The mixture was then quenched with saturated ammonium chloride (20 mL), extracted with dichloromethane (3 x 20 mL), washed with brine and dried with magnesium sulphate. The solvent was then removed *in vacuo*. The crude reaction product was purified by alumina column chromatography (5% ethyl acetate / petroleum ether) to give **8** as a yellow oil (68.7 mg, 82%, *d.r.* = 5 : 1). IR (λ_{max}/cm^{-1}) 2957, 2923, 2852, 1677, 1443, 1257, 1188. ^{1}H NMR (400.1 MHz, $CDCl_3$) 3.59 (1H, dd, J1 = 14.2 Hz, J2 = 9.4 Hz, H12a), 3.05 (1H, dd, J1 = 5.8 Hz, J2 = 3.9 Hz, H1), 2.54 (1H, dd, J1 = 14.1 Hz, J2 = 9.4 Hz), 2.13 – 2.03 (1H, m, H2), 2.01 – 1.94 (1H, m, H7), 1.84 – 1.27 (9H, m, H5, H8, H(13-18)a), 1.21 – 1.09 (6H, m, H10, H14b, H15b, H18b) 0.99 – 0.87 (10H, m, H3, H6, H9, H16b, H17b), 0.71 (3H, d, J = 5.4 Hz, H4). ^{13}C NMR (100.6 MHz, $CDCl_3$) 176.7 (C11), 68.0 (C1), 49.6 (C7), 45.7 (C12), 35.0 (C13), 33.7 (C18), 31.8 (C5), 31.2 (C14), 30.4 (C15), 27.4 (C2), 26.4 (C8), 25.9 (C16), 25.7 (C17), 22.9 (C10), 20.4 (C9), 18.4 (C6), 15.1 (C4), 14.2 (C3). HRMS (FTMS +p NSI) m/z: $[M + H]^+$ calculated for $C_{18}H_{34}NO$ 280.2635, found = 280.2633, Δ = -0.7 ppm.

Trans-1-(cyclohexylmethyl)-3,3-difluoro-5-isopropyl-4-methylpyrrolidin-2-one (9)

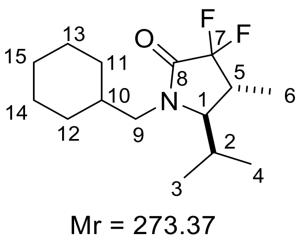

Mr = 273.37

Trans-1-(cyclohexylmethyl)-5-isopropyl-4-methylpyrrolidin-2-one (71.1 mg, 0.3 mmol) was dissolved in tetrahydrofuran (6 mL) and cooled to - 78 °C. LDA (2.0 M in THF / heptane/ ethylbenzene, 0.32 mL, 2.2 eq) was added dropwise and stirred for 10 mins. NFSI (208 mg 2.2 eq) was then added to the solution and stirred for another 1 hour, warming up to room temperature. The mixture was then quenched with saturated ammonium chloride (20 mL), extracted with dichloromethane (3 x 20 mL), washed with brine and dried with magnesium sulphate. The solvent was then removed *in vacuo*. The crude reaction product was purified by alumina column chromatography (5% ethyl

acetate / petroleum ether) to give **9** as a yellow oil (67.3 mg, 82%). IR (λ_{max}/cm^{-1}) 2925, 2851, 1719, 1450, 1394, 1331, 1249, 1180, 1107, 1045. ^{1}H NMR (400.1 MHz, $CDCl_3$) 3.66 (1H, dd, J1 = 13.5 Hz, J2 = 9.5 Hz, H9a), 3.16 (1H, q, J = 4.0 Hz, H1), 2.69 (1H, ddd, J1 = 14.0 Hz, J2 = 5.2 Hz, J3 = 2.2 Hz, H9b), 2.40 – 2.25 (1H, m, H5), 2.23 – 2.12 (1H, m, H2), 1.78 – 1.51 (6H, m, H(10-15)a), 1.21 (3H, dd, J1 = 7.4 Hz, J2 = 2.8 Hz, H6), 1.28 – 1.13 (3H, m, H11b, H12b, H15b), 1.02 (3H, d, J = 7.0 Hz, H3), 1.10 – 0.88 (2H, m, H13, H14), 0.78 (3H, d, J = 7.0 Hz, H4). ^{13}C NMR (100.6 MHz, $CDCl_3$) 164.1 (C8), 118.3 (C7), 65.2 (C1), 46.3 (C9), 34.9 (C10), 34.5 (C5), 31.0 (C11), 30.1 (C13), 27.3 (C2), 26.2 (C14), 25.7 (C12), 25.5 (C15), 18.8 (C3), 14.5 (C4), 13.5 (C6). ^{19}F NMR (376.5 MHz, $CDCl_3$) -103.5 (d), -115.4 (d). HRMS (FTMS +p NSI) m/z: $[M + H]^+$ calculated for $C_{15}H_{26}F_2NO$ 274.1977, found = 274.1977, exact match.

1.6 Palladacycle studies

N-(2,4-dimethylpentan-3-yl)-4,4-difluorocyclohexan-1-amine-palladacycle-trimer (1f-int-I)

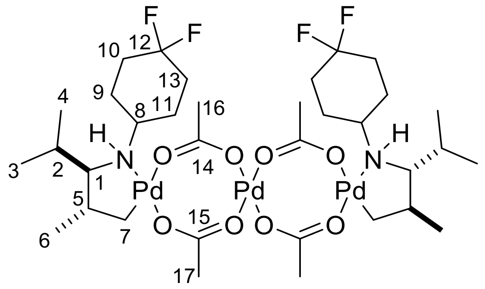

Mr = 1020.11

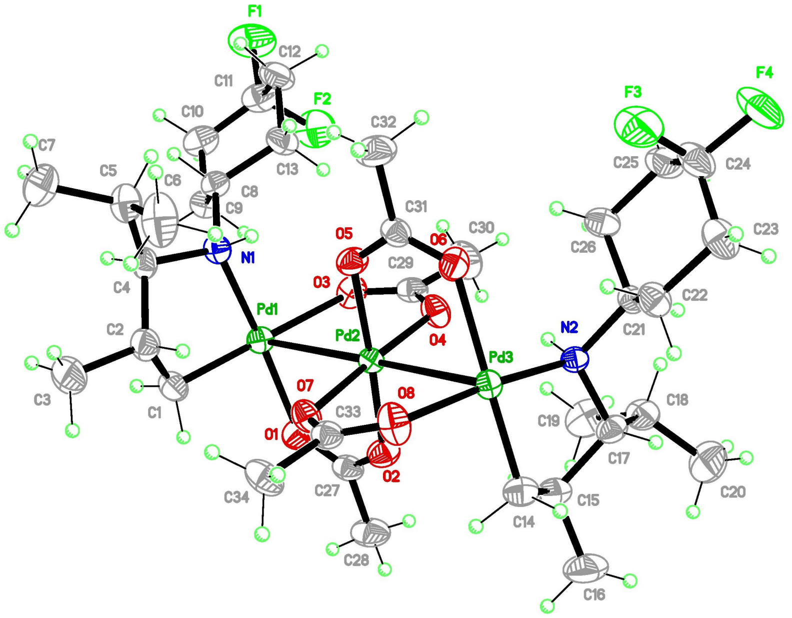

N-(2,4-dimethylpentan-3-yl)-4,4-difluorocyclohexan-1-amine (70.0 mg, 1 mmol) and palladium acetate (331 mg, 1.5 mmol, 1.5 eq) were dissolved in toluene (6 mL) and heated to 60 °C for 16 hours, The mixture was cooled, filtered through celite (eluting with ethyl acetate, 20 mL), concentrated *in vacuo*. The crude product was purified by recrystalisation in dichloromethane / Hexane mixture to give a **1f-int-I** as a green solid (72%, 240 mg). Crystals were grown by diffusion of hexane in a dichloromethane solution of the palladacycle. IR (λ_{max}/cm^{-1}) 2967, 1586, 1537, 1396, 1336, 1104, 975. ^{1}H NMR (400.1 MHz, $CDCl_3$) 4.46 (1H, d, J = 10.0 Hz, H7a), 3.22 – 3.04 (1H, m, H7b), 2.63 – 2.23 (6H, m, H5, H8, H9a – H12a), 2.12 – 1.90 (5H, m, H2, H9b – H12b), 1.86 (3H, s, H16), 1.72 (3H, s, H17), 1.03 (3H, d, J = 7.5 Hz, H3), 0.92 (3H, d, J = 7.5 Hz, H4), 0.88 (3H, t, J = 7.0 Hz, H6). ^{13}C NMR (100.6 MHz, $CDCl_3$) 184.11 (C14), 180.3 (C15), 76.7 (C1), 59.5 (C8), 39.3 (C5), 32.7 (C13), 31.6 (C2), 30.5 (C7), 23.3 (C9), 23.0 (C10), 22.7 (C11, C12), 20.7 (C6), 18.2 (C3), 16.6 (C4). ^{19}F NMR (376.5 MHz, $CDCl_3$) -94.5 (d, J = 236.9 Hz), -102.7 (d, J = 236.4 Hz). HRMS (FTMS +p NSI) m/z: $[M + H]^+$ calculated for $C_{34}H_{61}F_4N_2O_8Pd_3$ 1020.1420, found = 1020.1406 , Δ = -1.3 ppm.

(Cyclohexylmethyl)(2,4-dimethylpentan-3-yl)carbamic chloride (6)

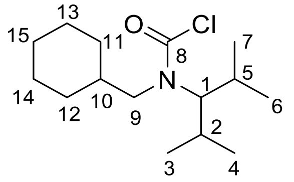

Mr = 273.85

To a slurry of sodium bicarbonate (250 mg, 3 mmol) and triphosgene (300 mg, 1 mmol) in anhydrous dichloromethane at - 10 °C was added a solution of N-(cyclohexylmethyl)-2,4-dimethylpentan-3-amine (211 mg, 1 mmol) in DCM dropwise under nitrogen. The mixture was allowed to warm up to room temperature overnight, then filtered and the solvent removed *in vacuo*. The product **6** was obtained without further purification as a white liquid (220 mg, 80 %). IR (λ_{max}/cm^{-1}) 2965, 2852, 2924, 1729, 1448, 1387, 1298, 1250, 1220, 1127, 1103. ^{1}H NMR (400.1 MHz, $CDCl_3$) 3.91 (1H, t, J = 6.3 Hz, H1), 3.26 (1H, d, J = 7.0 Hz, H8a), 3.07 (1H, d J = 7.0 Hz, H8b), 2.37 – 2.20 (1H, m, H2), 2.00 (1H, oct, J = 7.0 Hz, H3), 1.81 – 1.53 (6H, m, H(9-14)a), 1.31 – 1.03 (5H, m, H10 - 14)b), 1.00 (d, 6H, J = 6.9 Hz, H4, H5), 0.97 (d, 6H, J = 6.9 Hz, H6, H7). ^{13}C NMR (100.6 MHz, $CDCl_3$) 151.6 (C15), 71.8 (C1), 53.7 (C8), 37.9 (C9), 31.7 (C2), 31.4 (C3), 29.2, 26.2, 26.1, 26.0 (C10 – C14), 20.1 (C4, C5), 19.5 (C6, C7). HRMS (FTMS +p NSI) m/z: [M + H]$^+$ calculated for $C_{15}H_{29}NClO$ 274.1938, found = 274.1937, Δ = - 0.4 ppm.

2 SELECTED OPTIMISATION STUDIES

2.1 Acid Screening (2)

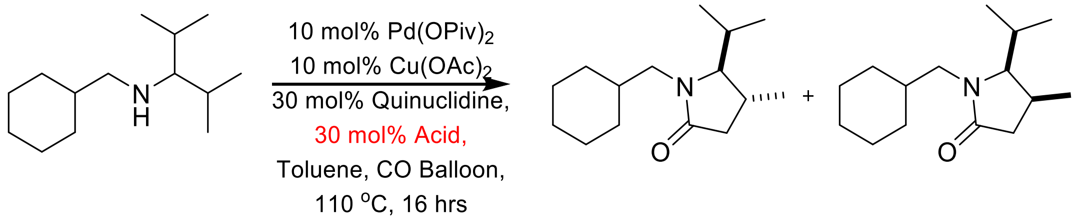

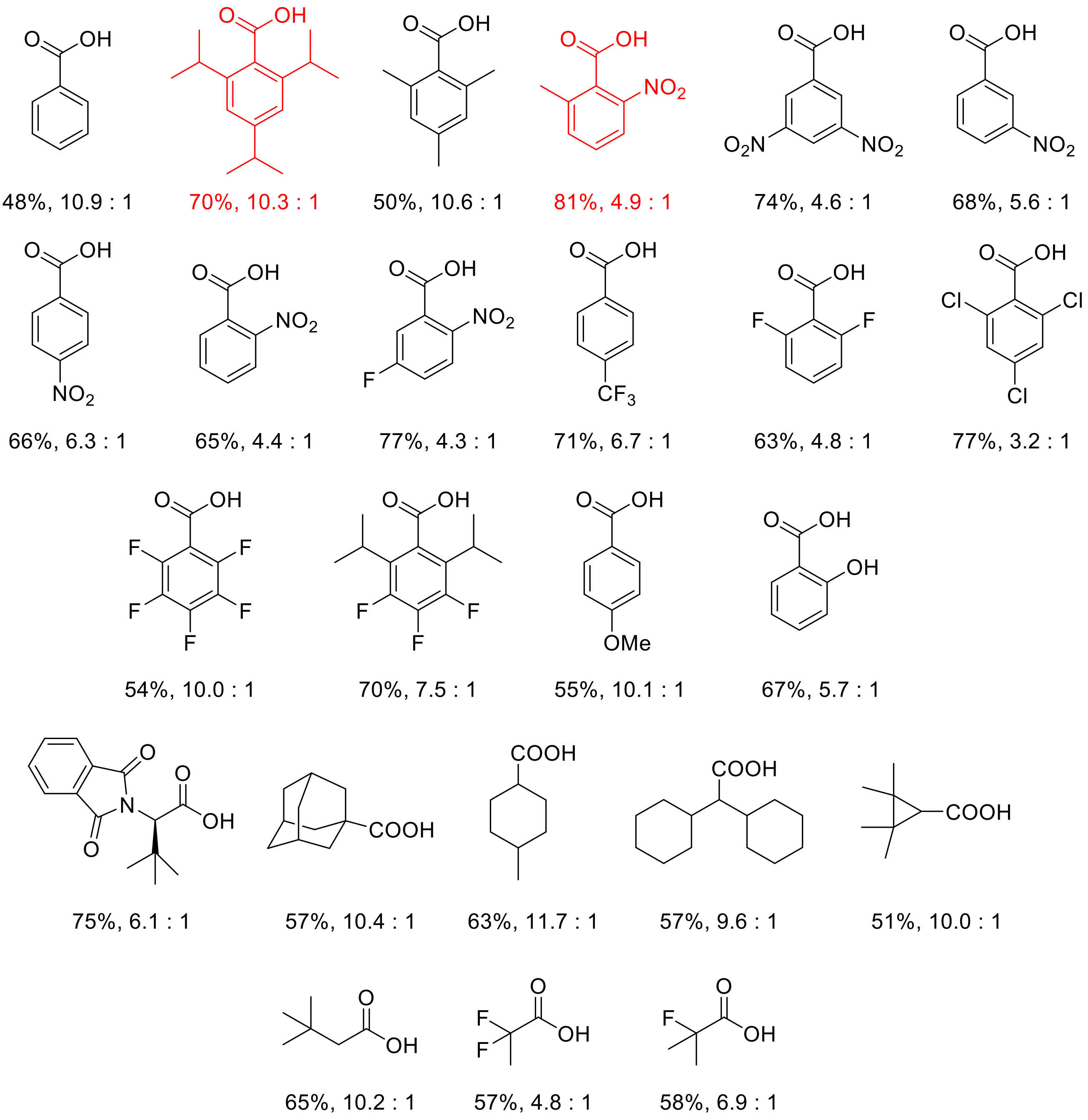

2.2 Ligand Screening (3)

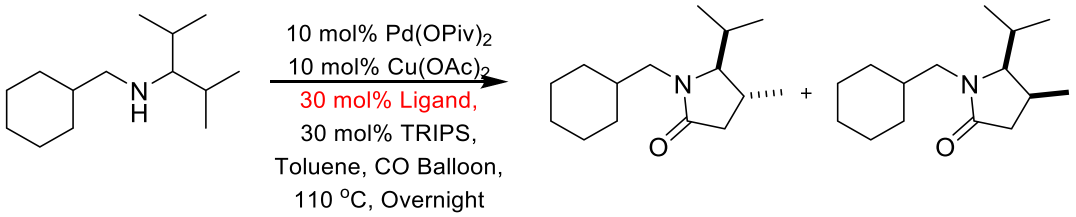

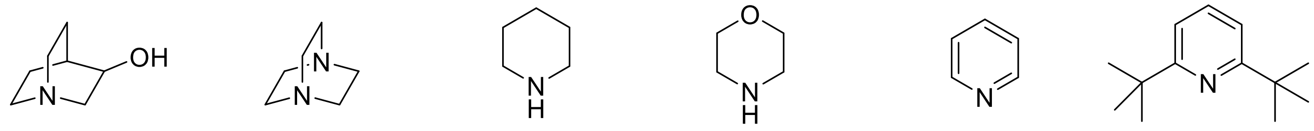

62%, 10.0 : 1 | 23%, 14.5 : 1 | 62%, 13.0 : 1 | 22%, 13.0 : 1 | 46%, 18.0 : 1 | 47%, 11.7 : 1

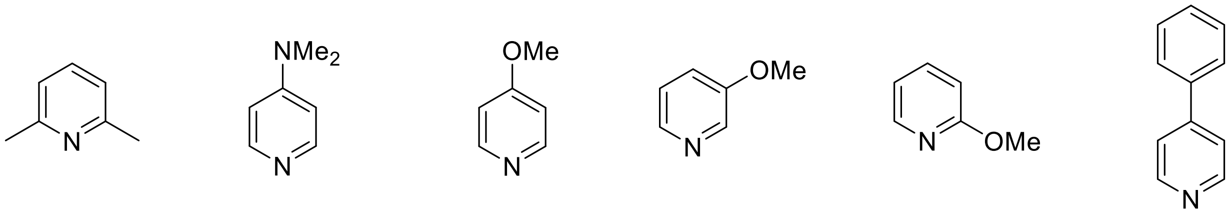

50%, 8.1 : 1 | 62%, 19.6 : 1 | 65%, 23.5 : 1 | 56%, 16.1 : 1 | 46%, 11.0 : 1 | 57%, 16.1 : 1

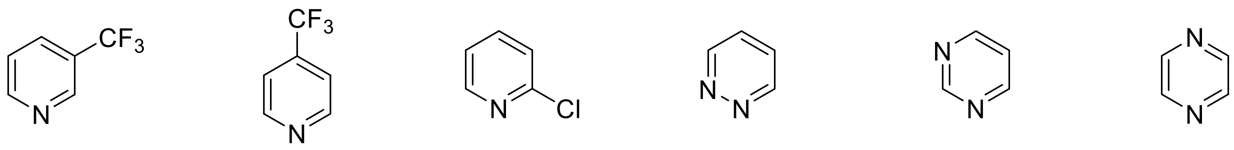

39%, 11.6 : 1 | 54%, 10.5 : 1 | 49%, 3.3 : 1 | 43%, 16.0 : 1 | 37%, 16.0 : 1 | 35%, 15.0 : 1

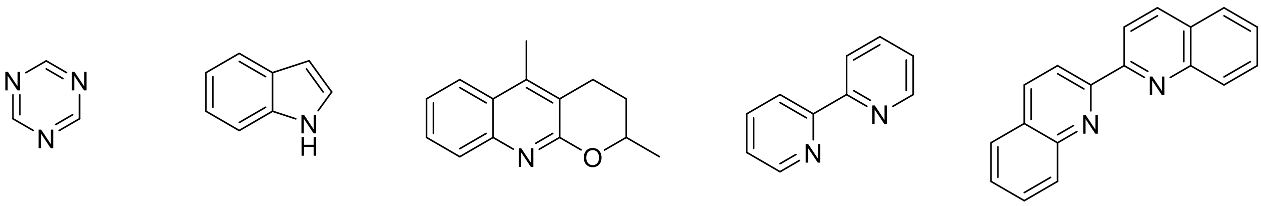

36%, 13.0 : 1 | 35%, 12.0 : 1 | 43%, 7.3 : 1 | 14%, 4.9 : 1 | 46%, 10.1 : 1

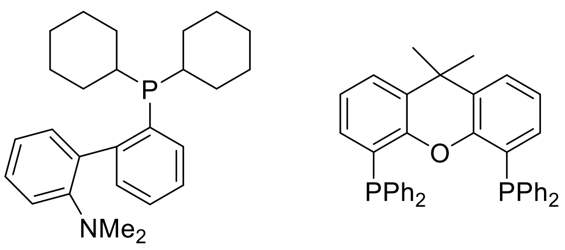

51%, 10.5 : 1 | 51%, 9.2 : 1

2.3 Ligand and acid loading screening

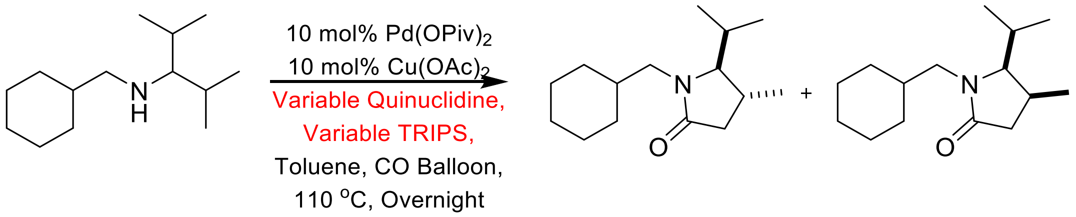

Quinuclidine	TRIPS	Yield	d.r.
**10**	**30**	67.1	9.9
**30**	**30**	70.0	10.1
**50**	**30**	67.6	9.4
**100**	**30**	66.7	9.8
**30**	**10**	39.3	16.5
**30**	**30**	70.0	10.1
**30**	**50**	42.1	10.3
**30**	**100**	37.0	6.3
**10**	**10**	34.6	18.5
**30**	**30**	70.0	10.1
**50**	**50**	43.3	10.1
**100**	**100**	42.1	7.2

2.4 Solvent screening

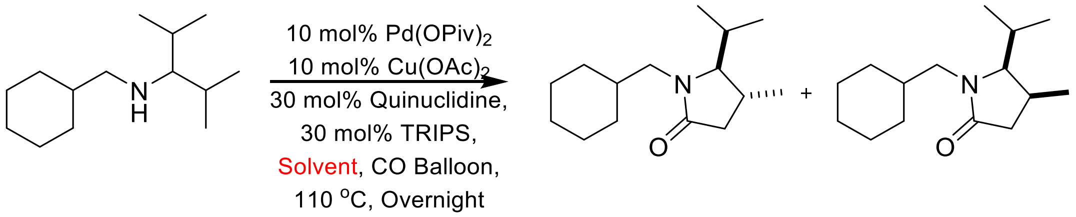

Solvent	Yield	d.r.
***o*-Xylene**	60.8	9.9
**Ethyl Acetate**	-	-
**DCE**	-	-
**Dioxane**	47.1	12.2
**Chlorobenzene**	65.8	7.8
**Ethylene Glycol**	-	-
**THF**	-	-
**DMF**	15.9	11.6

2.5 Oxidant screening

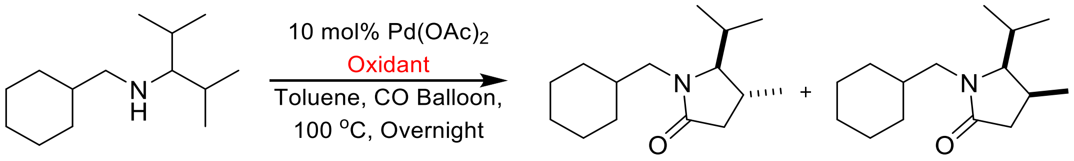

Oxidant	Yield	d.r.
10% $Cu(OAc)_2$	29.1	11.0
10% $Cu(OAc)_2$	25.3	10.8
100% $Cu(OAc)_2$	16.2	12.5
200% AgOAc, 200% BQ	14.1	9.3
100% Ag_2CO_3	26.3	12.3
200% BQ	14.3	9.1
400% TEMPO	23.6	12.3

2.6 Palladium source screening

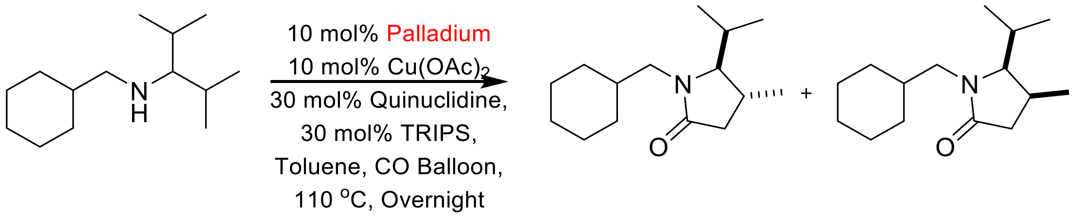

Palladium Source	Yield	d.r.
$Pd(OAc)_2$	48	7.5
$Pd(OBz)_2$	31	6.7
$PdCl_2$	-	-
$Pd(OTFA)_2$	-	-

2.7 Stiochiometric studies

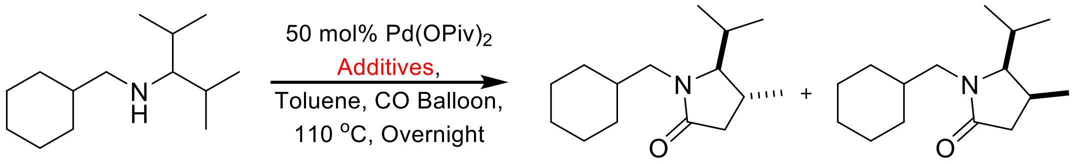

1a **1bi** **1bii**

Quinuclidine	Trips	Yield	d.r.
-	-	27.0	3.9
-	50%	19.9	3.0
50%	-	42.2	1.2
50%	50%	11.4	11.8

2.7 Other control experiments

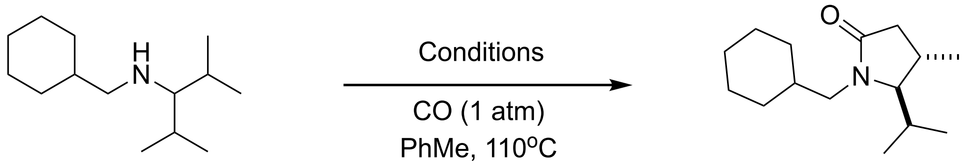

1 2

10 mol% Pd(TRIP-OH)$_2$ + 10 mol% Cu(TRIP-OH)$_2$ = 50% 11:1 d.r.

10 mol% Pd(TRIP-OH)$_2$ + 10 mol% Cu(TRIP-OH)$_2$ + 30 mol% TRIP-OH + 30 mol% quinuclidine = 65% 10:1 d.r

10 mol% Pd(OPiv)$_2$ + 10 mol% Cu(TRIP-OH)$_2$ + 30 mol% TRIP-OH + 30 mol% quinuclidine = 71% 9:1 d.r

10 mol% Pd(TRIP-OH)$_2$ + 10 mol% Cu(OAc)$_2$ + 30 mol% TRIP-OH + 30 mol% quinuclidine = 68% 10:1 d.r.

3.1 References

(1) Kashima, C.; Harada, K.; Fujioka, Y.; Maruyama, T.; Omote, Y. *J. Chem. Soc. Perkin Trans. 1* **1988**, No. 3, 535–539.

(2) Zhdanko, A. G.; Nenajdenko, V. G. *J. Org. Chem.* **2009**, *74* (2), 884–887.

Appendix 1: Proton and Carbon NMRs

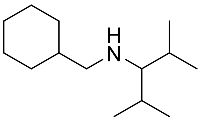

1a

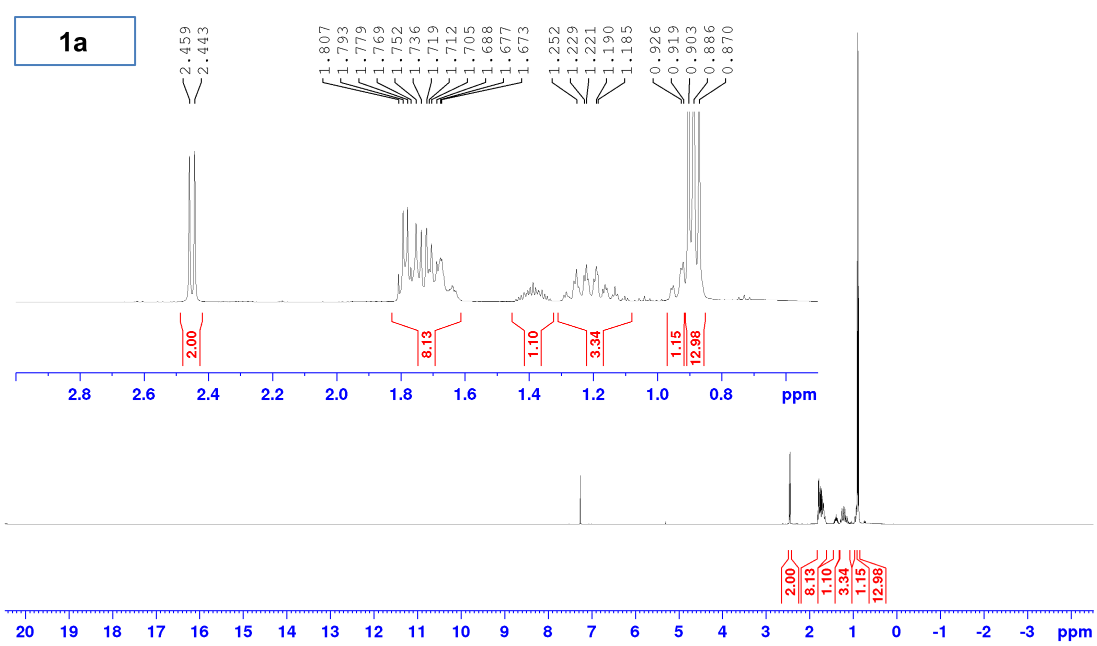

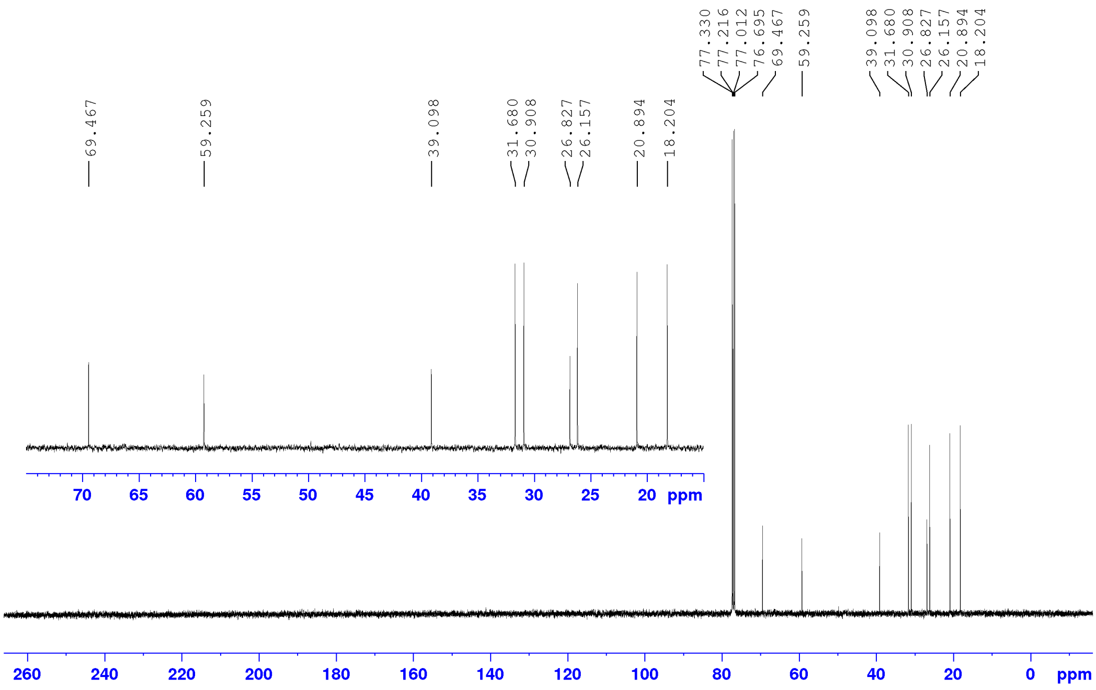

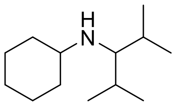

1b

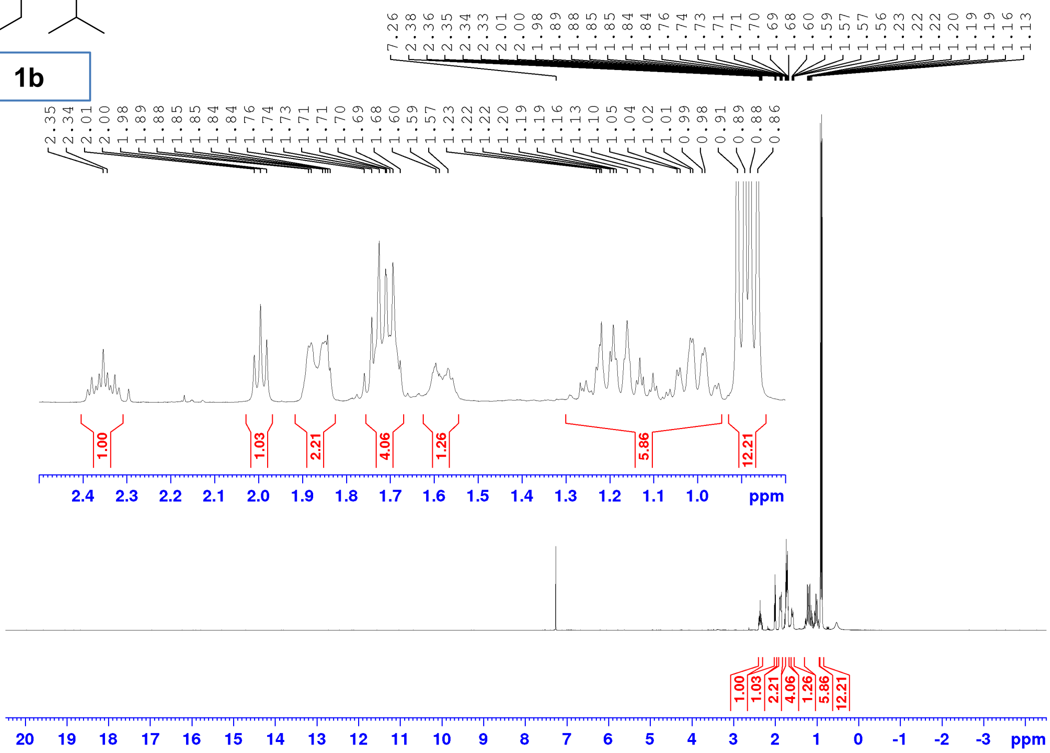

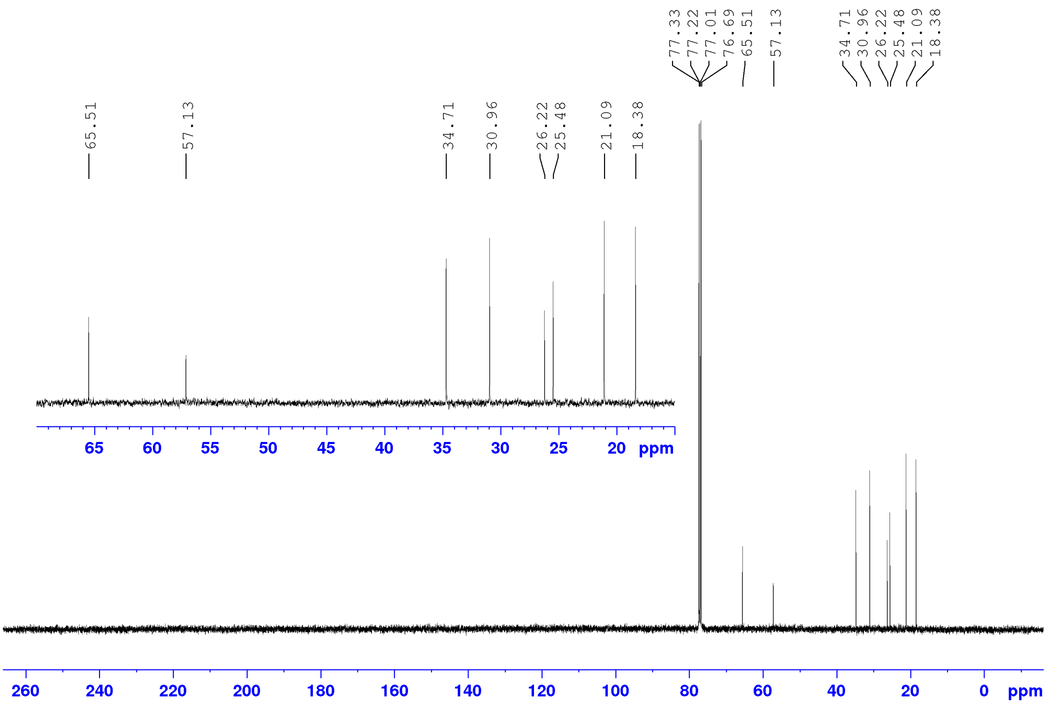

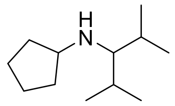

1c

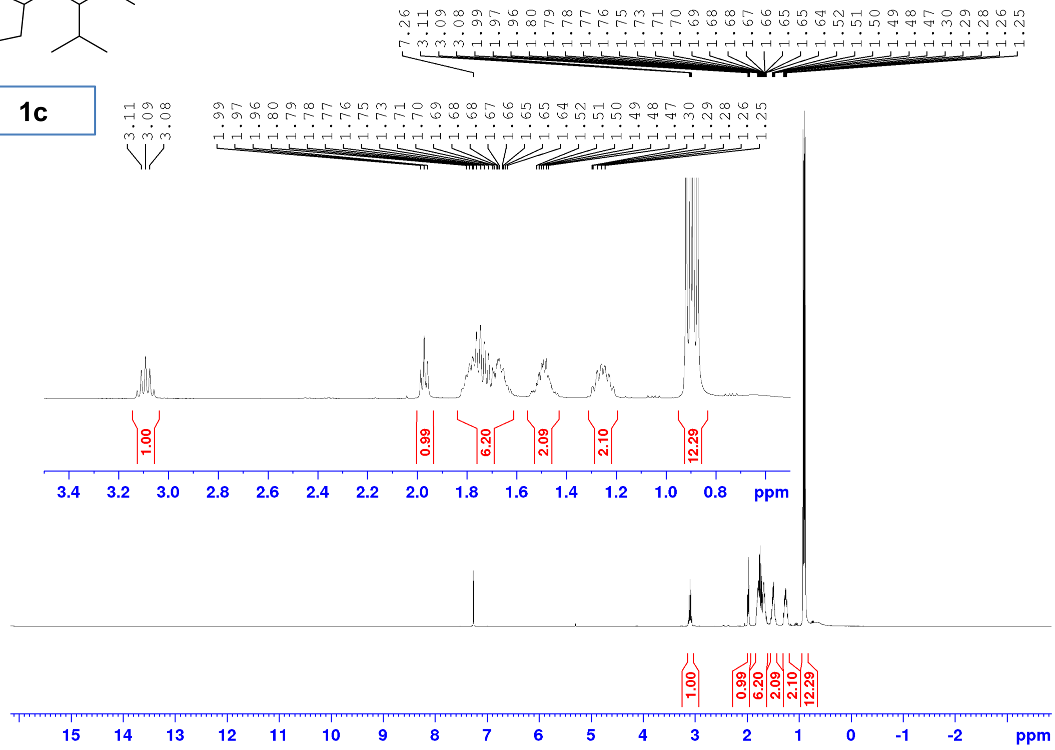
7.26 3.11 3.09 3.08 1.99 1.97 1.96 1.80 1.79 1.78 1.77 1.76 1.75 1.73 1.71 1.70 1.69 1.68 1.68 1.67 1.66 1.65 1.65 1.64 1.52 1.51 1.50 1.49 1.48 1.47 1.30 1.29 1.28 1.26 1.25
3.11 3.09 3.08
1.99 1.97 1.96 1.80 1.79 1.78 1.77 1.76 1.75 1.73 1.71 1.70 1.69 1.68 1.68 1.67 1.66 1.65 1.65 1.64 1.52 1.51 1.50 1.49 1.48 1.47 1.30 1.29 1.28 1.26 1.25
1.00
0.99
6.20
2.09
2.10
12.29
3.4 3.2 3.0 2.8 2.6 2.4 2.2 2.0 1.8 1.6 1.4 1.2 1.0 0.8 ppm
15 14 13 12 11 10 9 8 7 6 5 4 3 2 1 0 -1 -2 ppm

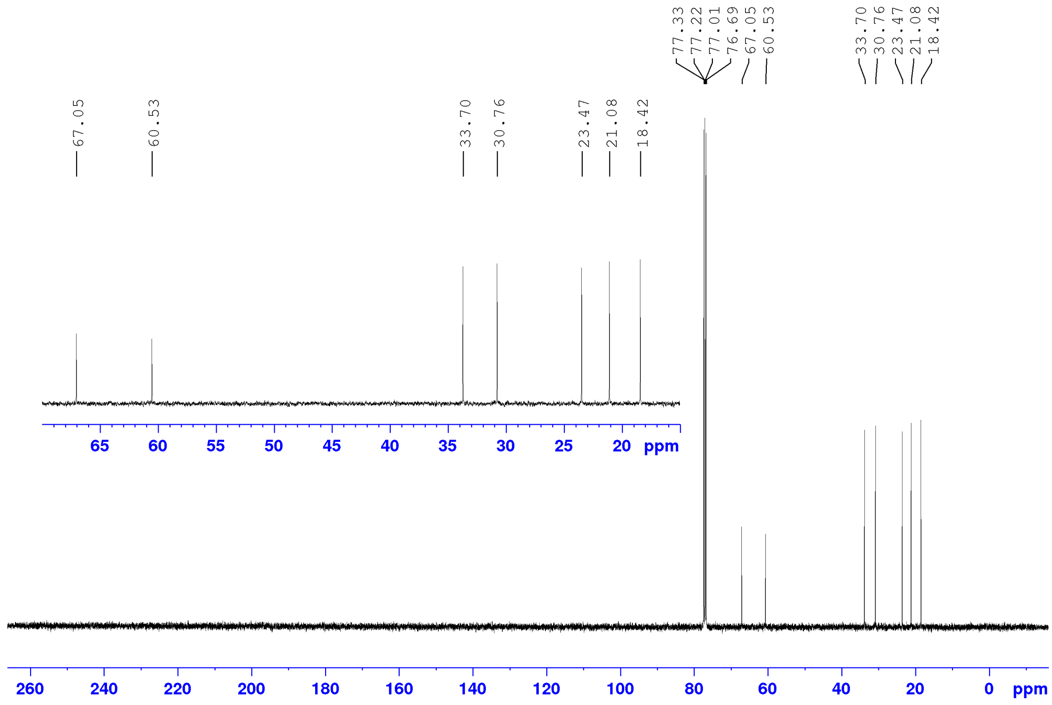
77.33 77.22 77.01 76.69 67.05 60.53
33.70 30.76 23.47 21.08 18.42
67.05
60.53
33.70
30.76
23.47
21.08
18.42
65 60 55 50 45 40 35 30 25 20 ppm
260 240 220 200 180 160 140 120 100 80 60 40 20 0 ppm

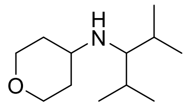

1d

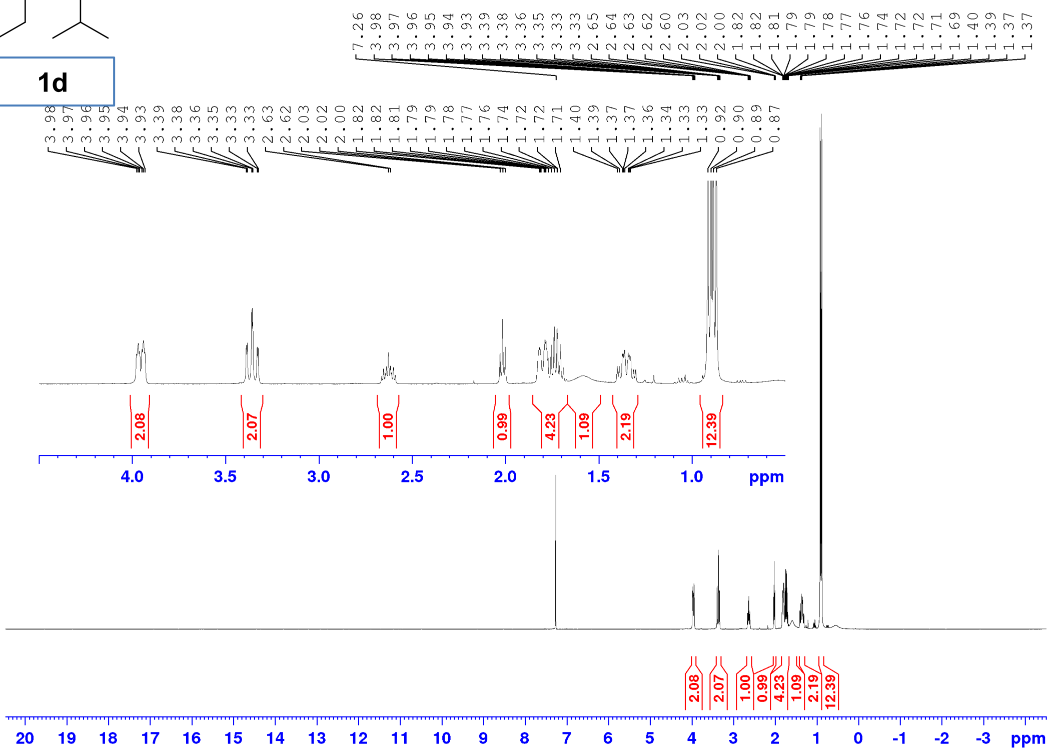
7.26 3.98 3.97 3.96 3.95 3.94 3.93 3.39 3.38 3.36 3.35 3.33 3.33 2.65 2.64 2.63 2.62 2.60 2.03 2.02 2.00 1.82 1.82 1.81 1.79 1.79 1.78 1.77 1.76 1.74 1.72 1.72 1.71 1.69 1.40 1.39 1.37 1.37
2.08 2.07 1.00 0.99 4.23 1.09 2.19 12.39
ppm

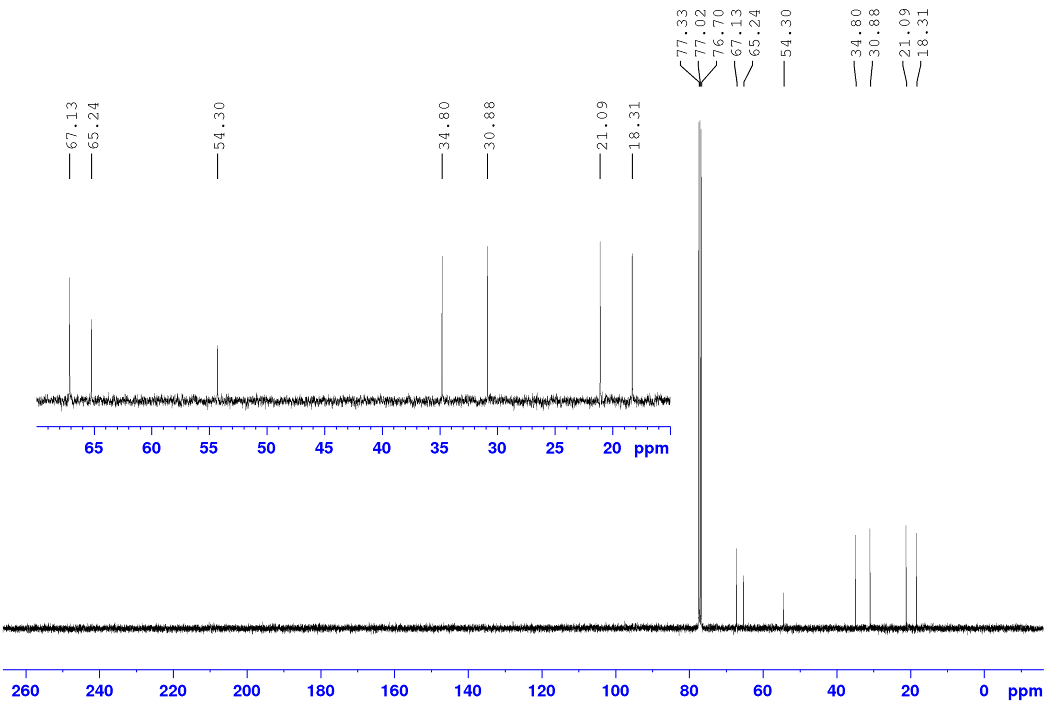
77.33 77.02 76.70 67.13 65.24 54.30 34.80 30.88 21.09 18.31
ppm

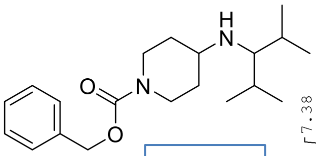
O
N
O
H
N

1e

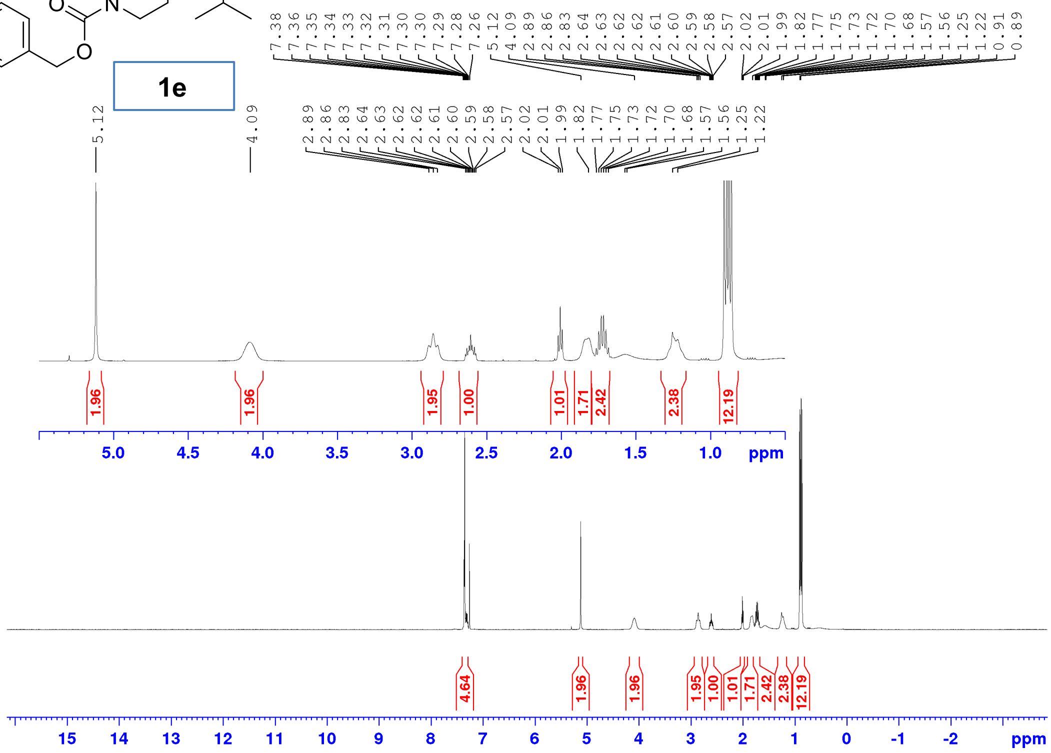
7.38 7.36 7.35 7.34 7.33 7.32 7.31 7.30 7.30 7.29 7.28 7.26 5.12 4.09 2.89 2.86 2.83 2.64 2.63 2.62 2.62 2.61 2.60 2.59 2.58 2.57 2.02 2.01 1.99 1.82 1.77 1.75 1.73 1.72 1.70 1.68 1.57 1.56 1.25 1.22 0.91 0.89
5.12
4.09
2.89 2.86 2.83 2.64 2.63 2.62 2.62 2.61 2.60 2.59 2.58 2.57 2.02 2.01 1.99 1.82 1.77 1.75 1.73 1.72 1.70 1.68 1.57 1.56 1.25 1.22
1.96 1.96 1.95 1.00 1.01 1.71 2.42 2.38 12.19
5.0 4.5 4.0 3.5 3.0 2.5 2.0 1.5 1.0 ppm
4.64 1.96 1.96 1.95 1.00 1.01 1.71 2.42 2.38 12.19
15 14 13 12 11 10 9 8 7 6 5 4 3 2 1 0 -1 -2 ppm

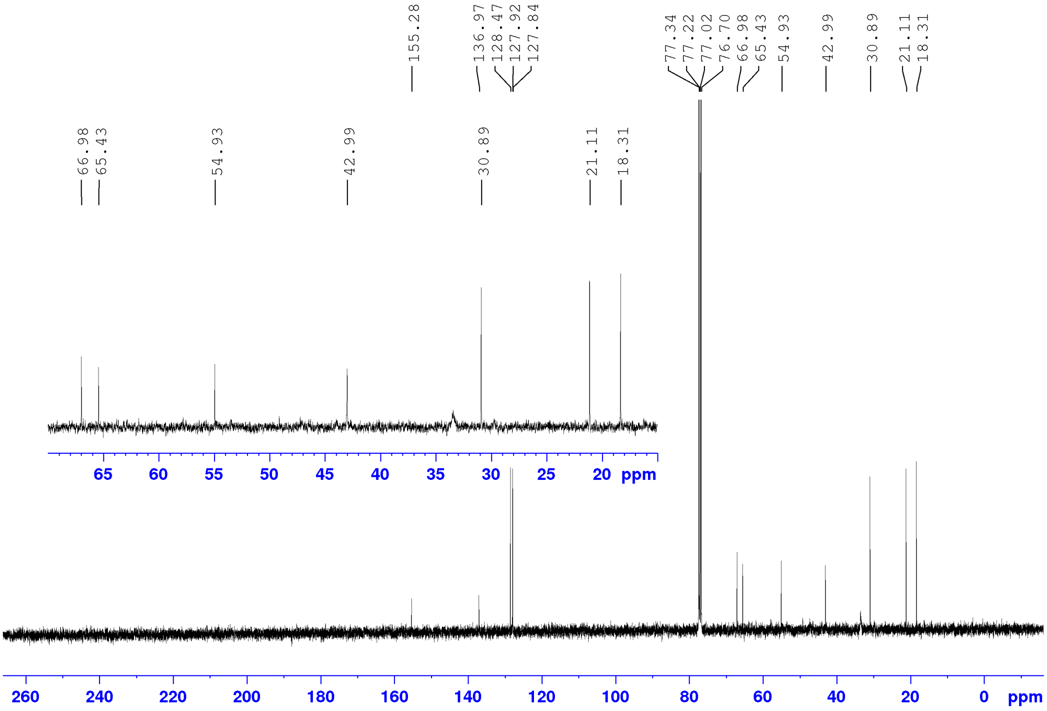
155.28
136.97 128.47 127.92 127.84
77.34 77.22 77.02 76.70 66.98 65.43 54.93 42.99 30.89 21.11 18.31
66.98 65.43
54.93
42.99
30.89
21.11
18.31
65 60 55 50 45 40 35 30 25 20 ppm
260 240 220 200 180 160 140 120 100 80 60 40 20 0 ppm

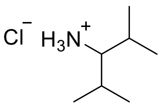

1f-I

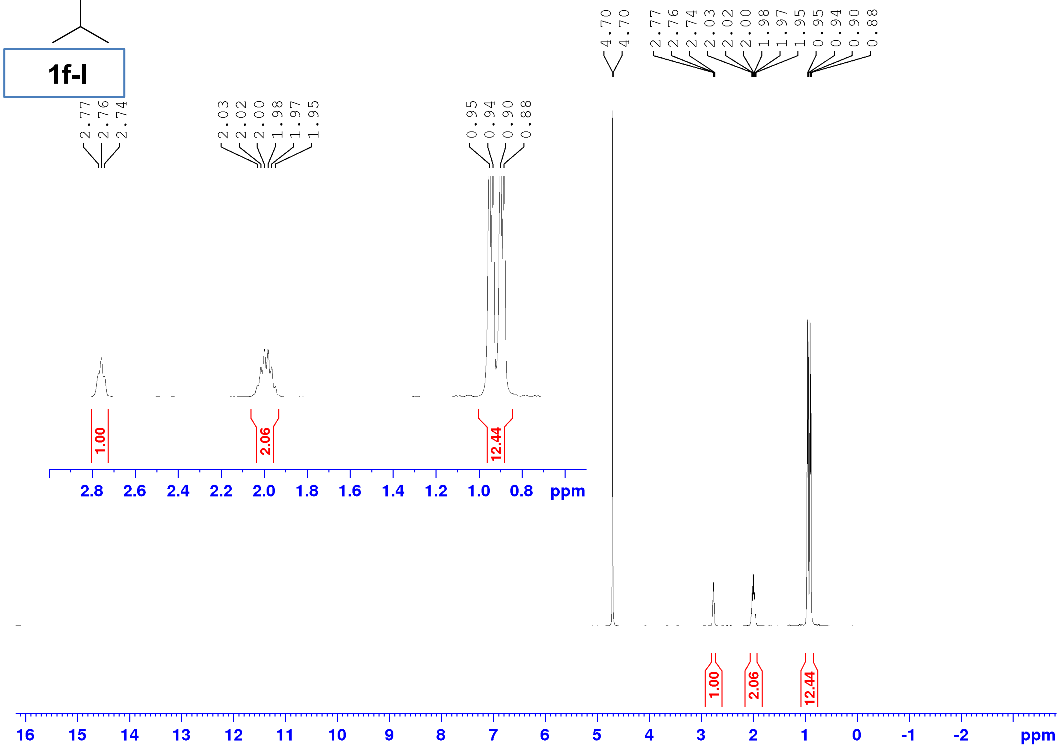

4.70
4.70
2.77
2.76
2.74
2.03
2.02
2.00
1.98
1.97
1.95
0.95
0.94
0.90
0.88
1.00
2.06
12.44
ppm

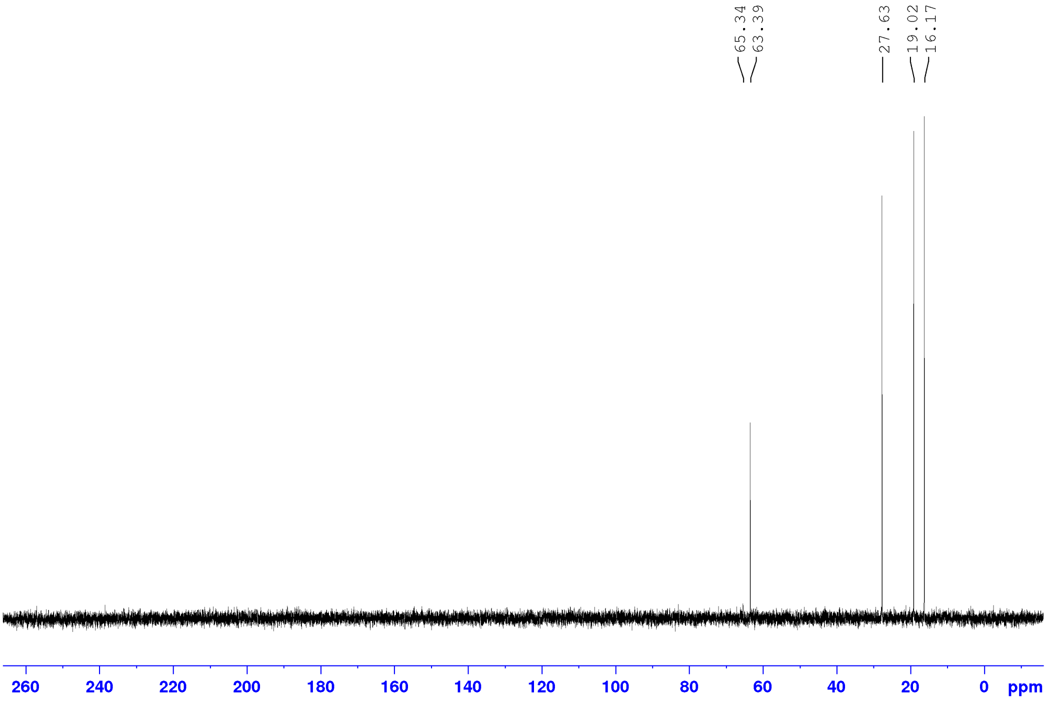

65.34
63.39
27.63
19.02
16.17
ppm

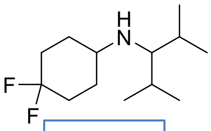

1f

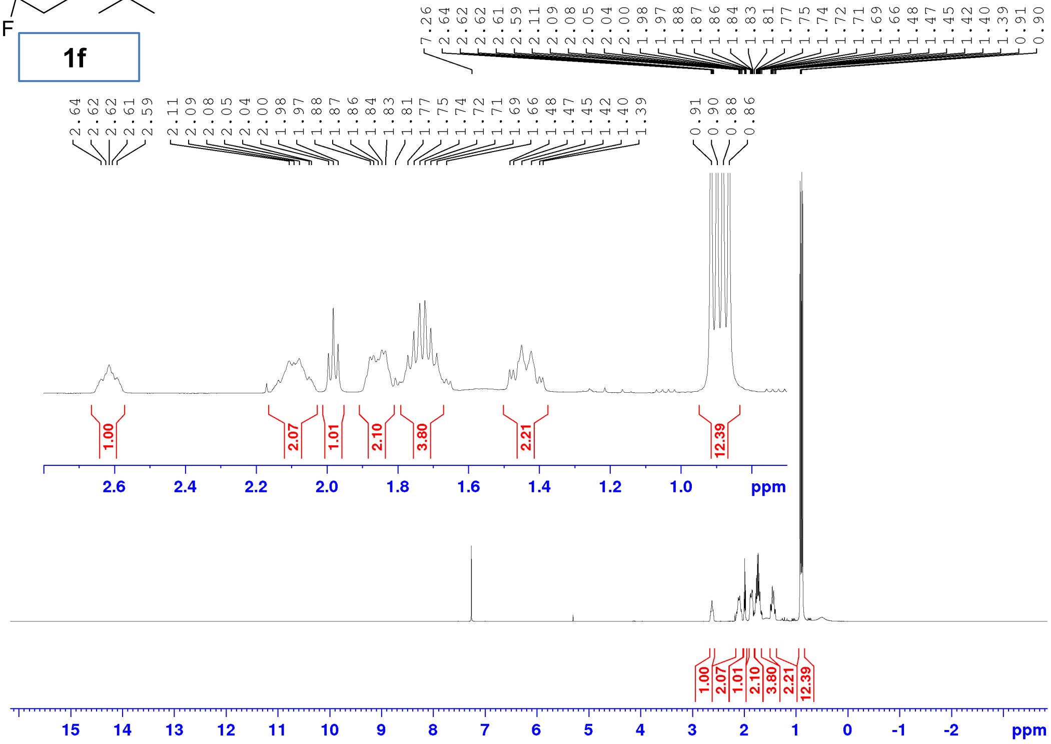

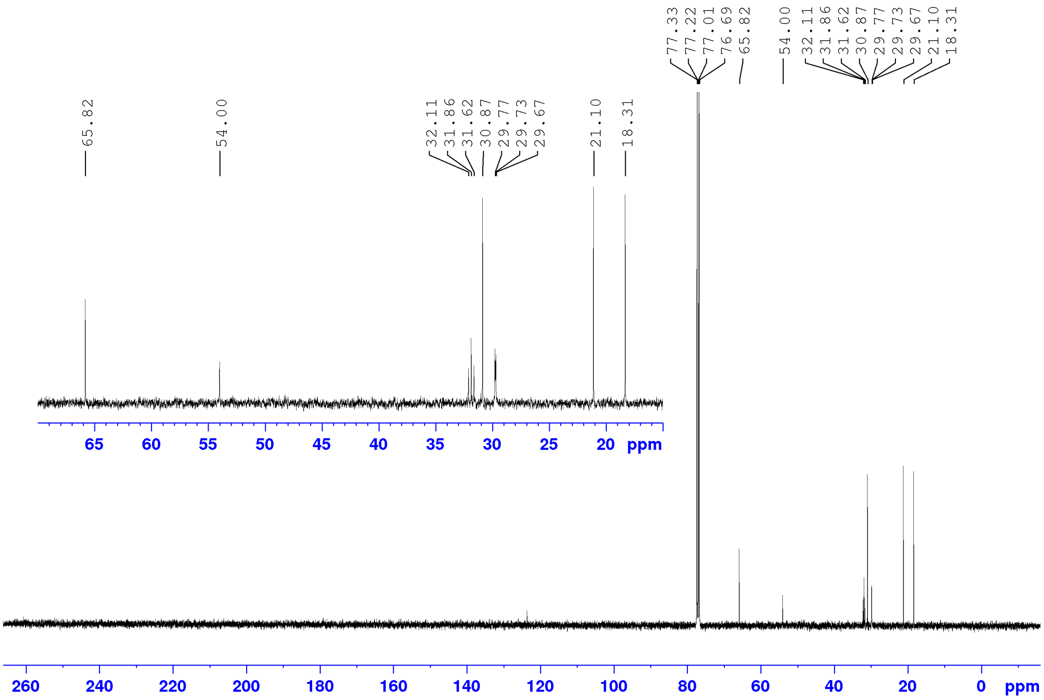

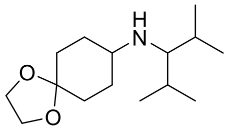

1g

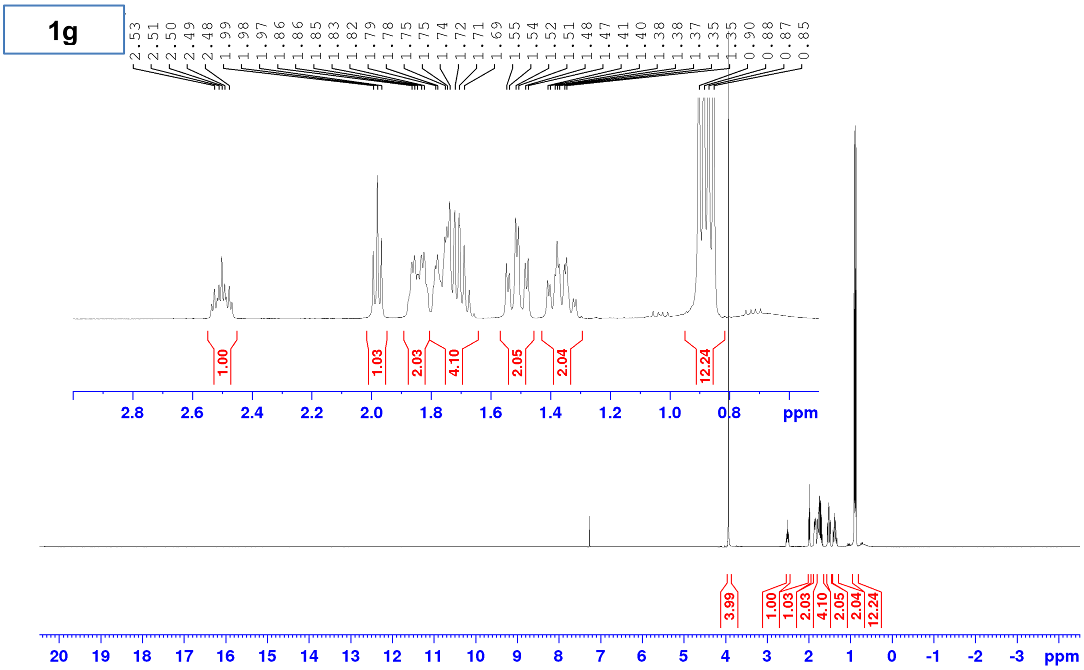

7.26
3.92
2.53
2.51
2.50
2.49
2.48
1.99
1.98
1.97
1.86
1.86
1.85
1.83
1.82
1.79
1.78
1.75
1.75
1.74
1.72
1.71
1.69
1.55
1.54
1.52
1.51
1.48
1.47
1.41
1.40
1.38
1.38
1.37
1.35
0.90
0.88
0.87
0.85
1.00
1.03
2.03
4.10
2.05
2.04
12.24
3.99
ppm

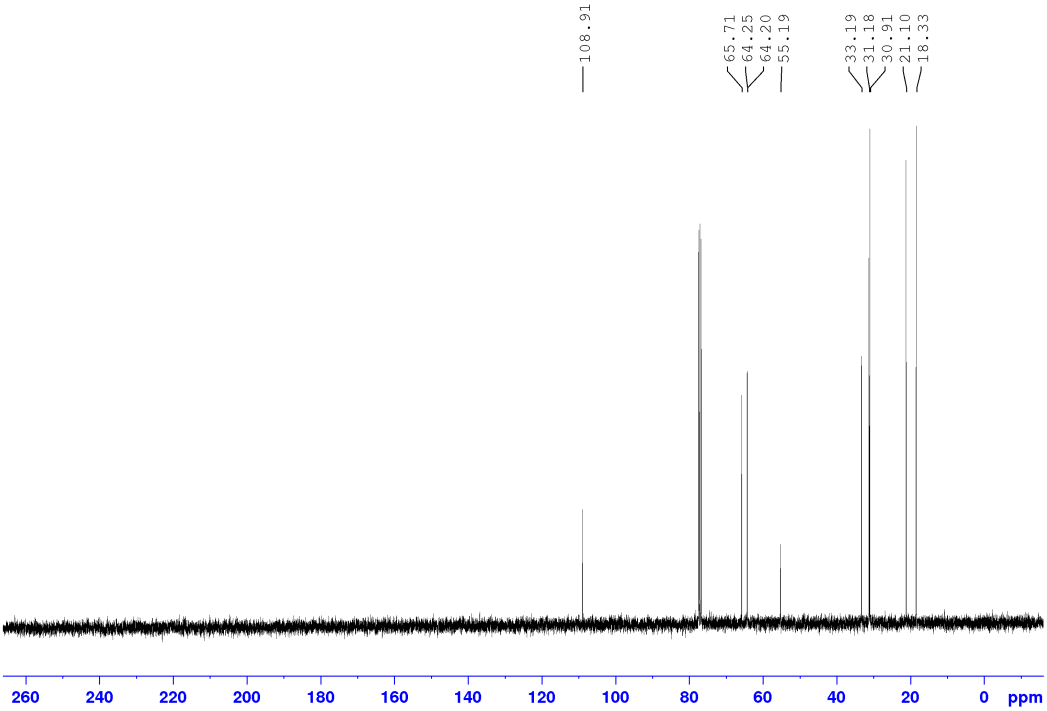

108.91
65.71
64.25
64.20
55.19
33.19
31.18
30.91
21.10
18.33
ppm

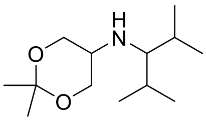

1h

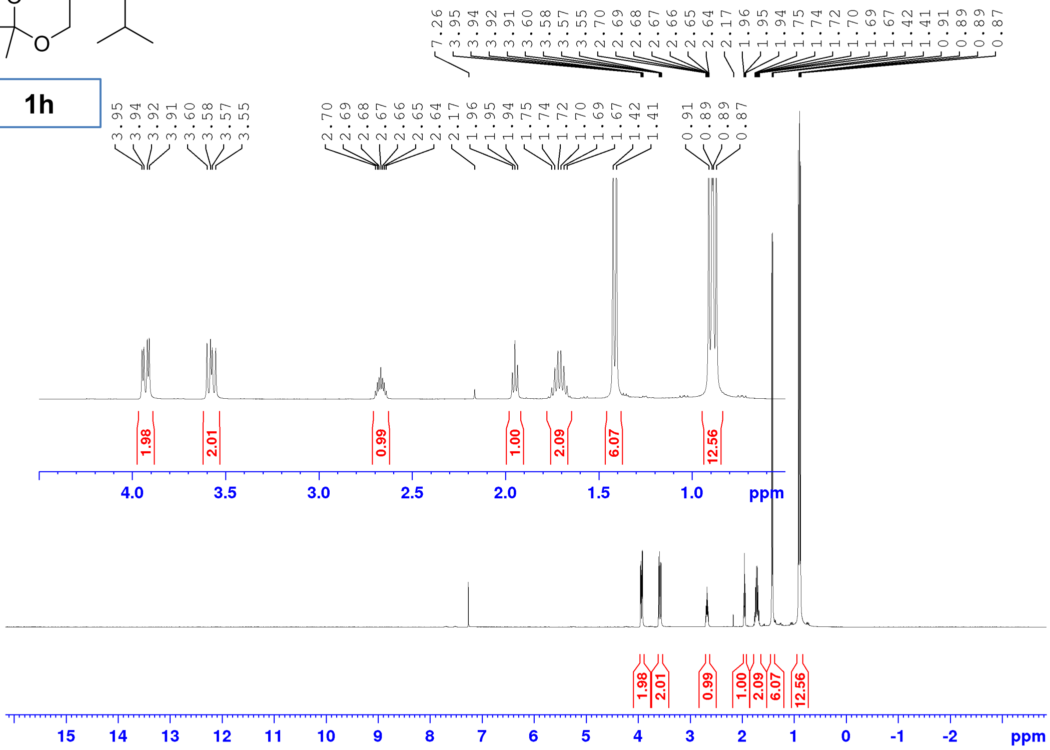
7.26 3.95 3.94 3.92 3.91 3.60 3.58 3.57 3.55 2.70 2.69 2.68 2.67 2.66 2.65 2.64 2.17 1.96 1.95 1.94 1.75 1.74 1.72 1.70 1.69 1.67 1.42 1.41 0.91 0.89 0.89 0.87
1.98 2.01 0.99 1.00 2.09 6.07 12.56
ppm

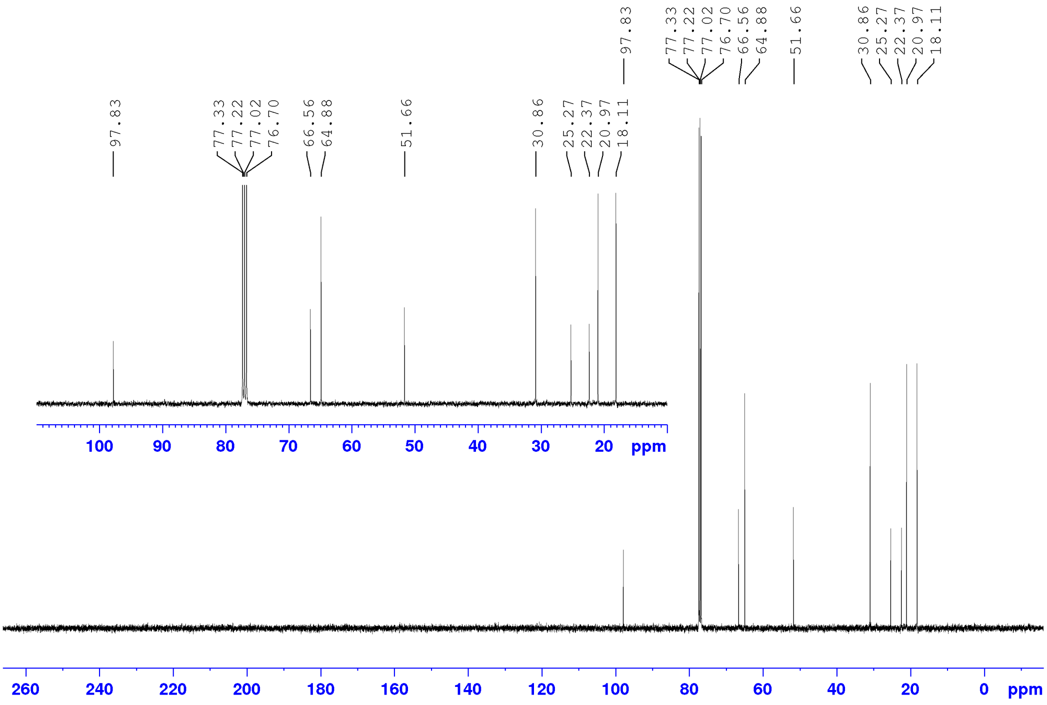
97.83 77.33 77.22 77.02 76.70 66.56 64.88 51.66 30.86 25.27 22.37 20.97 18.11
ppm

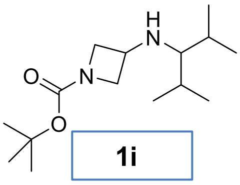

1i

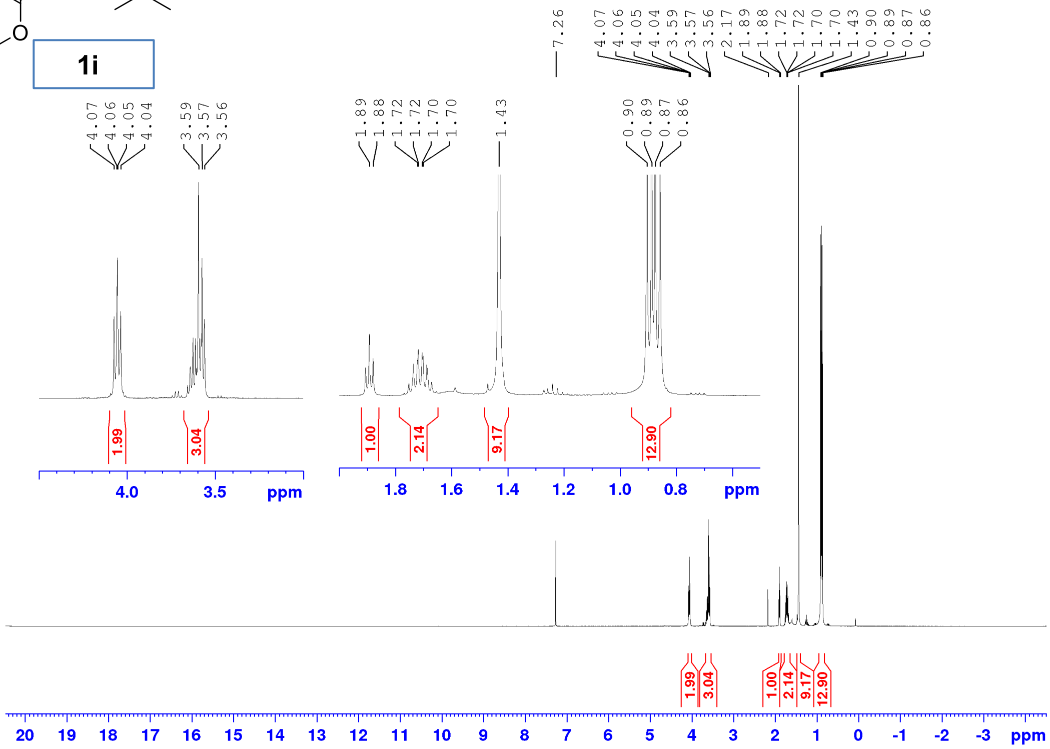
7.26
4.07
4.06
4.05
4.04
3.59
3.57
3.56
2.17
1.89
1.88
1.72
1.72
1.70
1.70
1.43
0.90
0.89
0.87
0.86
1.99
3.04
1.00
2.14
9.17
12.90
ppm

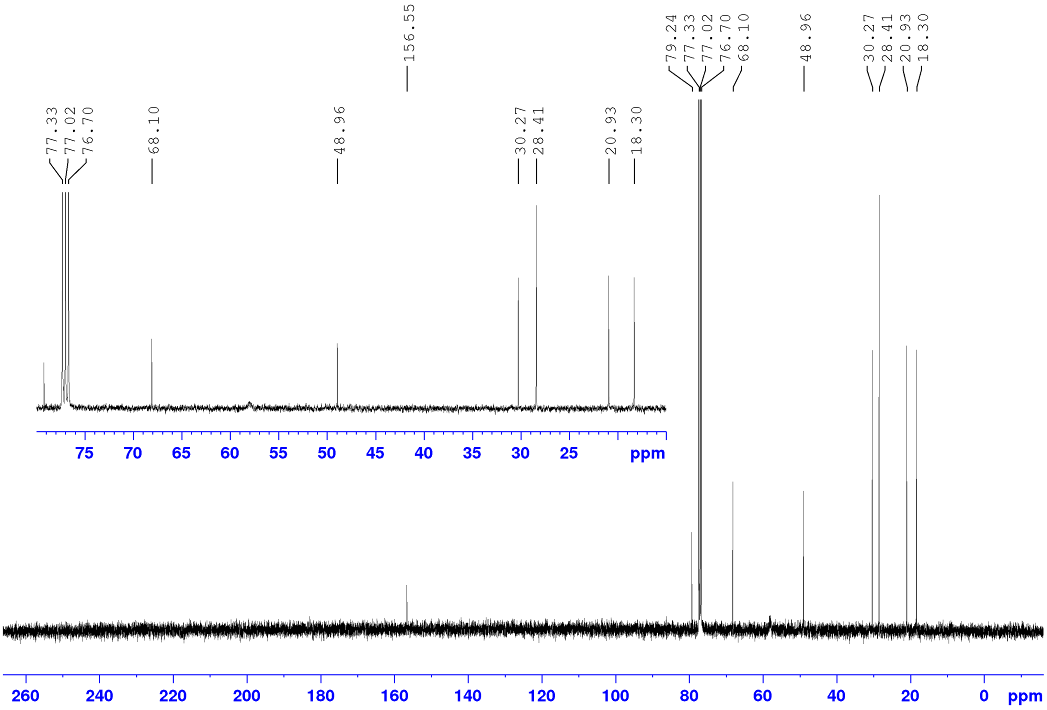
156.55
79.24
77.33
77.02
76.70
68.10
48.96
30.27
28.41
20.93
18.30
ppm

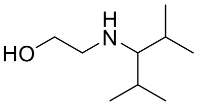
HO
H
N

1j-I

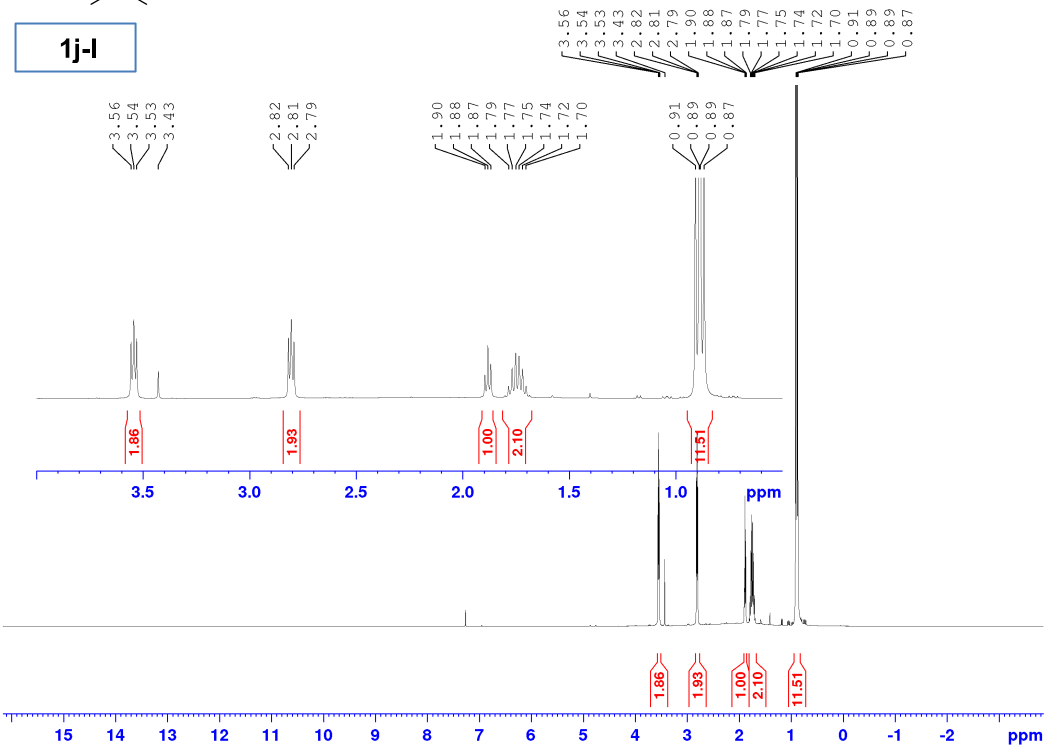
3.56
3.54
3.53
3.43
2.82
2.81
2.79
1.90
1.88
1.87
1.79
1.77
1.75
1.74
1.72
1.70
0.91
0.89
0.89
0.87
1.86
1.93
1.00
2.10
11.51
ppm
ppm

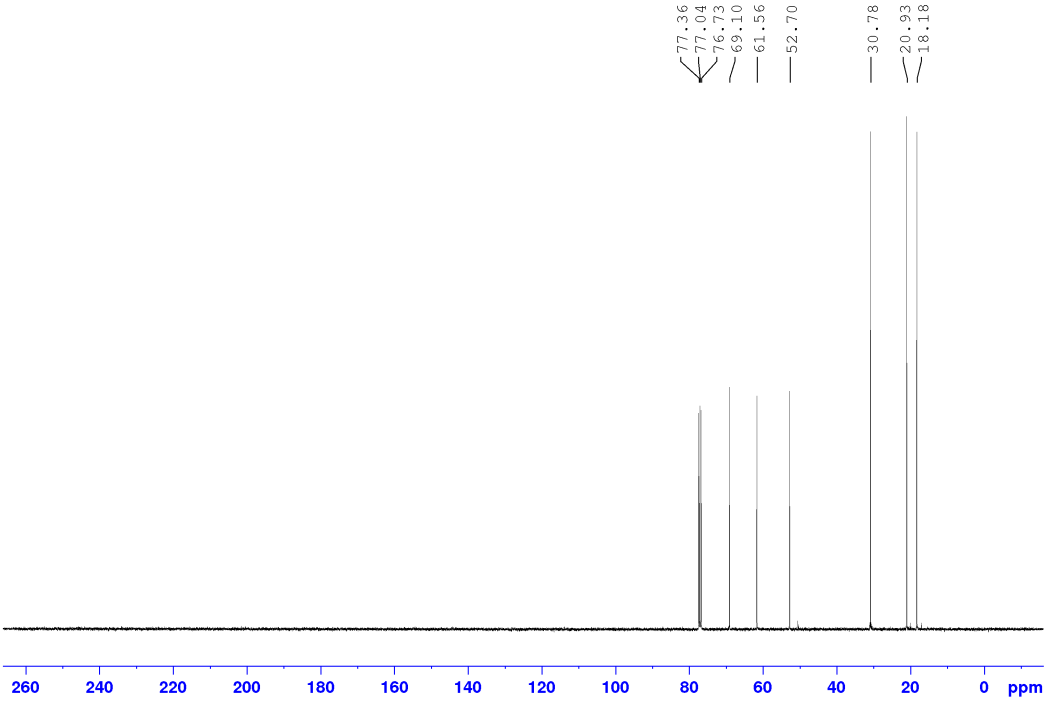
77.36
77.04
76.73
69.10
61.56
52.70
30.78
20.93
18.18
ppm

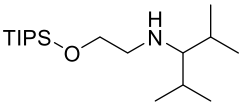
TIPS
O
H
N

1j

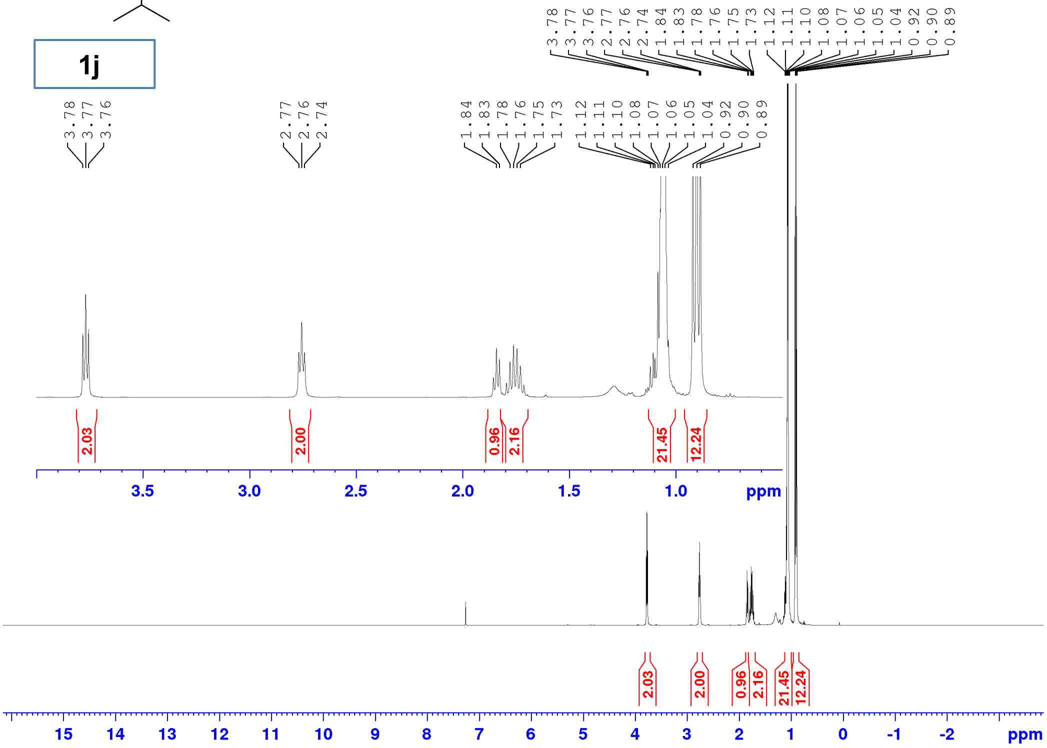
3.78
3.77
3.76
2.77
2.76
2.74
1.84
1.83
1.78
1.76
1.75
1.73
1.12
1.11
1.10
1.08
1.07
1.06
1.05
1.04
0.92
0.90
0.89
2.03
2.00
0.96
2.16
21.45
12.24
3.5
3.0
2.5
2.0
1.5
1.0
ppm
15
14
13
12
11
10
9
8
7
6
5
4
3
2
1
0
-1
-2
ppm

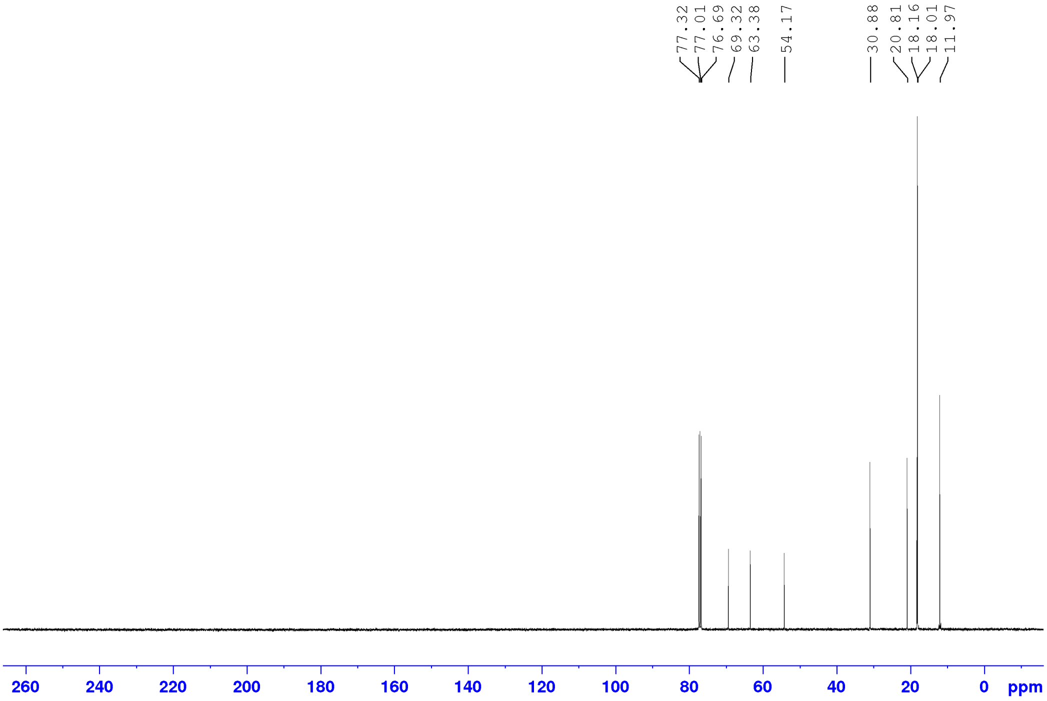
77.32
77.01
76.69
69.32
63.38
54.17
30.88
20.81
18.16
18.01
11.97
260
240
220
200
180
160
140
120
100
80
60
40
20
0
ppm

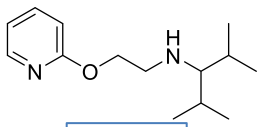

1k

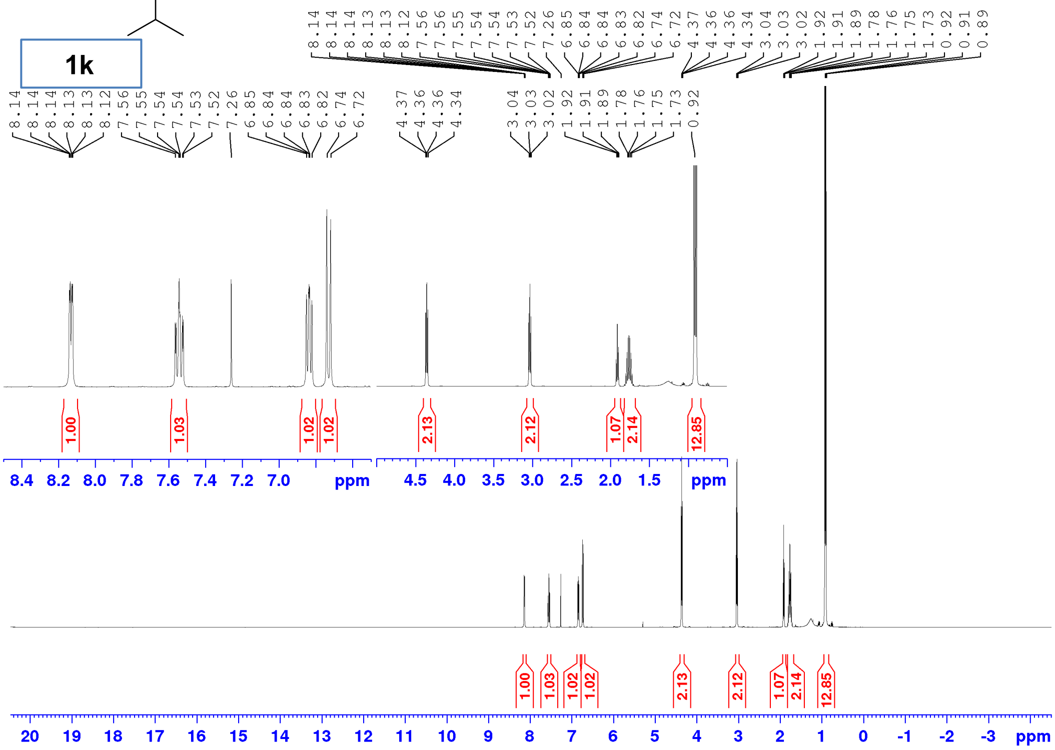

8.14 8.14 8.14 8.13 8.13 8.12 7.56 7.56 7.55 7.54 7.54 7.53 7.52 7.26 6.85 6.84 6.84 6.83 6.82 6.74 6.72 4.37 4.36 4.36 4.34 3.04 3.03 3.02 1.92 1.91 1.89 1.78 1.76 1.75 1.73 0.92 0.91 0.89
1.00 1.03 1.02 1.02 2.13 2.12 1.07 2.14 12.85
ppm

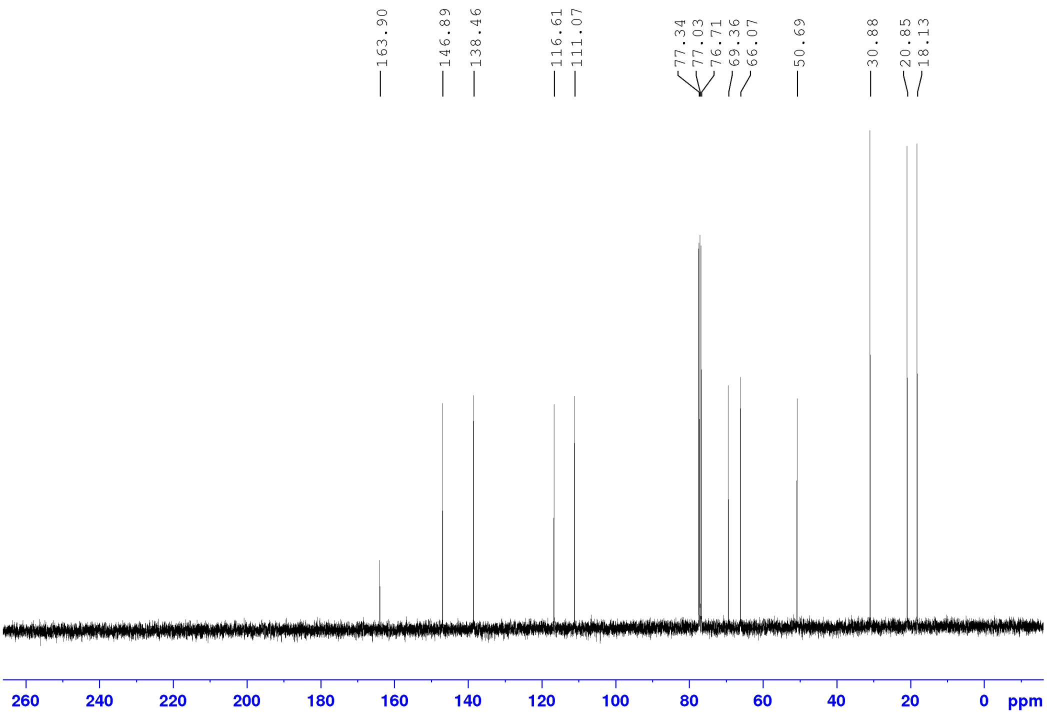

163.90 146.89 138.46 116.61 111.07 77.34 77.03 76.71 69.36 66.07 50.69 30.88 20.85 18.13
ppm

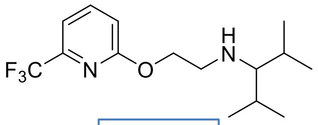

1l

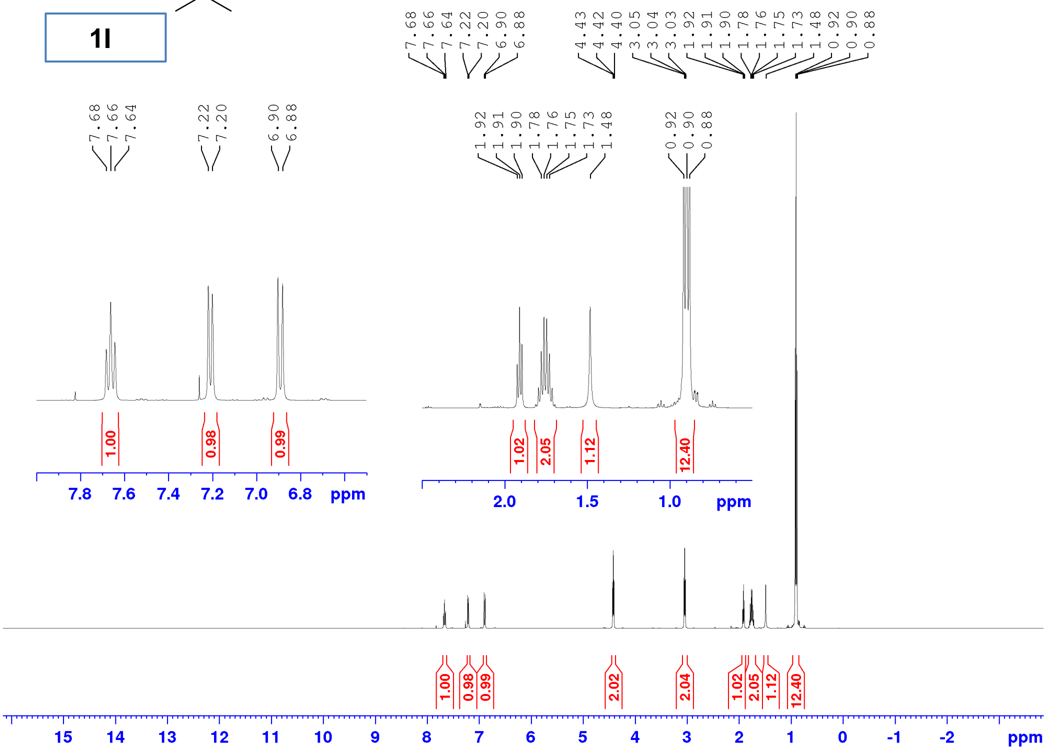

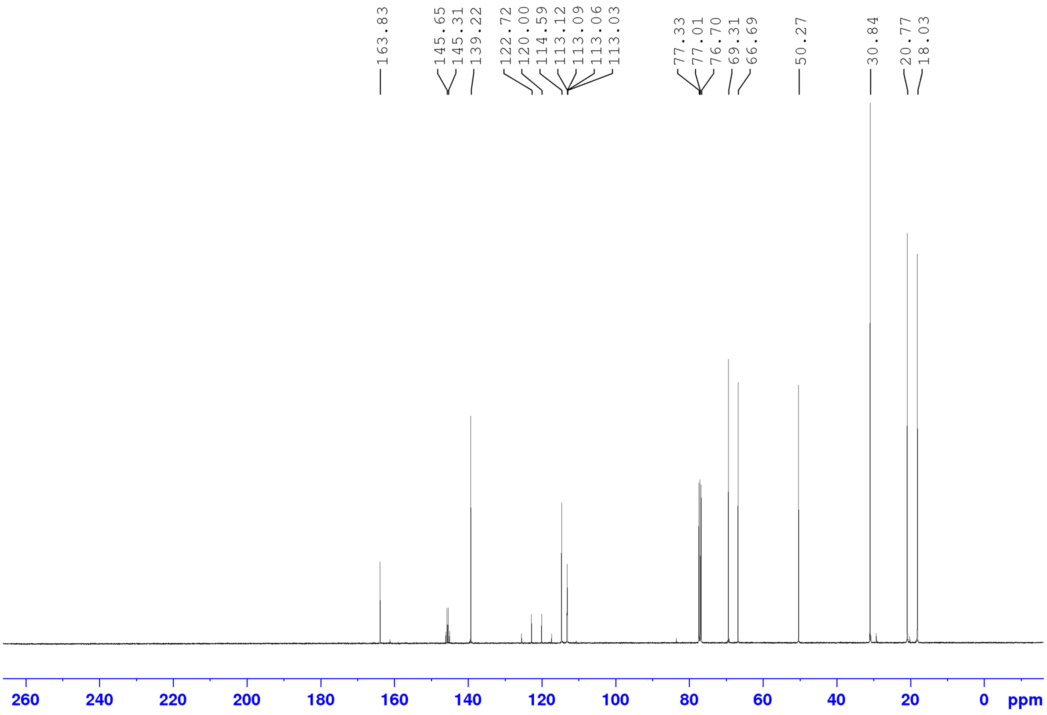

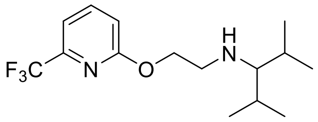

1l

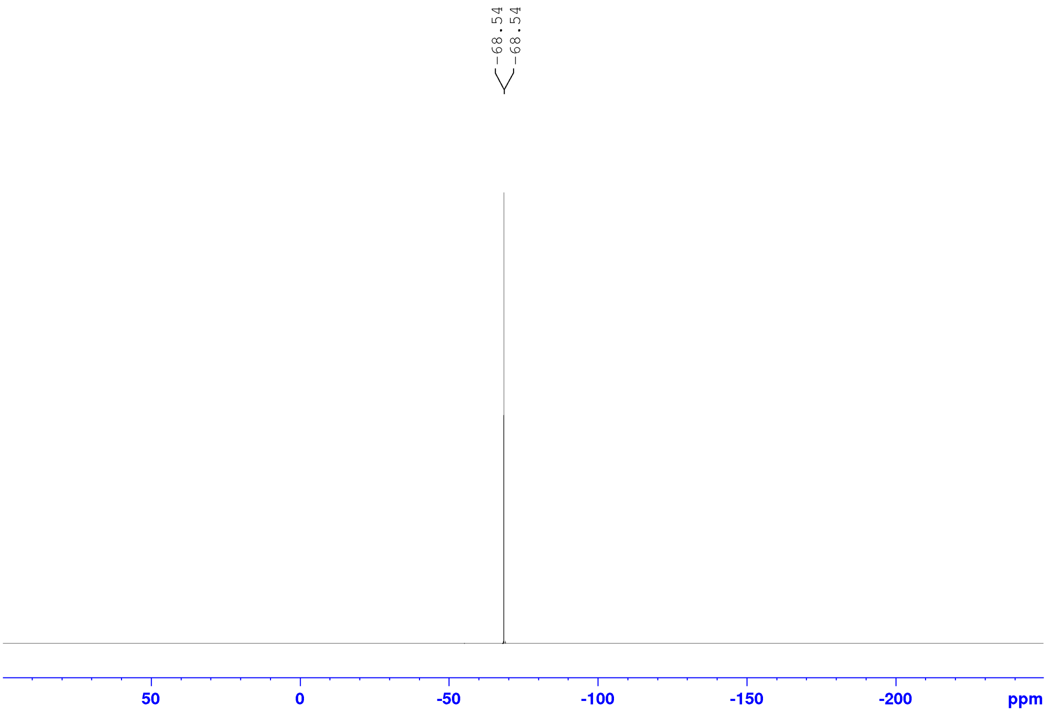

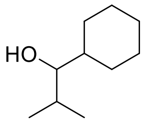

1m-I

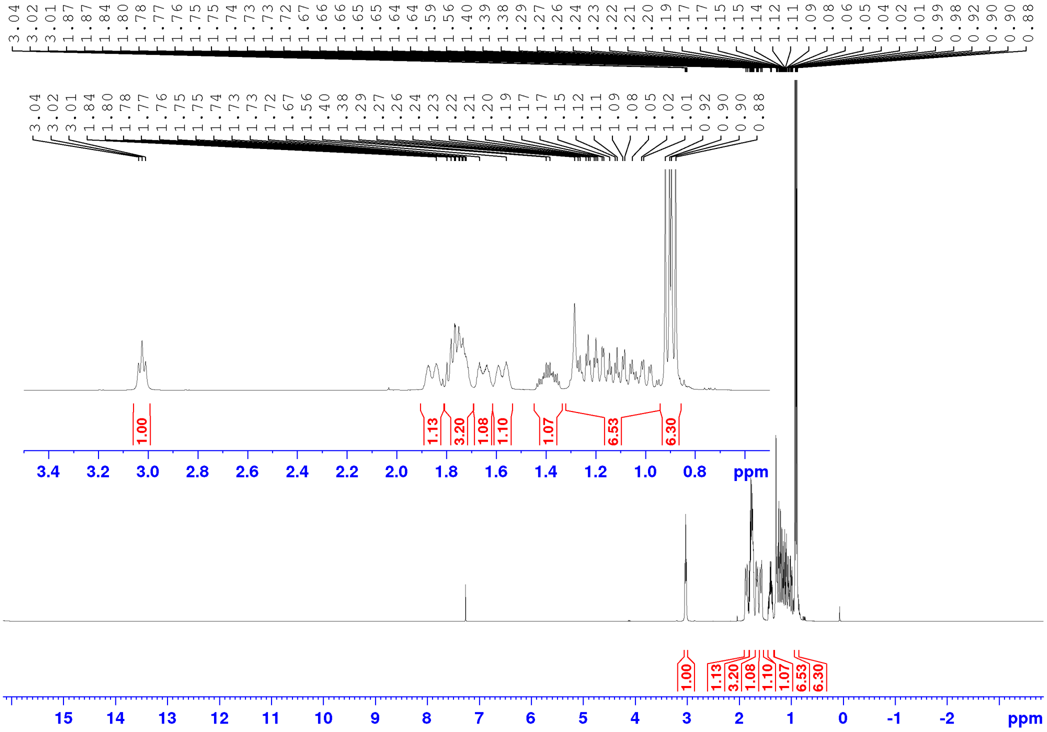
3.04 3.02 3.01 1.87 1.87 1.84 1.80 1.78 1.77 1.76 1.75 1.75 1.74 1.73 1.73 1.72 1.67 1.66 1.66 1.65 1.64 1.64 1.59 1.56 1.40 1.39 1.38 1.29 1.27 1.26 1.24 1.23 1.22 1.21 1.20 1.19 1.17 1.17 1.15 1.15 1.14 1.12 1.11 1.09 1.08 1.06 1.05 1.04 1.02 1.01 0.99 0.98 0.92 0.90 0.90 0.88
1.00 1.13 3.20 1.08 1.10 1.07 6.53 6.30
ppm

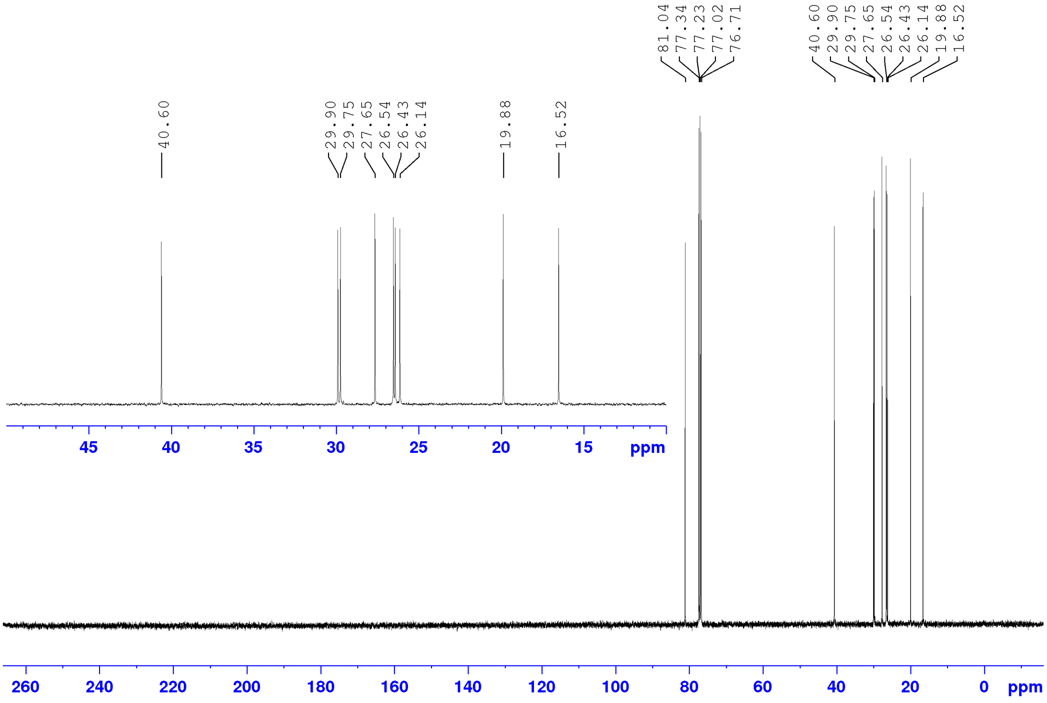
81.04 77.34 77.23 77.02 76.71
40.60 29.90 29.75 27.65 26.54 26.43 26.14 19.88 16.52
ppm

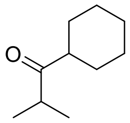

1m-II

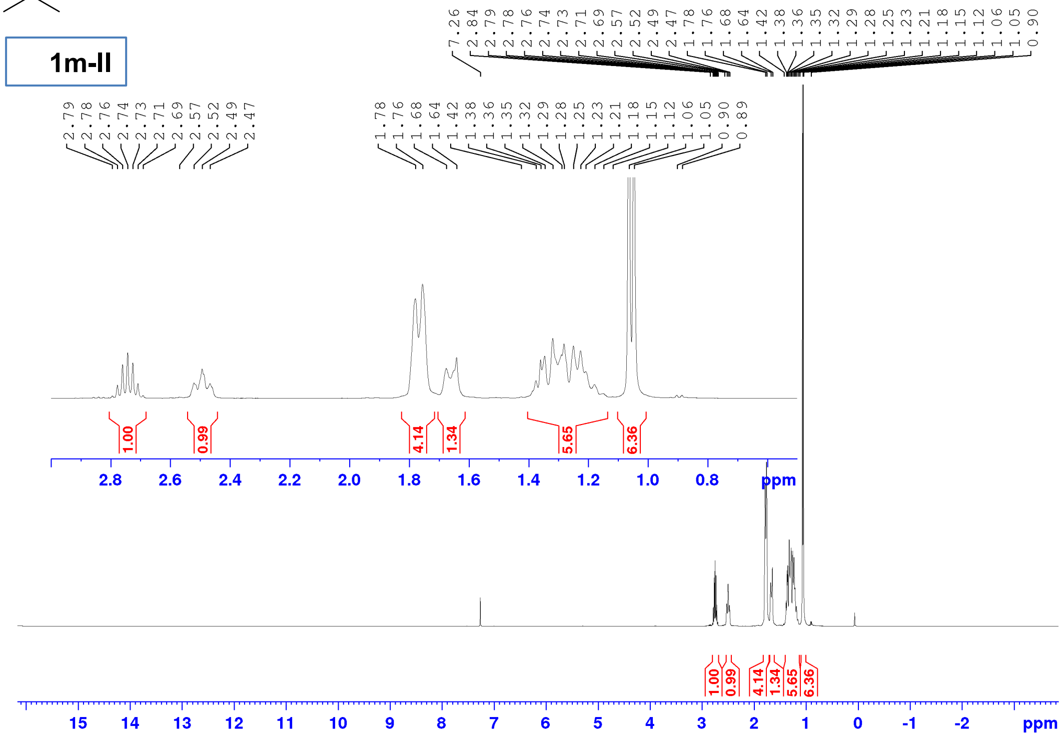
7.26
2.84 2.79 2.78 2.76 2.74 2.73 2.71 2.69 2.57 2.52 2.49 2.47 1.78 1.76 1.68 1.64 1.42 1.38 1.36 1.35 1.32 1.29 1.28 1.25 1.23 1.21 1.18 1.15 1.12 1.06 1.05 0.90
2.79 2.78 2.76 2.74 2.73 2.71 2.69 2.57 2.52 2.49 2.47
1.78 1.76 1.68 1.64 1.42 1.38 1.36 1.35 1.32 1.29 1.28 1.25 1.23 1.21 1.18 1.15 1.12 1.06 1.05 0.90 0.89
1.00 0.99 4.14 1.34 5.65 6.36
2.8 2.6 2.4 2.2 2.0 1.8 1.6 1.4 1.2 1.0 0.8 ppm
15 14 13 12 11 10 9 8 7 6 5 4 3 2 1 0 -1 -2 ppm

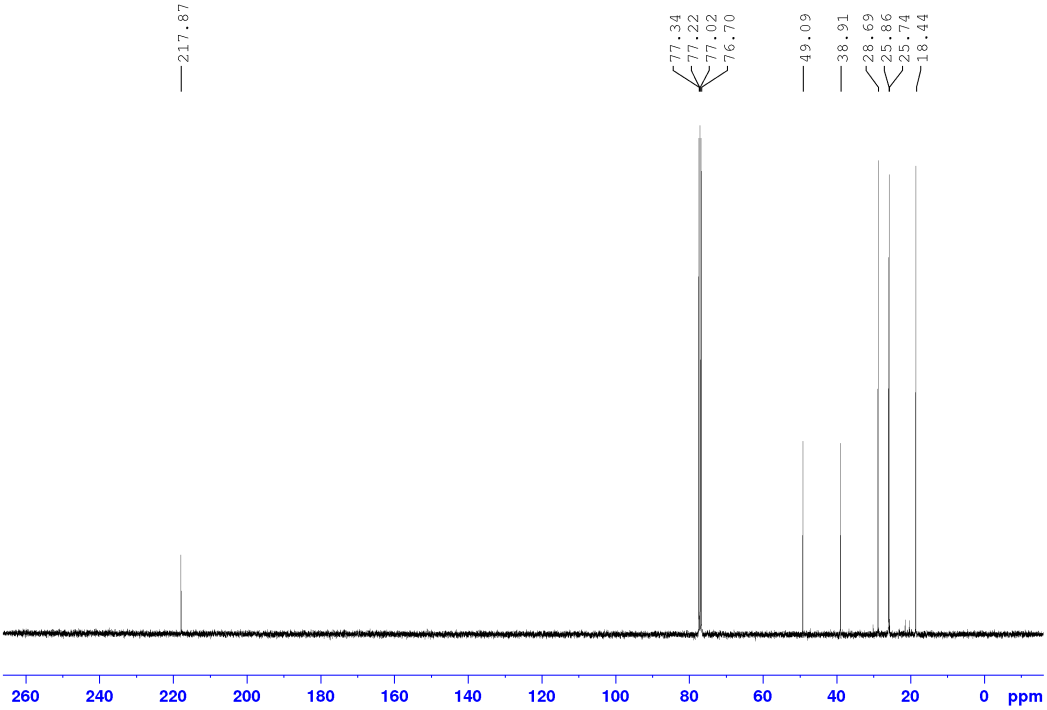
217.87
77.34 77.22 77.02 76.70
49.09
38.91
28.69 25.86 25.74 18.44
260 240 220 200 180 160 140 120 100 80 60 40 20 0 ppm

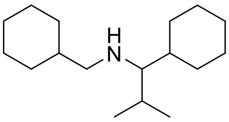

1m

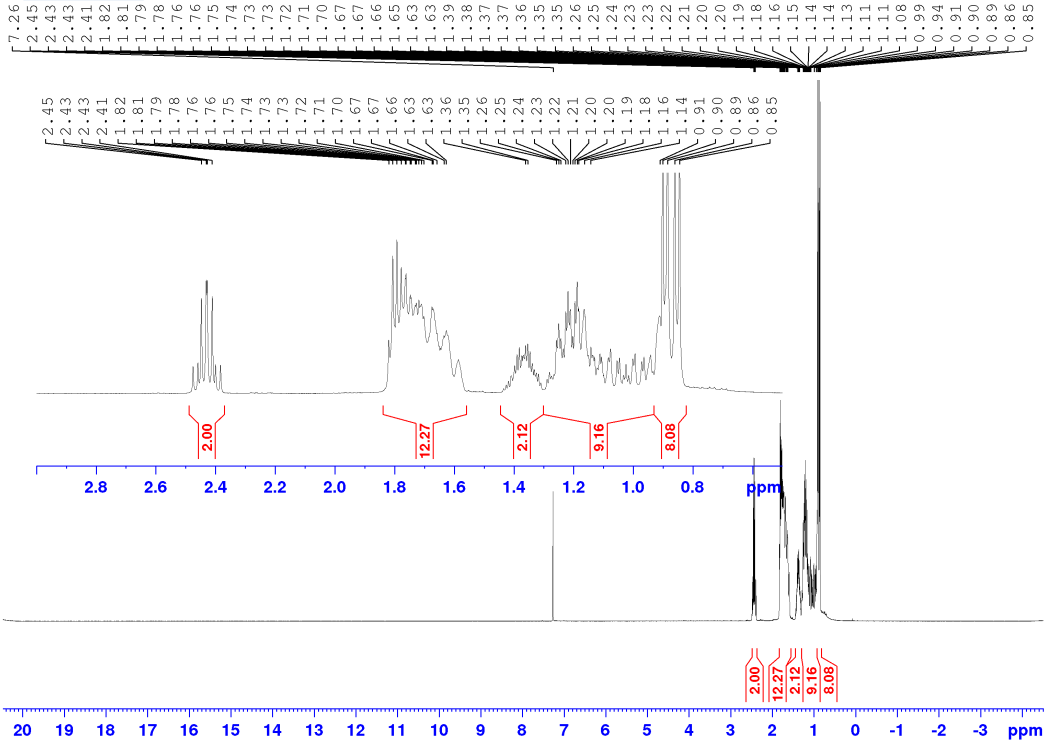
7.26 2.45 2.43 2.43 2.41 1.82 1.81 1.79 1.78 1.76 1.76 1.75 1.74 1.73 1.73 1.72 1.71 1.70 1.67 1.66 1.65 1.63 1.63 1.39 1.38 1.37 1.37 1.36 1.35 1.35 1.26 1.25 1.24 1.23 1.23 1.22 1.21 1.20 1.19 1.18 1.16 1.15 1.14 1.14 1.13 1.11 1.11 1.08 0.99 0.94 0.91 0.90 0.89 0.86 0.85
2.00
12.27
2.12
9.16
8.08
ppm

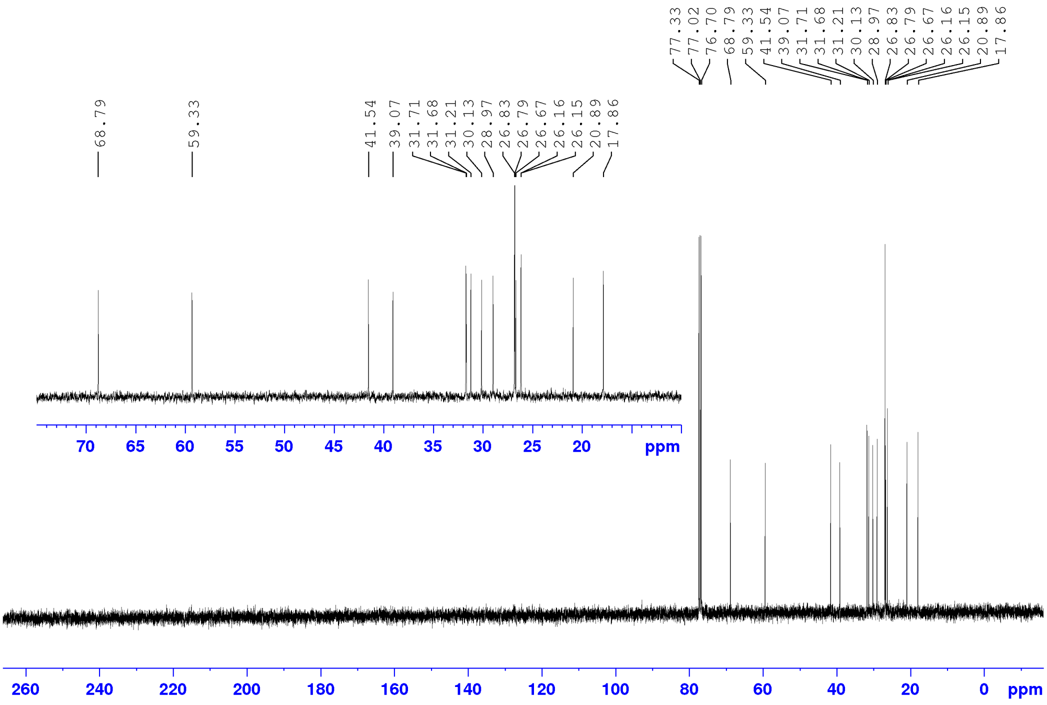
77.33 77.02 76.70 68.79 59.33 41.54 39.07 31.71 31.68 31.21 30.13 28.97 26.83 26.79 26.67 26.16 26.15 20.89 17.86
ppm

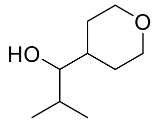

1n-I

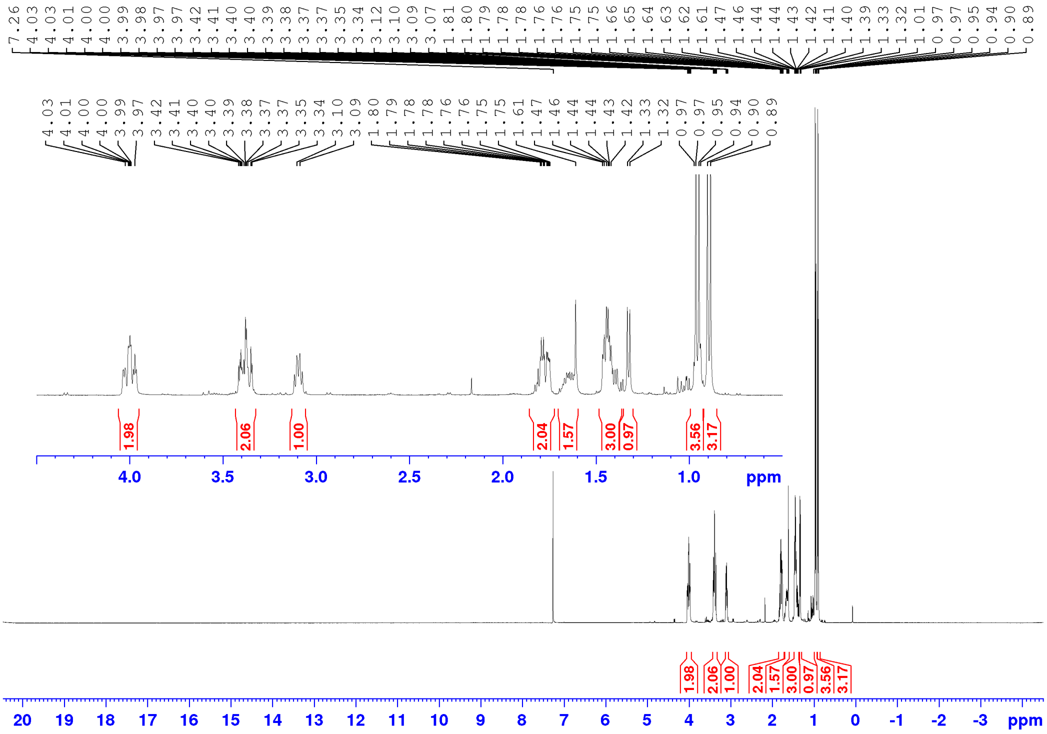
7.26
4.03
4.03
4.01
4.00
4.00
3.99
3.98
3.97
3.97
3.42
3.41
3.40
3.40
3.39
3.38
3.37
3.37
3.35
3.34
3.12
3.10
3.09
3.07
1.81
1.80
1.79
1.78
1.78
1.76
1.76
1.75
1.75
1.66
1.65
1.64
1.63
1.62
1.61
1.47
1.46
1.44
1.44
1.43
1.42
1.41
1.40
1.39
1.33
1.32
1.01
0.97
0.97
0.95
0.94
0.90
0.89
1.98
2.06
1.00
2.04
1.57
3.00
0.97
3.56
3.17
ppm

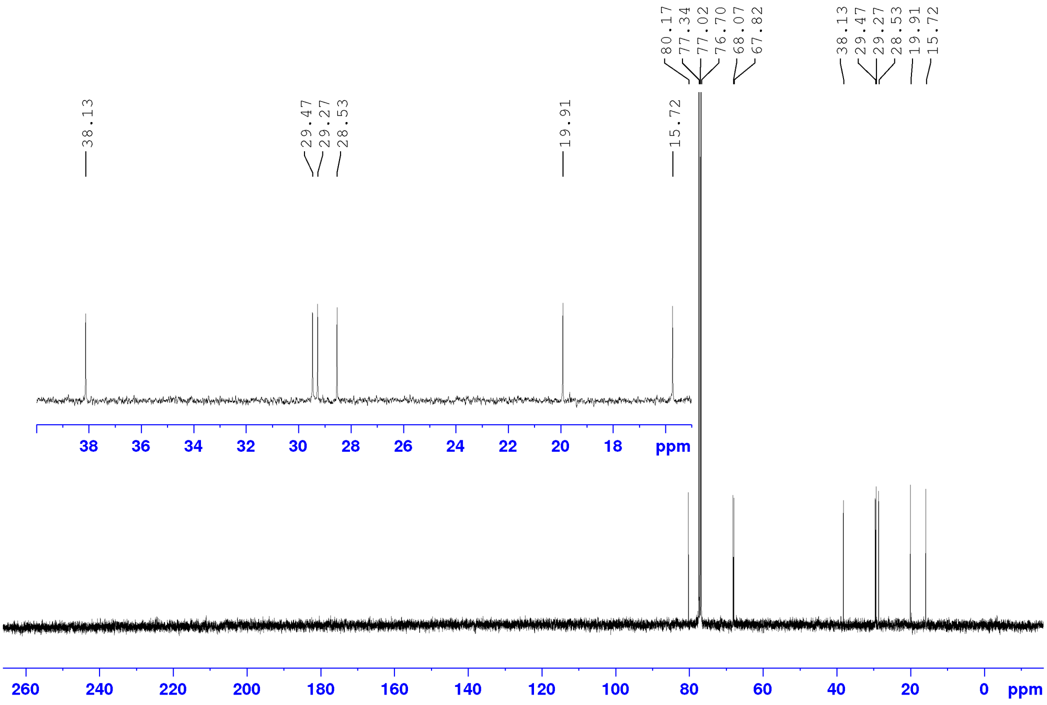
80.17
77.34
77.02
76.70
68.07
67.82
38.13
29.47
29.27
28.53
19.91
15.72
ppm

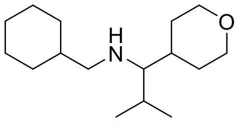

1n

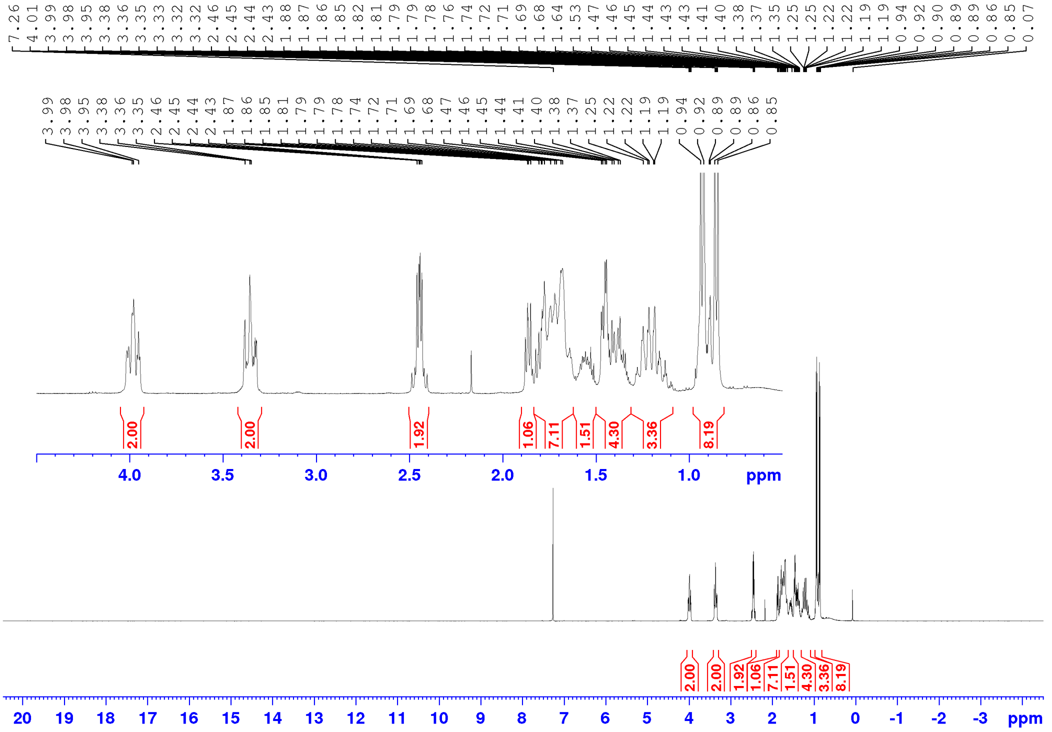
7.26 4.01 3.99 3.98 3.95 3.38 3.36 3.35 3.33 3.32 2.46 2.45 2.44 2.43 1.88 1.87 1.86 1.85 1.82 1.81 1.79 1.79 1.78 1.76 1.74 1.72 1.71 1.69 1.68 1.64 1.53 1.47 1.46 1.45 1.44 1.43 1.43 1.41 1.40 1.38 1.37 1.35 1.25 1.25 1.22 1.22 1.19 1.19 0.94 0.92 0.90 0.89 0.89 0.86 0.85 0.07
2.00 2.00 1.92 1.06 7.11 1.51 4.30 3.36 8.19
ppm

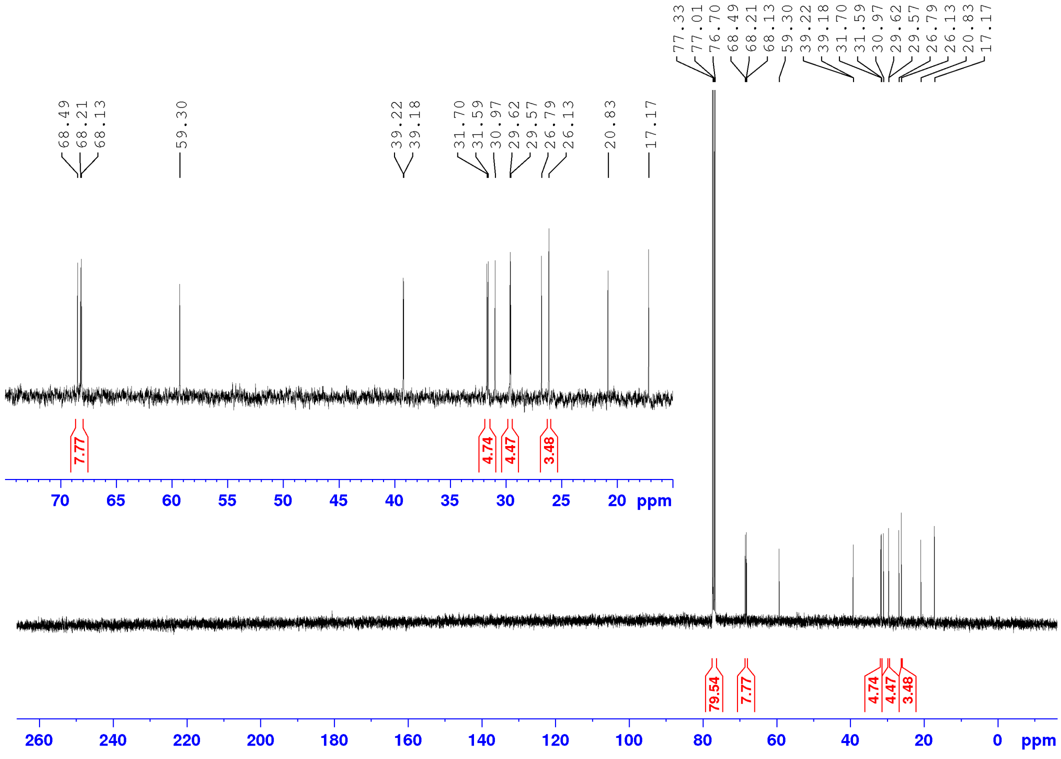
77.33 77.01 76.70 68.49 68.21 68.13 59.30 39.22 39.18 31.70 31.59 30.97 29.62 29.57 26.79 26.13 20.83 17.17
7.77 4.74 4.47 3.48 79.54
ppm

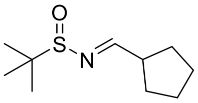
O
S
N

1o-l

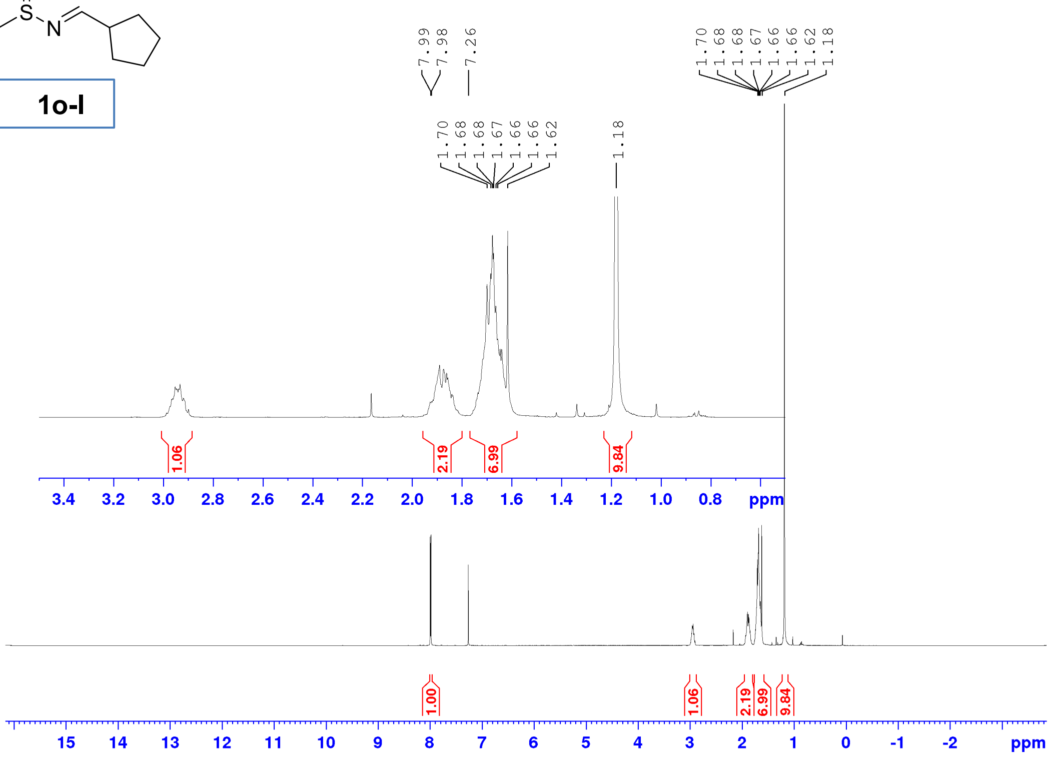
7.99
7.98
7.26
1.70
1.68
1.68
1.67
1.66
1.66
1.62
1.18
1.70
1.68
1.68
1.67
1.66
1.66
1.62
1.18
1.06
2.19
6.99
9.84
3.4 3.2 3.0 2.8 2.6 2.4 2.2 2.0 1.8 1.6 1.4 1.2 1.0 0.8 ppm
1.00
1.06
2.19
6.99
9.84
15 14 13 12 11 10 9 8 7 6 5 4 3 2 1 0 -1 -2 ppm

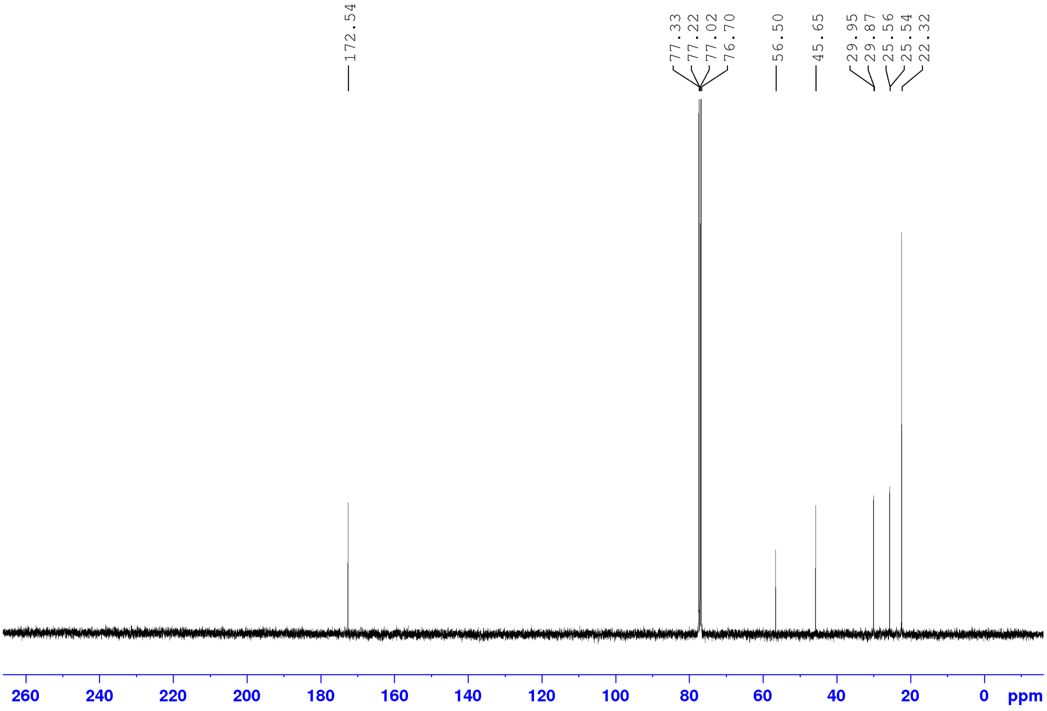
172.54
77.33
77.22
77.02
76.70
56.50
45.65
29.95
29.87
25.56
25.54
22.32
260 240 220 200 180 160 140 120 100 80 60 40 20 0 ppm

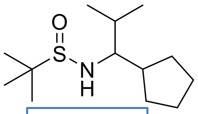
O
S
N
H

1o-II

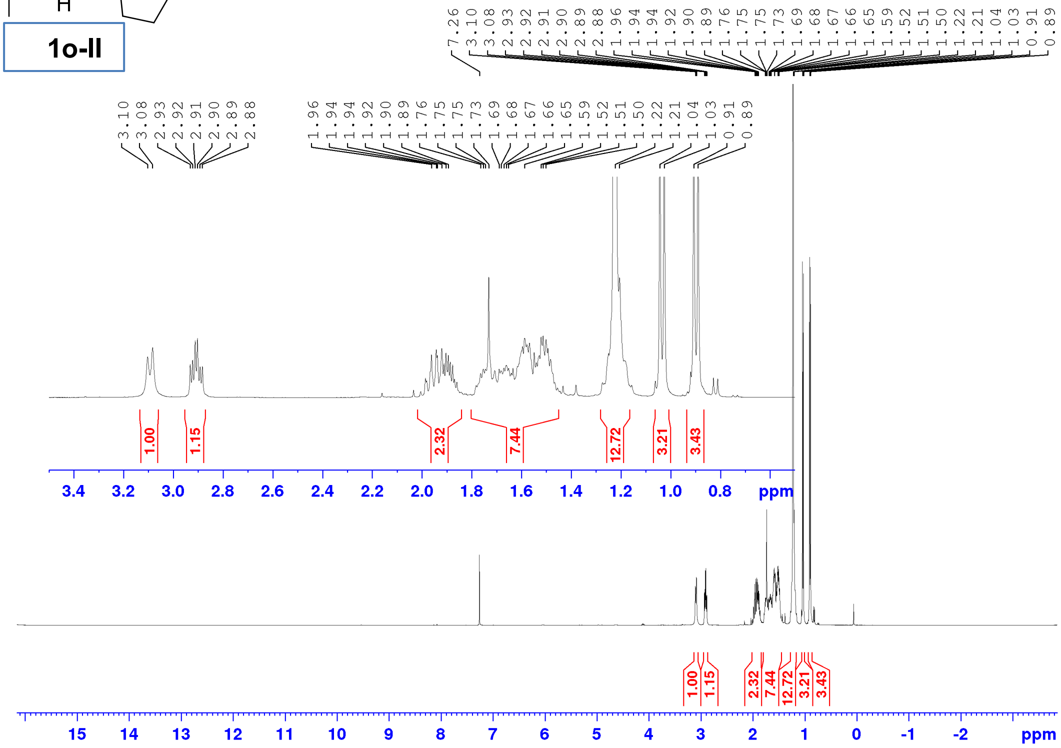
7.26
3.10
3.08
2.93
2.92
2.91
2.90
2.89
2.88
1.96
1.94
1.94
1.92
1.90
1.89
1.76
1.75
1.75
1.73
1.69
1.68
1.67
1.66
1.65
1.59
1.52
1.51
1.50
1.22
1.21
1.04
1.03
0.91
0.89
1.00
1.15
2.32
7.44
12.72
3.21
3.43
3.4
3.2
3.0
2.8
2.6
2.4
2.2
2.0
1.8
1.6
1.4
1.2
1.0
0.8
ppm
15
14
13
12
11
10
9
8
7
6
5
4
3
2
1
0
-1
-2
ppm

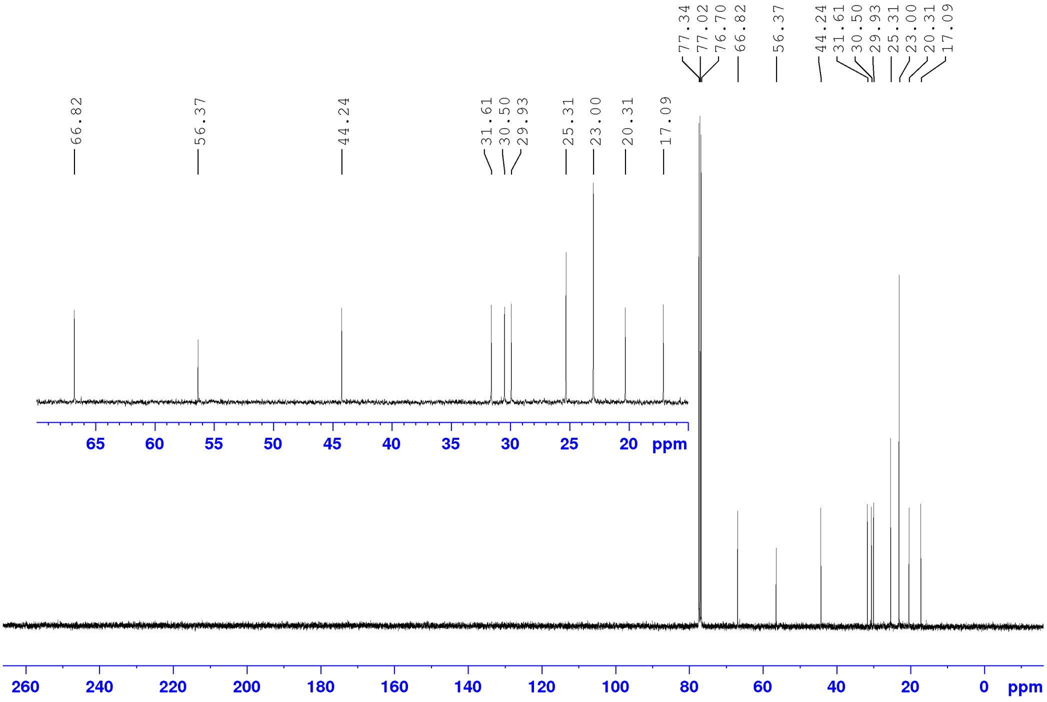
77.34
77.02
76.70
66.82
56.37
44.24
31.61
30.50
29.93
25.31
23.00
20.31
17.09
65
60
55
50
45
40
35
30
25
20
ppm
260
240
220
200
180
160
140
120
100
80
60
40
20
0
ppm

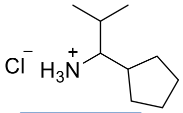
Cl⁻ H3N⁺

1o-III

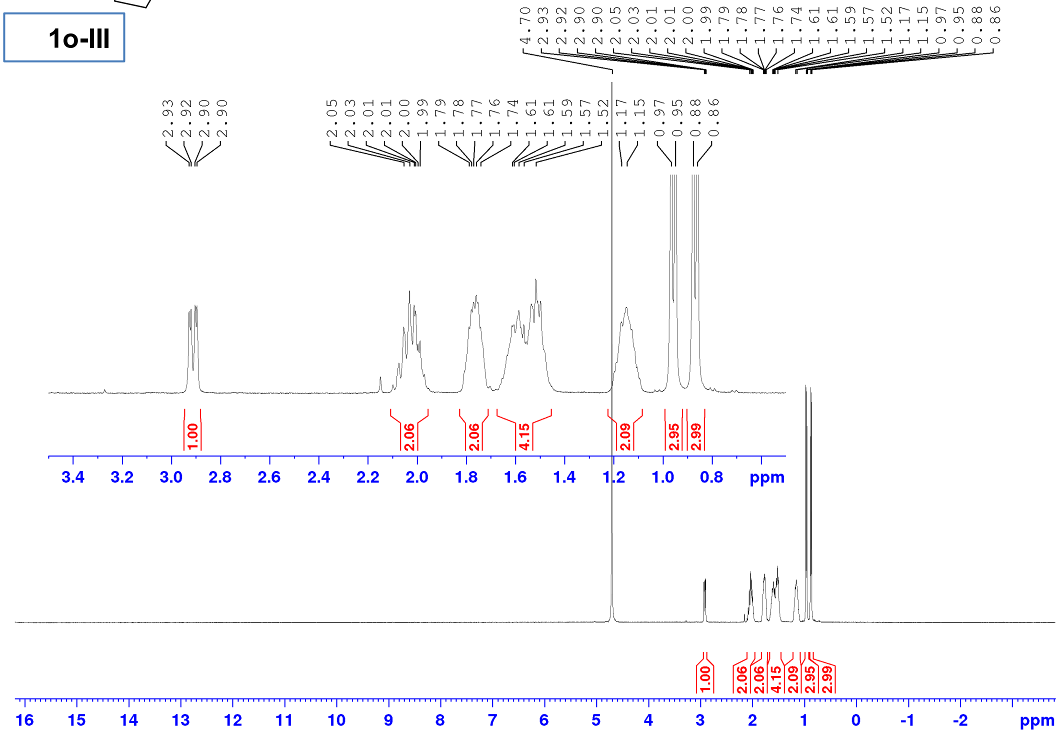
4.70 2.93 2.92 2.90 2.90 2.05 2.03 2.01 2.01 2.00 1.99 1.79 1.78 1.77 1.76 1.74 1.61 1.61 1.59 1.57 1.52 1.17 1.15 0.97 0.95 0.88 0.86
2.93 2.92 2.90 2.90
2.05 2.03 2.01 2.01 2.00 1.99 1.79 1.78 1.77 1.76 1.74 1.61 1.61 1.59 1.57 1.52 1.17 1.15 0.97 0.95 0.88 0.86
1.00 2.06 2.06 4.15 2.09 2.95 2.99
3.4 3.2 3.0 2.8 2.6 2.4 2.2 2.0 1.8 1.6 1.4 1.2 1.0 0.8 ppm
1.00 2.06 2.06 4.15 2.09 2.95 2.99
16 15 14 13 12 11 10 9 8 7 6 5 4 3 2 1 0 -1 -2 ppm

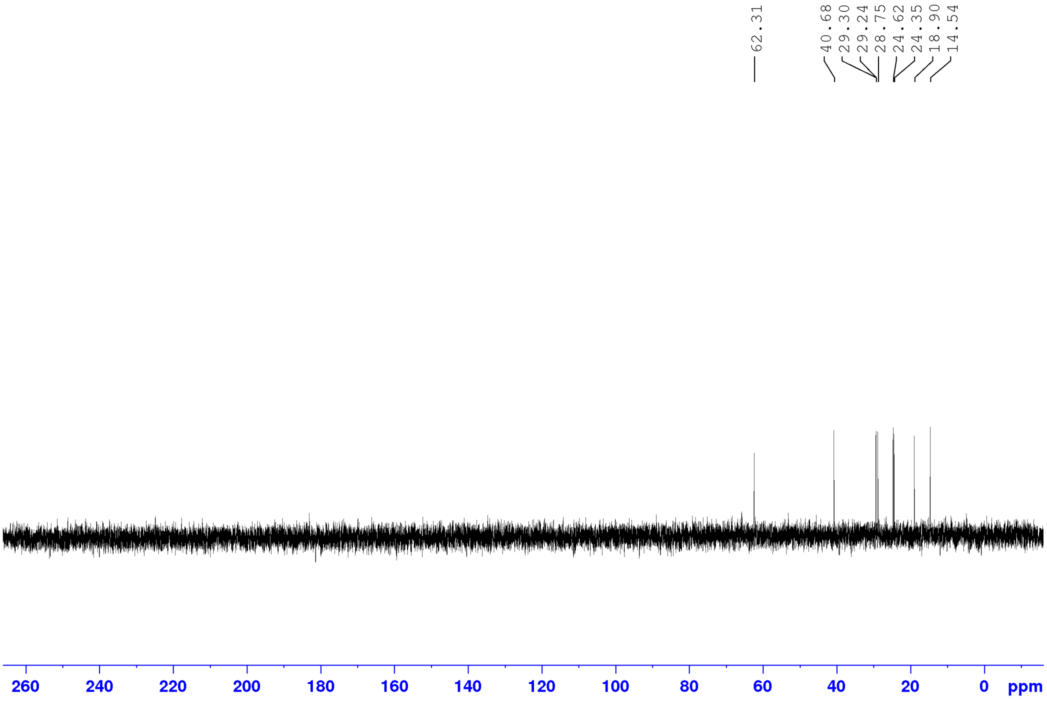
62.31
40.68 29.30 29.24 28.75 24.62 24.35 18.90 14.54
260 240 220 200 180 160 140 120 100 80 60 40 20 0 ppm

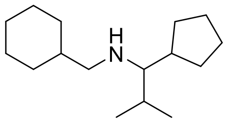
H
N

1o

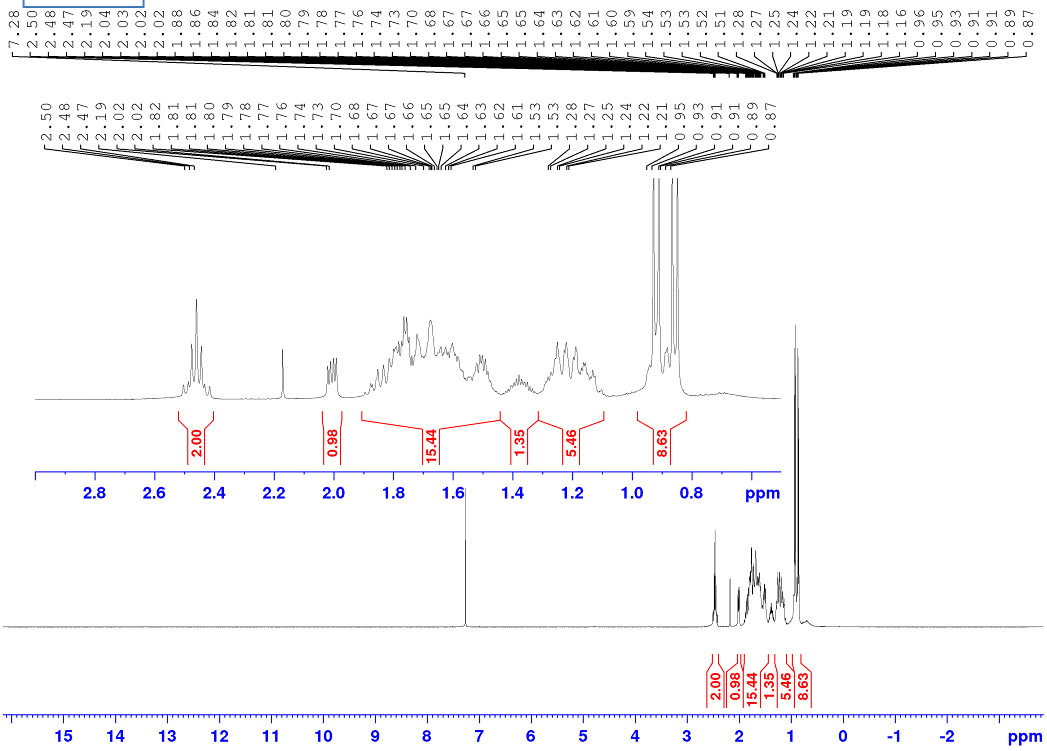
7.28
2.50
2.48
2.47
2.19
2.04
2.03
2.02
2.02
1.88
1.86
1.84
1.82
1.81
1.81
1.80
1.79
1.78
1.77
1.76
1.74
1.73
1.70
1.68
1.67
1.67
1.66
1.65
1.65
1.64
1.63
1.62
1.61
1.60
1.59
1.54
1.53
1.53
1.52
1.51
1.28
1.27
1.25
1.24
1.22
1.21
1.19
1.19
1.18
1.16
0.96
0.95
0.93
0.91
0.91
0.89
0.87
2.00
0.98
15.44
1.35
5.46
8.63
ppm

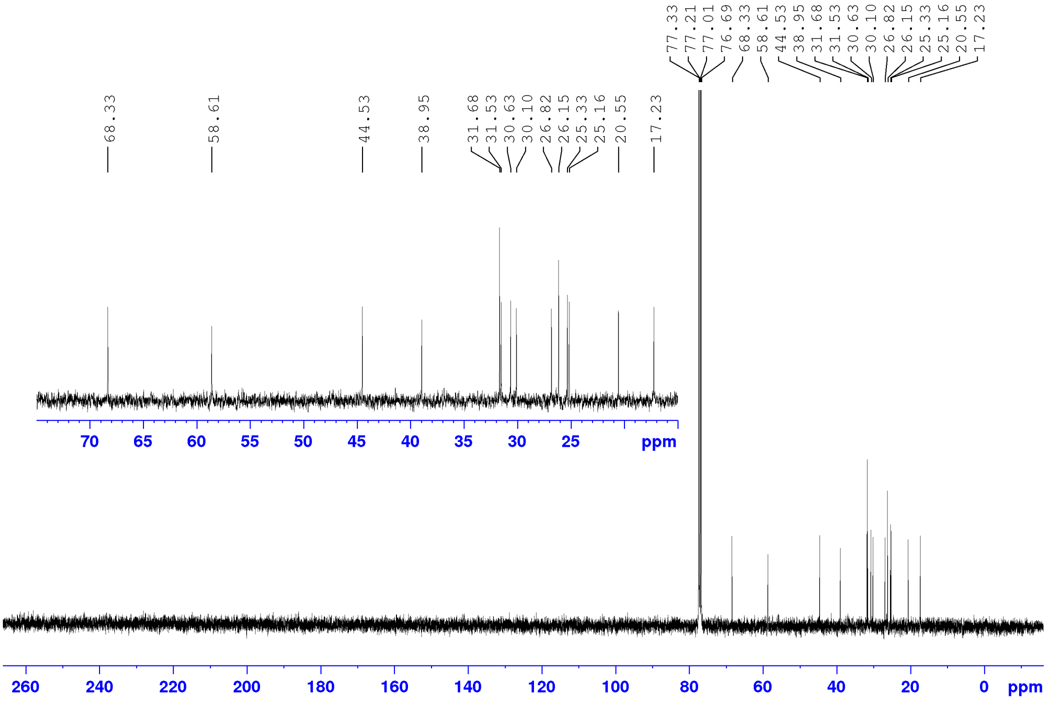
77.33
77.21
77.01
76.69
68.33
58.61
44.53
38.95
31.68
31.53
30.63
30.10
26.82
26.15
25.33
25.16
20.55
17.23
ppm

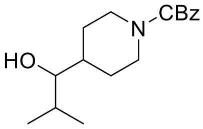

1p-I

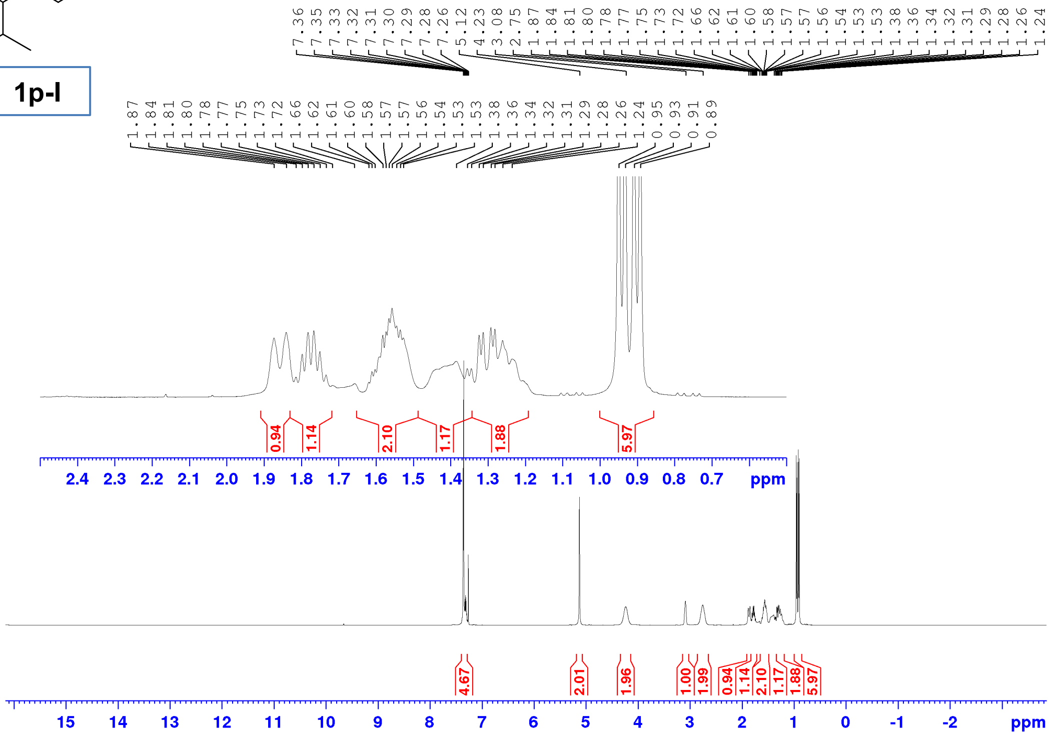

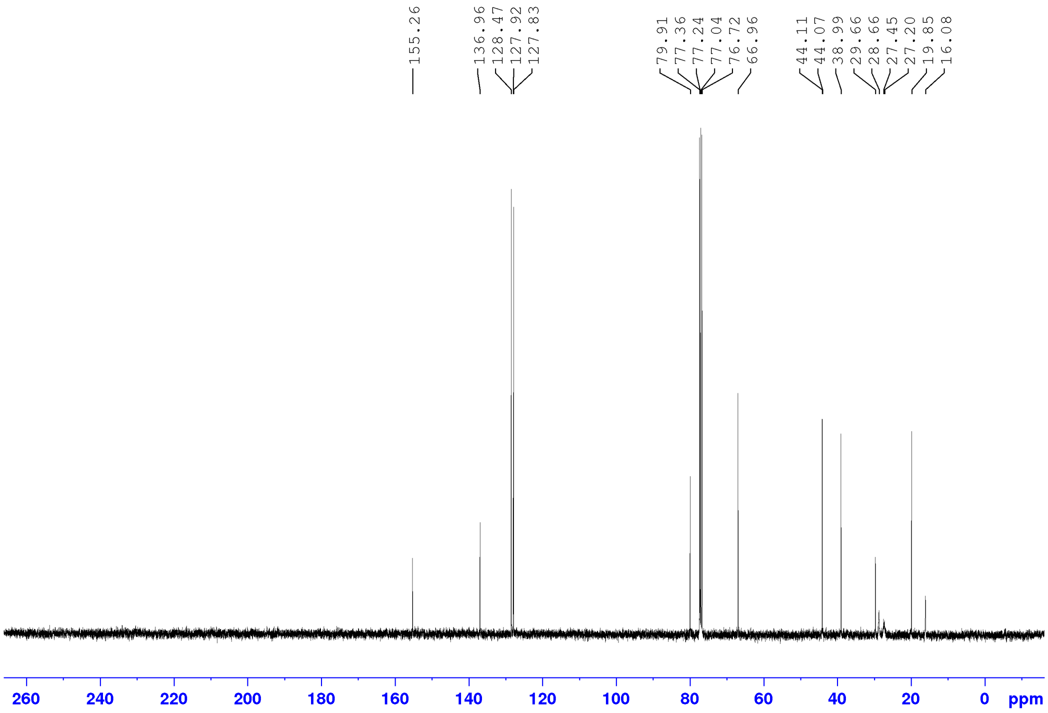

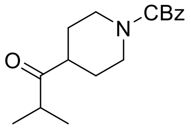
O
N
CBz

1p-II

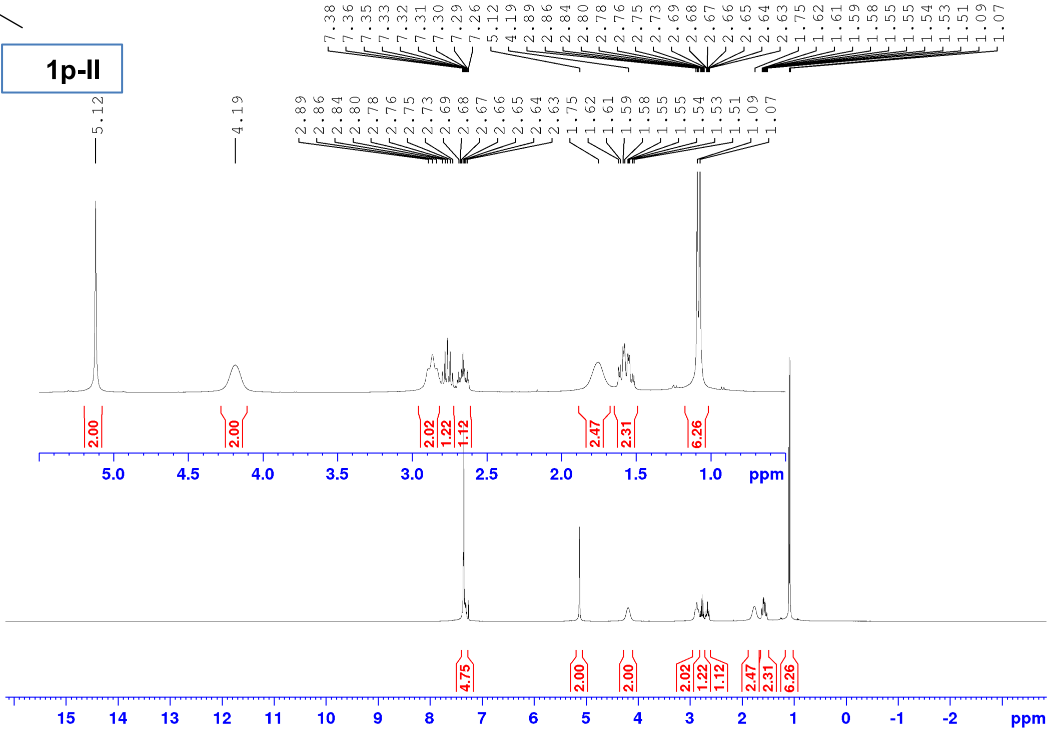
7.38 7.36 7.35 7.33 7.32 7.31 7.30 7.29 7.26 5.12 4.19 2.89 2.86 2.84 2.80 2.78 2.76 2.75 2.73 2.69 2.68 2.67 2.66 2.65 2.64 2.63 1.75 1.62 1.61 1.59 1.58 1.55 1.55 1.54 1.53 1.51 1.09 1.07
5.12
4.19
2.89 2.86 2.84 2.80 2.78 2.76 2.75 2.73 2.69 2.68 2.67 2.66 2.65 2.64 2.63 1.75 1.62 1.61 1.59 1.58 1.55 1.55 1.54 1.53 1.51 1.09 1.07
2.00
2.00
2.02
1.22
1.12
2.47
2.31
6.26
5.0 4.5 4.0 3.5 3.0 2.5 2.0 1.5 1.0 ppm
4.75
2.00
2.00
2.02
1.22
1.12
2.47
2.31
6.26
15 14 13 12 11 10 9 8 7 6 5 4 3 2 1 0 -1 -2 ppm

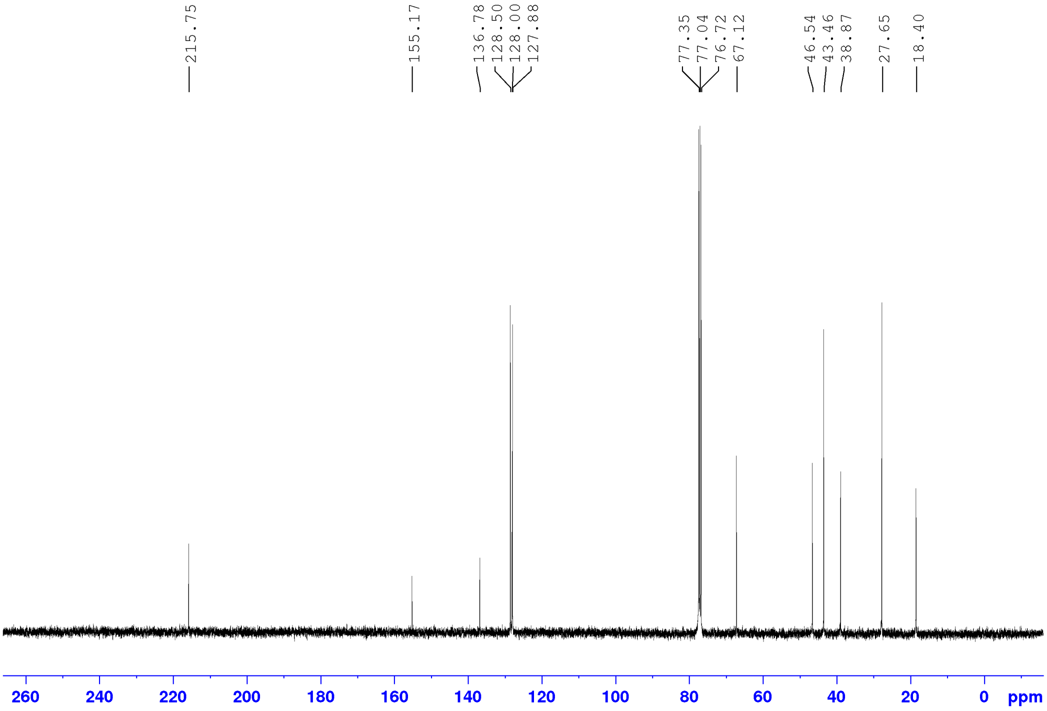
215.75
155.17
136.78 128.50 128.00 127.88
77.35 77.04 76.72 67.12
46.54 43.46 38.87
27.65
18.40
260 240 220 200 180 160 140 120 100 80 60 40 20 0 ppm

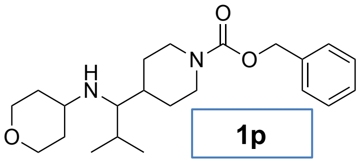

1p

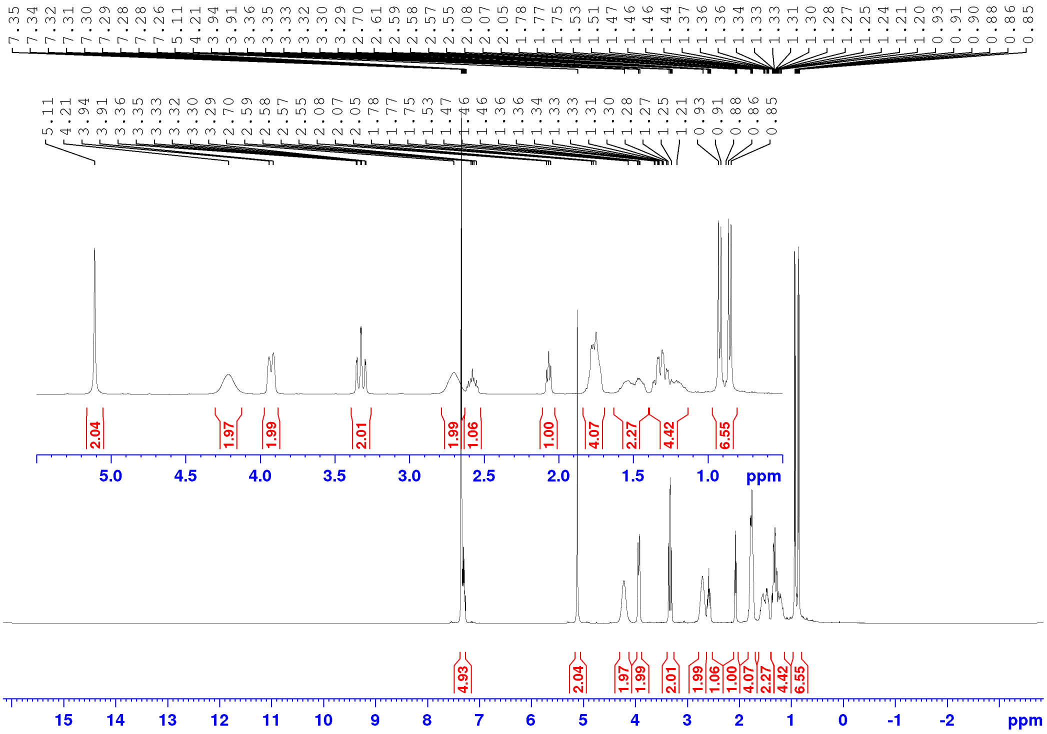
7.35 7.34 7.32 7.31 7.30 7.29 7.28 7.28 7.26 5.11 4.21 3.94 3.91 3.36 3.35 3.33 3.32 3.30 3.29 2.70 2.61 2.59 2.58 2.57 2.55 2.08 2.07 2.05 1.78 1.77 1.75 1.53 1.51 1.47 1.46 1.46 1.44 1.37 1.36 1.36 1.34 1.33 1.33 1.31 1.30 1.28 1.27 1.25 1.24 1.21 1.20 0.93 0.91 0.90 0.88 0.86 0.85
ppm

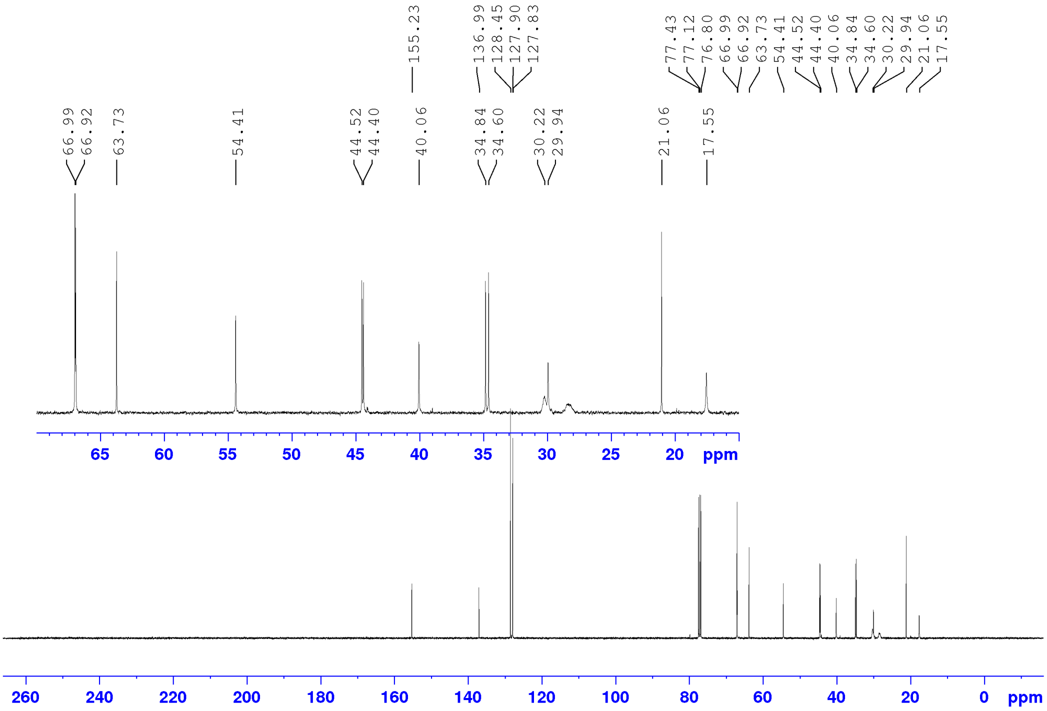
155.23 136.99 128.45 127.90 127.83 77.43 77.12 76.80 66.99 66.92 63.73 54.41 44.52 44.40 40.06 34.84 34.60 30.22 29.94 21.06 17.55
ppm

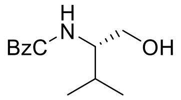
BzC
N
H
OH

1q-l

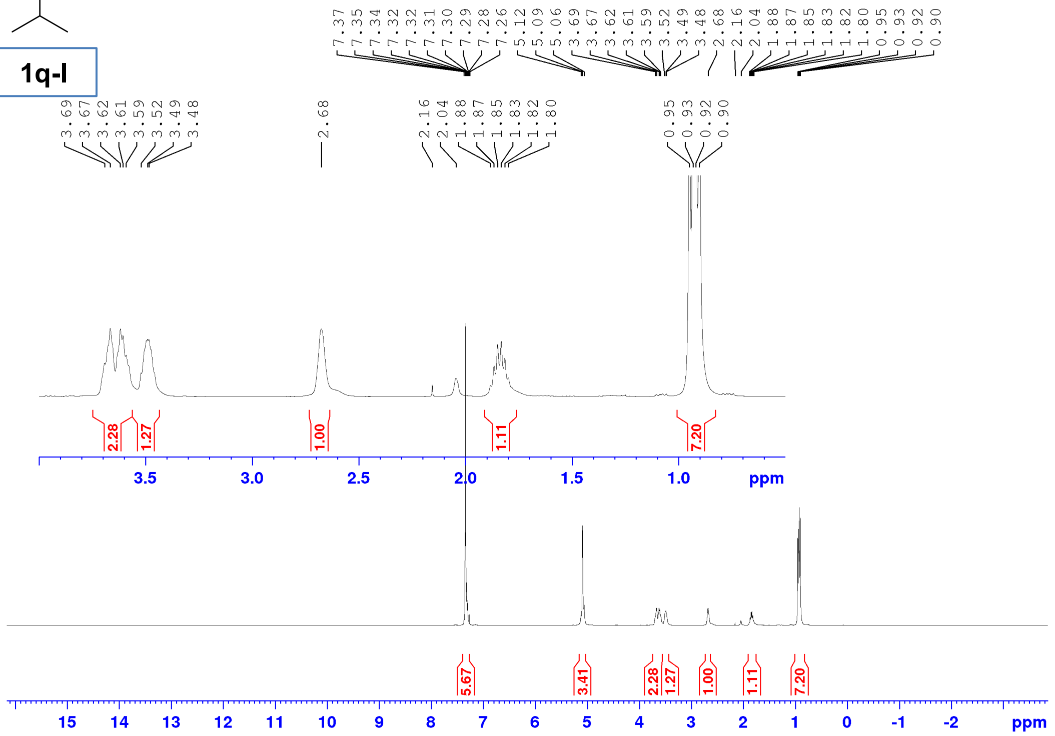
7.37 7.35 7.34 7.32 7.32 7.31 7.30 7.29 7.28 7.26 5.12 5.09 5.06 3.69 3.67 3.62 3.61 3.59 3.52 3.49 3.48 2.68 2.16 2.04 1.88 1.87 1.85 1.83 1.82 1.80 0.95 0.93 0.92 0.90
3.69 3.67 3.62 3.61 3.59 3.52 3.49 3.48
2.68
2.16 2.04 1.88 1.87 1.85 1.83 1.82 1.80
0.95 0.93 0.92 0.90
2.28 1.27 1.00 1.11 7.20
3.5 3.0 2.5 2.0 1.5 1.0 ppm
5.67 3.41 2.28 1.27 1.00 1.11 7.20
15 14 13 12 11 10 9 8 7 6 5 4 3 2 1 0 -1 -2 ppm

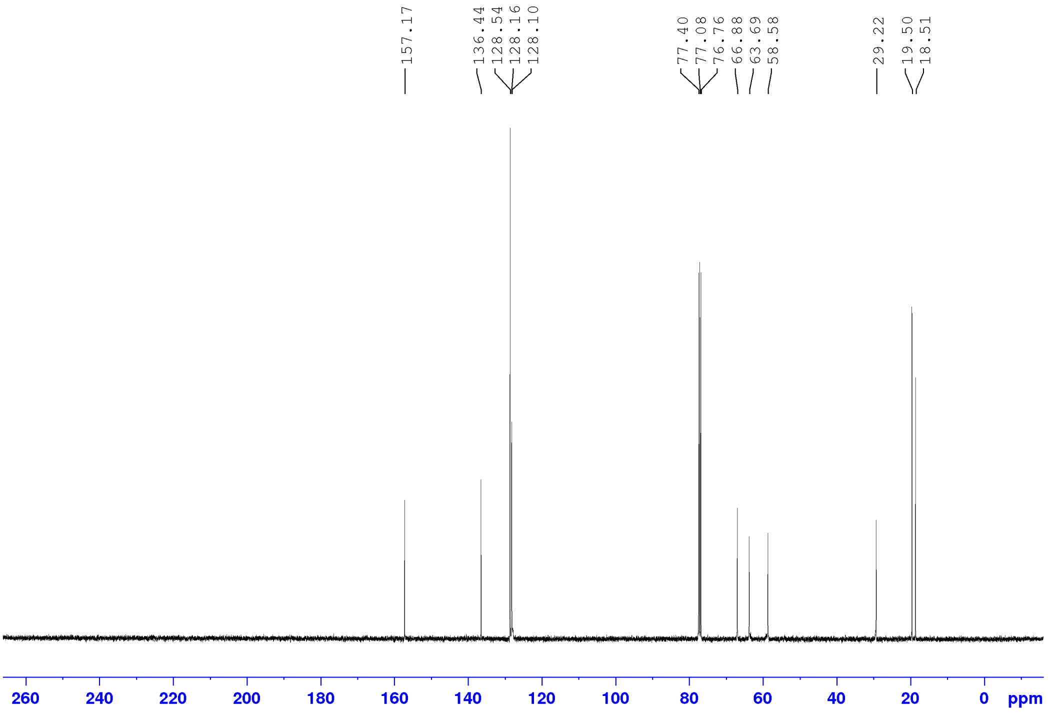
157.17
136.44 128.54 128.16 128.10
77.40 77.08 76.76 66.88 63.69 58.58
29.22 19.50 18.51
260 240 220 200 180 160 140 120 100 80 60 40 20 0 ppm

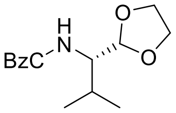

1q-II

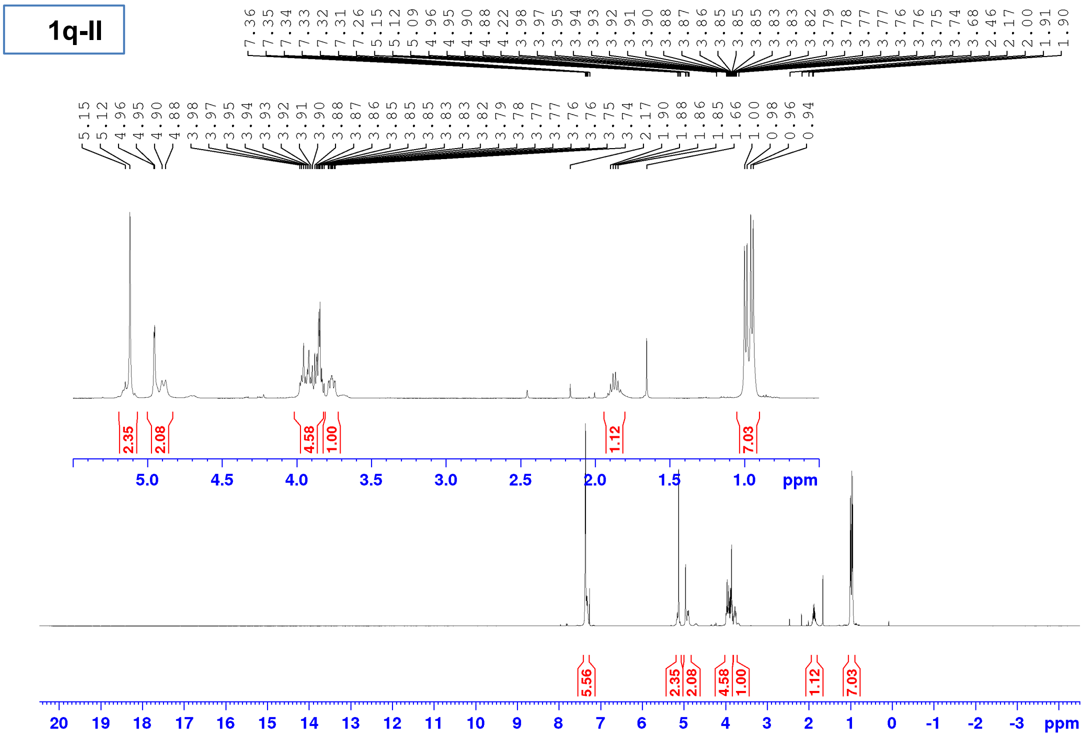

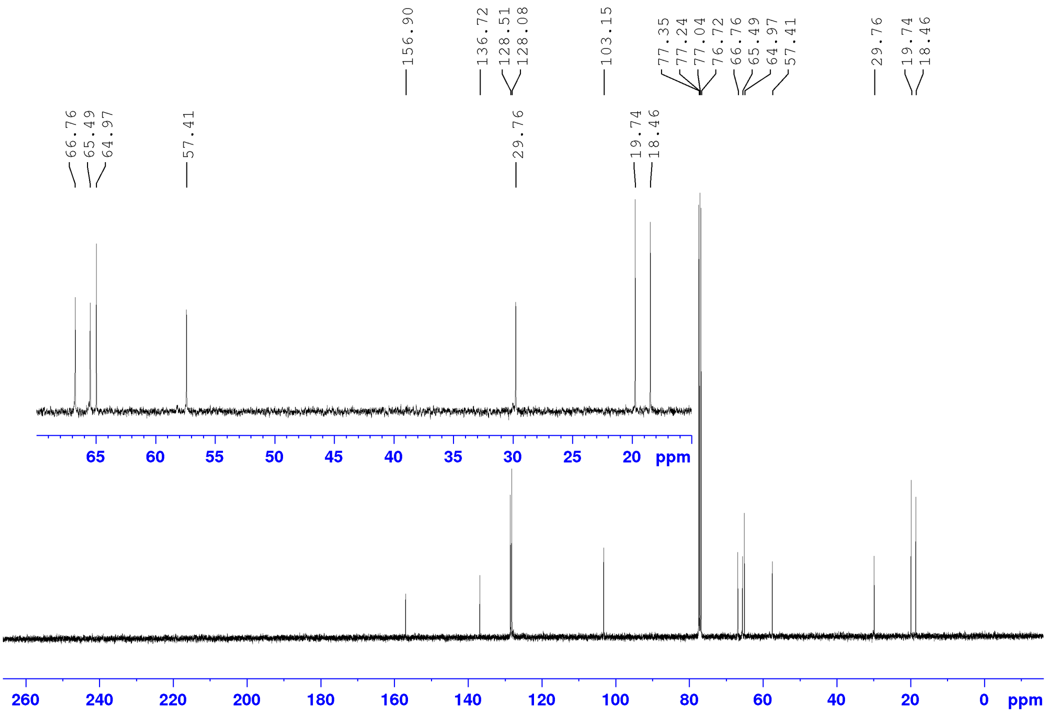

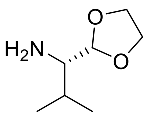
H2N
O
O

1q-III

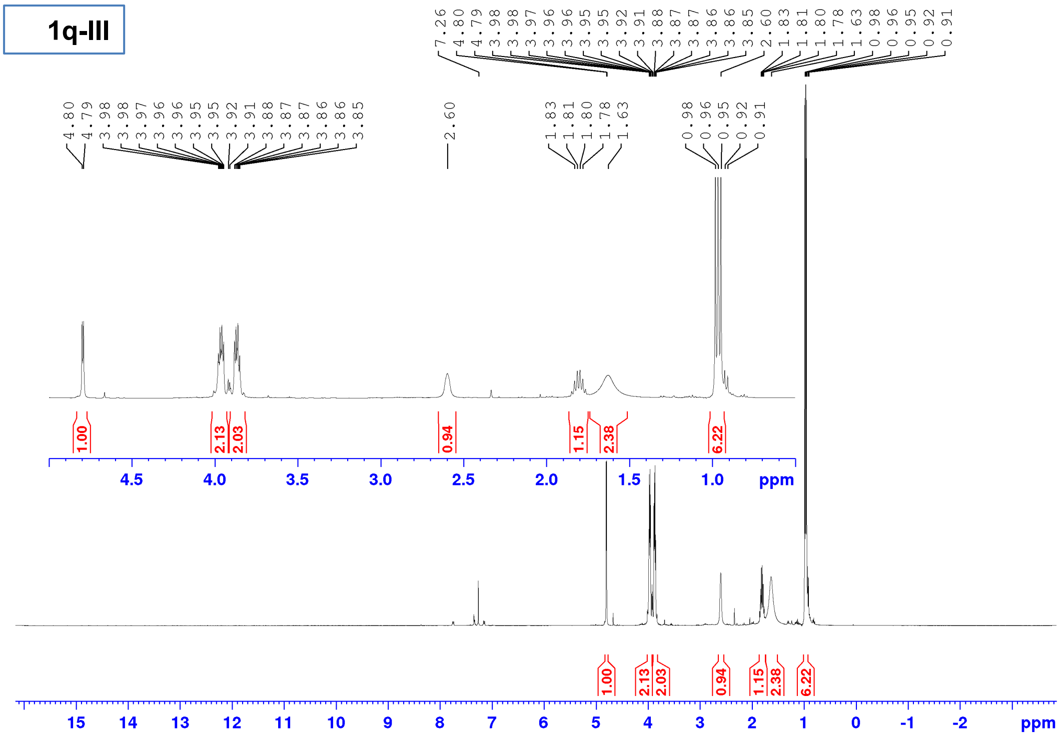
7.26
4.80
4.79
3.98
3.98
3.97
3.96
3.96
3.95
3.95
3.92
3.91
3.88
3.87
3.86
3.86
3.85
2.60
1.83
1.81
1.80
1.78
1.63
0.98
0.96
0.95
0.92
0.91
1.00
2.13
2.03
0.94
1.15
2.38
6.22
ppm

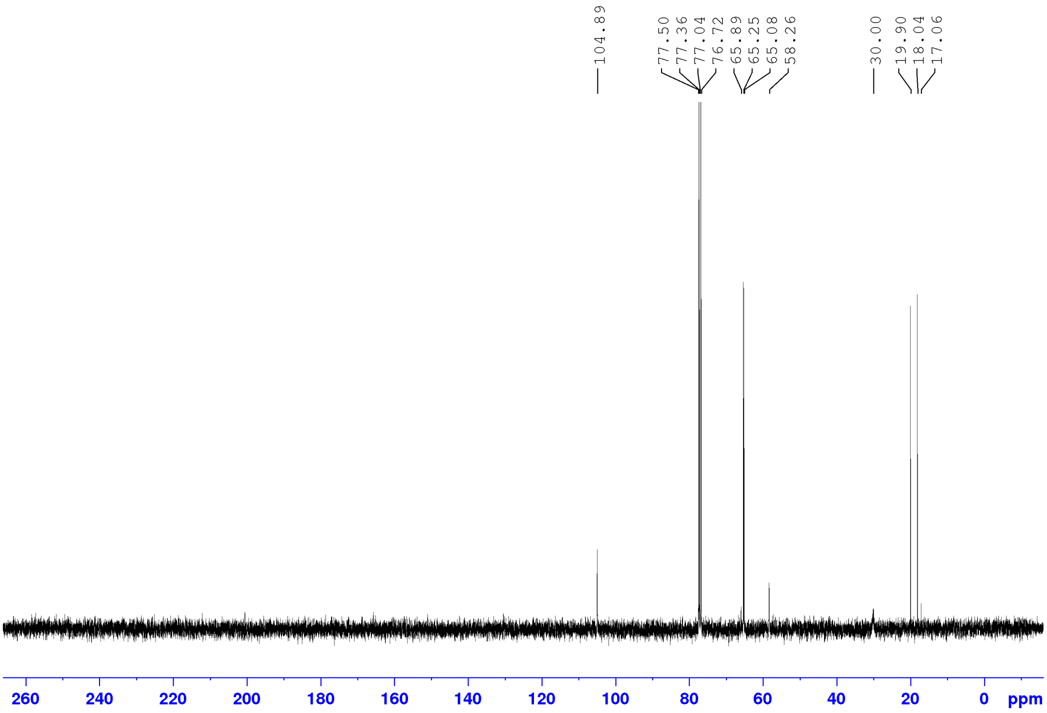
104.89
77.50
77.36
77.04
76.72
65.89
65.25
65.08
58.26
30.00
19.90
18.04
17.06
ppm

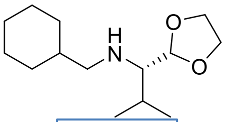

1q

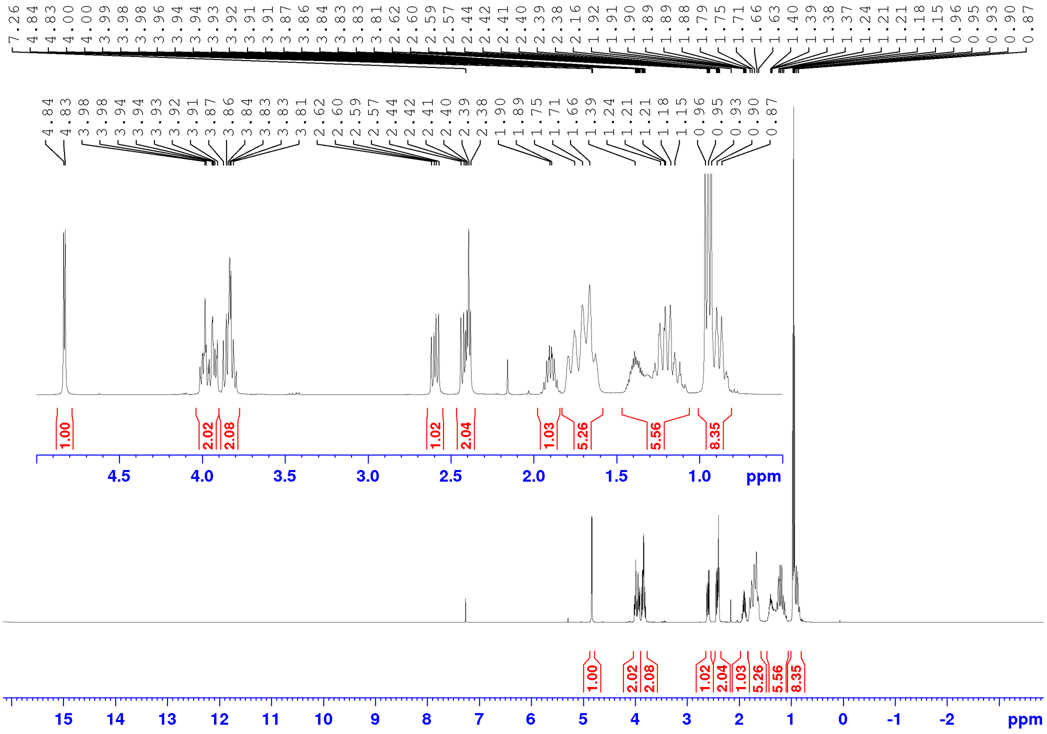

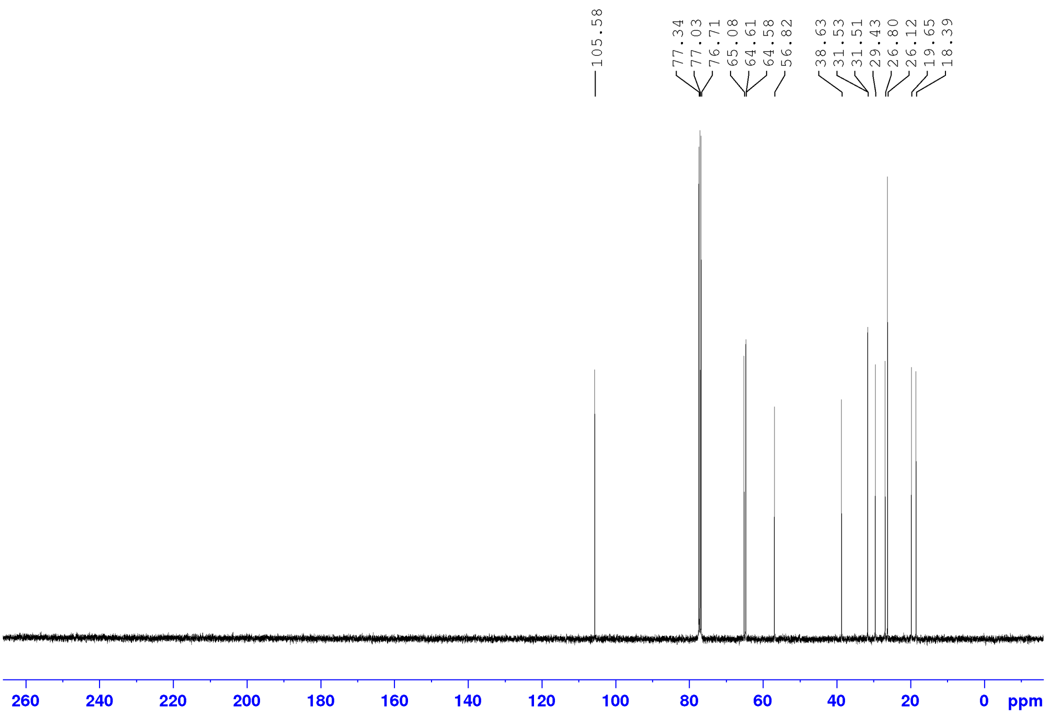

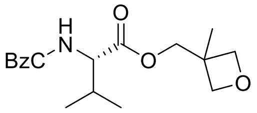
BzC
H
N
O
O
O

1r-I

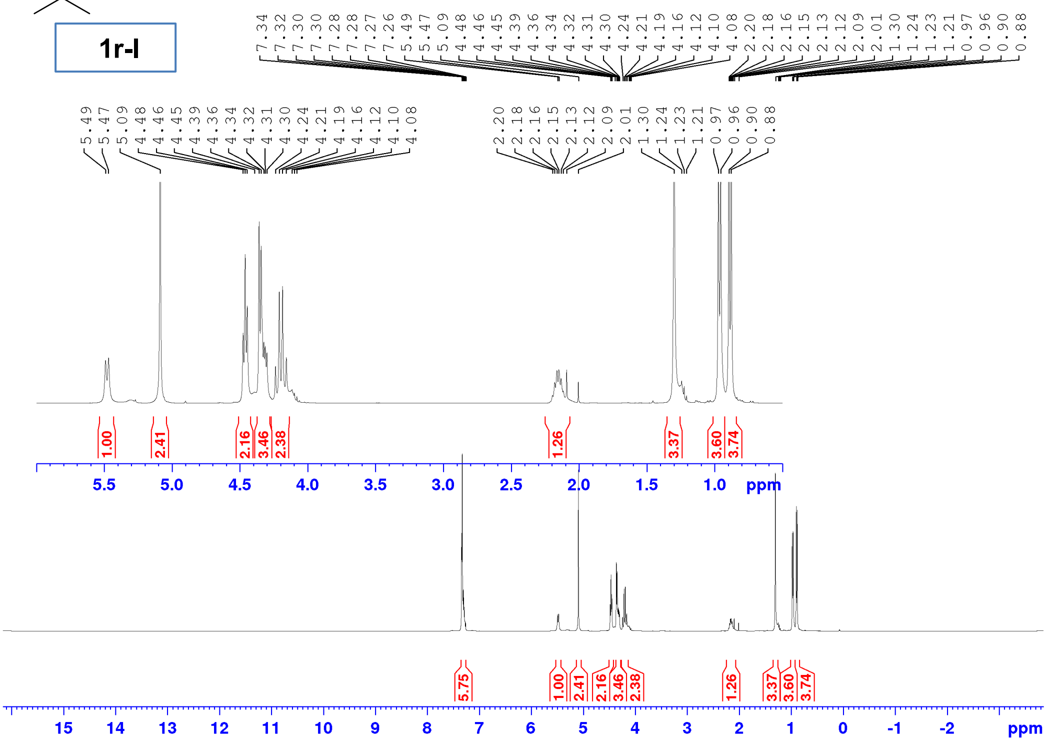
7.34 7.32 7.30 7.30 7.28 7.28 7.27 7.26 5.49 5.47 5.09 4.48 4.46 4.45 4.39 4.36 4.34 4.32 4.31 4.30 4.24 4.21 4.19 4.16 4.12 4.10 4.08 2.20 2.18 2.16 2.15 2.13 2.12 2.09 2.01 1.30 1.24 1.23 1.21 0.97 0.96 0.90 0.88
5.49 5.47 5.09 4.48 4.46 4.45 4.39 4.36 4.34 4.32 4.31 4.30 4.24 4.21 4.19 4.16 4.12 4.10 4.08
2.20 2.18 2.16 2.15 2.13 2.12 2.09 2.01 1.30 1.24 1.23 1.21 0.97 0.96 0.90 0.88
1.00 2.41 2.16 3.46 2.38 1.26 3.37 3.60 3.74
5.5 5.0 4.5 4.0 3.5 3.0 2.5 2.0 1.5 1.0 ppm
5.75 1.00 2.41 2.16 3.46 2.38 1.26 3.37 3.60 3.74
15 14 13 12 11 10 9 8 7 6 5 4 3 2 1 0 -1 -2 ppm

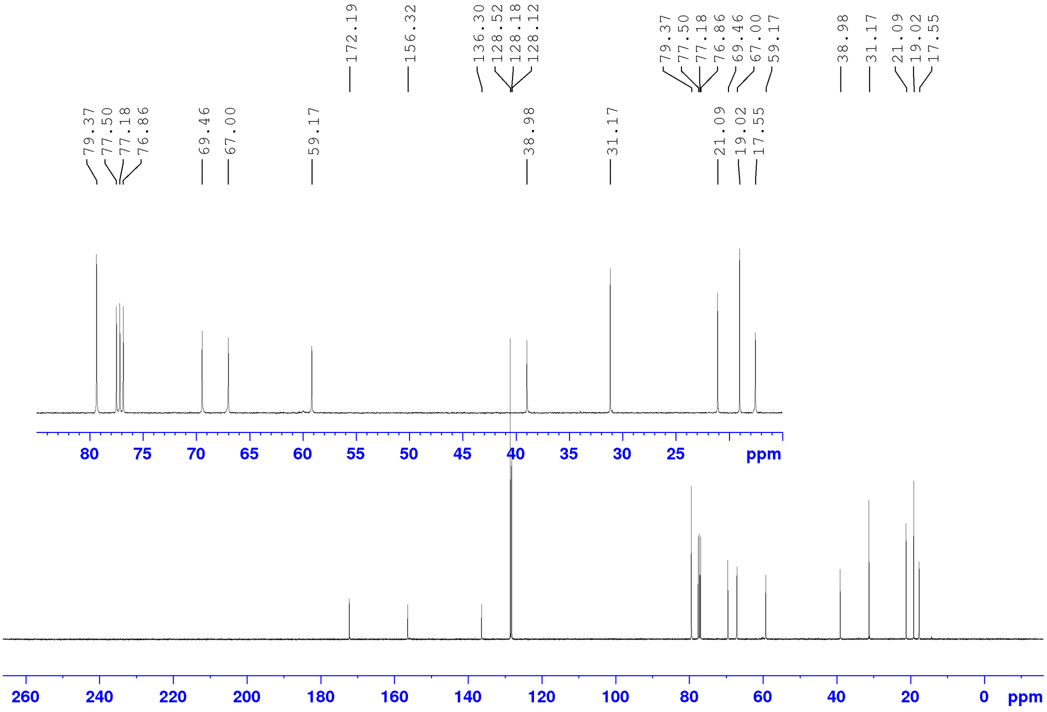
172.19 156.32 136.30 128.52 128.18 128.12 79.37 77.50 77.18 76.86 69.46 67.00 59.17 38.98 31.17 21.09 19.02 17.55
79.37 77.50 77.18 76.86 69.46 67.00 59.17 38.98 31.17 21.09 19.02 17.55
80 75 70 65 60 55 50 45 40 35 30 25 ppm
260 240 220 200 180 160 140 120 100 80 60 40 20 0 ppm

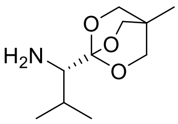
H2N
O
O
O

1r-II

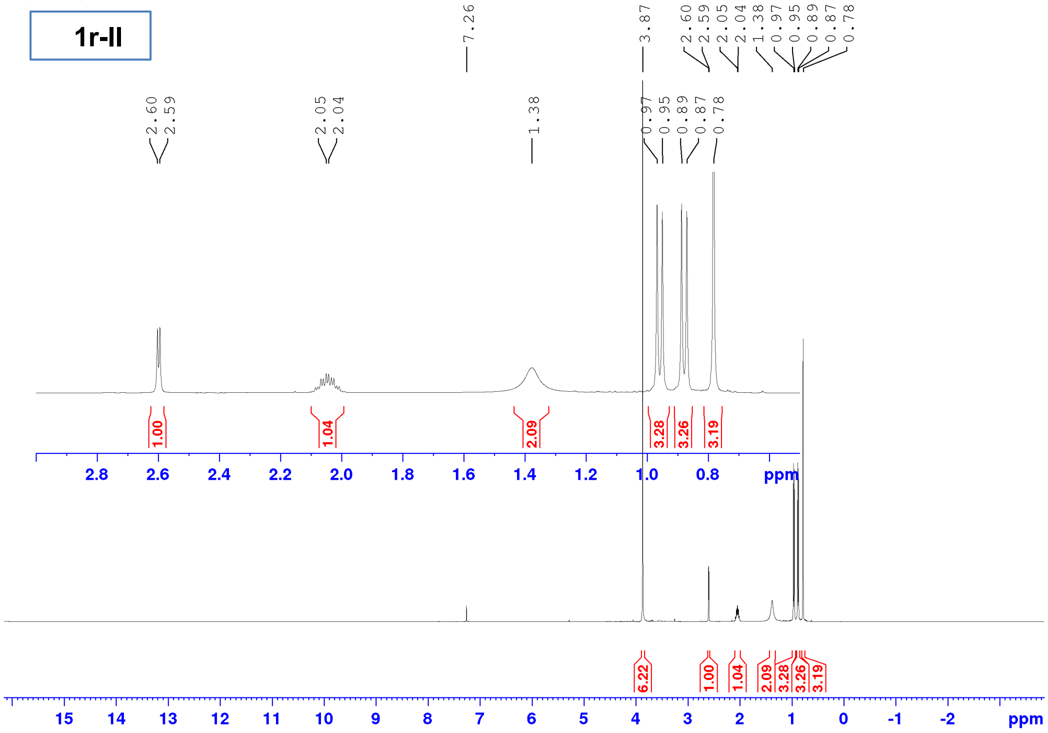
7.26
3.87
2.60
2.59
2.05
2.04
1.38
0.97
0.95
0.89
0.87
0.78
1.00
1.04
2.09
3.28
3.26
3.19
6.22
ppm

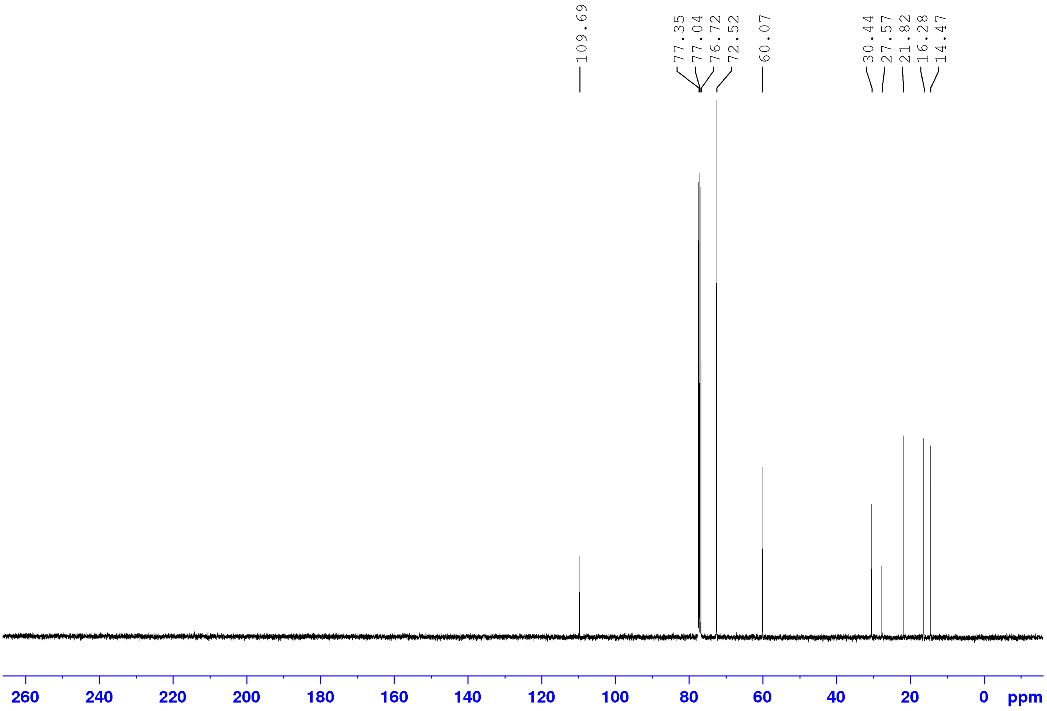
109.69
77.35
77.04
76.72
72.52
60.07
30.44
27.57
21.82
16.28
14.47
ppm

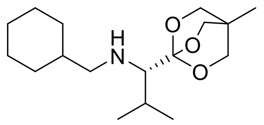

1r

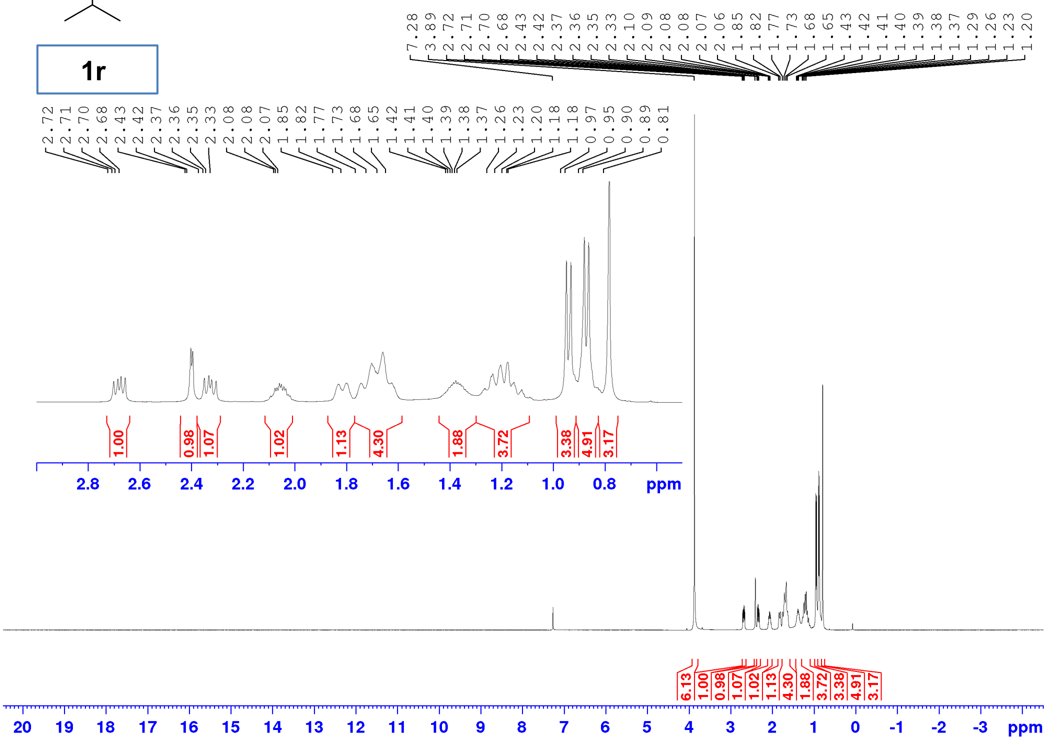

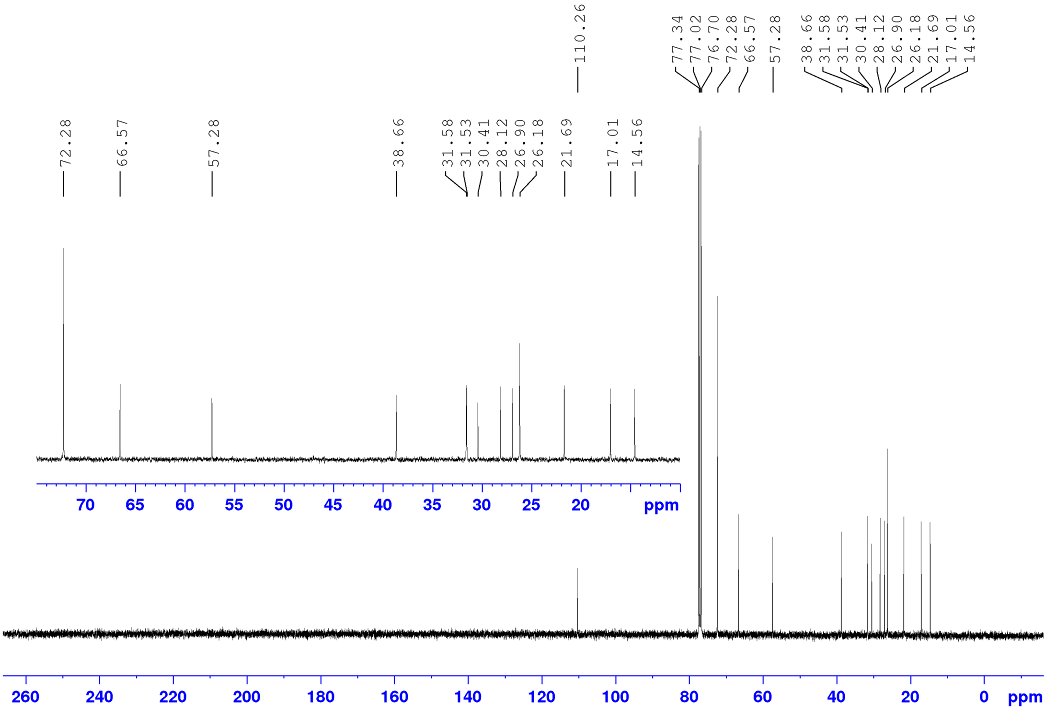

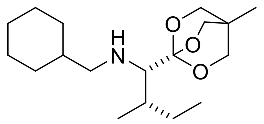

1s

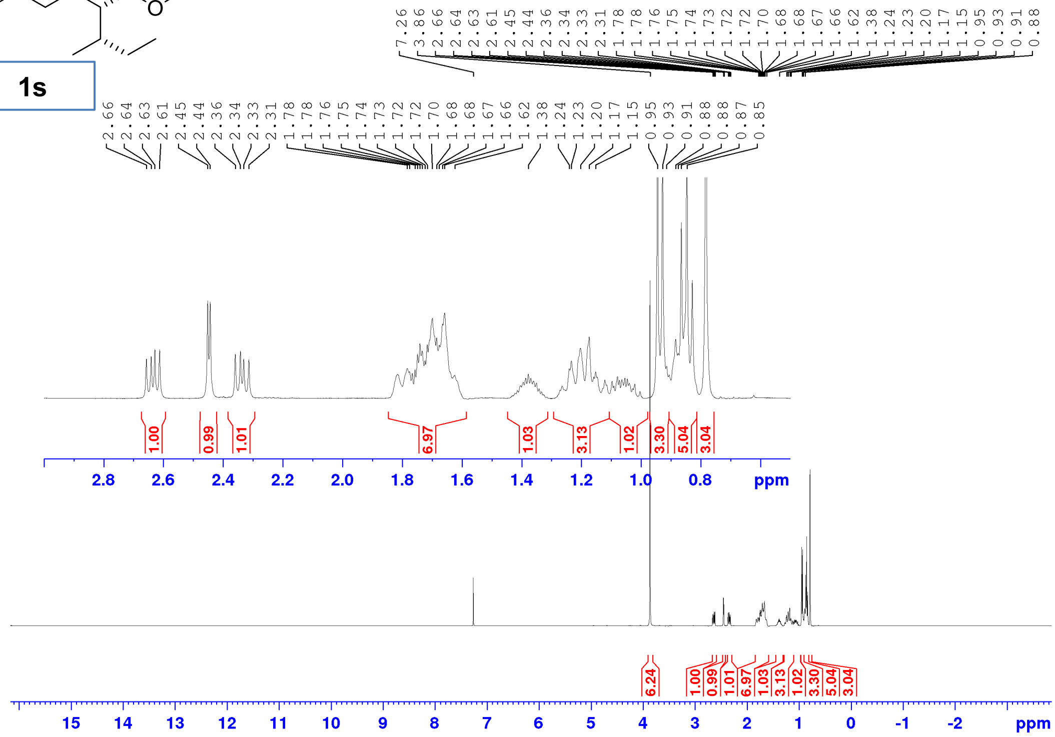

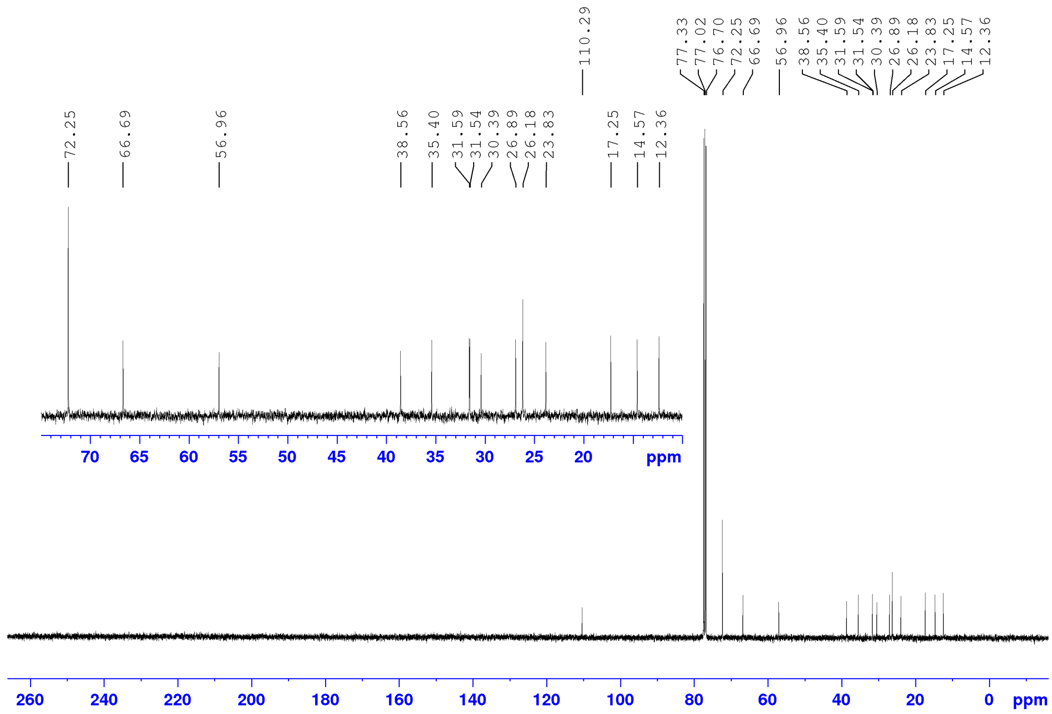

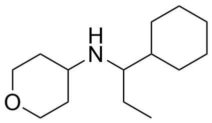

H
N
O

1t

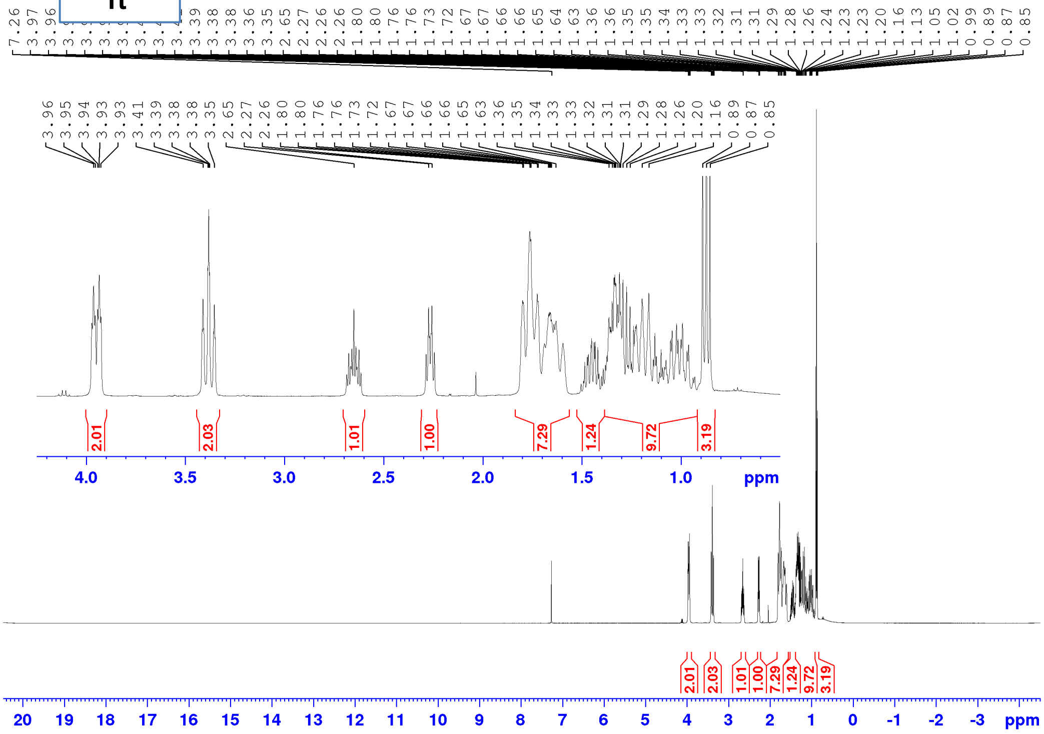

3.96 3.95 3.94 3.93 3.93 3.41 3.39 3.38 3.38 3.35 2.65 2.27 2.26 1.80 1.80 1.76 1.76 1.73 1.72 1.67 1.67 1.66 1.66 1.65 1.63 1.36 1.35 1.34 1.33 1.33 1.32 1.31 1.31 1.29 1.28 1.26 1.20 1.16 0.89 0.87 0.85
2.01 2.03 1.01 1.00 7.29 1.24 9.72 3.19
ppm

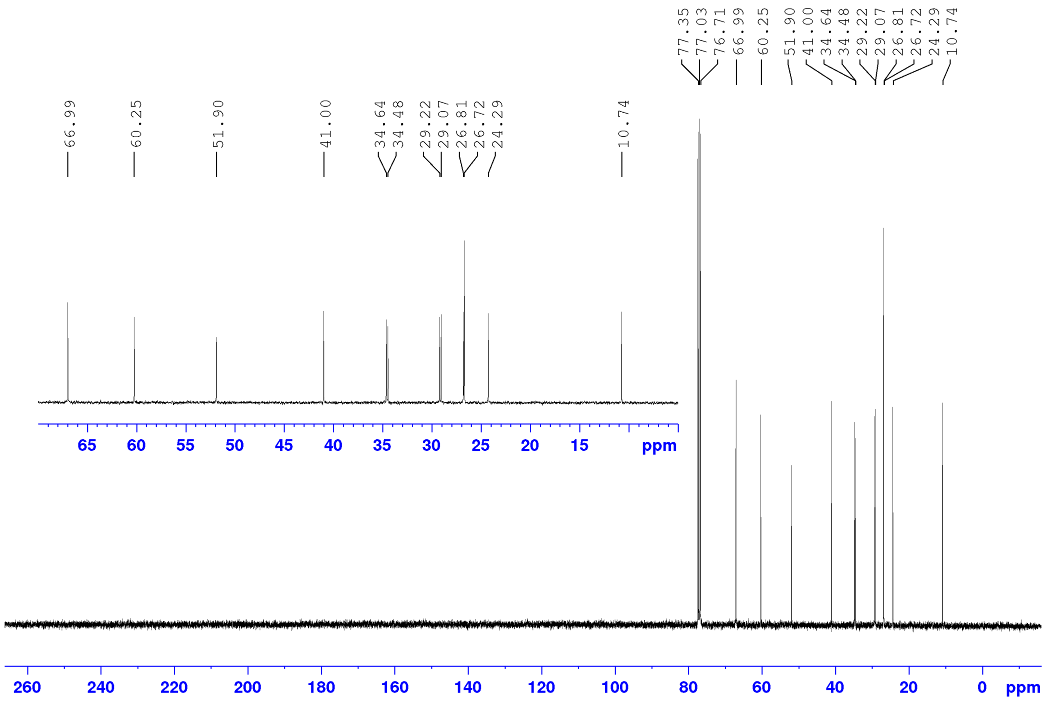

77.35 77.03 76.71 66.99 60.25 51.90 41.00 34.64 34.48 29.22 29.07 26.81 26.72 24.29 10.74
ppm

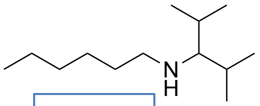

1u

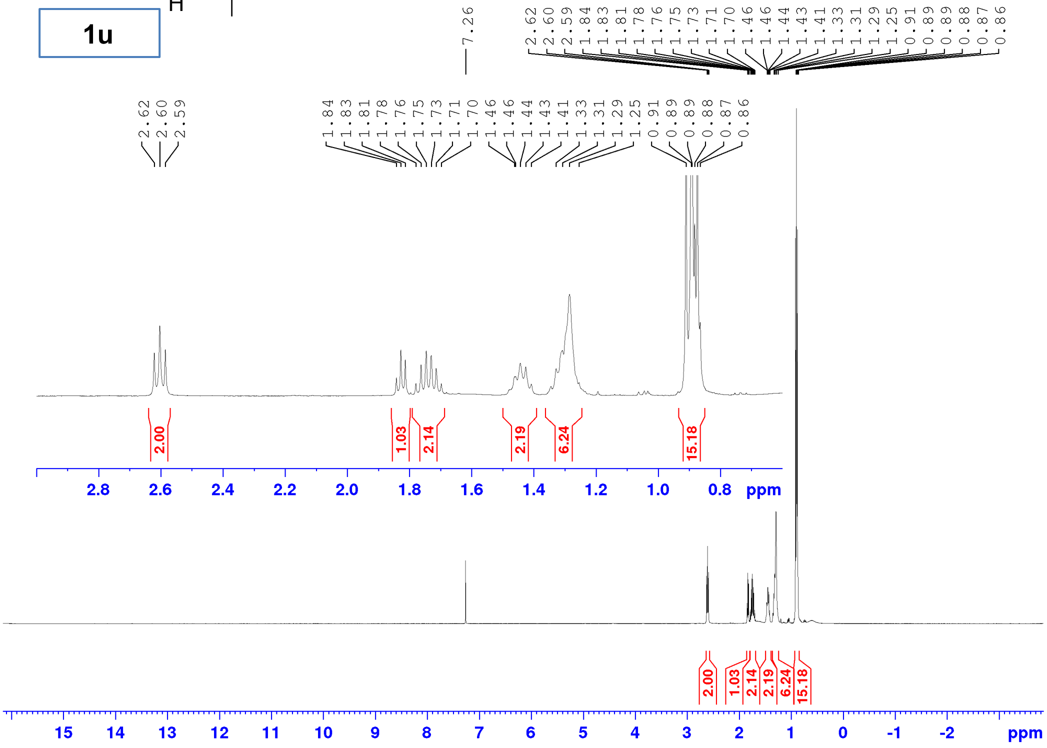
7.26
2.62
2.60
2.59
1.84
1.83
1.81
1.78
1.76
1.75
1.73
1.71
1.70
1.46
1.44
1.43
1.41
1.33
1.31
1.29
1.25
0.91
0.89
0.88
0.87
0.86
2.00
1.03
2.14
2.19
6.24
15.18
ppm

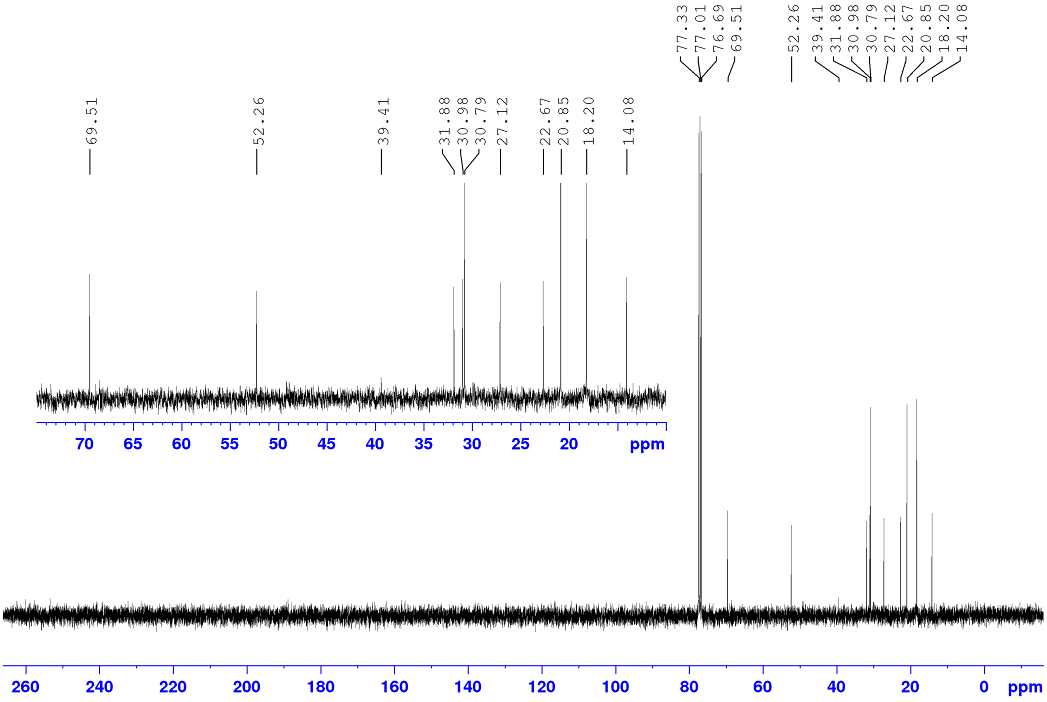
77.33
77.01
76.69
69.51
52.26
39.41
31.88
30.98
30.79
27.12
22.67
20.85
18.20
14.08
ppm

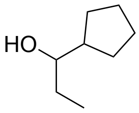

1v-l

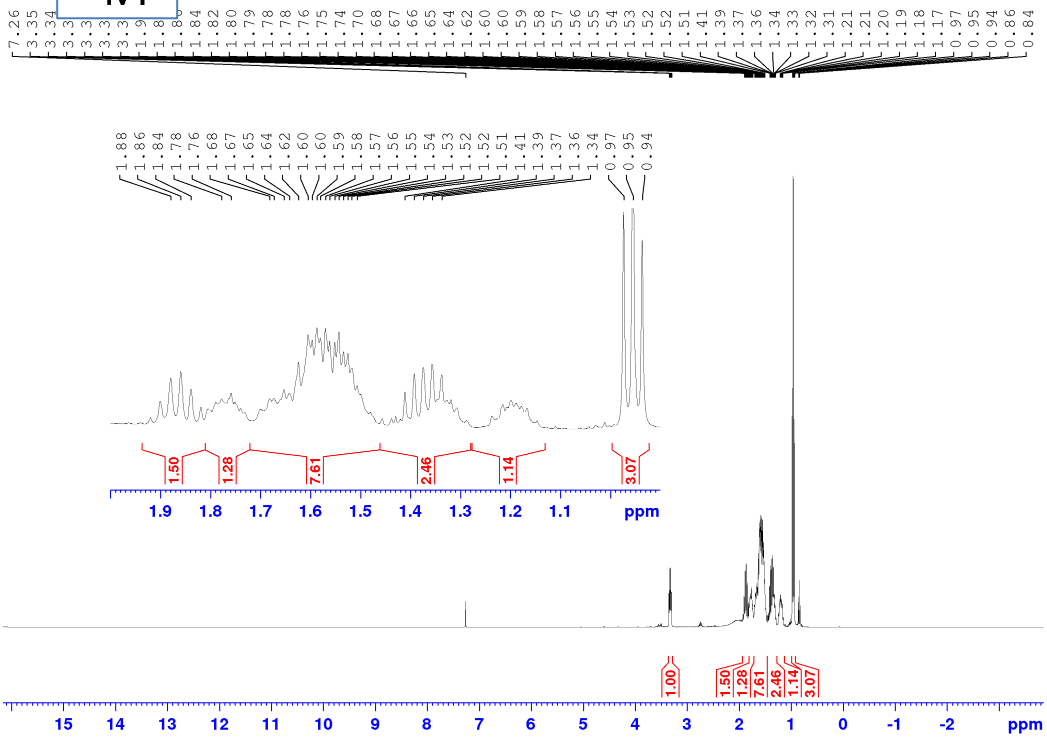

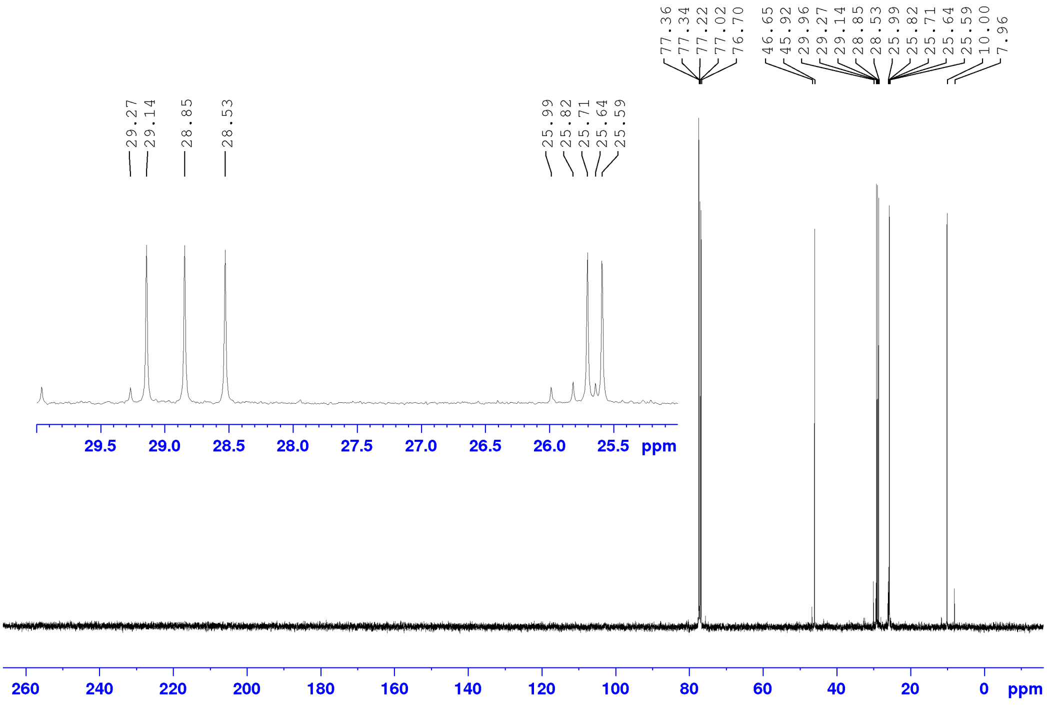

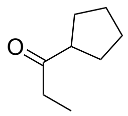

1v-II

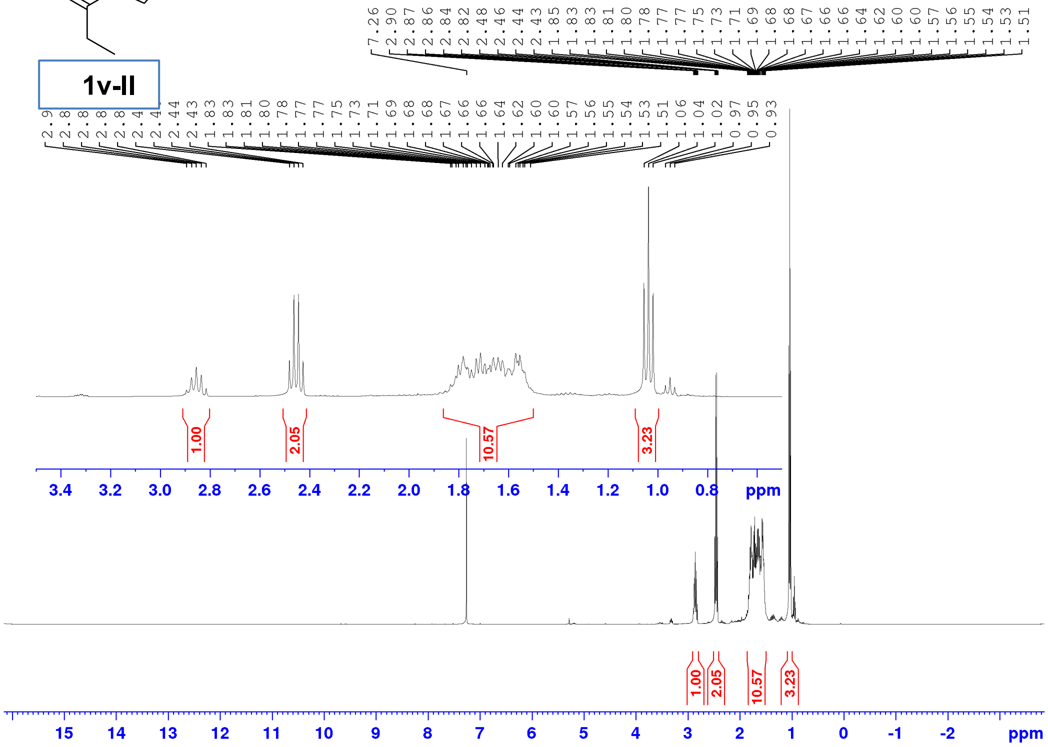
7.26
2.90
2.87
2.86
2.84
2.82
2.48
2.46
2.44
2.43
1.85
1.83
1.83
1.81
1.80
1.78
1.77
1.77
1.75
1.73
1.71
1.69
1.68
1.68
1.67
1.66
1.66
1.64
1.62
1.60
1.60
1.57
1.56
1.55
1.54
1.53
1.51
1.06
1.04
1.02
0.97
0.95
0.93
1.00
2.05
10.57
3.23
3.4 3.2 3.0 2.8 2.6 2.4 2.2 2.0 1.8 1.6 1.4 1.2 1.0 0.8 ppm
15 14 13 12 11 10 9 8 7 6 5 4 3 2 1 0 -1 -2 ppm

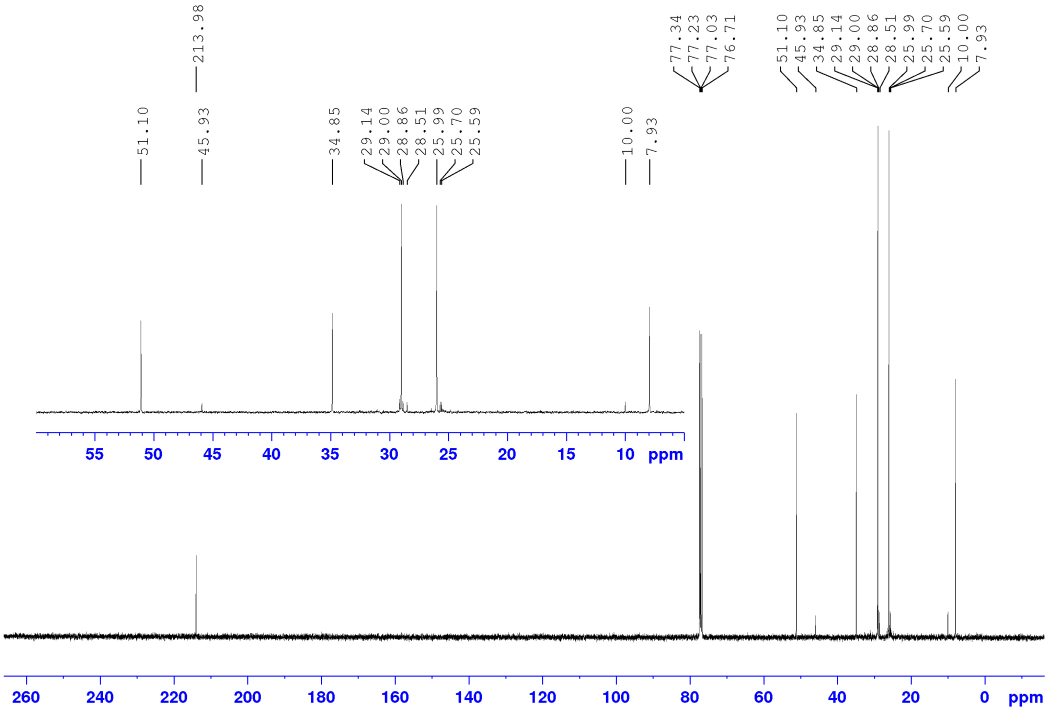
213.98
77.34
77.23
77.03
76.71
51.10
45.93
34.85
29.14
29.00
28.86
28.51
25.99
25.70
25.59
10.00
7.93
55 50 45 40 35 30 25 20 15 10 ppm
260 240 220 200 180 160 140 120 100 80 60 40 20 0 ppm

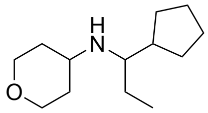
H
N
O

1v

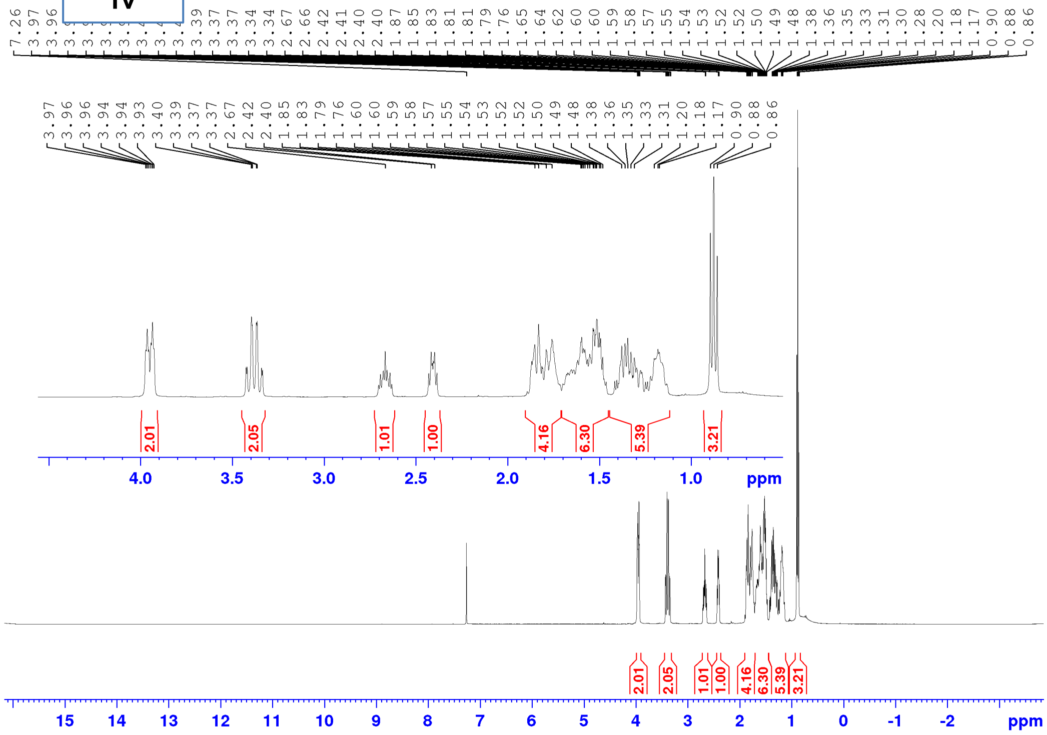
7.26
3.97
3.96
3.39
3.37
3.37
3.34
3.34
2.67
2.66
2.42
2.41
2.40
2.40
1.87
1.85
1.83
1.81
1.81
1.79
1.76
1.65
1.64
1.62
1.60
1.60
1.59
1.58
1.57
1.55
1.54
1.53
1.52
1.52
1.50
1.49
1.48
1.38
1.36
1.35
1.33
1.31
1.30
1.28
1.20
1.18
1.17
0.90
0.88
0.86
3.97
3.96
3.96
3.94
3.94
3.93
3.40
3.39
3.37
3.37
2.67
2.42
2.40
1.85
1.83
1.79
1.76
1.60
1.59
1.58
1.57
1.55
1.54
1.53
1.52
1.50
1.49
1.48
1.38
1.36
1.35
1.33
1.31
1.20
1.18
1.17
0.90
0.88
0.86
2.01
2.05
1.01
1.00
4.16
6.30
5.39
3.21
4.0
3.5
3.0
2.5
2.0
1.5
1.0
ppm
15
14
13
12
11
10
9
8
7
6
5
4
3
2
1
0
-1
-2
ppm

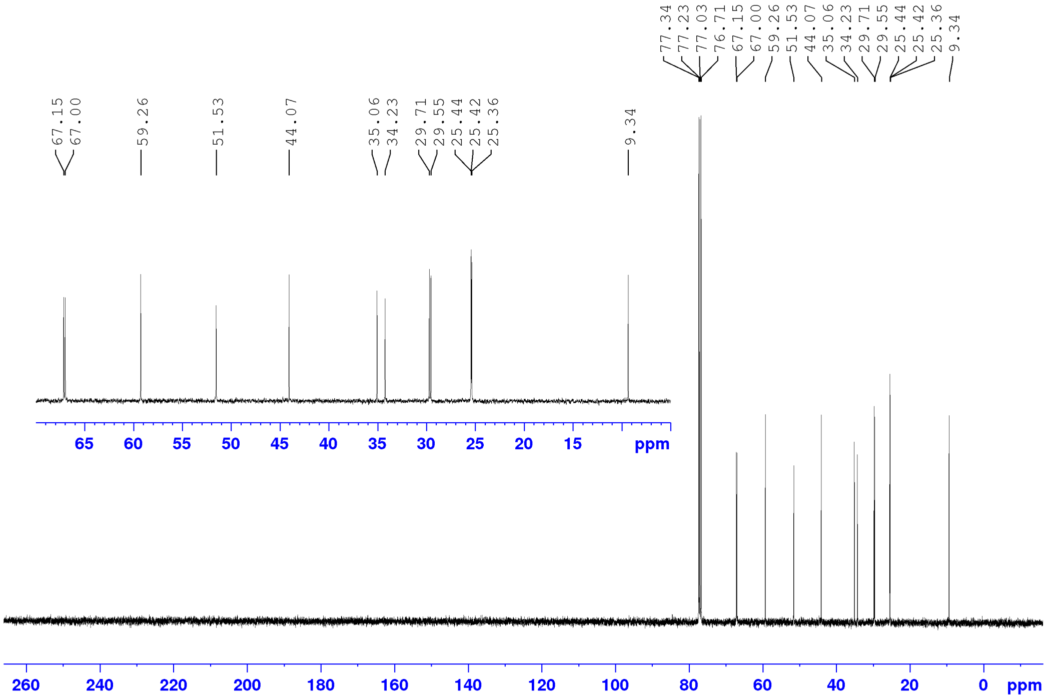
77.34
77.23
77.03
76.71
67.15
67.00
59.26
51.53
44.07
35.06
34.23
29.71
29.55
25.44
25.42
25.36
9.34
67.15
67.00
59.26
51.53
44.07
35.06
34.23
29.71
29.55
25.44
25.42
25.36
9.34
65
60
55
50
45
40
35
30
25
20
15
ppm
260
240
220
200
180
160
140
120
100
80
60
40
20
0
ppm

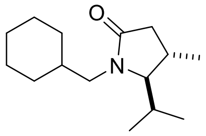

4a

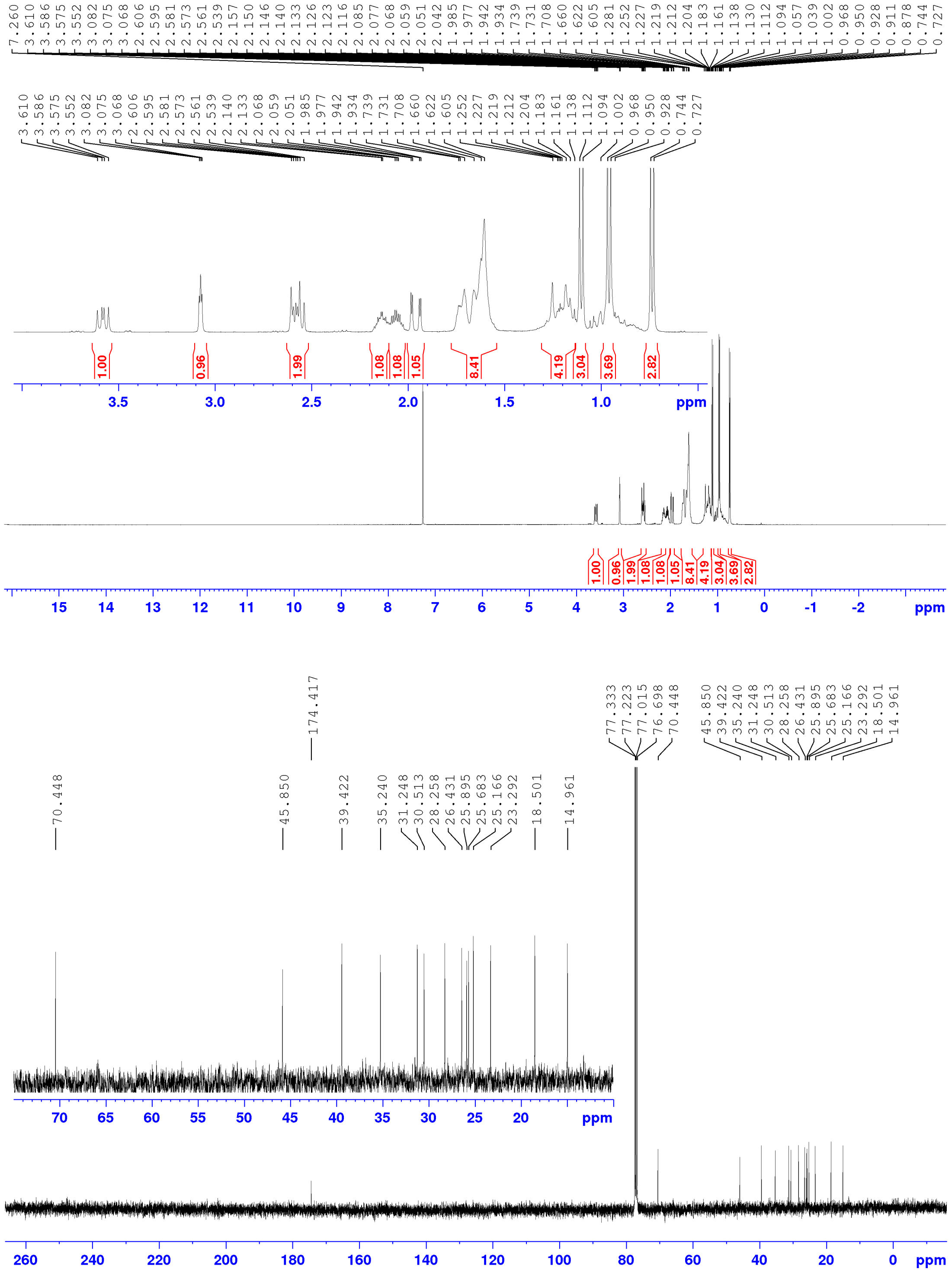
7.260 3.610 3.586 3.575 3.552 3.082 3.075 3.068 2.606 2.595 2.581 2.573 2.561 2.539 2.157 2.150 2.146 2.140 2.133 2.126 2.123 2.116 2.085 2.077 2.068 2.059 2.051 2.042 1.985 1.977 1.942 1.934 1.739 1.731 1.708 1.660 1.622 1.605 1.281 1.252 1.227 1.219 1.212 1.204 1.183 1.161 1.138 1.130 1.112 1.094 1.057 1.039 1.002 0.968 0.950 0.928 0.911 0.878 0.744 0.727
1.00 0.96 1.99 1.08 1.08 1.05 8.41 4.19 3.04 3.69 2.82
ppm
174.417
77.333 77.223 77.015 76.698 70.448
45.850 39.422 35.240 31.248 30.513 28.258 26.431 25.895 25.683 25.166 23.292 18.501 14.961
ppm

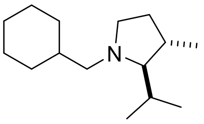

7

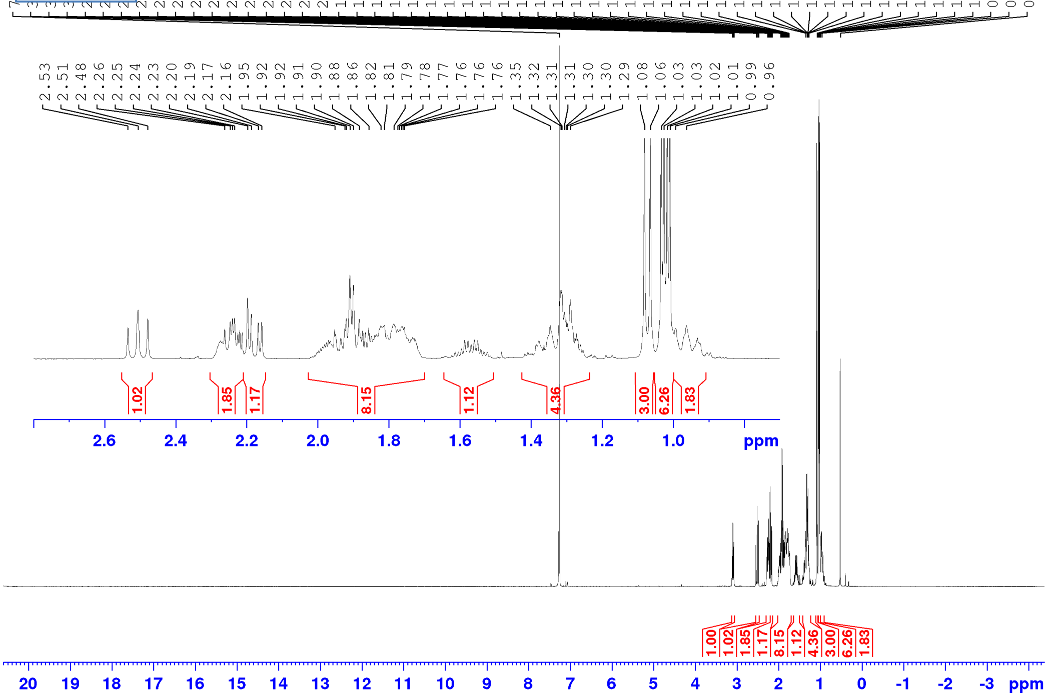
2.53 2.51 2.48 2.26 2.25 2.24 2.23 2.20 2.19 2.17 2.16 1.95 1.92 1.92 1.91 1.90 1.88 1.86 1.82 1.81 1.79 1.78 1.77 1.76 1.76 1.76 1.35 1.32 1.31 1.31 1.30 1.30 1.29 1.08 1.06 1.03 1.03 1.02 1.01 0.99 0.96
1.02 1.85 1.17 8.15 1.12 4.36 3.00 6.26 1.83
2.6 2.4 2.2 2.0 1.8 1.6 1.4 1.2 1.0 ppm
1.00 1.02 1.85 1.17 8.15 1.12 4.36 3.00 6.26 1.83
20 19 18 17 16 15 14 13 12 11 10 9 8 7 6 5 4 3 2 1 0 -1 -2 -3 ppm

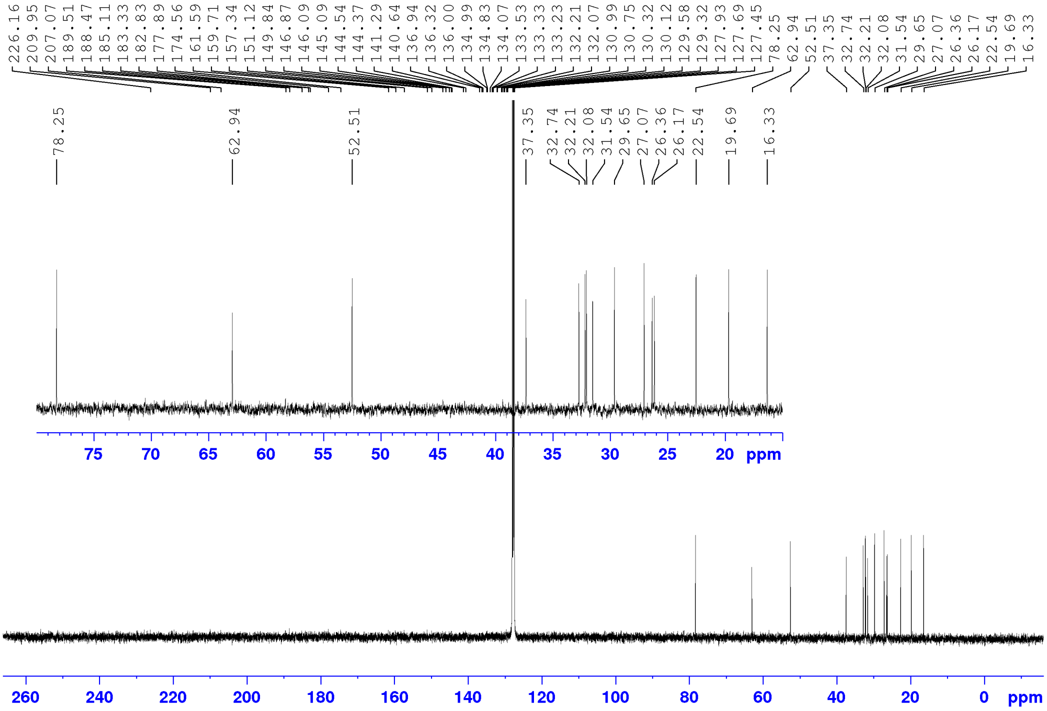
78.25 62.94 52.51 37.35 32.74 32.21 32.08 31.54 29.65 27.07 26.36 26.17 22.54 19.69 16.33
75 70 65 60 55 50 45 40 35 30 25 20 ppm
260 240 220 200 180 160 140 120 100 80 60 40 20 0 ppm

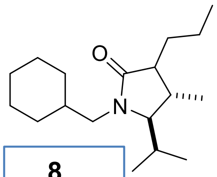

8

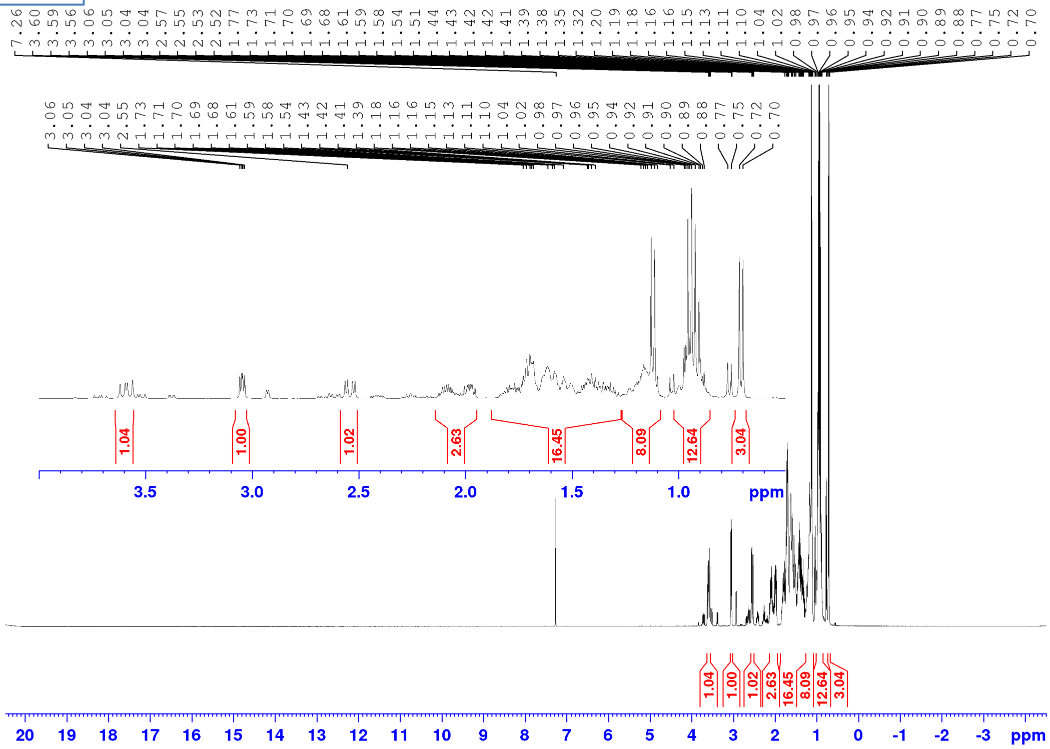
7.26 3.60 3.59 3.56 3.06 3.05 3.04 3.04 2.57 2.55 2.53 2.52 1.77 1.73 1.71 1.70 1.69 1.68 1.61 1.59 1.58 1.54 1.51 1.44 1.43 1.42 1.42 1.41 1.39 1.38 1.35 1.32 1.20 1.19 1.18 1.16 1.16 1.15 1.13 1.11 1.10 1.04 1.02 0.98 0.97 0.96 0.95 0.94 0.92 0.91 0.90 0.89 0.88 0.77 0.75 0.72 0.70
3.06 3.05 3.04 3.04 2.55 1.73 1.71 1.70 1.69 1.68 1.61 1.59 1.58 1.54 1.43 1.42 1.41 1.39 1.18 1.16 1.16 1.15 1.13 1.11 1.10 1.04 1.02 0.98 0.97 0.96 0.95 0.94 0.92 0.91 0.90 0.89 0.88 0.77 0.75 0.72 0.70
1.04
1.00
1.02
2.63
16.45
8.09
12.64
3.04
3.5 3.0 2.5 2.0 1.5 1.0 ppm
20 19 18 17 16 15 14 13 12 11 10 9 8 7 6 5 4 3 2 1 0 -1 -2 -3 ppm

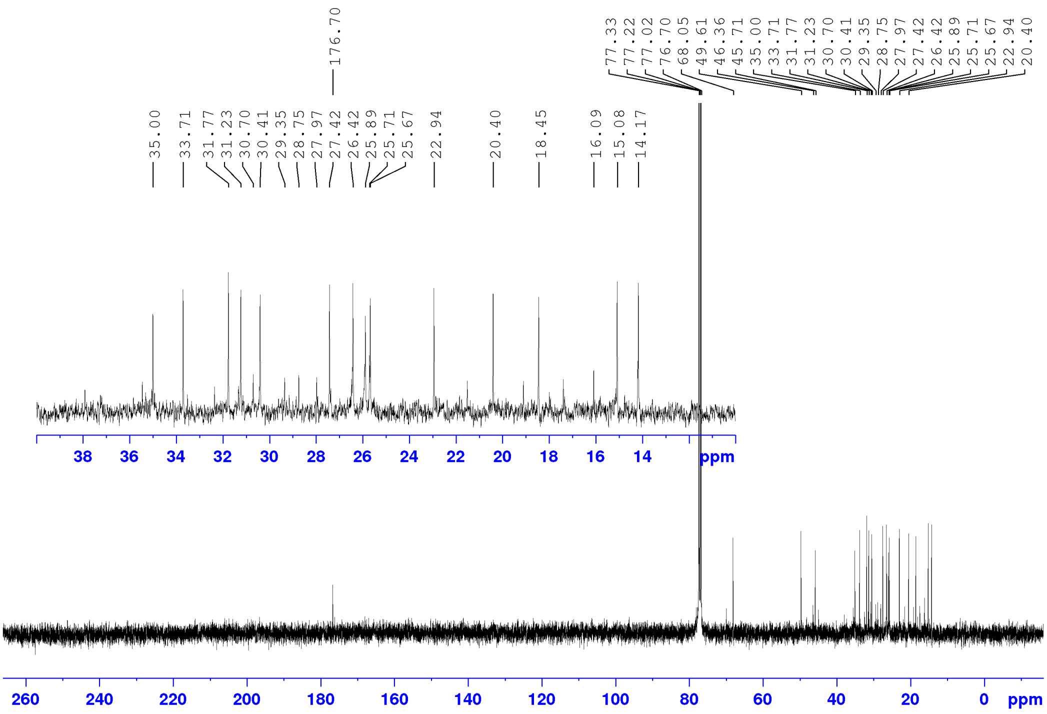
176.70
77.33 77.22 77.02 76.70 68.05 49.61 46.36 45.71 35.00 33.71 31.77 31.23 30.70 30.41 29.35 28.75 27.97 27.42 26.42 25.89 25.71 25.67 22.94 20.40
35.00 33.71 31.77 31.23 30.70 30.41 29.35 28.75 27.97 27.42 26.42 25.89 25.71 25.67 22.94 20.40 18.45 16.09 15.08 14.17
38 36 34 32 30 28 26 24 22 20 18 16 14 ppm
260 240 220 200 180 160 140 120 100 80 60 40 20 0 ppm

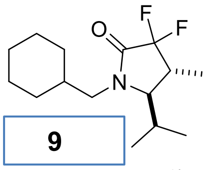

9

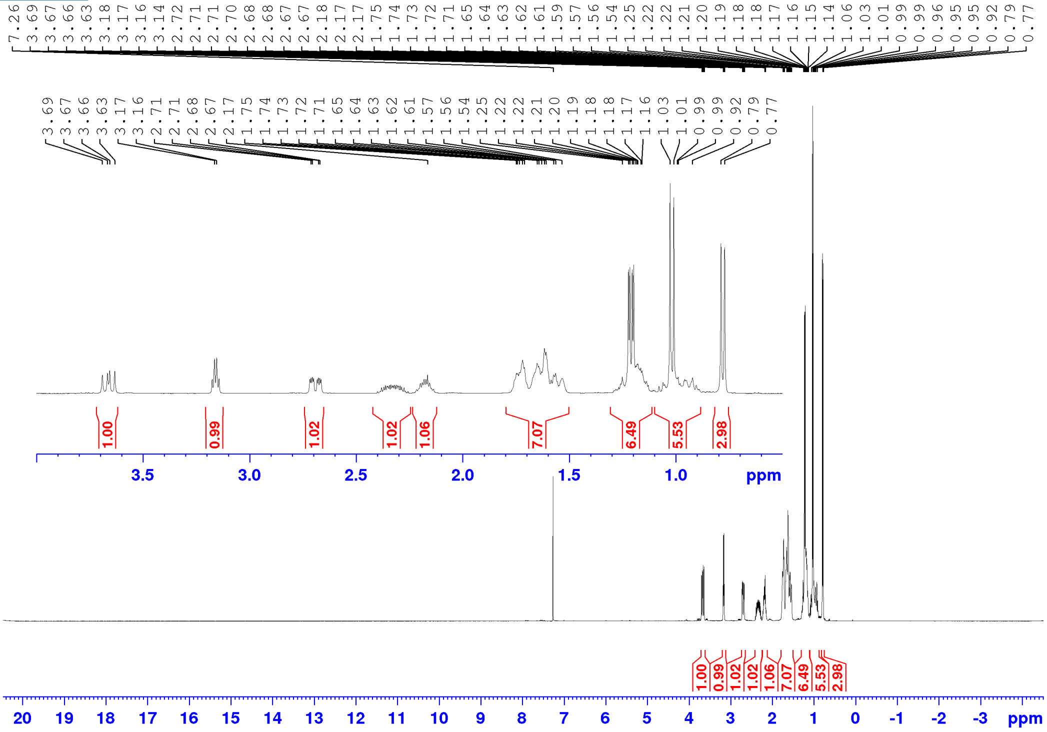
7.26 3.69 3.67 3.66 3.63 3.18 3.17 3.16 3.14 2.72 2.71 2.71 2.70 2.68 2.68 2.67 2.67 2.18 2.17 2.17 1.75 1.74 1.73 1.72 1.71 1.65 1.64 1.63 1.62 1.61 1.59 1.57 1.56 1.54 1.25 1.22 1.22 1.21 1.20 1.19 1.18 1.18 1.17 1.16 1.15 1.14 1.06 1.03 1.01 0.99 0.99 0.96 0.95 0.92 0.79 0.77
1.00 0.99 1.02 1.02 1.06 7.07 6.49 5.53 2.98
ppm

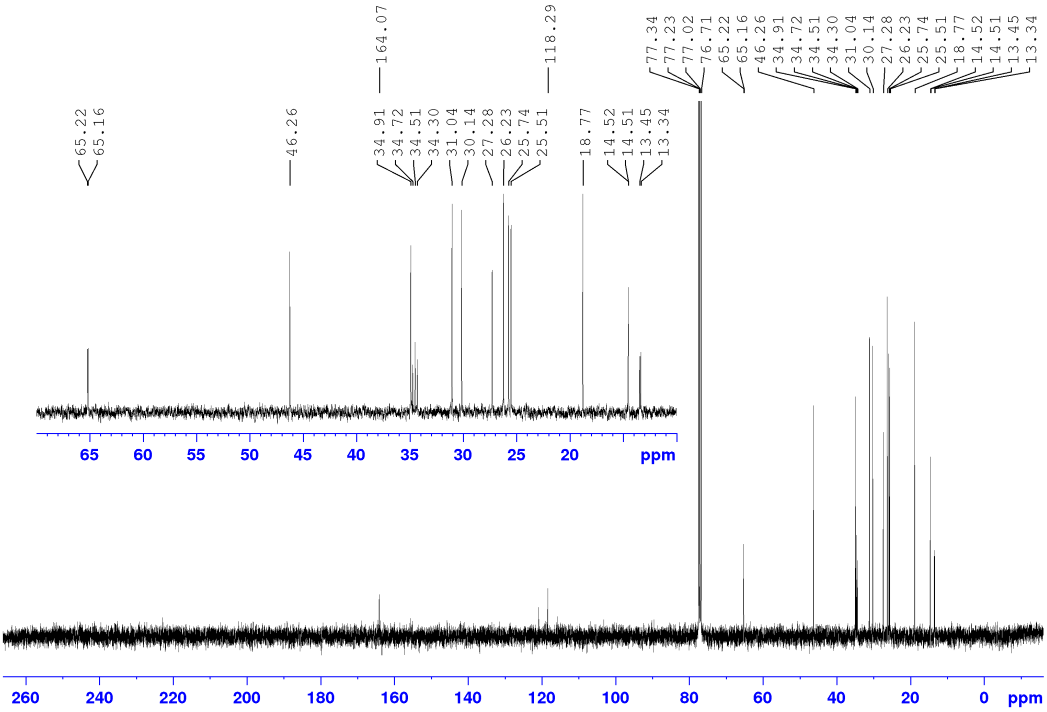
164.07 118.29 77.34 77.23 77.02 76.71 65.22 65.16 46.26 34.91 34.72 34.51 34.30 31.04 30.14 27.28 26.23 25.74 25.51 18.77 14.52 14.51 13.45 13.34
ppm

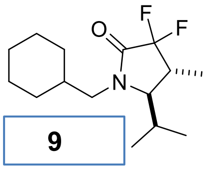

9

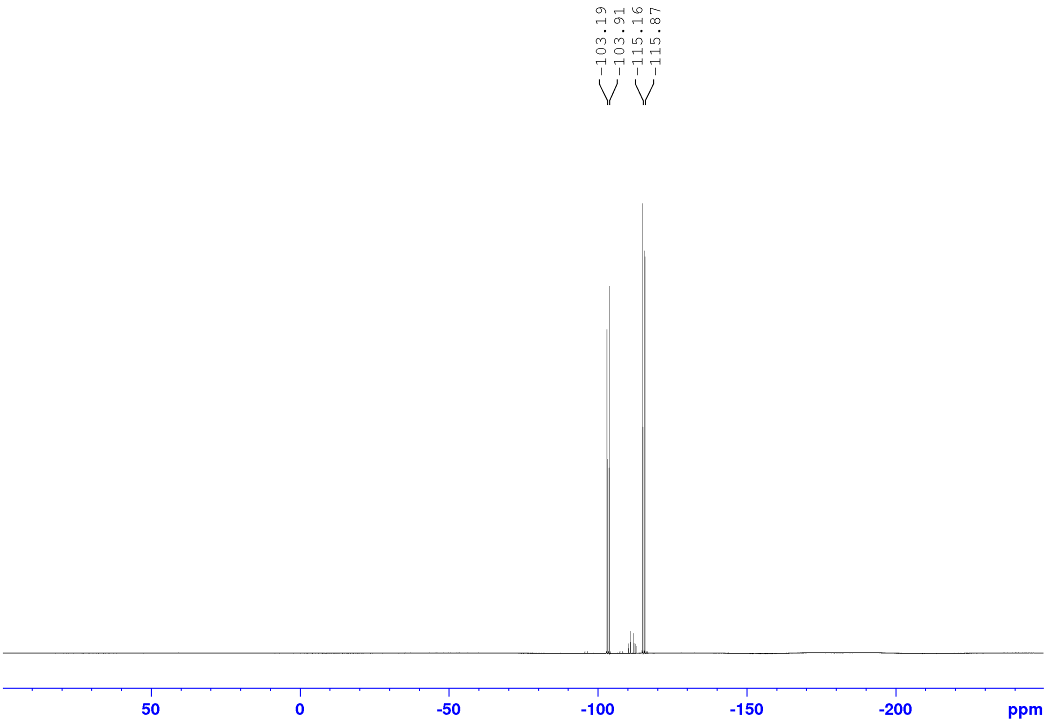

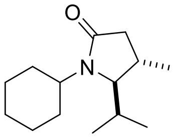
O
N

4b

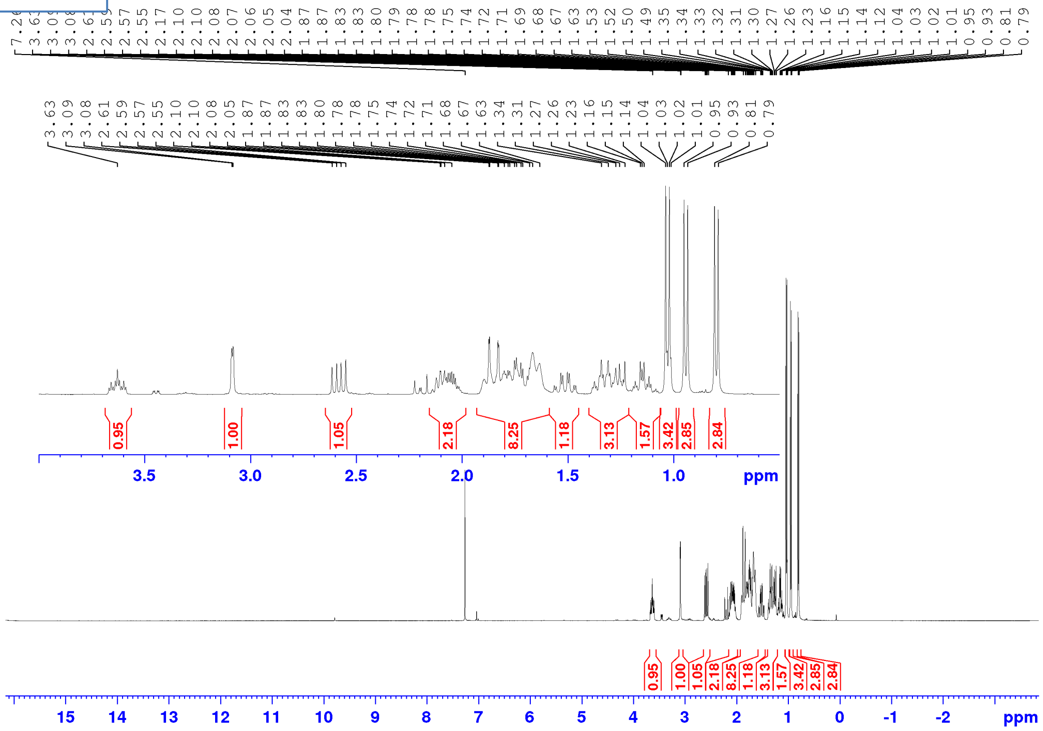
7.26 3.63 3.09 3.08 2.61 2.59 2.57 2.55 2.17 2.10 2.10 2.08 2.07 2.06 2.05 2.04 1.87 1.87 1.83 1.80 1.79 1.78 1.78 1.75 1.74 1.72 1.71 1.69 1.68 1.67 1.63 1.53 1.52 1.50 1.49 1.35 1.34 1.33 1.32 1.31 1.30 1.27 1.26 1.23 1.16 1.15 1.14 1.12 1.04 1.03 1.02 1.01 0.95 0.93 0.81 0.79
3.63 3.09 3.08 2.61 2.59 2.57 2.55 2.10 2.10 2.08 2.05 1.87 1.87 1.83 1.83 1.80 1.78 1.78 1.75 1.74 1.72 1.71 1.68 1.67 1.63 1.34 1.31 1.27 1.26 1.23 1.16 1.15 1.15 1.14 1.04 1.03 1.02 1.01 0.95 0.93 0.81 0.79
0.95 1.00 1.05 2.18 8.25 1.18 3.13 1.57 3.42 2.85 2.84
3.5 3.0 2.5 2.0 1.5 1.0 ppm
0.95 1.00 1.05 2.18 8.25 1.18 3.13 1.57 3.42 2.85 2.84
15 14 13 12 11 10 9 8 7 6 5 4 3 2 1 0 -1 -2 ppm

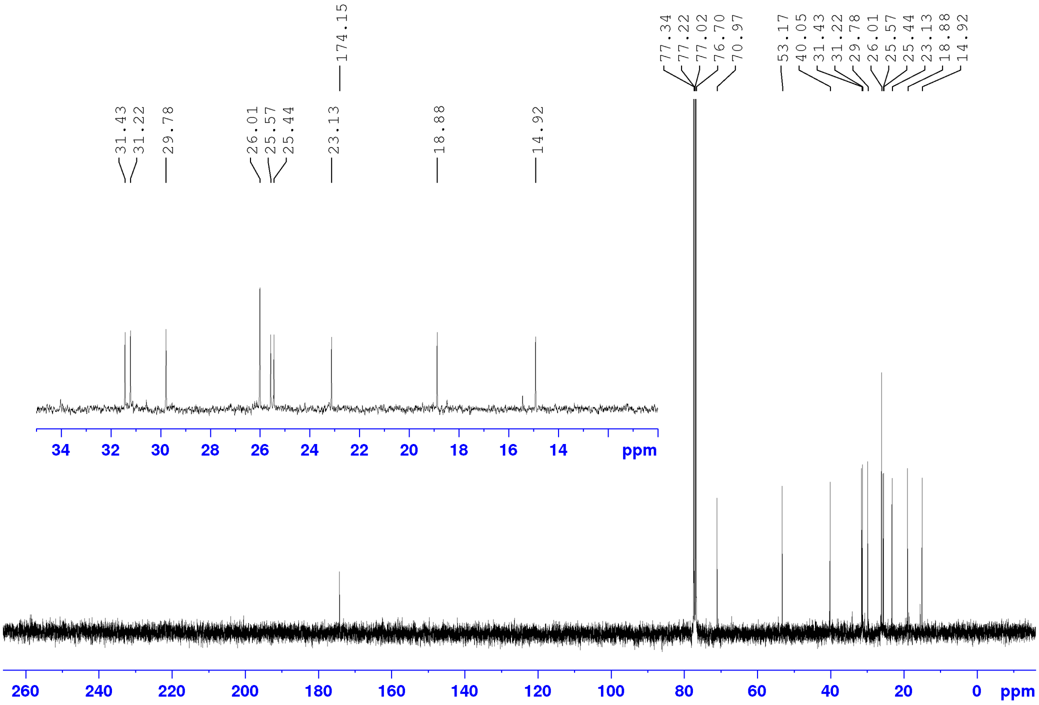
174.15
77.34 77.22 77.02 76.70 70.97
53.17 40.05 31.43 31.22 29.78 26.01 25.57 25.44 23.13 18.88 14.92
31.43 31.22 29.78 26.01 25.57 25.44 23.13 18.88 14.92
34 32 30 28 26 24 22 20 18 16 14 ppm
260 240 220 200 180 160 140 120 100 80 60 40 20 0 ppm

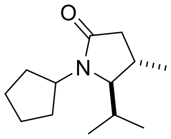

4c

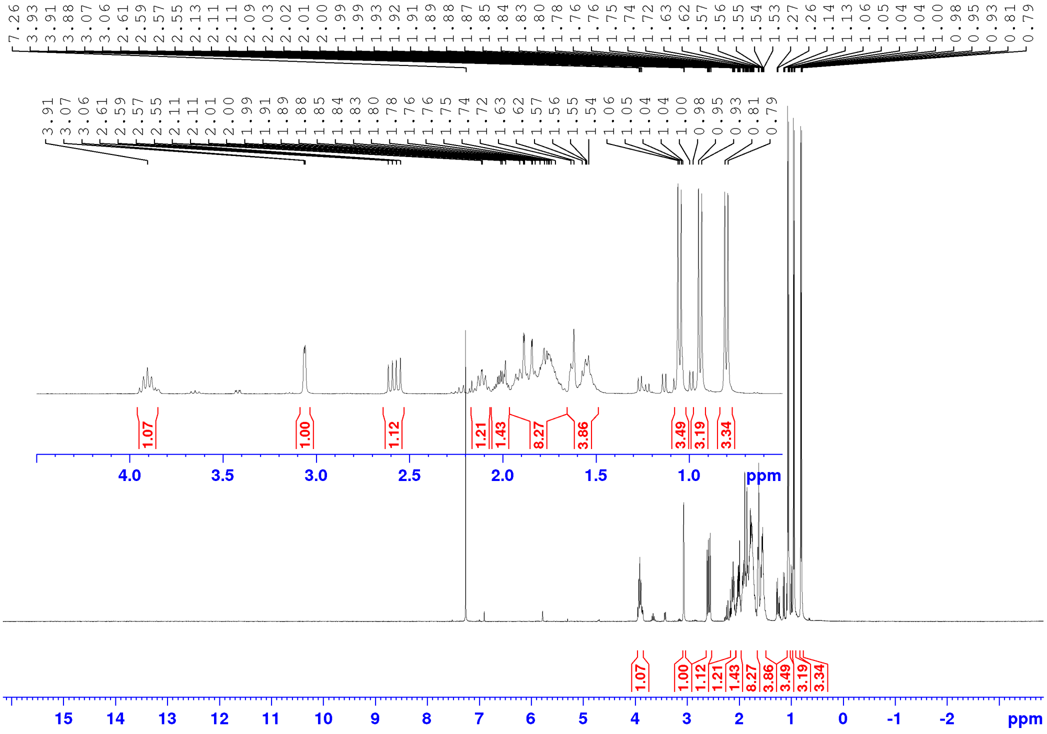
7.26 3.93 3.91 3.88 3.07 3.06 2.61 2.59 2.57 2.55 2.13 2.11 2.11 2.09 2.03 2.02 2.01 2.00 1.99 1.99 1.93 1.92 1.91 1.89 1.88 1.87 1.85 1.84 1.83 1.80 1.78 1.76 1.76 1.75 1.74 1.72 1.63 1.62 1.57 1.56 1.55 1.54 1.53 1.27 1.26 1.14 1.13 1.06 1.05 1.04 1.04 1.00 0.98 0.95 0.93 0.81 0.79
3.91 3.07 3.06 2.61 2.59 2.57 2.55 2.11 2.11 2.01 2.00 1.99 1.91 1.89 1.88 1.85 1.84 1.83 1.80 1.78 1.76 1.76 1.75 1.74 1.72 1.63 1.62 1.57 1.56 1.55 1.54 1.06 1.05 1.04 1.04 1.00 0.98 0.95 0.93 0.81 0.79
1.07 1.00 1.12 1.21 1.43 8.27 3.86 3.49 3.19 3.34
ppm

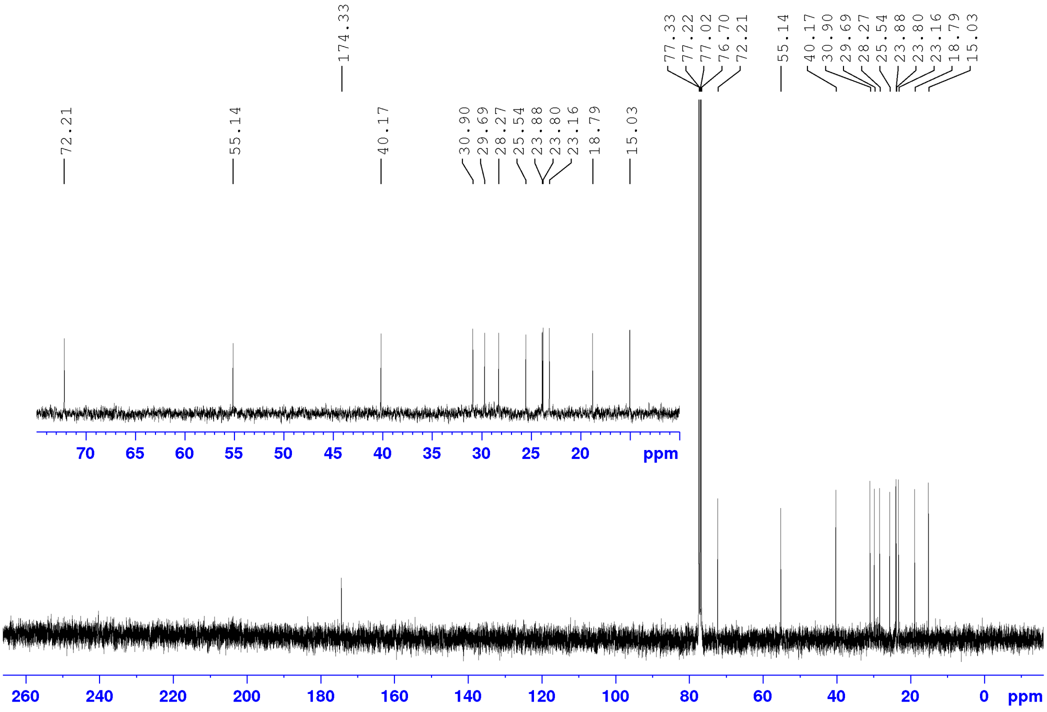
174.33
77.33 77.22 77.02 76.70 72.21 55.14 40.17 30.90 29.69 28.27 25.54 23.88 23.80 23.16 18.79 15.03
72.21 55.14 40.17 30.90 29.69 28.27 25.54 23.88 23.80 23.16 18.79 15.03
ppm

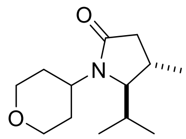

4d

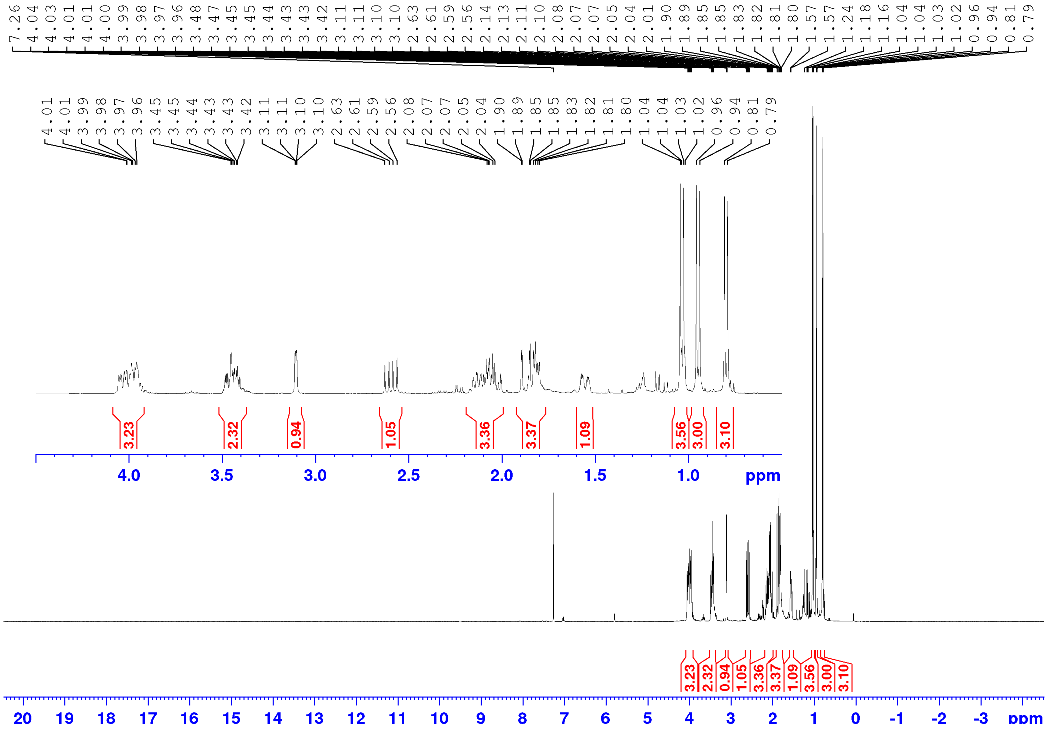
7.26 4.04 4.03 4.01 4.01 4.00 3.99 3.98 3.97 3.96 3.48 3.47 3.45 3.45 3.44 3.43 3.43 3.42 3.11 3.11 3.10 3.10 2.63 2.61 2.59 2.56 2.14 2.13 2.11 2.10 2.08 2.07 2.07 2.05 2.04 2.01 1.90 1.89 1.85 1.85 1.83 1.82 1.81 1.80 1.57 1.57 1.24 1.18 1.16 1.04 1.04 1.03 1.02 0.96 0.94 0.81 0.79
4.01 4.01 3.99 3.98 3.97 3.96 3.45 3.45 3.44 3.43 3.43 3.42 3.11 3.11 3.10 3.10 2.63 2.61 2.59 2.56 2.08 2.07 2.05 2.04 1.90 1.89 1.85 1.83 1.82 1.81 1.80 1.04 1.04 1.03 1.02 0.96 0.94 0.81 0.79
3.23 2.32 0.94 1.05 3.36 3.37 1.09 3.56 3.00 3.10
4.0 3.5 3.0 2.5 2.0 1.5 1.0 ppm
20 19 18 17 16 15 14 13 12 11 10 9 8 7 6 5 4 3 2 1 0 -1 -2 -3 ppm

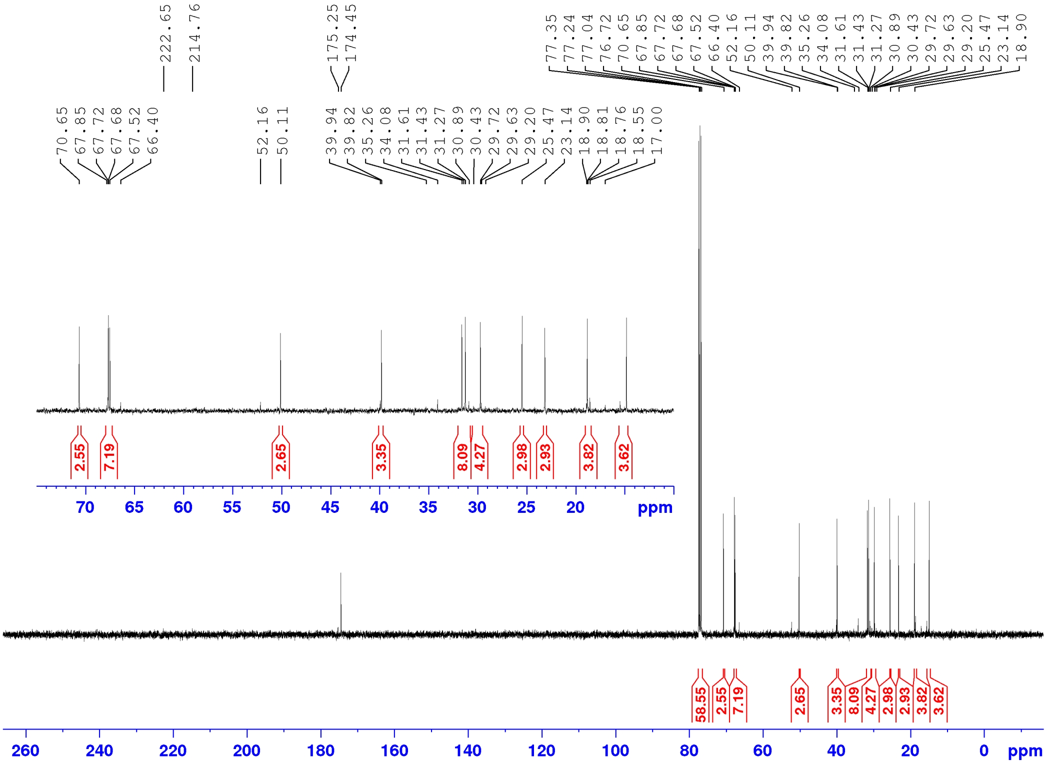
222.65 214.76 175.25 174.45 77.35 77.24 77.04 76.72 70.65 67.85 67.72 67.68 67.52 66.40 52.16 50.11 39.94 39.82 35.26 34.08 31.61 31.43 31.27 30.89 30.43 29.72 29.63 29.20 25.47 23.14 18.90
70.65 67.85 67.72 67.68 67.52 66.40 52.16 50.11 39.94 39.82 35.26 34.08 31.61 31.43 31.27 30.89 30.43 29.72 29.63 29.20 25.47 23.14 18.90 18.81 18.76 18.55 17.00
2.55 7.19 2.65 3.35 8.09 4.27 2.98 2.93 3.82 3.62
70 65 60 55 50 45 40 35 30 25 20 ppm
58.55 2.55 7.19 2.65 3.35 8.09 4.27 2.98 2.93 3.82 3.62
260 240 220 200 180 160 140 120 100 80 60 40 20 0 ppm

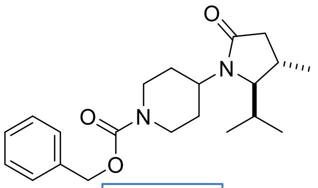

4e

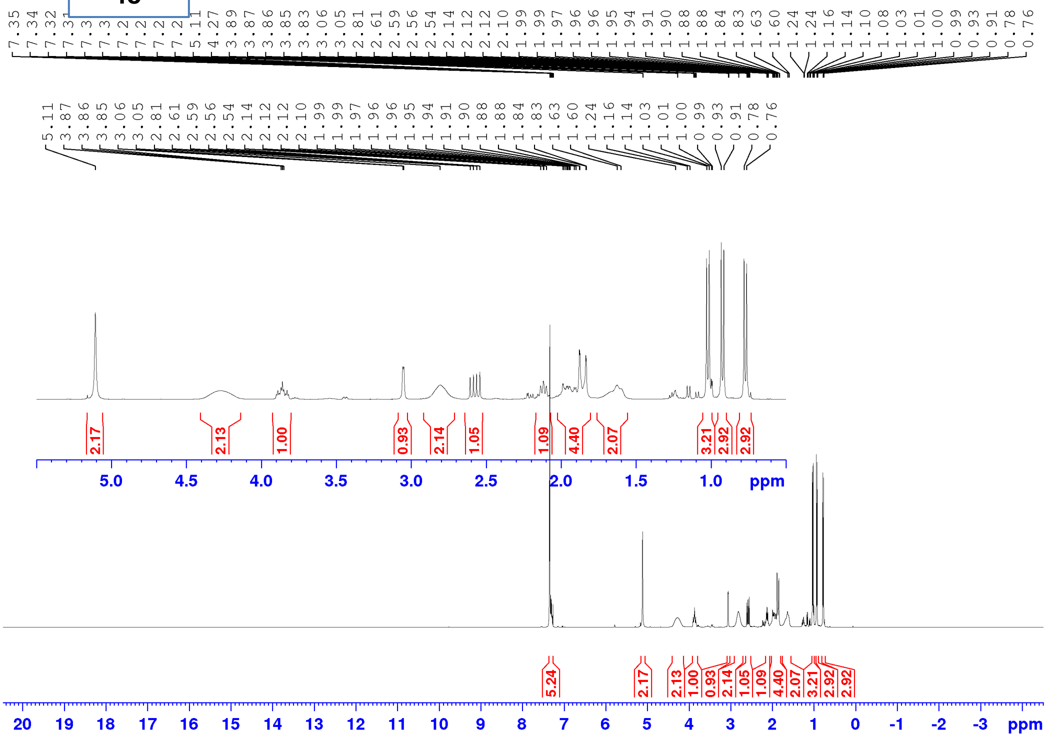
7.35 7.34 7.32 7.31 7.3 7.3 7.2 7.2 7.2 7.2 5.11 4.27 3.89 3.87 3.86 3.85 3.83 3.06 3.05 2.81 2.61 2.59 2.56 2.54 2.14 2.12 2.12 2.10 1.99 1.99 1.97 1.96 1.96 1.95 1.94 1.91 1.90 1.88 1.88 1.84 1.83 1.63 1.60 1.24 1.24 1.16 1.14 1.10 1.08 1.03 1.01 1.00 0.99 0.93 0.91 0.78 0.76
5.11 3.87 3.86 3.85 3.06 3.05 2.81 2.61 2.59 2.56 2.54 2.14 2.12 2.12 2.10 1.99 1.99 1.97 1.96 1.96 1.95 1.94 1.91 1.90 1.88 1.88 1.84 1.83 1.63 1.60 1.24 1.16 1.14 1.03 1.01 1.00 0.99 0.93 0.91 0.78 0.76
2.17 2.13 1.00 0.93 2.14 1.05 1.09 4.40 2.07 3.21 2.92 2.92
5.0 4.5 4.0 3.5 3.0 2.5 2.0 1.5 1.0 ppm
5.24 2.17 2.13 1.00 0.93 2.14 1.05 1.09 4.40 2.07 3.21 2.92 2.92
20 19 18 17 16 15 14 13 12 11 10 9 8 7 6 5 4 3 2 1 0 -1 -2 -3 ppm

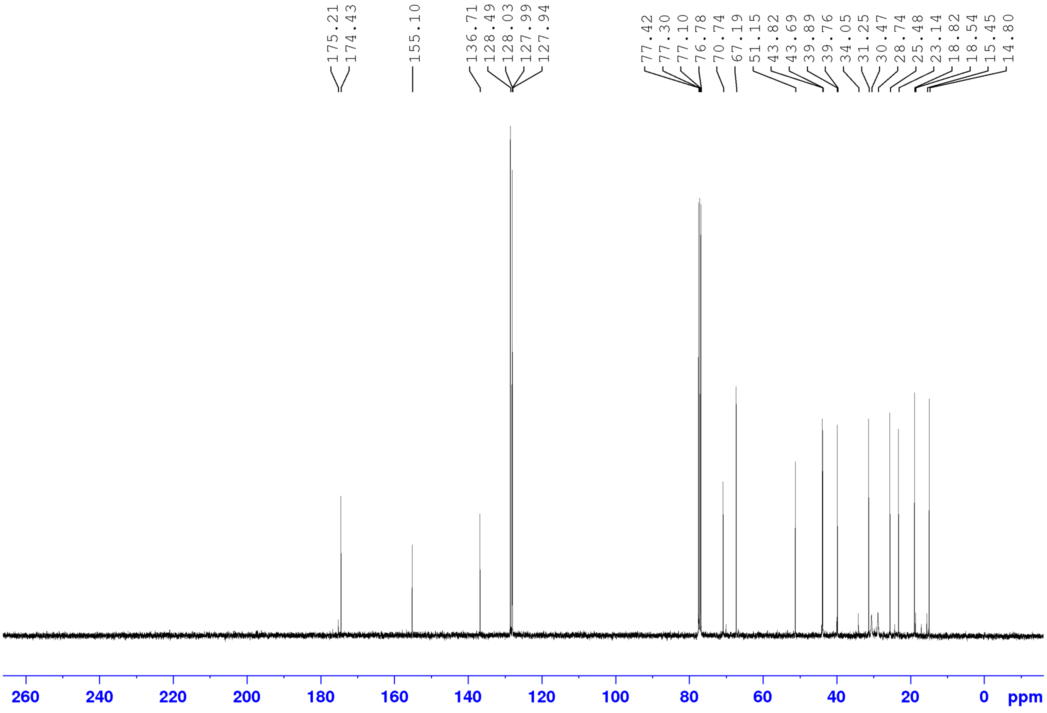
175.21 174.43 155.10 136.71 128.49 128.03 127.99 127.94 77.42 77.30 77.10 76.78 70.74 67.19 51.15 43.82 43.69 39.89 39.76 34.05 31.25 30.47 28.74 25.48 23.14 18.82 18.54 15.45 14.80
260 240 220 200 180 160 140 120 100 80 60 40 20 0 ppm

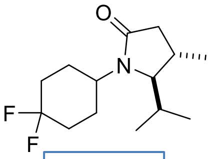
O
N
F
F

4f

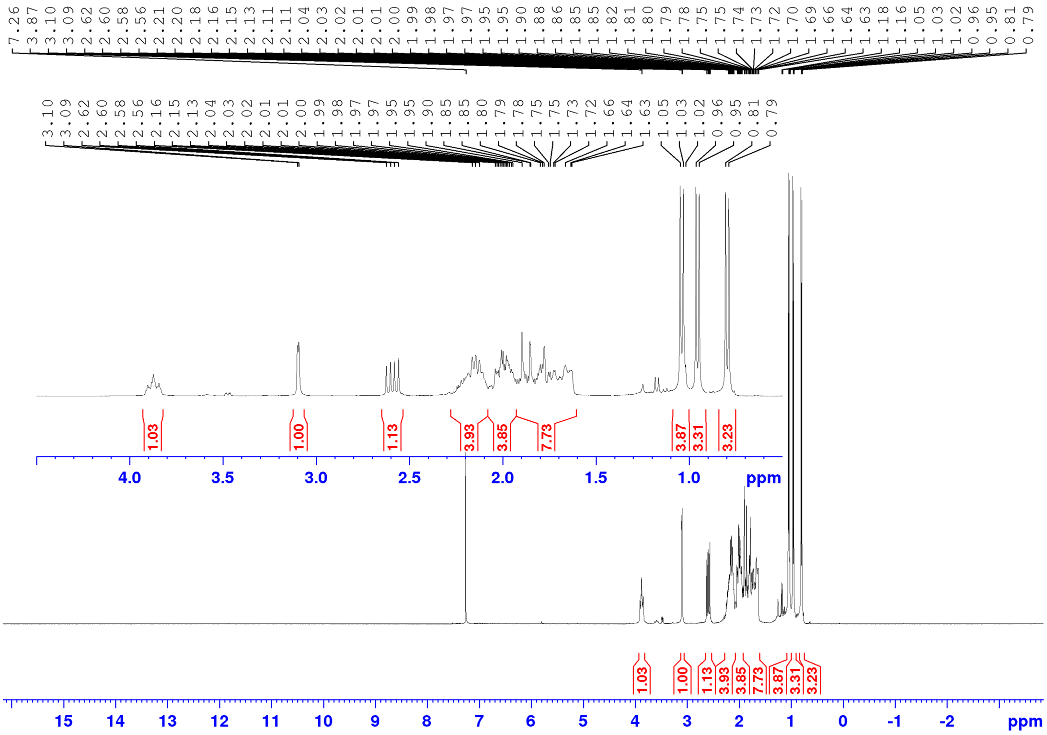
7.26 3.87 3.10 3.09 2.62 2.60 2.58 2.56 2.21 2.20 2.18 2.16 2.15 2.13 2.11 2.11 2.04 2.03 2.02 2.01 2.01 2.00 1.99 1.98 1.97 1.97 1.95 1.95 1.90 1.88 1.86 1.85 1.85 1.82 1.81 1.80 1.79 1.78 1.75 1.75 1.74 1.73 1.73 1.72 1.70 1.69 1.66 1.64 1.63 1.18 1.16 1.05 1.03 1.02 0.96 0.95 0.81 0.79
3.10 3.09 2.62 2.60 2.58 2.56 2.16 2.15 2.13 2.04 2.03 2.02 2.01 2.01 2.00 1.99 1.98 1.97 1.97 1.95 1.95 1.90 1.85 1.85 1.80 1.79 1.78 1.75 1.75 1.73 1.72 1.66 1.64 1.63 1.05 1.03 1.02 0.96 0.95 0.81 0.79
1.03 1.00 1.13 3.93 3.85 7.73 3.87 3.31 3.23
4.0 3.5 3.0 2.5 2.0 1.5 1.0 ppm
1.03 1.00 1.13 3.93 3.85 7.73 3.87 3.31 3.23
15 14 13 12 11 10 9 8 7 6 5 4 3 2 1 0 -1 -2 ppm

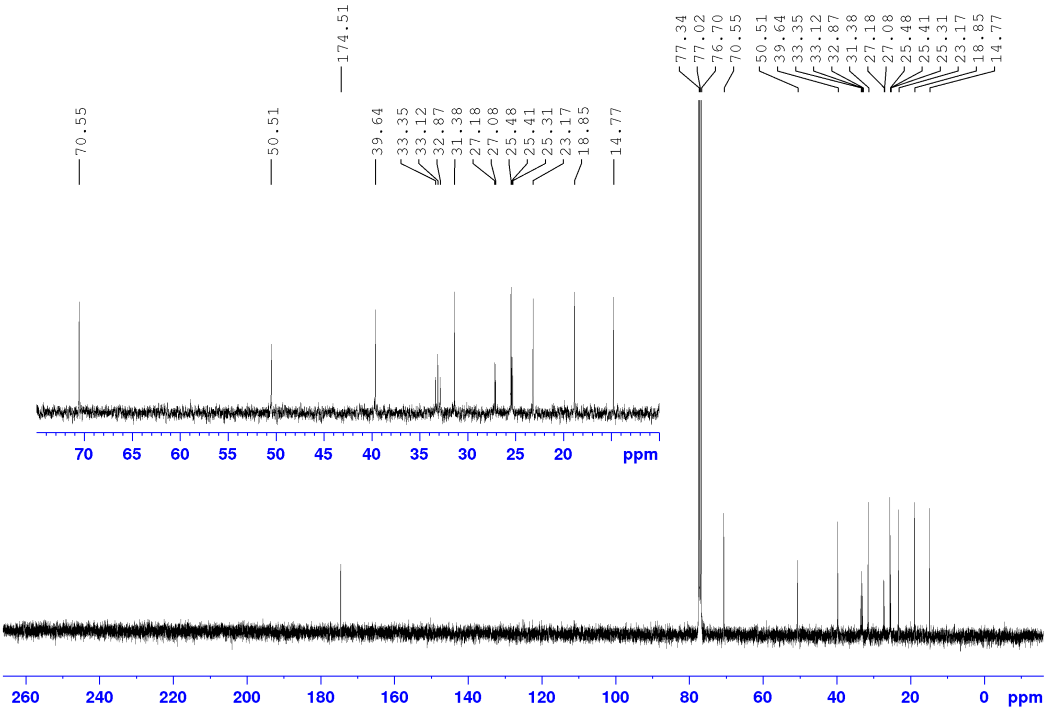
174.51
77.34 77.02 76.70 70.55 50.51 39.64 33.35 33.12 32.87 31.38 27.18 27.08 25.48 25.41 25.31 23.17 18.85 14.77
70.55 50.51 39.64 33.35 33.12 32.87 31.38 27.18 27.08 25.48 25.41 25.31 23.17 18.85 14.77
70 65 60 55 50 45 40 35 30 25 20 ppm
260 240 220 200 180 160 140 120 100 80 60 40 20 0 ppm

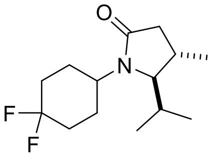

4f

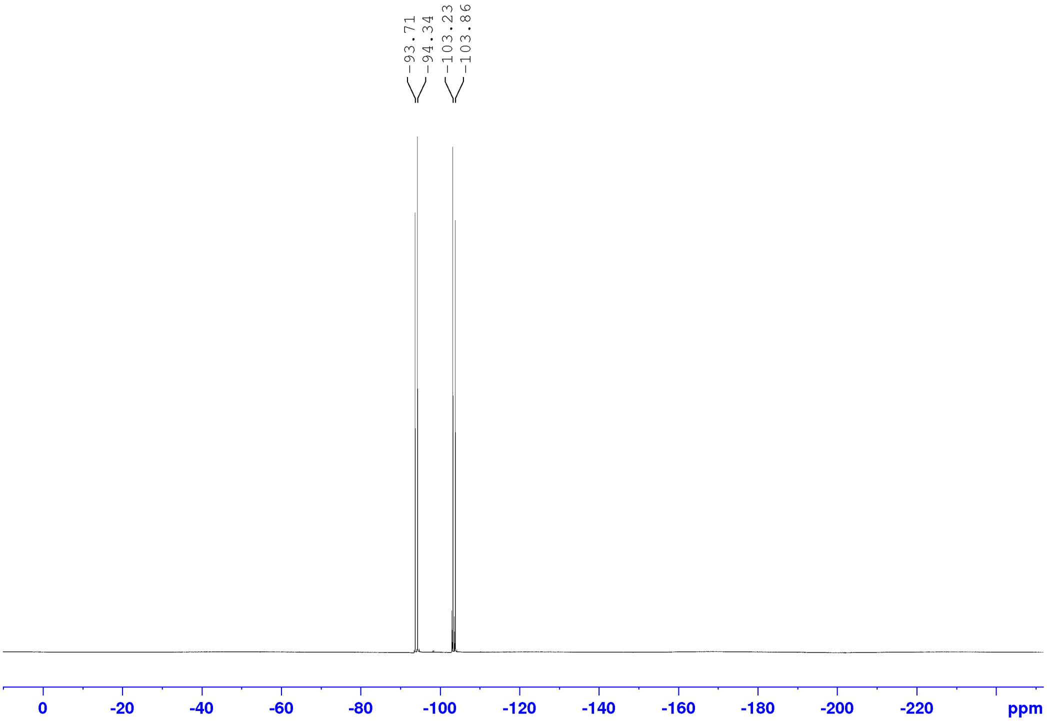
-93.71
-94.34
-103.23
-103.86
0
-20
-40
-60
-80
-100
-120
-140
-160
-180
-200
-220
ppm

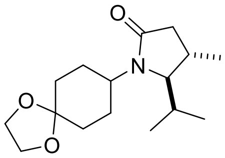

4g

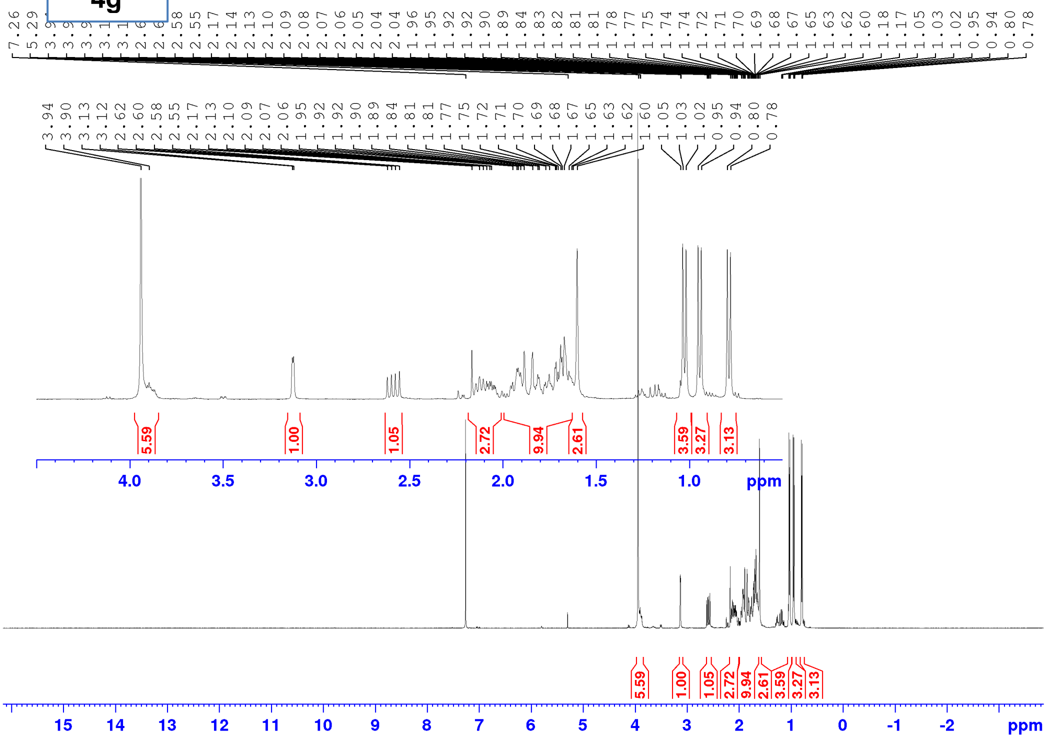
3.94 3.90 3.13 3.12 2.62 2.60 2.58 2.55 2.17 2.13 2.10 2.09 2.07 2.06 1.95 1.92 1.90 1.89 1.84 1.81 1.77 1.75 1.72 1.71 1.70 1.69 1.68 1.67 1.65 1.63 1.62 1.60 1.05 1.03 1.02 0.95 0.94 0.80 0.78
5.59 1.00 1.05 2.72 9.94 2.61 3.59 3.27 3.13
ppm

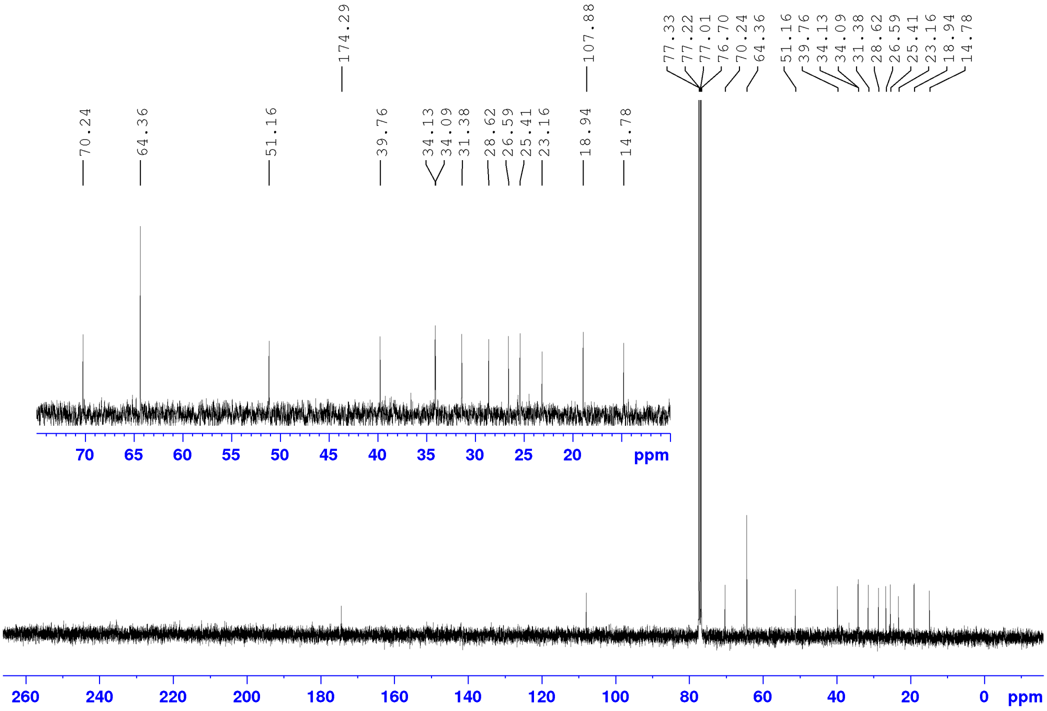
174.29
107.88
77.33 77.22 77.01 76.70 70.24 64.36 51.16 39.76 34.13 34.09 31.38 28.62 26.59 25.41 23.16 18.94 14.78
ppm

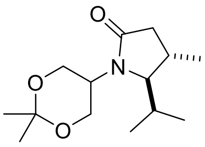
O
N
O
O

4h

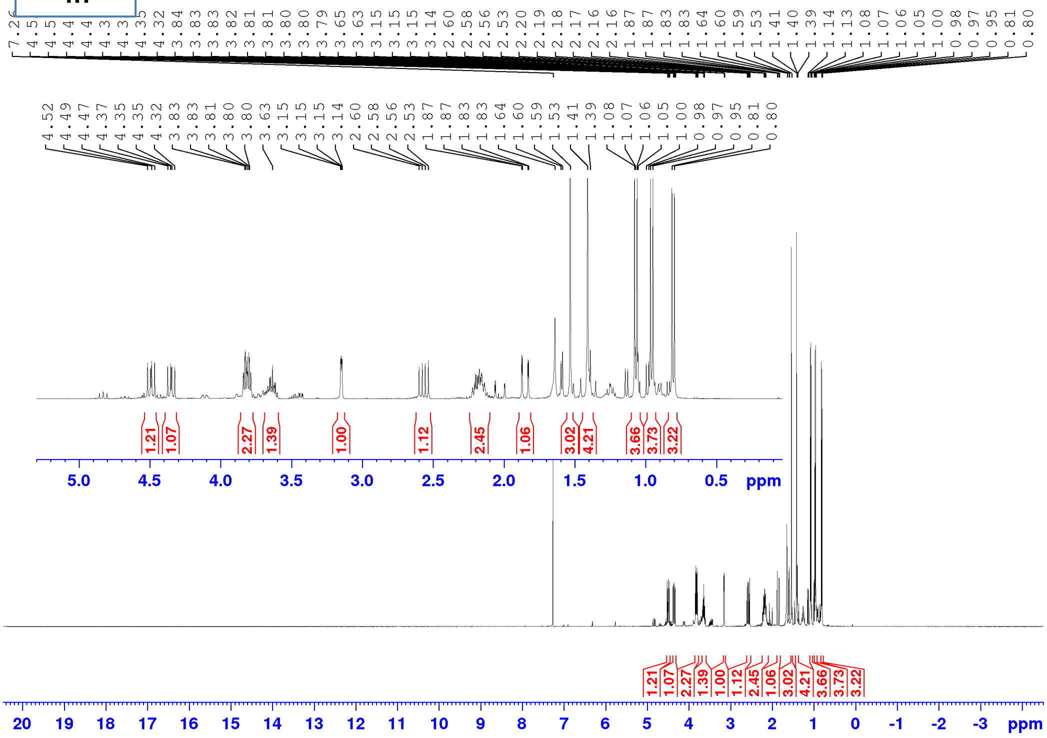
4.52 4.49 4.47 4.37 4.35 4.35 4.32 3.83 3.83 3.81 3.80 3.80 3.63 3.15 3.15 3.15 3.14 2.60 2.58 2.56 2.53 1.87 1.87 1.83 1.83 1.64 1.60 1.59 1.53 1.41 1.39 1.08 1.07 1.06 1.05 1.00 0.98 0.97 0.95 0.81 0.80
1.21 1.07 2.27 1.39 1.00 1.12 2.45 1.06 3.02 4.21 3.66 3.73 3.22
ppm

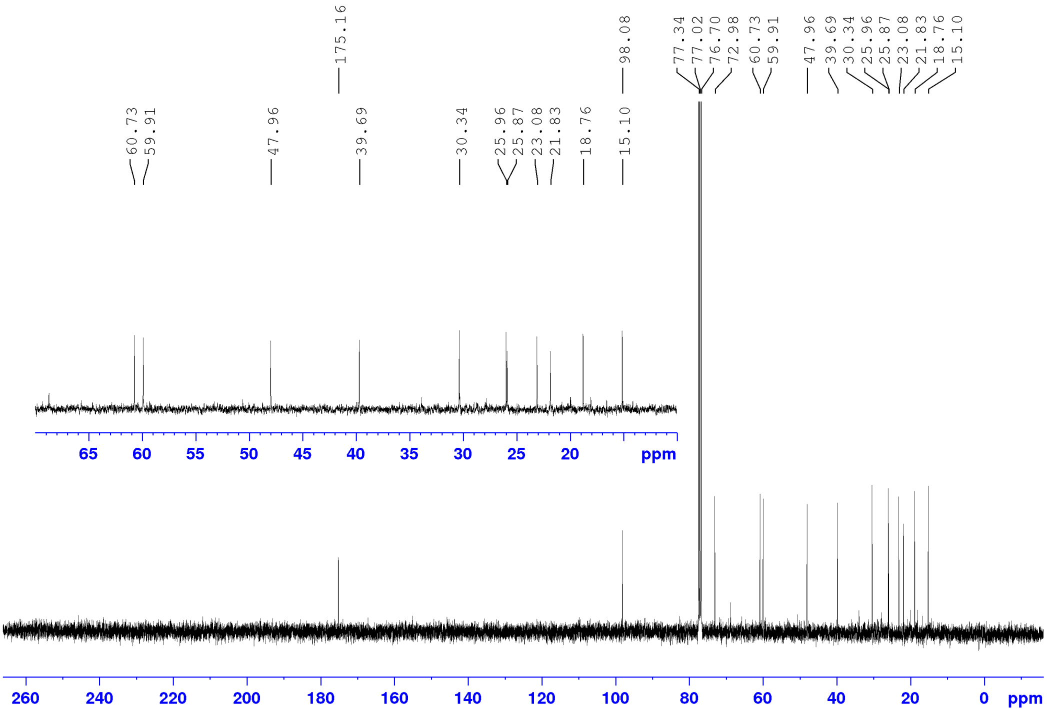
175.16 98.08 77.34 77.02 76.70 72.98 60.73 59.91 47.96 39.69 30.34 25.96 25.87 23.08 21.83 18.76 15.10
60.73 59.91 47.96 39.69 30.34 25.96 25.87 23.08 21.83 18.76 15.10
ppm

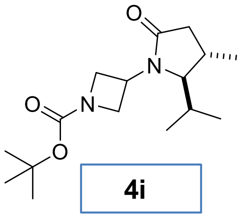

4i

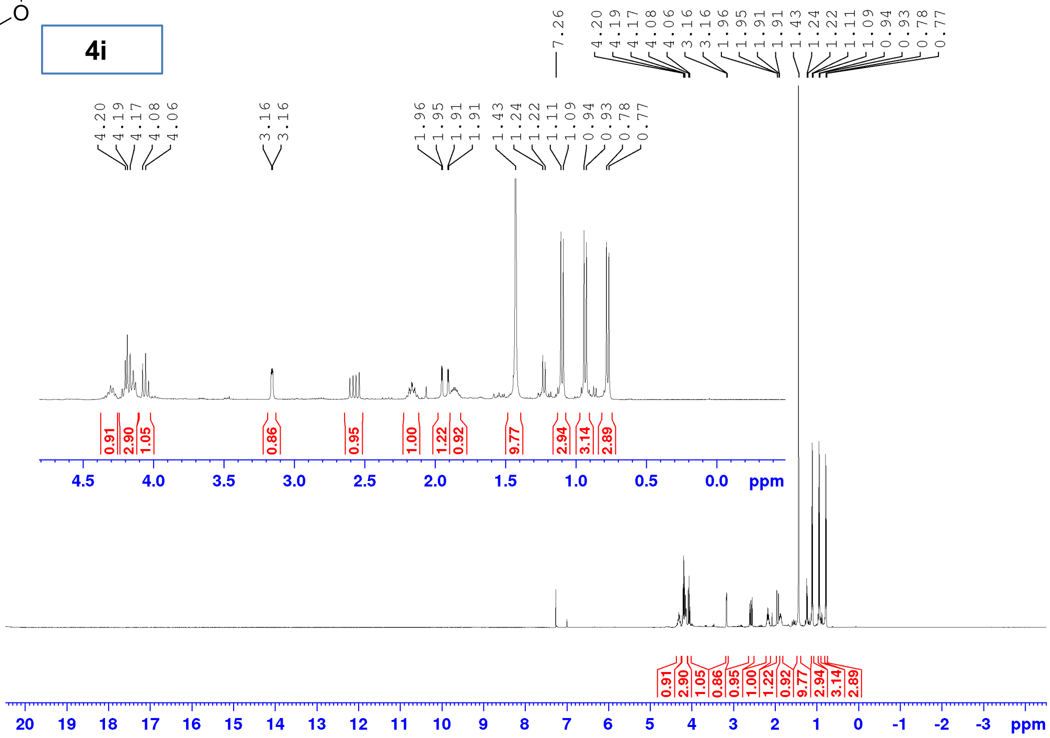

7.26
4.20
4.19
4.17
4.08
4.06
3.16
3.16
1.96
1.95
1.91
1.91
1.43
1.24
1.22
1.11
1.09
0.94
0.93
0.78
0.77
0.91
2.90
1.05
0.86
0.95
1.00
1.22
0.92
9.77
2.94
3.14
2.89
4.5 4.0 3.5 3.0 2.5 2.0 1.5 1.0 0.5 0.0 ppm
20 19 18 17 16 15 14 13 12 11 10 9 8 7 6 5 4 3 2 1 0 -1 -2 -3 ppm

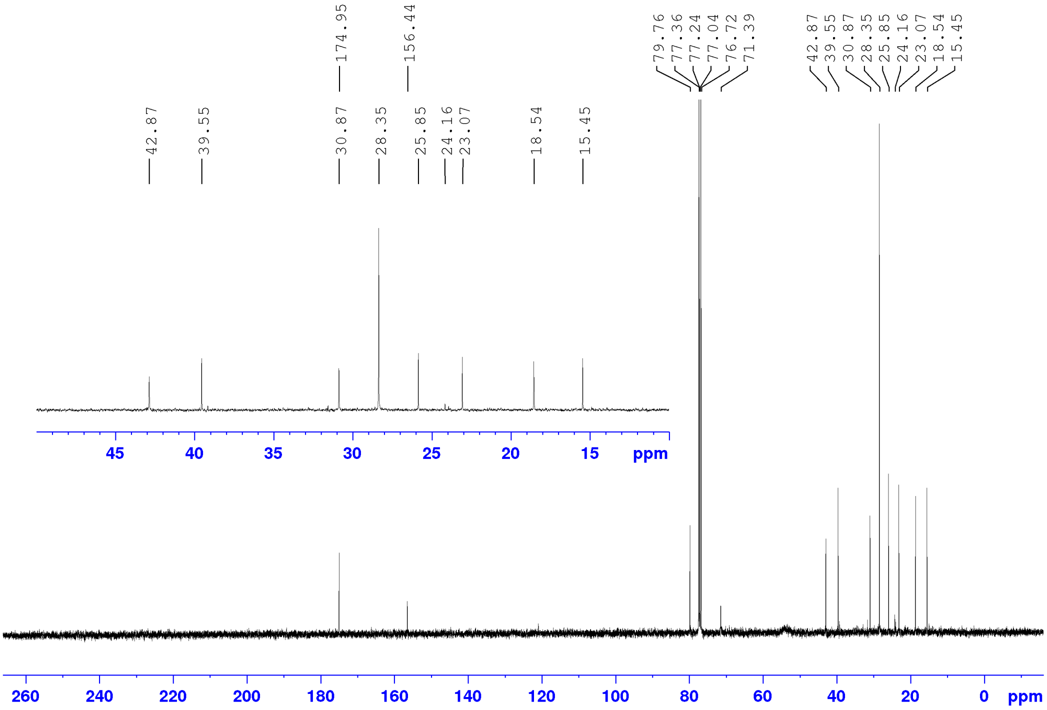

174.95
156.44
79.76
77.36
77.24
77.04
76.72
71.39
42.87
39.55
30.87
28.35
25.85
24.16
23.07
18.54
15.45
45 40 35 30 25 20 15 ppm
260 240 220 200 180 160 140 120 100 80 60 40 20 0 ppm

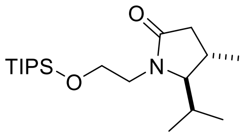

4j

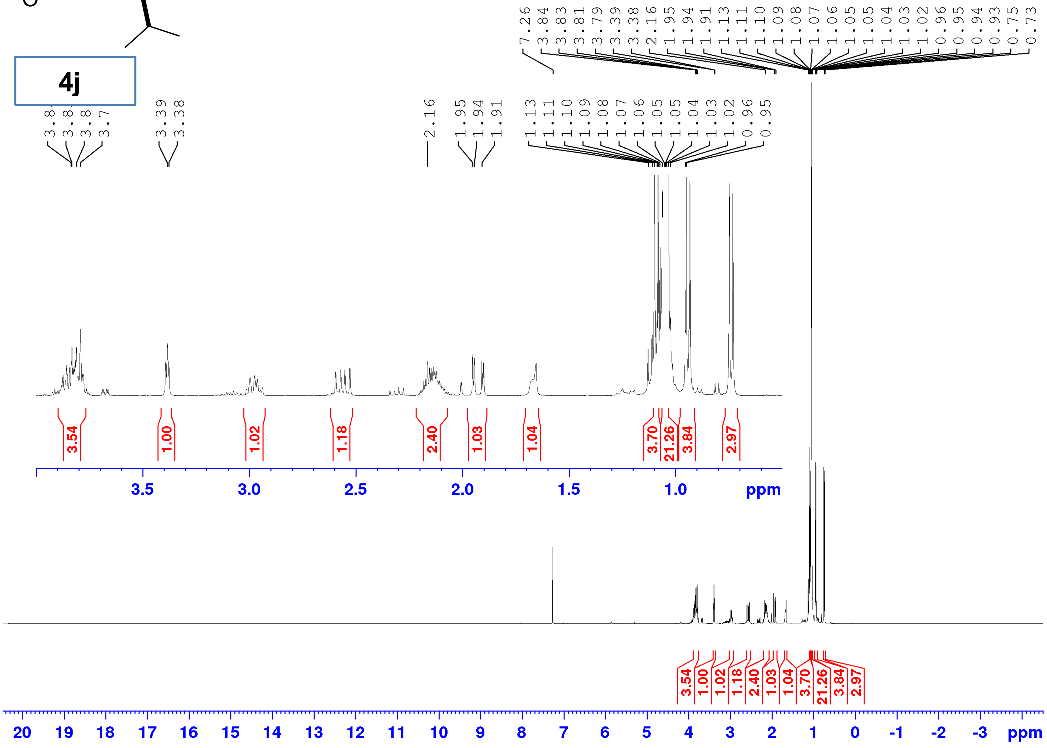

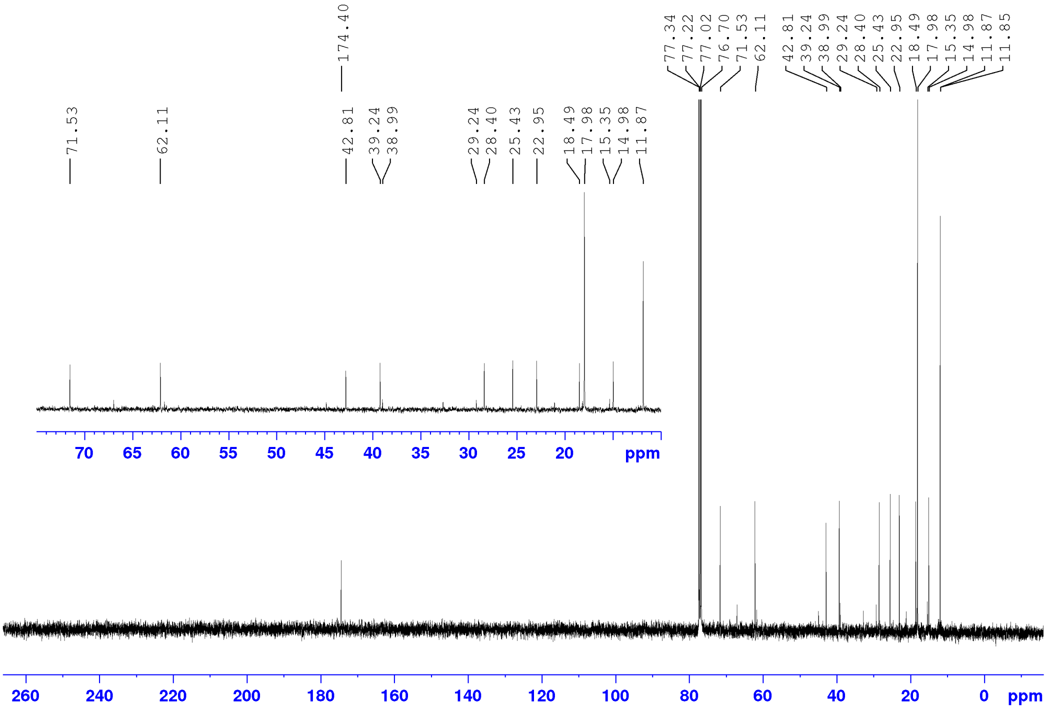

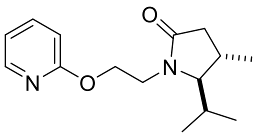
O
N
O
N

4k

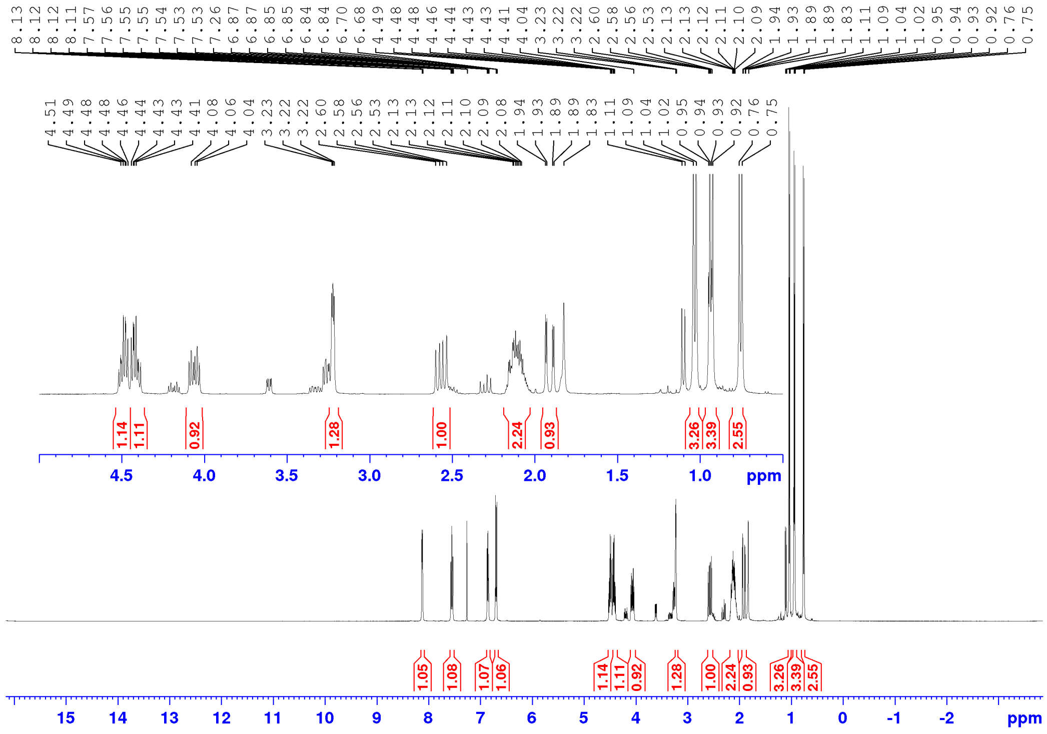
8.13 8.12 8.12 8.11 7.57 7.56 7.55 7.55 7.54 7.53 7.53 7.26 6.87 6.87 6.85 6.85 6.84 6.84 6.70 6.68 4.49 4.48 4.48 4.46 4.44 4.43 4.43 4.41 4.04 3.23 3.22 3.22 2.60 2.58 2.56 2.53 2.13 2.13 2.12 2.12 2.11 2.10 2.09 1.94 1.93 1.89 1.89 1.83 1.11 1.09 1.04 1.02 0.95 0.94 0.93 0.93 0.92 0.76 0.75
4.51 4.49 4.48 4.48 4.46 4.44 4.43 4.43 4.41 4.08 4.06 4.04 3.23 3.22 3.22 2.60 2.58 2.56 2.53 2.13 2.13 2.12 2.11 2.10 2.09 2.08 1.94 1.93 1.89 1.89 1.83 1.11 1.09 1.04 1.02 0.95 0.94 0.93 0.92 0.76 0.75
1.14 1.11 0.92 1.28 1.00 2.24 0.93 3.26 3.39 2.55
4.5 4.0 3.5 3.0 2.5 2.0 1.5 1.0 ppm
1.05 1.08 1.07 1.06 1.14 1.11 0.92 1.28 1.00 2.24 0.93 3.26 3.39 2.55
15 14 13 12 11 10 9 8 7 6 5 4 3 2 1 0 -1 -2 ppm

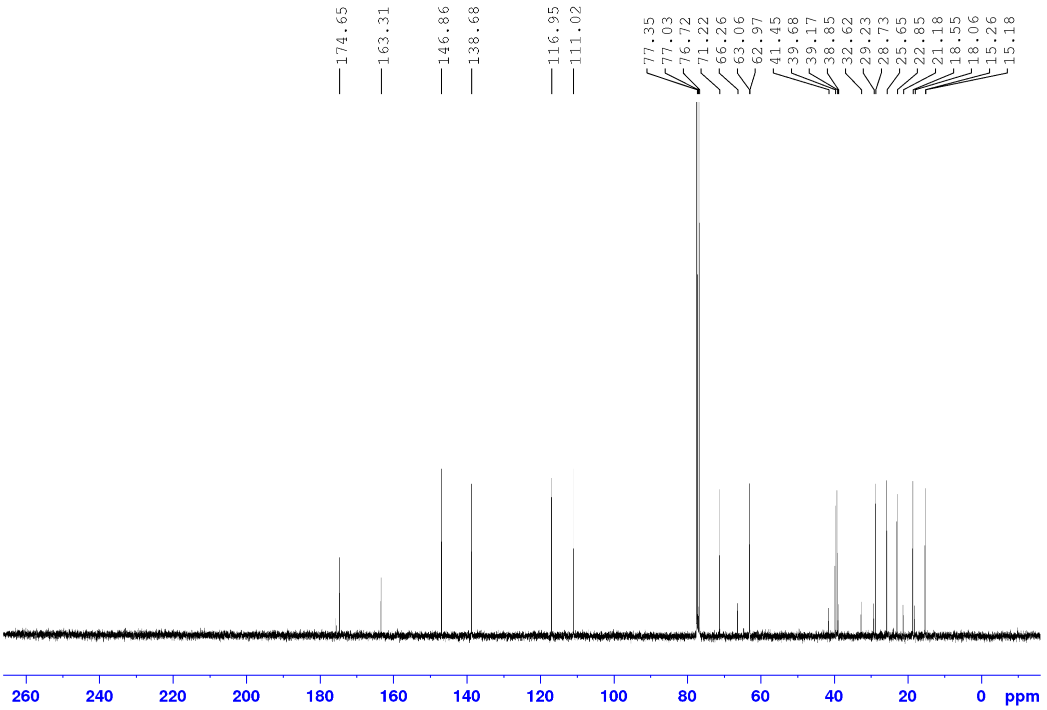
174.65 163.31 146.86 138.68 116.95 111.02
77.35 77.03 76.72 71.22 66.26 63.06 62.97 41.45 39.68 39.17 38.85 32.62 29.23 28.73 25.65 22.85 21.18 18.55 18.06 15.26 15.18
260 240 220 200 180 160 140 120 100 80 60 40 20 0 ppm

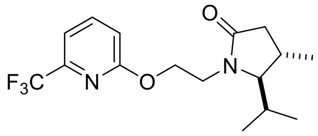

4l

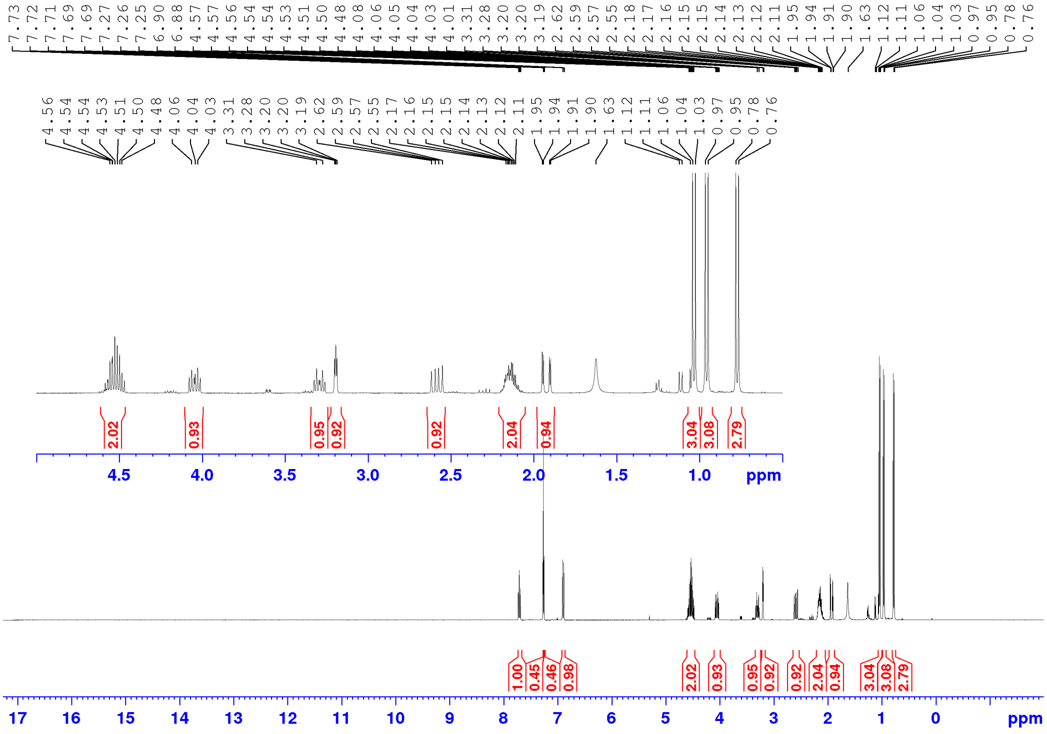

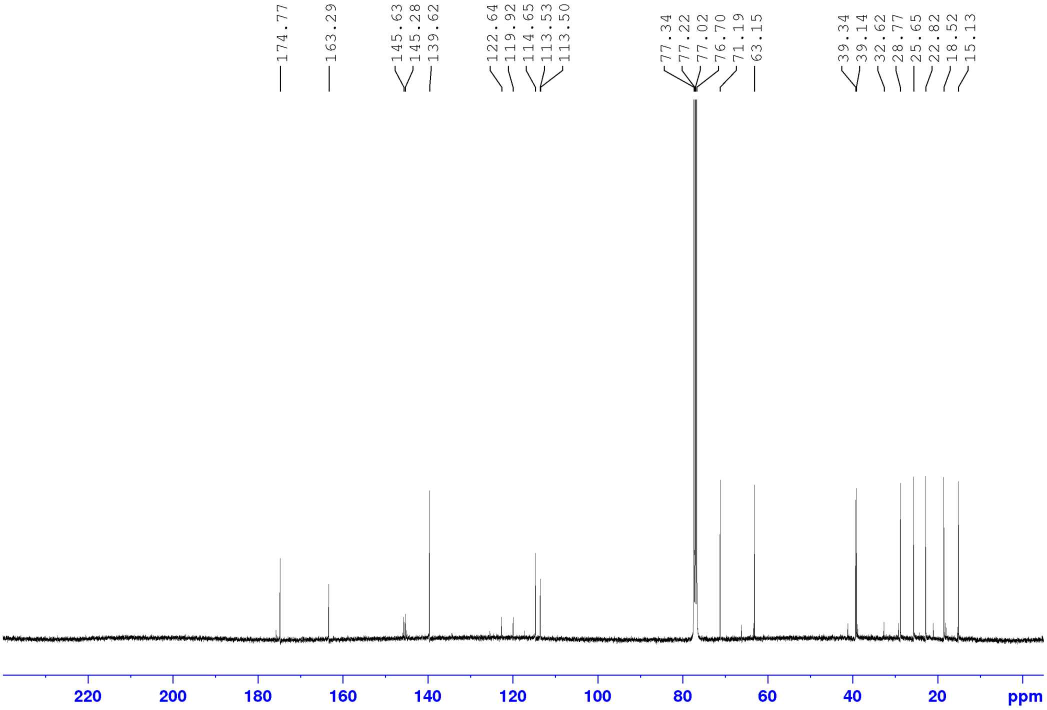

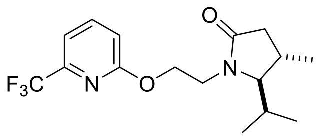

4l

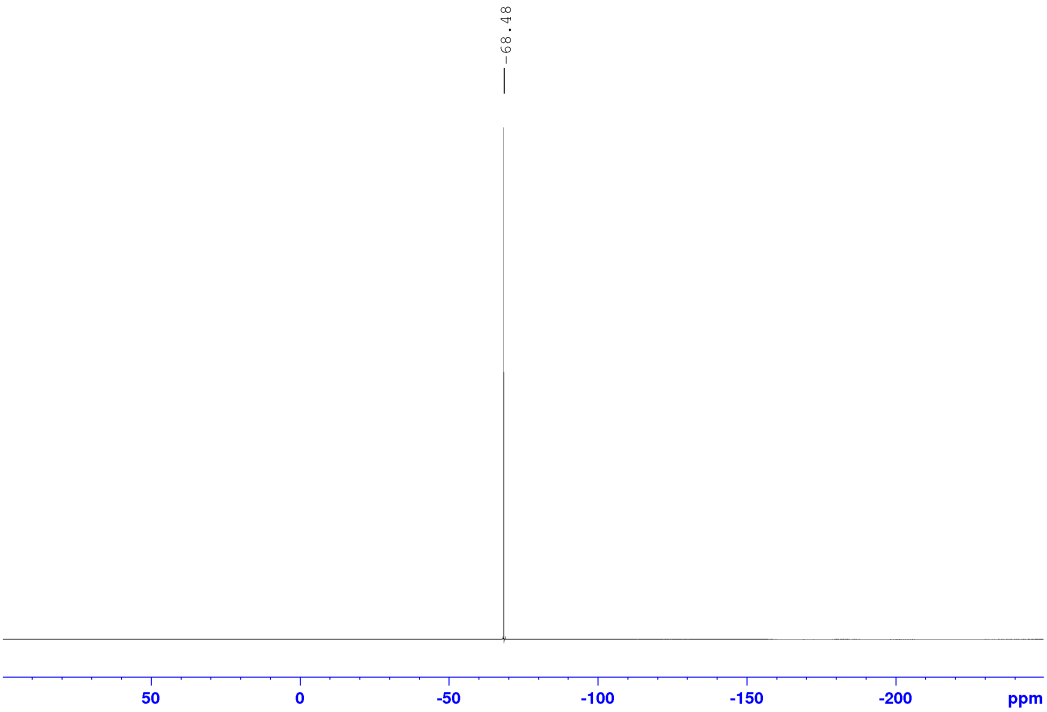

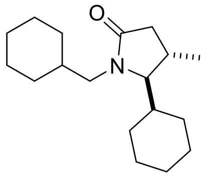

4m

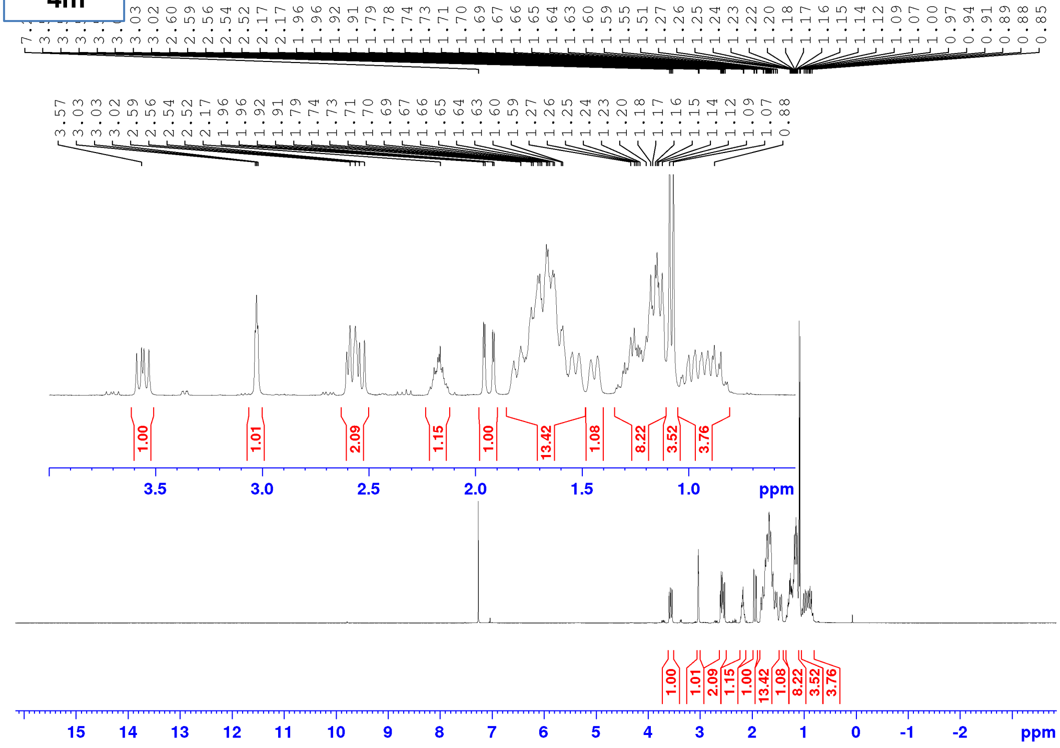
3.03 3.02 2.60 2.59 2.56 2.54 2.52 2.17 2.17 1.96 1.96 1.92 1.91 1.79 1.78 1.74 1.73 1.71 1.70 1.69 1.67 1.66 1.65 1.64 1.63 1.60 1.59 1.55 1.51 1.27 1.26 1.25 1.24 1.23 1.22 1.20 1.18 1.17 1.16 1.15 1.14 1.12 1.09 1.07 1.00 0.97 0.94 0.91 0.89 0.88 0.85
3.57 3.03 3.03 3.02 2.59 2.56 2.54 2.52 2.17 1.96 1.96 1.92 1.91 1.79 1.74 1.73 1.71 1.70 1.69 1.67 1.66 1.65 1.64 1.63 1.60 1.59 1.27 1.26 1.25 1.24 1.23 1.20 1.18 1.17 1.16 1.15 1.14 1.12 1.09 1.07 0.88
1.00 1.01 2.09 1.15 1.00 13.42 1.08 8.22 3.52 3.76
3.5 3.0 2.5 2.0 1.5 1.0 ppm
15 14 13 12 11 10 9 8 7 6 5 4 3 2 1 0 -1 -2 ppm

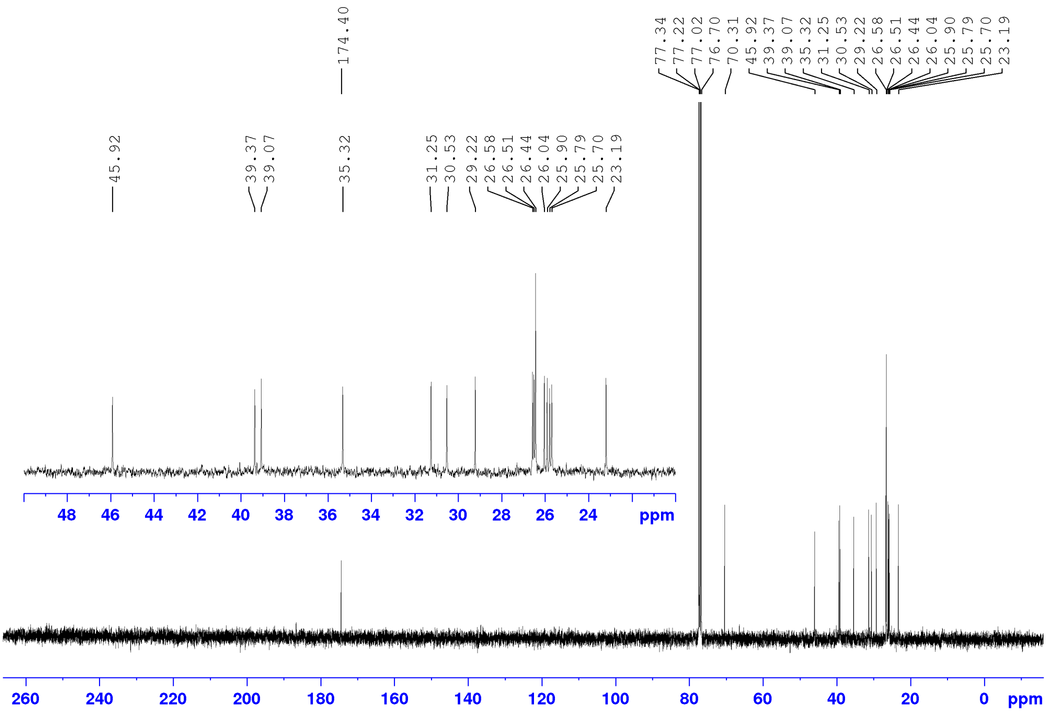
174.40
77.34 77.22 77.02 76.70 70.31 45.92 39.37 39.07 35.32 31.25 30.53 29.22 26.58 26.51 26.44 26.04 25.90 25.79 25.70 23.19
45.92 39.37 39.07 35.32 31.25 30.53 29.22 26.58 26.51 26.44 26.04 25.90 25.79 25.70 23.19
48 46 44 42 40 38 36 34 32 30 28 26 24 ppm
260 240 220 200 180 160 140 120 100 80 60 40 20 0 ppm

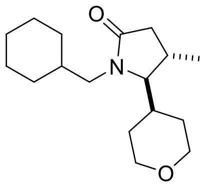

4n

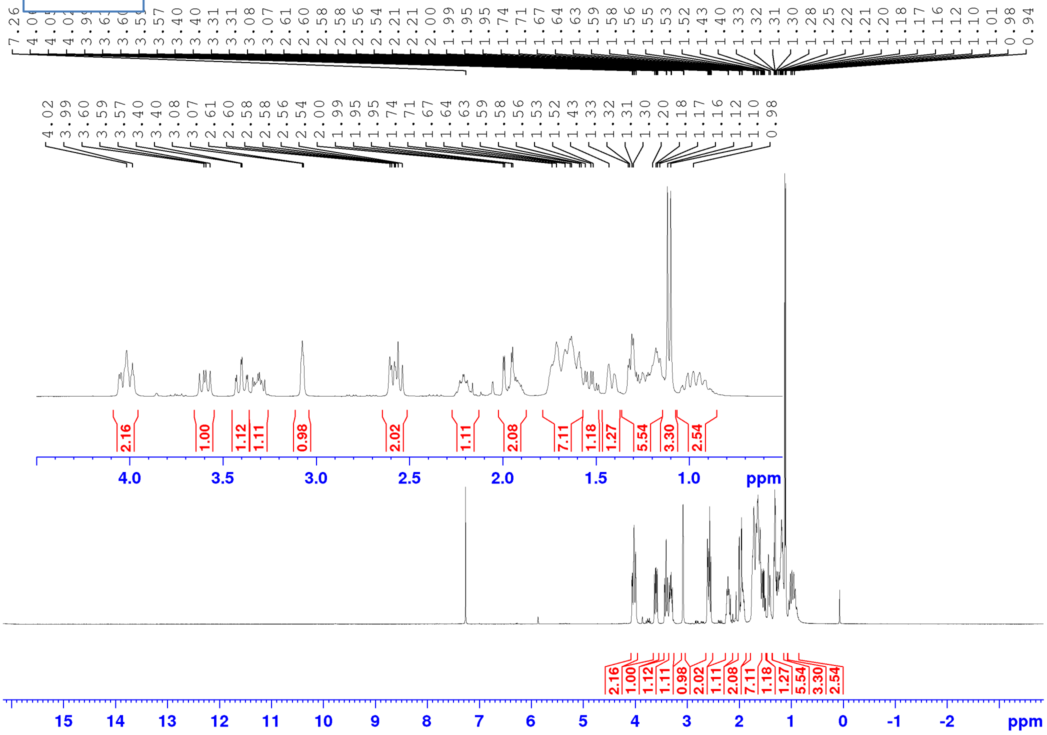
4.02 3.99 3.60 3.59 3.57 3.40 3.40 3.08 3.07 2.61 2.60 2.58 2.56 2.54 2.00 1.99 1.95 1.95 1.74 1.71 1.67 1.64 1.63 1.59 1.58 1.56 1.53 1.52 1.43 1.33 1.32 1.31 1.30 1.20 1.18 1.17 1.16 1.12 1.10 0.98
2.16 1.00 1.12 1.11 0.98 2.02 1.11 2.08 7.11 1.18 1.27 5.54 3.30 2.54
ppm

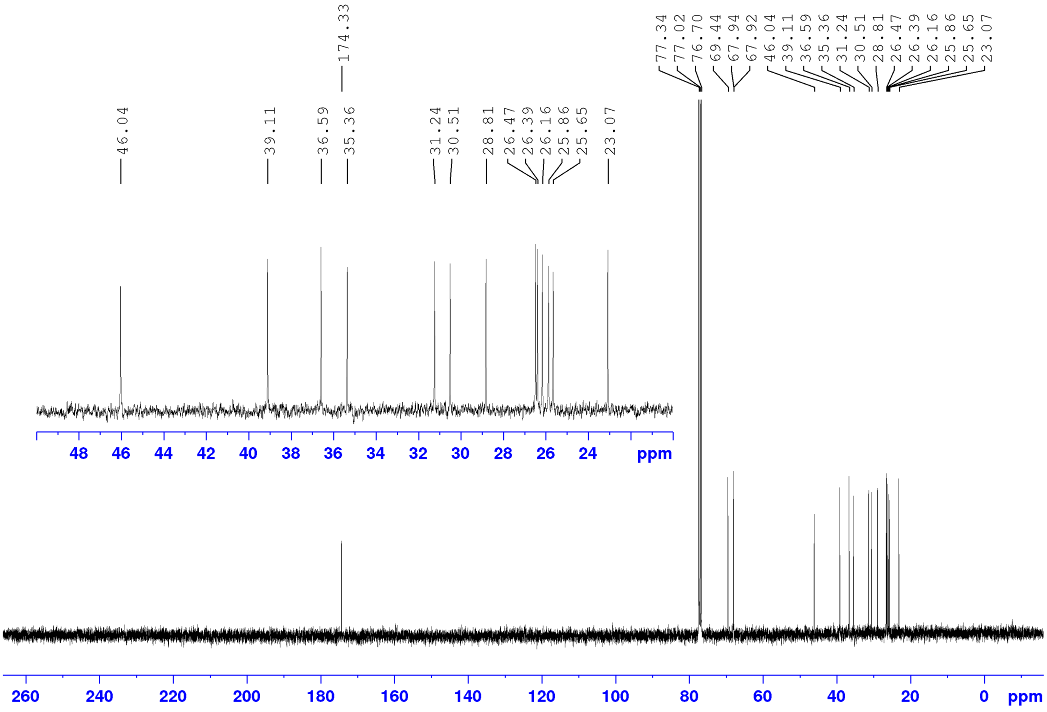
174.33
77.34 77.02 76.70 69.44 67.94 67.92 46.04 39.11 36.59 35.36 31.24 30.51 28.81 26.47 26.39 26.16 25.86 25.65 23.07
ppm

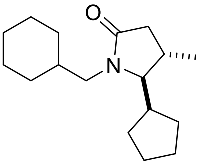

4o

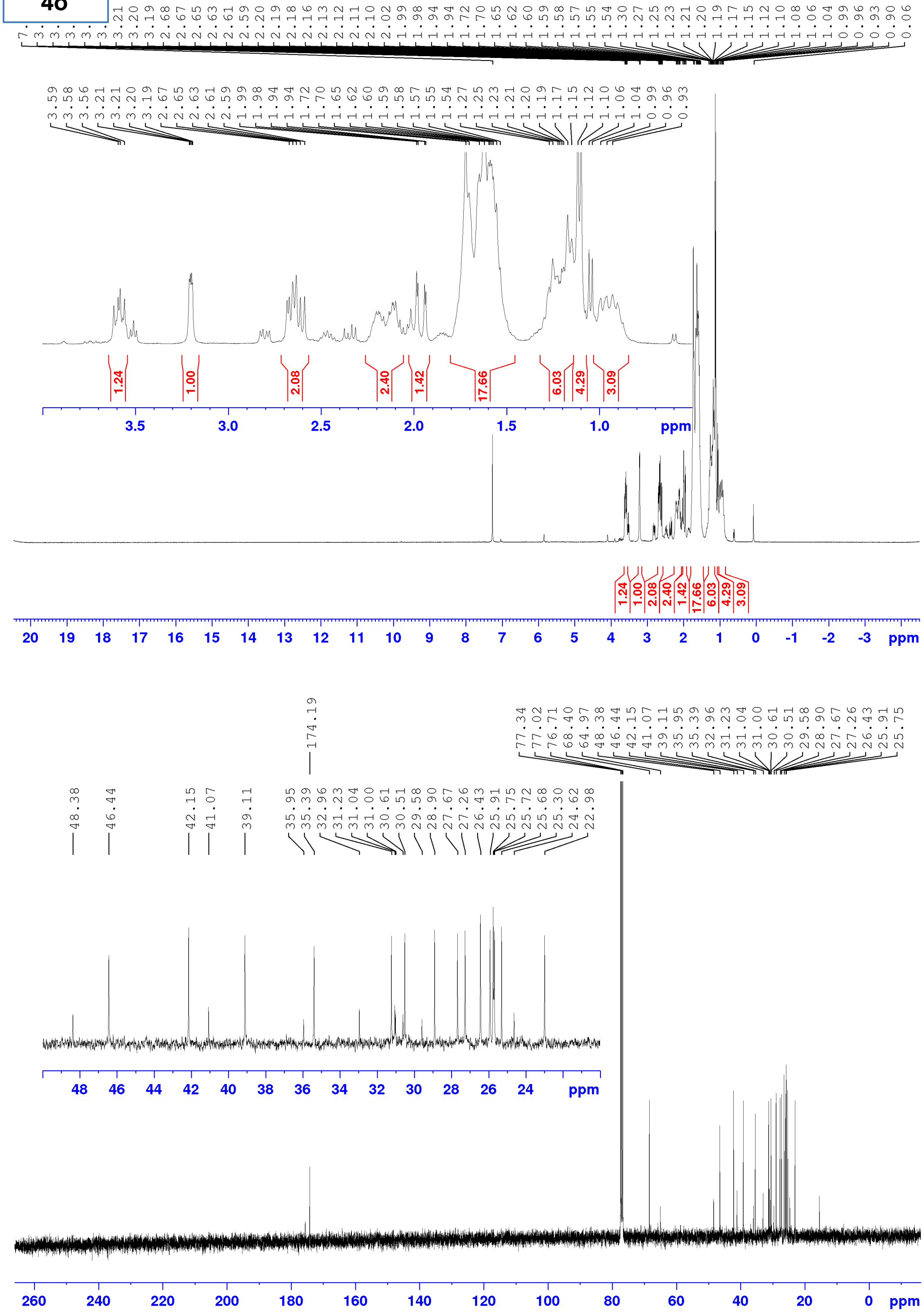

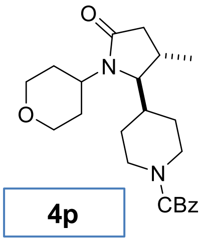

4p

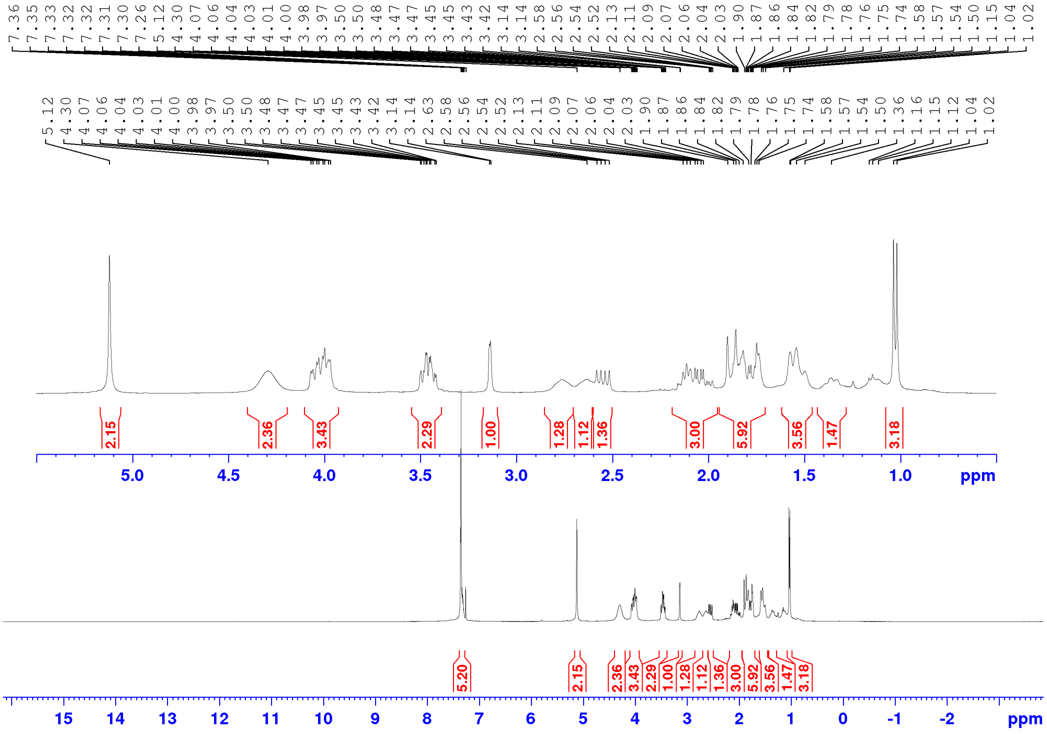

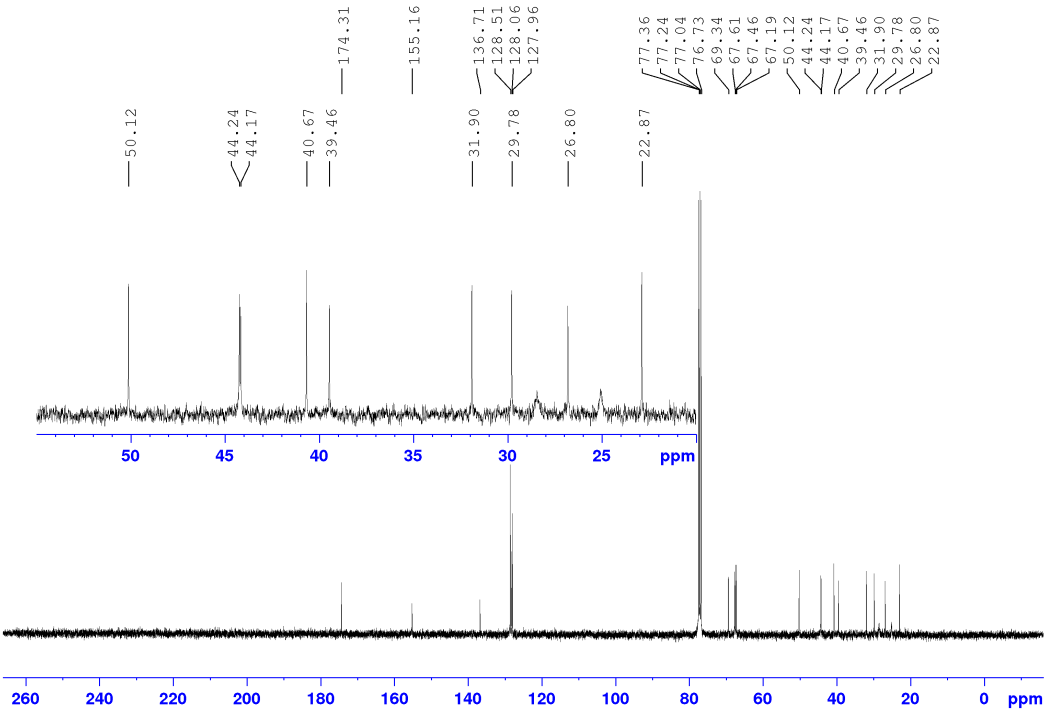

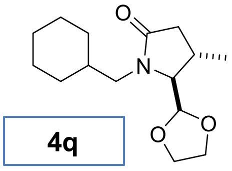

4q

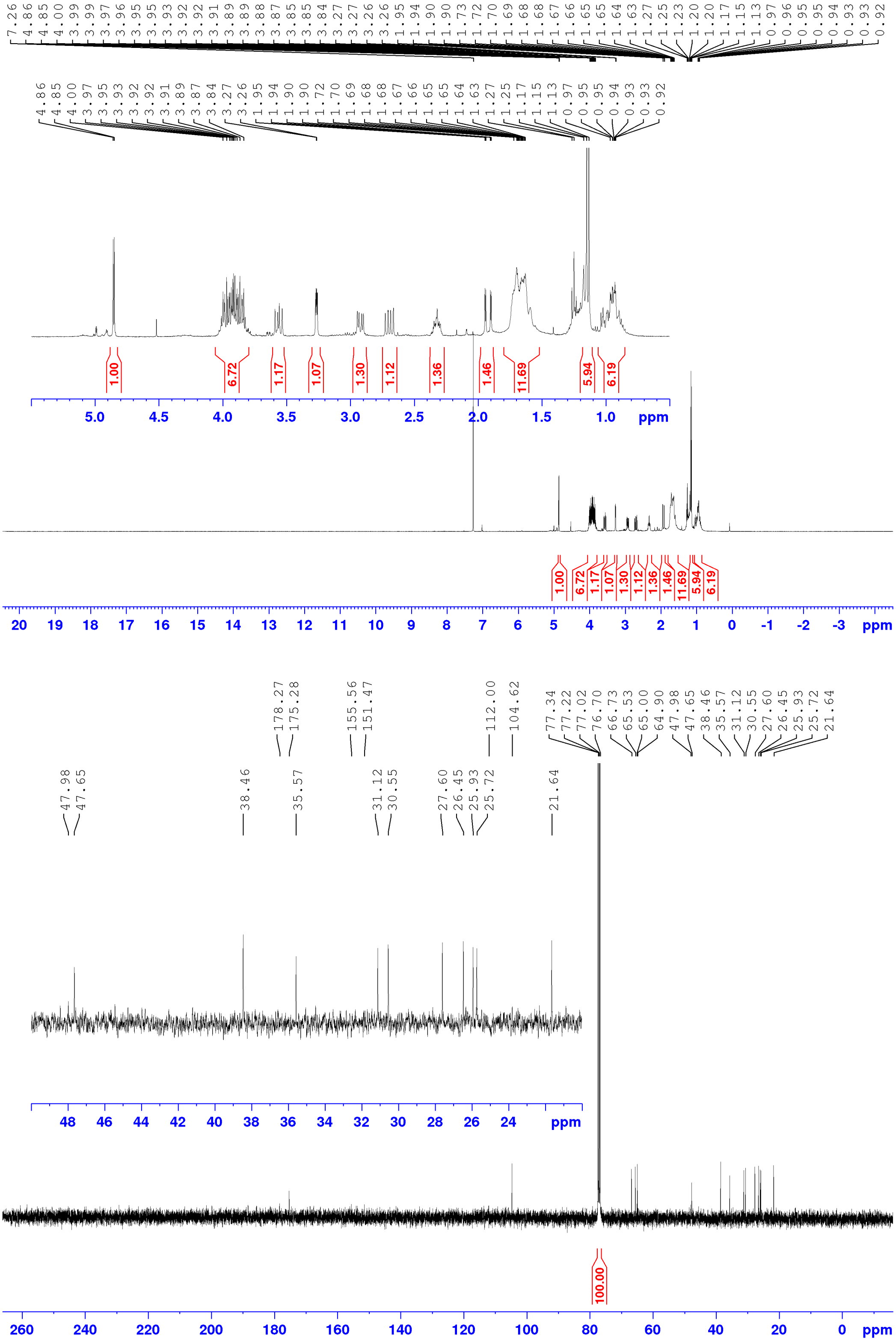

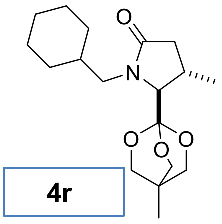

4r

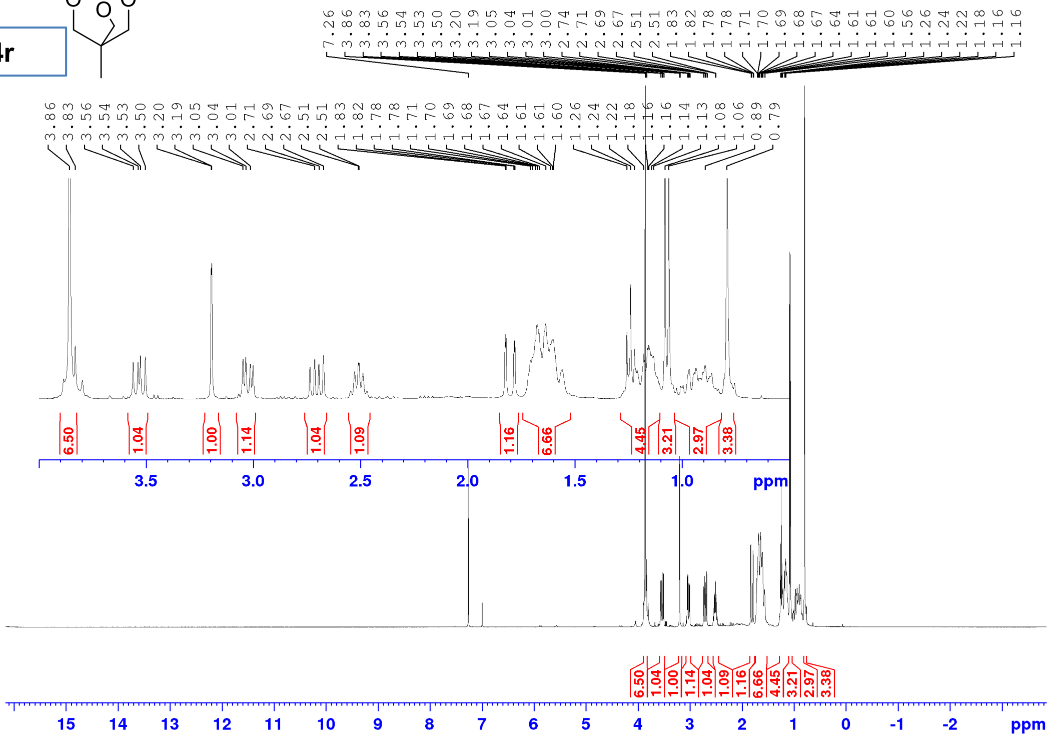

7.26 3.86 3.83 3.56 3.54 3.53 3.50 3.20 3.19 3.05 3.04 3.01 3.00 2.74 2.71 2.69 2.67 2.51 2.51 1.83 1.82 1.78 1.71 1.70 1.69 1.68 1.67 1.64 1.61 1.61 1.60 1.56 1.26 1.24 1.22 1.18 1.16 1.16
6.50 1.04 1.00 1.14 1.04 1.09 1.16 6.66 4.45 3.21 2.97 3.38
ppm
ppm

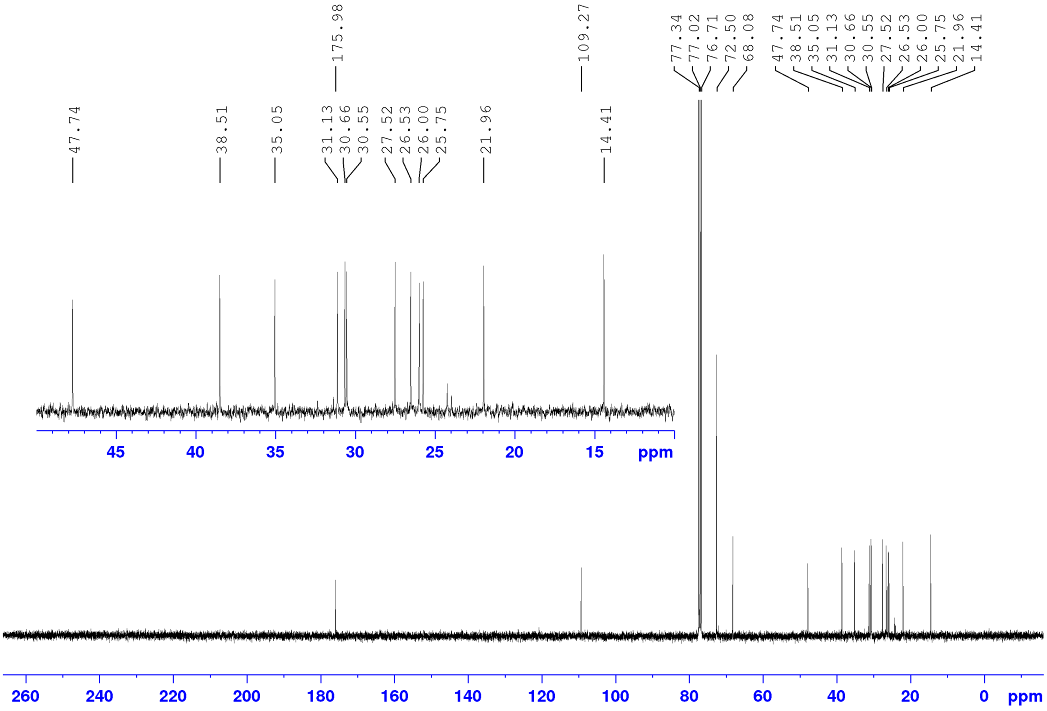

175.98 109.27 77.34 77.02 76.71 72.50 68.08 47.74 38.51 35.05 31.13 30.66 30.55 27.52 26.53 26.00 25.75 21.96 14.41
ppm
ppm

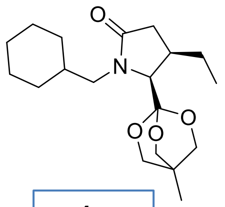
O
N
O
O
O

4s

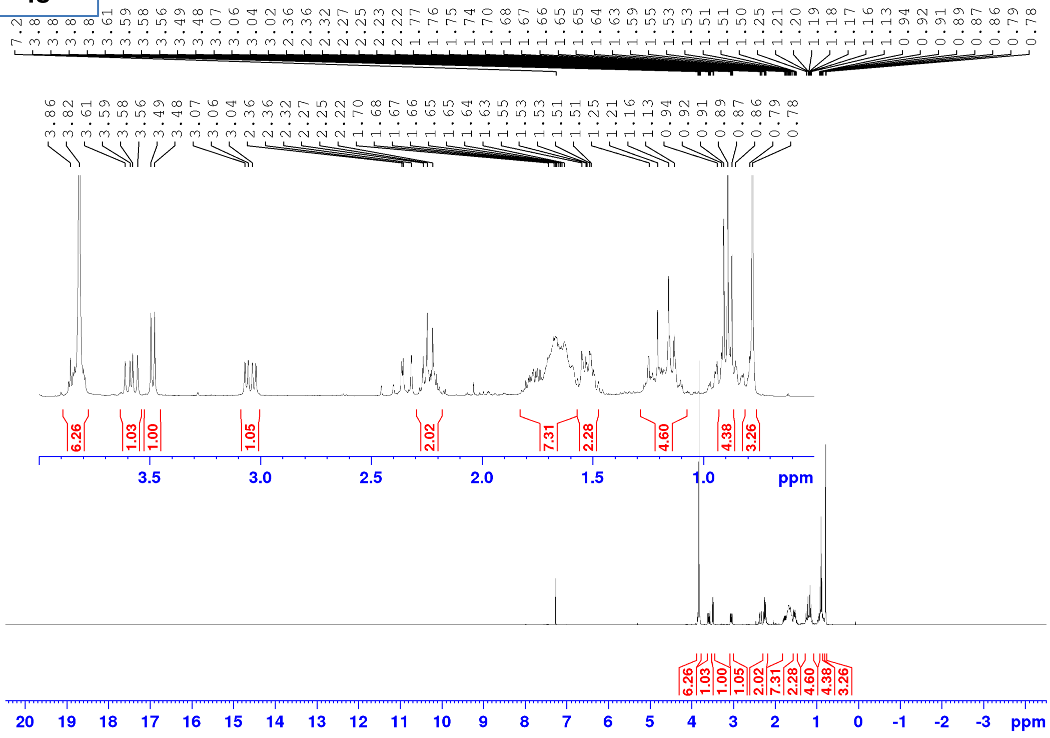
3.86 3.82 3.61 3.59 3.58 3.56 3.49 3.48 3.07 3.06 3.04 2.36 2.32 2.27 2.25 2.22 1.70 1.68 1.67 1.66 1.65 1.65 1.64 1.63 1.55 1.53 1.53 1.51 1.51 1.25 1.21 1.16 1.13 0.94 0.92 0.91 0.89 0.87 0.86 0.79 0.78
6.26 1.03 1.00 1.05 2.02 7.31 2.28 4.60 4.38 3.26
3.5 3.0 2.5 2.0 1.5 1.0 ppm
20 19 18 17 16 15 14 13 12 11 10 9 8 7 6 5 4 3 2 1 0 -1 -2 -3 ppm

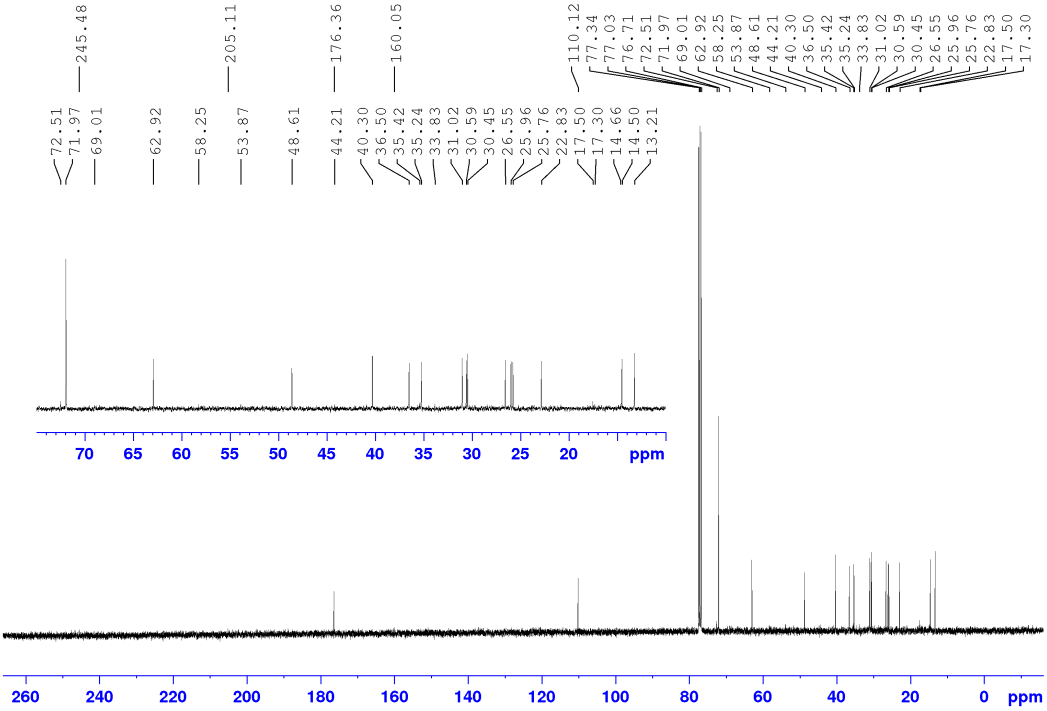
245.48 205.11 176.36 160.05 110.12 77.34 77.03 76.71 72.51 71.97 69.01 62.92 58.25 53.87 48.61 44.21 40.30 36.50 35.42 35.24 33.83 31.02 30.59 30.45 26.55 25.96 25.76 22.83 17.50 17.30
72.51 71.97 69.01 62.92 58.25 53.87 48.61 44.21 40.30 36.50 35.42 35.24 33.83 31.02 30.59 30.45 26.55 25.96 25.76 22.83 17.50 17.30 14.66 14.50 13.21
70 65 60 55 50 45 40 35 30 25 20 ppm
260 240 220 200 180 160 140 120 100 80 60 40 20 0 ppm

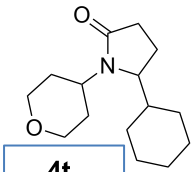
O
N
O

4t

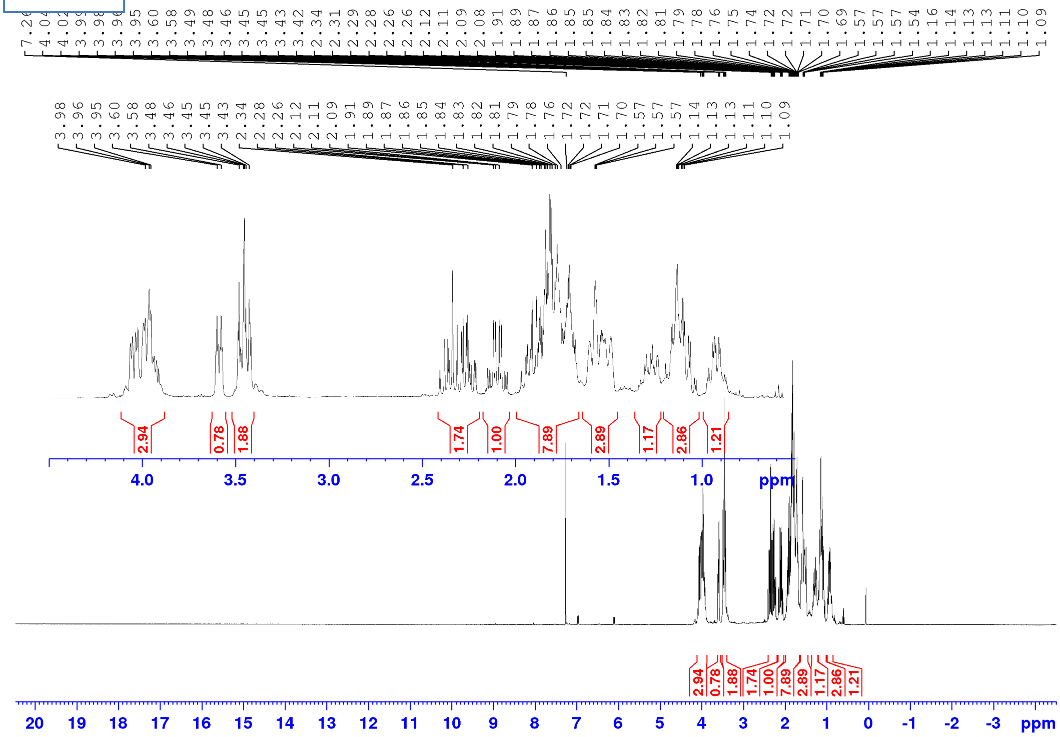
3.98
3.96
3.95
3.60
3.58
3.48
3.46
3.45
3.45
3.43
2.34
2.28
2.26
2.12
2.11
2.09
1.91
1.89
1.87
1.86
1.85
1.84
1.83
1.82
1.81
1.79
1.78
1.76
1.72
1.72
1.71
1.70
1.57
1.57
1.57
1.14
1.13
1.13
1.11
1.10
1.09
2.94
0.78
1.88
1.74
1.00
7.89
2.89
1.17
2.86
1.21
4.0
3.5
3.0
2.5
2.0
1.5
1.0
ppm
20
19
18
17
16
15
14
13
12
11
10
9
8
7
6
5
4
3
2
1
0
-1
-2
-3
ppm

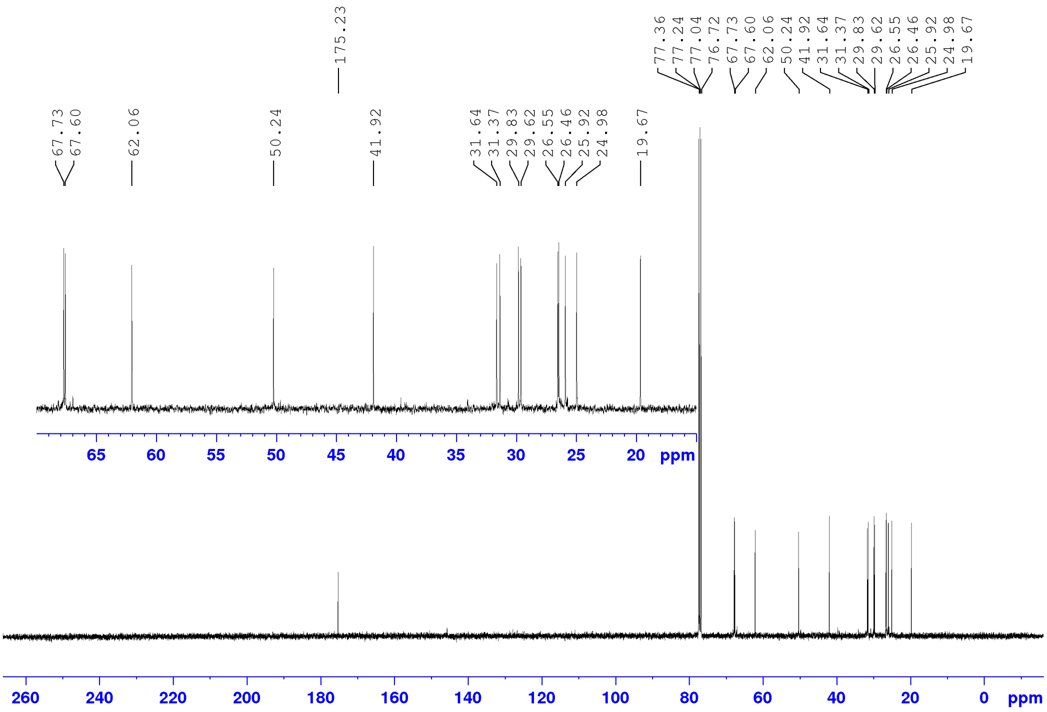
175.23
77.36
77.24
77.04
76.72
67.73
67.60
62.06
50.24
41.92
31.64
31.37
29.83
29.62
26.55
26.46
25.92
24.98
19.67
65
60
55
50
45
40
35
30
25
20
ppm
260
240
220
200
180
160
140
120
100
80
60
40
20
0
ppm

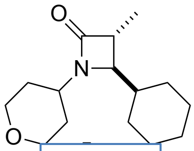

5t

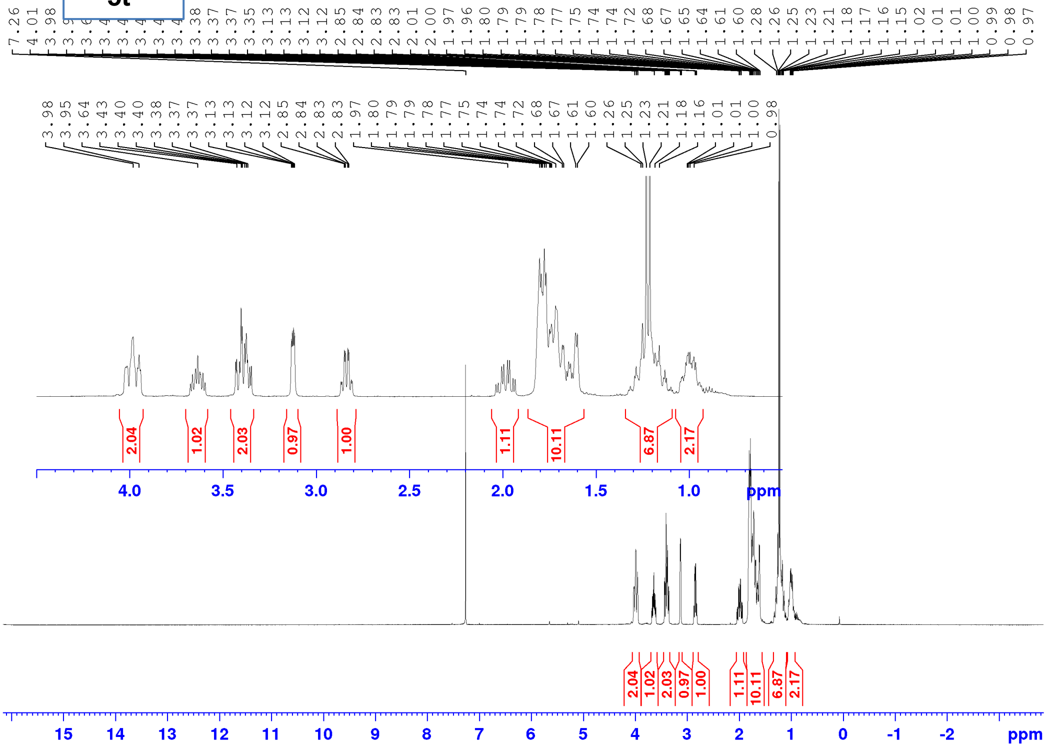
7.26
4.01
3.98
3.38
3.37
3.37
3.35
3.13
3.13
3.12
3.12
2.85
2.84
2.83
2.83
2.01
2.00
1.97
1.96
1.80
1.79
1.79
1.78
1.77
1.75
1.74
1.74
1.72
1.68
1.67
1.65
1.64
1.61
1.60
1.28
1.26
1.25
1.23
1.21
1.18
1.17
1.16
1.15
1.02
1.01
1.01
1.00
0.99
0.98
0.97
3.98
3.95
3.64
3.43
3.40
3.40
3.38
3.37
3.37
3.13
3.12
3.12
2.85
2.84
2.83
2.83
1.97
1.80
1.79
1.79
1.78
1.77
1.75
1.74
1.74
1.72
1.68
1.67
1.61
1.60
1.26
1.25
1.23
1.21
1.18
1.16
1.01
1.01
1.00
0.98
2.04
1.02
2.03
0.97
1.00
1.11
10.11
6.87
2.17
4.0
3.5
3.0
2.5
2.0
1.5
1.0
ppm
15
14
13
12
11
10
9
8
7
6
5
4
3
2
1
0
-1
-2
ppm

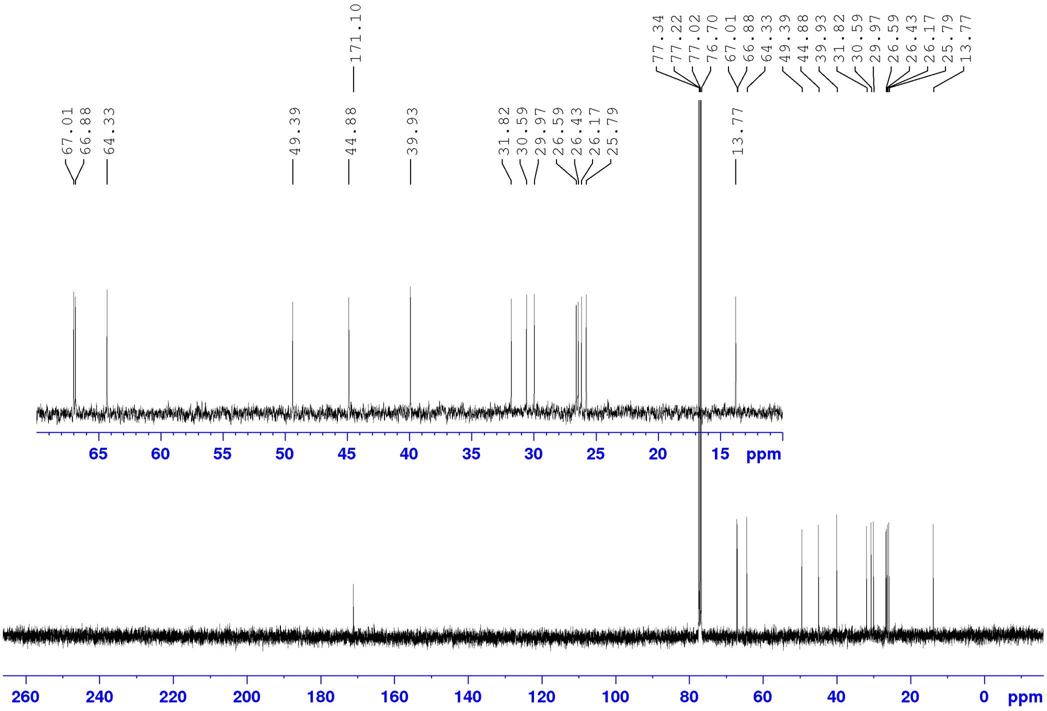
77.34
77.22
77.02
76.70
67.01
66.88
64.33
49.39
44.88
39.93
31.82
30.59
29.97
26.59
26.43
26.17
25.79
13.77
171.10
67.01
66.88
64.33
49.39
44.88
39.93
31.82
30.59
29.97
26.59
26.43
26.17
25.79
13.77
65
60
55
50
45
40
35
30
25
20
15
ppm
260
240
220
200
180
160
140
120
100
80
60
40
20
0
ppm

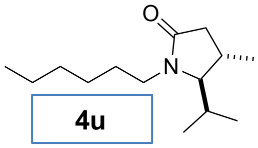

4u

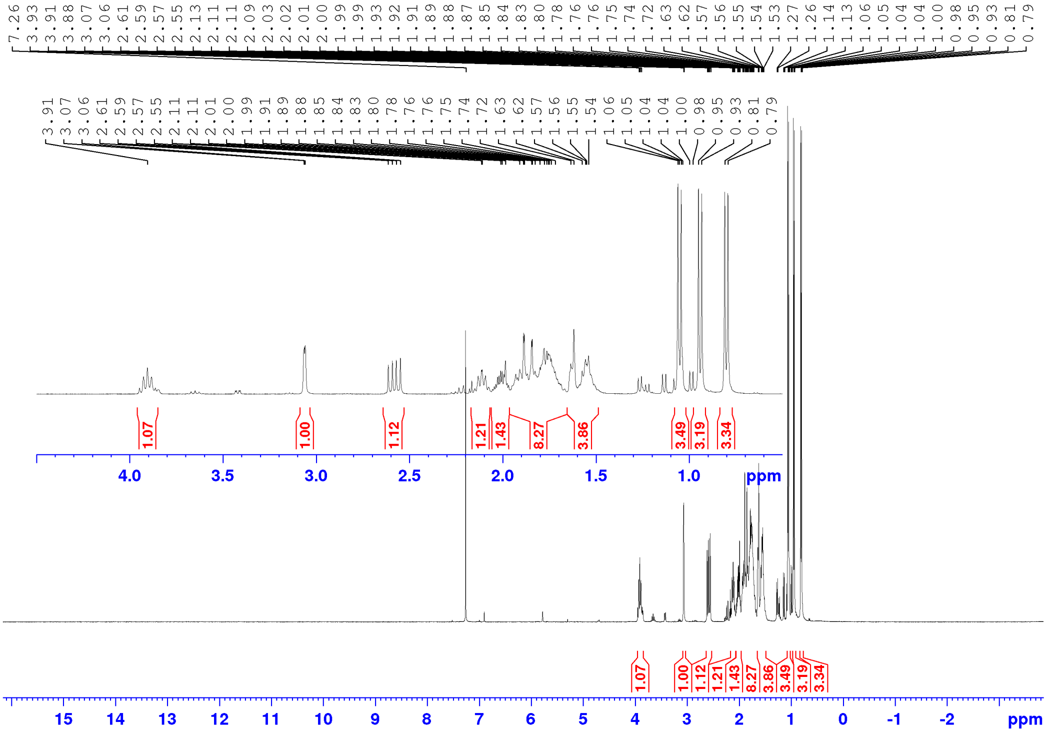
7.26 3.93 3.91 3.88 3.07 3.06 2.61 2.59 2.57 2.55 2.13 2.11 2.11 2.09 2.03 2.02 2.01 2.00 1.99 1.99 1.93 1.92 1.91 1.89 1.88 1.87 1.85 1.84 1.83 1.80 1.78 1.76 1.76 1.75 1.74 1.72 1.63 1.62 1.57 1.56 1.55 1.54 1.53 1.27 1.26 1.14 1.13 1.06 1.05 1.04 1.04 1.00 0.98 0.95 0.93 0.81 0.79
1.07 1.00 1.12 1.21 1.43 8.27 3.86 3.49 3.19 3.34
4.0 3.5 3.0 2.5 2.0 1.5 1.0 ppm
15 14 13 12 11 10 9 8 7 6 5 4 3 2 1 0 -1 -2 ppm

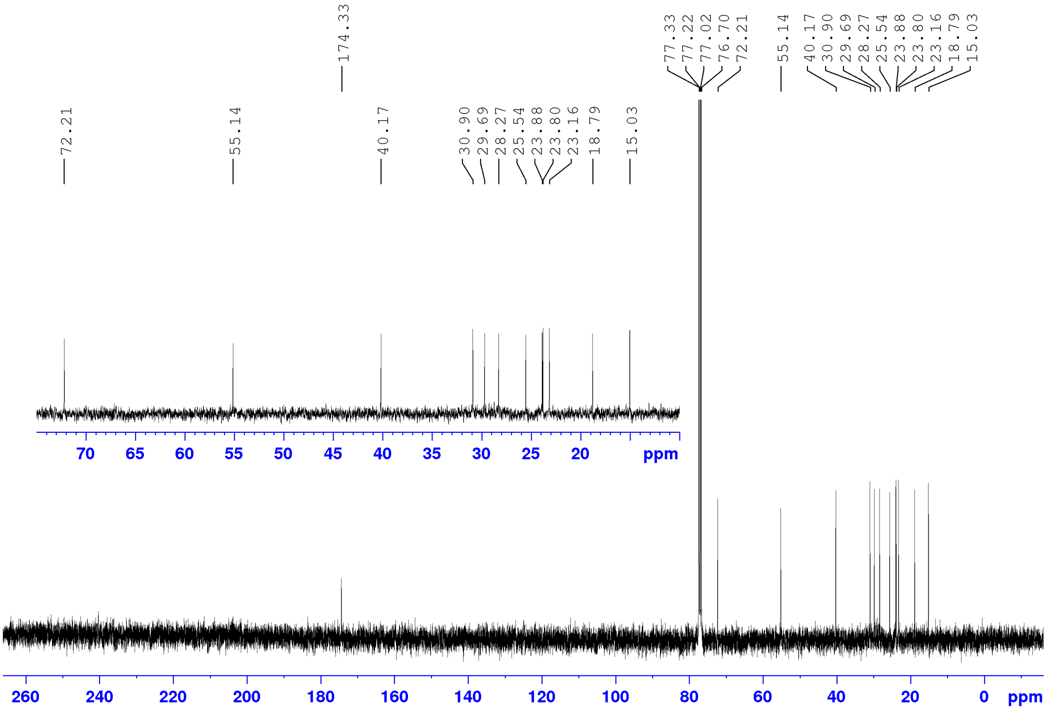
174.33
77.33 77.22 77.02 76.70 72.21
55.14
40.17 30.90 29.69 28.27 25.54 23.88 23.80 23.16 18.79 15.03
70 65 60 55 50 45 40 35 30 25 20 ppm
260 240 220 200 180 160 140 120 100 80 60 40 20 0 ppm

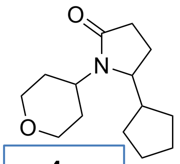

4v

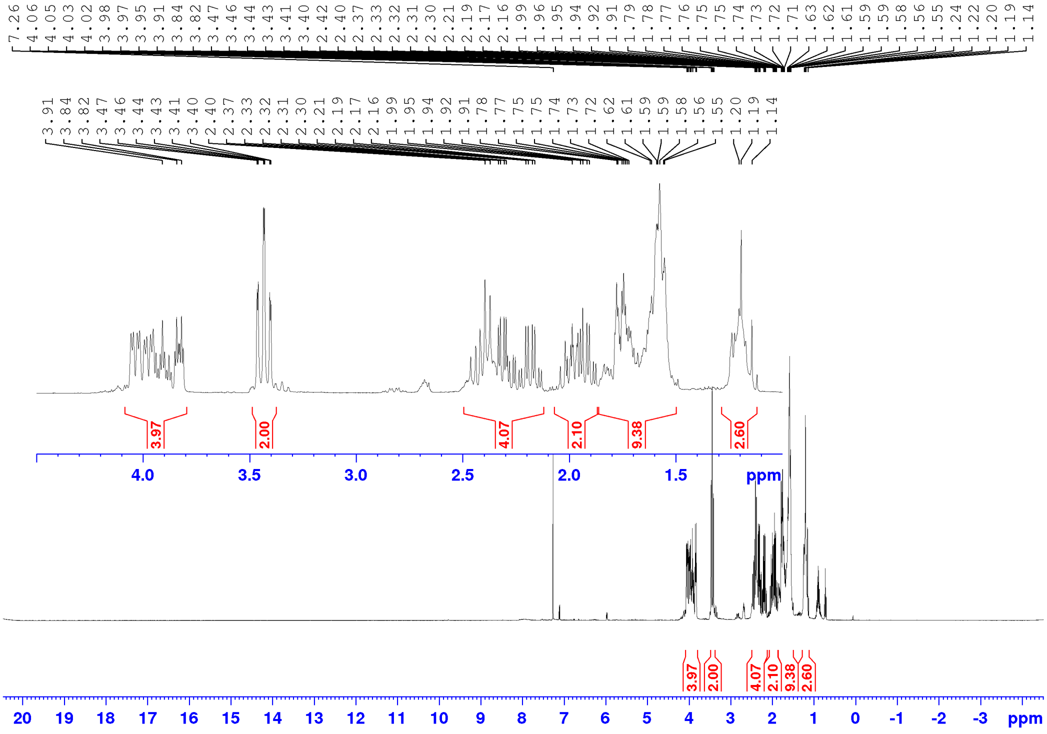

7.26 4.06 4.05 4.03 4.02 3.98 3.97 3.95 3.91 3.84 3.82 3.47 3.46 3.44 3.43 3.41 3.40 2.42 2.40 2.37 2.33 2.32 2.31 2.30 2.21 2.19 2.17 2.16 1.99 1.96 1.95 1.94 1.92 1.91 1.79 1.78 1.77 1.76 1.75 1.75 1.74 1.73 1.72 1.71 1.63 1.62 1.61 1.59 1.59 1.58 1.56 1.55 1.24 1.22 1.20 1.19 1.14
3.91 3.84 3.82 3.47 3.46 3.44 3.43 3.41 3.40 2.40 2.37 2.33 2.32 2.31 2.30 2.21 2.19 2.17 2.16 1.99 1.95 1.94 1.92 1.91 1.78 1.77 1.75 1.75 1.74 1.73 1.72 1.62 1.61 1.59 1.59 1.58 1.56 1.55 1.20 1.19 1.14
3.97 2.00 4.07 2.10 9.38 2.60
4.0 3.5 3.0 2.5 2.0 1.5 ppm
20 19 18 17 16 15 14 13 12 11 10 9 8 7 6 5 4 3 2 1 0 -1 -2 -3 ppm

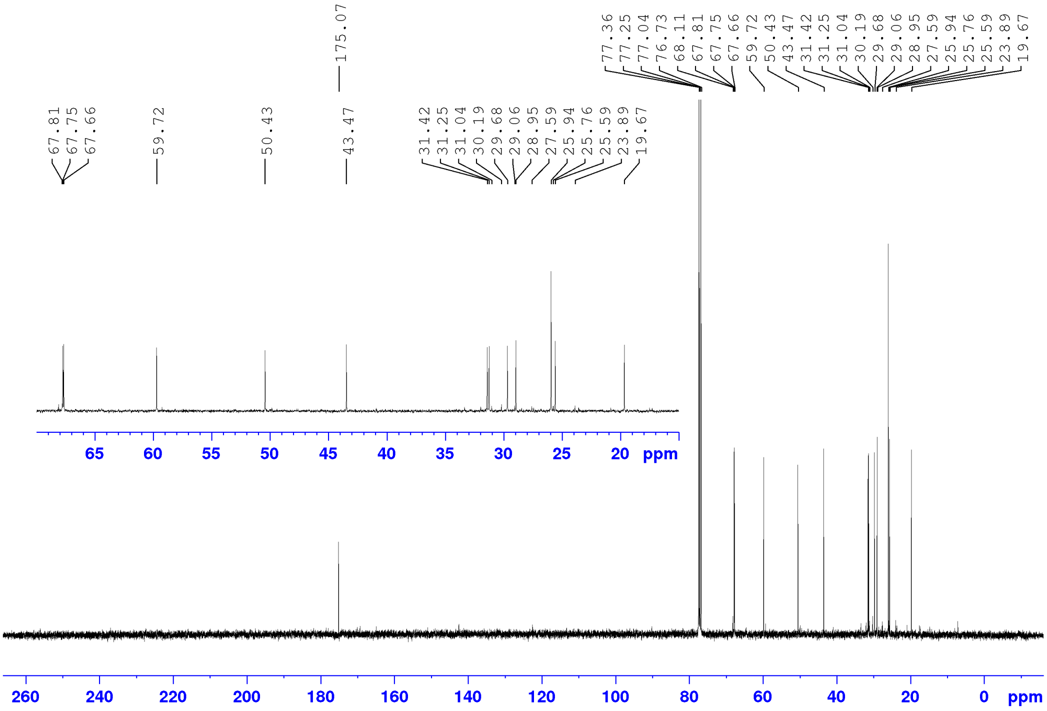

175.07
77.36 77.25 77.04 76.73 68.11 67.81 67.75 67.66 59.72 50.43 43.47 31.42 31.25 31.04 30.19 29.68 29.06 28.95 27.59 25.94 25.76 25.59 23.89 19.67
67.81 67.75 67.66 59.72 50.43 43.47 31.42 31.25 31.04 30.19 29.68 29.06 28.95 27.59 25.94 25.76 25.59 23.89 19.67
65 60 55 50 45 40 35 30 25 20 ppm
260 240 220 200 180 160 140 120 100 80 60 40 20 0 ppm

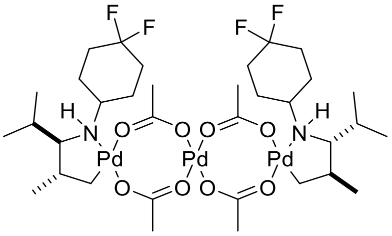

1f-int-I

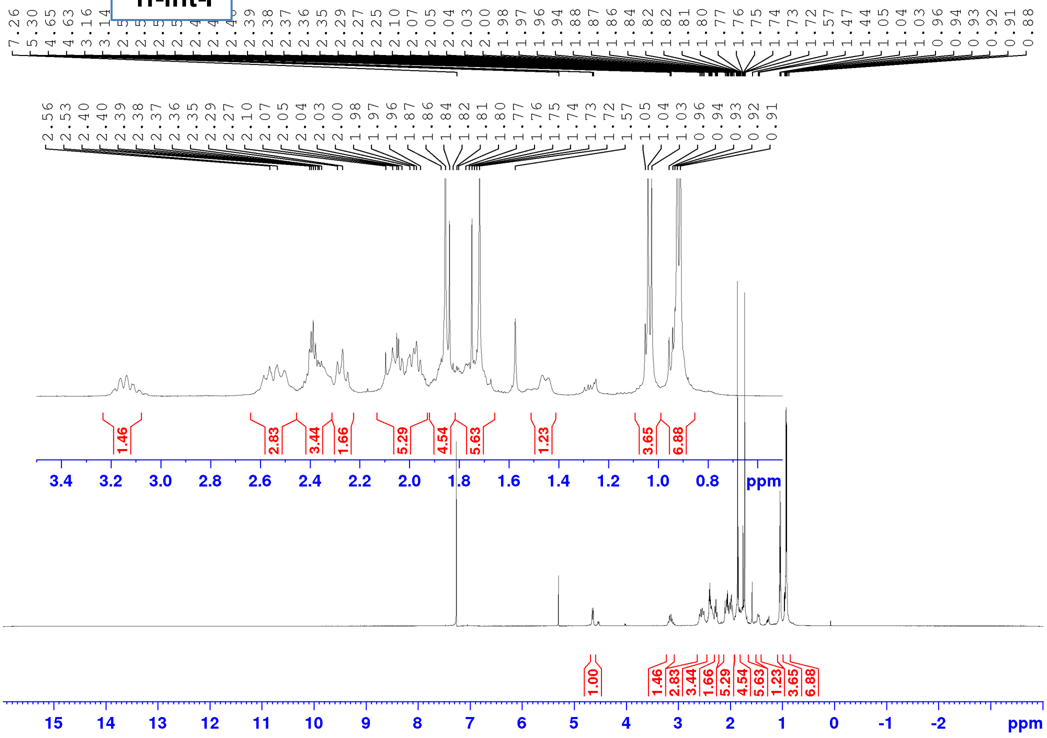
7.26 5.30 4.65 4.63 3.16 3.14 2.39 2.38 2.37 2.36 2.35 2.29 2.27 2.25 2.10 2.07 2.05 2.04 2.03 2.00 1.98 1.97 1.96 1.94 1.88 1.87 1.86 1.84 1.82 1.82 1.81 1.80 1.77 1.76 1.75 1.74 1.73 1.72 1.57 1.47 1.44 1.05 1.04 1.03 0.96 0.94 0.93 0.92 0.91 0.88
2.56 2.53 2.40 2.40 2.39 2.38 2.37 2.36 2.35 2.29 2.27 2.10 2.07 2.05 2.04 2.03 2.00 1.98 1.97 1.96 1.87 1.86 1.84 1.82 1.81 1.80 1.77 1.76 1.75 1.74 1.73 1.72 1.57 1.05 1.04 1.03 0.96 0.94 0.93 0.92 0.91
1.46 2.83 3.44 1.66 5.29 4.54 5.63 1.23 3.65 6.88
3.4 3.2 3.0 2.8 2.6 2.4 2.2 2.0 1.8 1.6 1.4 1.2 1.0 0.8 ppm
1.00 1.46 2.83 3.44 1.66 5.29 4.54 5.63 1.23 3.65 6.88
15 14 13 12 11 10 9 8 7 6 5 4 3 2 1 0 -1 -2 ppm

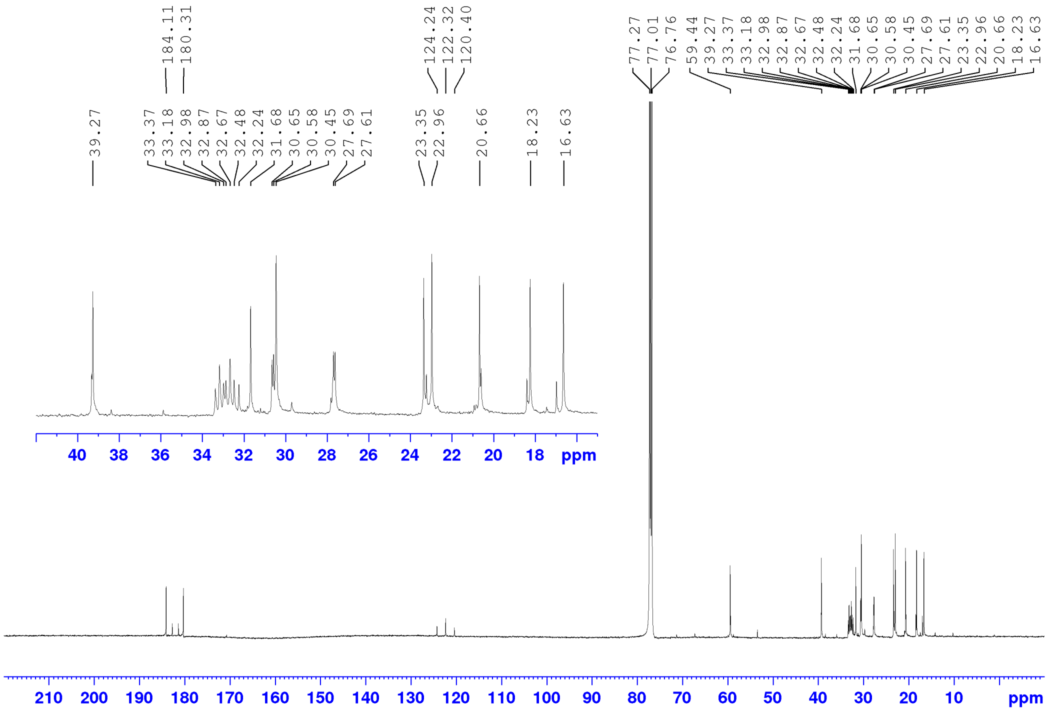
184.11 180.31 124.24 122.32 120.40 77.27 77.01 76.76 59.44 39.27 33.37 33.18 32.98 32.87 32.67 32.48 32.24 31.68 30.65 30.58 30.45 27.69 27.61 23.35 22.96 20.66 18.23 16.63
39.27 33.37 33.18 32.98 32.87 32.67 32.48 32.24 31.68 30.65 30.58 30.45 27.69 27.61 23.35 22.96 20.66 18.23 16.63
40 38 36 34 32 30 28 26 24 22 20 18 ppm
210 200 190 180 170 160 150 140 130 120 110 100 90 80 70 60 50 40 30 20 10 ppm

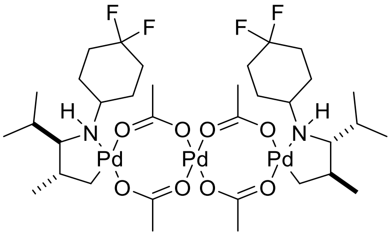
F
F
F
F
H
N
H
N
O
O
O
O
Pd
Pd
Pd
O
O
O
O

1f-int-I

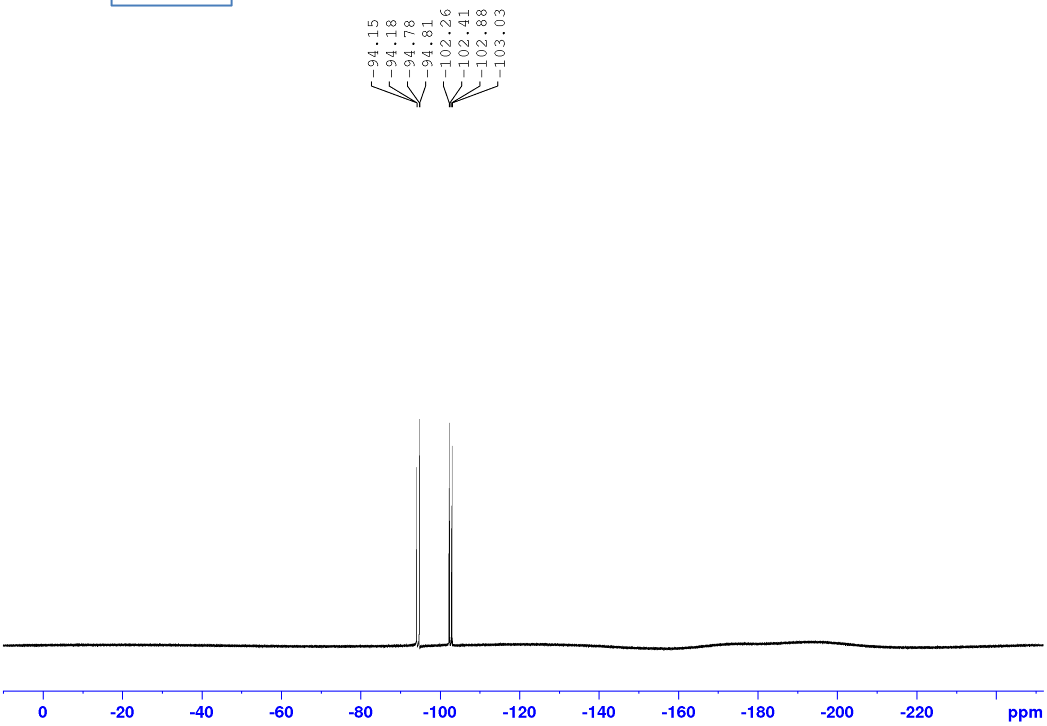
-94.15
-94.18
-94.78
-94.81
-102.26
-102.41
-102.88
-103.03
0
-20
-40
-60
-80
-100
-120
-140
-160
-180
-200
-220
ppm

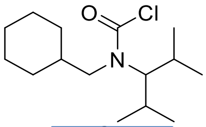

6

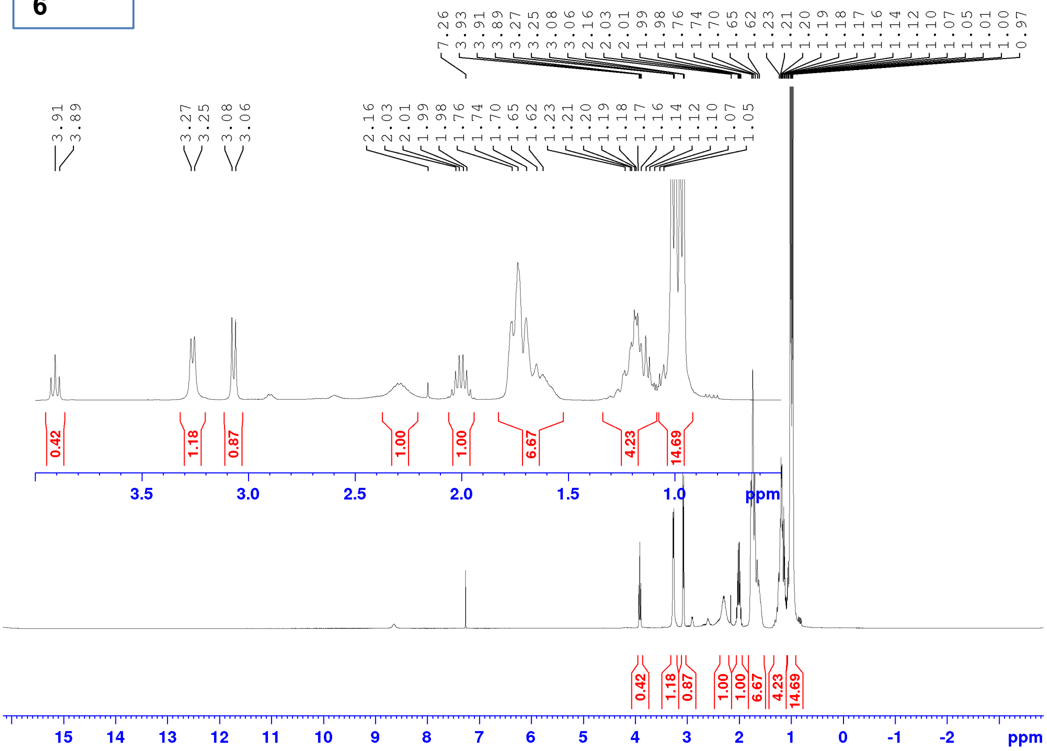

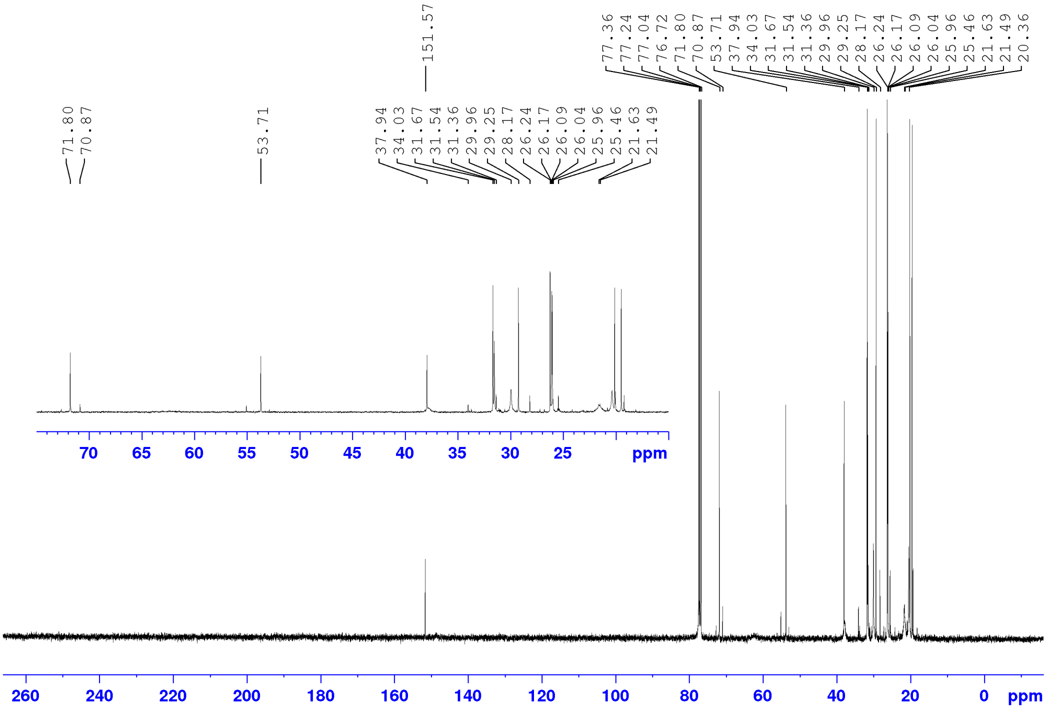

Supplement: Supplementary file 1 [file SC-009-C8SC02855A-s001.pdf]
